# 75th Congress of the Italian Society of Pediatrics: Bologna, Italy. 29 May - 1 June 2019

**DOI:** 10.1186/s13052-019-0746-3

**Published:** 2019-12-18

## A1 Complementary feeding in preterm infants

### Arianna Aceti (arianna.aceti2@unibo.it)

#### Department of Medical and Surgical Sciences, University of Bologna, Bologna, Italy

Optimal nutrition in the first 1000 days, from conception to the second year of life, has the potential to shape the individual health status during both childhood and adult life. This is particularly relevant to preterm infants, whose intrinsic immaturity makes nutritional management a daily challenge for the neonatologist.

Very little attention has been paid by scientific research to complementary feeding (CF) for preterm infants, and both the European [1] and the Italian [2] guidelines are specifically intended to guide the introduction of solid foods to “healthy term infants”.

A recent survey promoted by the Italian Society of Pediatrics, conducted among primary care paediatricians, has documented a wide variability in clinicians’ attitude towards timing of CF introduction and type of foods proposed to start CF [3].

As for timing, CF introduction might theoretically follow two different criteria: a 5 kg weight cut-off was originally proposed in the mid-90s in the UK (COMA report [4]); however, such a minimum weight does not take into account the infants’ development and can be reached very late by infants born extremely preterm. For this reason, a second criterion, which takes into account infants’ age and maturation, has been later proposed by dieticians’ working groups and professional associations in the UK [5]: a temporal window between 5 and 8 months uncorrected age has been identified as the time when virtually all former preterm infants should have acquired the developmental skills which allow the consumption of foods other than milk, such as the progressive disappearance of the protrusion reflex of the tongue, the reduction of reflexive suck in favour of lateral tongue movements, and the gradual appearance of lip seal. Furthermore, this time window is the optimal one for introducing new flavours and textures in term infants, and, even if it is not known how this sensitive period is affected by preterm birth, it is highly likely that the later preterm infants are introduced to new flavours and textures, the less likely they are to accept a wide variety of foods.

Even if no specific guideline is available, there is consensus that the introduction of CF in preterm infants should be strictly individualized, and that the timing should be guided more by the infant’s developmental acquisitions than by nutritional issues. Nevertheless, given the intrinsic risk for preterm infants of extrauterine growth retardation, a careful choice of high-protein, energy- and nutrient-dense solid foods should be performed.

**References**

1. Fewtrell M, Bronsky J, Campoy C, Domellöf M, Embleton N, Fidler Mis N, et al. Complementary Feeding: a position paper by the European Society for Paediatric Gastroenterology, Hepatology, and Nutrition (ESPGHAN) Committee on Nutrition. J Pediatr Gastroenterol Nutr. 2017;64:119–132.

2. Alvisi P, Brusa S, Alboresi S, Amarri S, Bottau P, Cavagni G, et al. Recommendations on complementary feeding for healthy, full-term infants. Ital J Pediatr. 2015;41:36.

3. Baldassarre ME, Di Mauro A, Pedico A, Rizzo V, Capozza M, Meneghin F, et al. Weaning time in preterm infants: An audit of italian primary care paediatricians. Nutrients. 2018;10:1–6.

4. Weaning and the weaning diet. Report of the Working Group on the Weaning Diet of the Committee on Medical Aspects of Food Policy. Rep Health Soc Subj. (Lond). 1994;45:1–113.

5. King C. An evidence based guide to weaning preterm infants. Paediatr Child Health (Oxford). 2009;19:405–414.

## A2 The child with medical complexity

### Sergio Amarri^1^, Alice Ottaviani^2^

#### ^1^Division of Pediatrcis, ASMN, AUSL-IRCCS Reggio Emilia, Reggio Emilia, Italy; ^2^Fondazione MT, Chiantore Seragnoli, Bologna, Italy

##### **Correspondence:** Sergio Amarri (sergio.amarri@ausl.re.it)

Children with medical complexity (CMC), who may also be known as “complex chronic” or “medically complex”, have multiple significant chronic health problems that affect multiple organ systems and resulting functional limitations, high health care need or utilization, and often the need for or use of medical technology. Children and youth with special health care needs (CYSHCN), who require health and related services for a chronic physical, developmental, behavioural, or emotional condition beyond what is typically required for children, (5) have long been designated as a priority population of interest for health care policy. CMC, a subset of CYSHCN because of their extensive and costly health care use, are increasingly recognized as requiring additional and specific consideration from physicians, payers, and policymakers. Approximately 1% of children, most of whom are CMC, account for up to one-third of overall health care spending for children, an increasing percentage of paediatric hospitalizations, and recurrent hospital admissions. Evidence suggests that CMC have among the highest risk of all children for adverse medical, developmental, psychosocial, and family outcomes. CMC are also potentially eligible for paediatric palliative care (PPC). Emilia Romagna Region and Fondazione Isabella Seragnoli are working since 2012 to develop a regional model for PPC that will also include a paediatric hospice. To better organize the PPC network a survey was administered to the entire region health authority. The data were collected at individual level mostly through a set of closed questions, the results of the survey were used to investigate patients’ characteristics and complexity, health conditions and offer. Table 1 is summarising the main results:

Neurological diseases affect almost 50% of the patients, followed by congenital diseases (Figure 1). As expected and differently from adult palliative care, oncologic diseases only represent 4% of the patients. Prevalence results to be very heterogeneous among provinces, revealing a different sensibility and knowledge on the topic of PPC. Regional prevalence, which is 8.44 cases every 10.000 residents below 18, is lower as compared to other EU countries, which would strengthen the necessity of spreading the knowledge of PPC.

Patient’s complexity is also demonstrated by the high percentage of devices and supports needed. In fact, on average 32% of the patients has a gastrostomy, 22% has respiratory support and 15% of the patients does have both gastrostomy and respiratory support.

**Table 1 (abstract A2).** Personal Information - Main results

Province	Patients at 31/12/2017	Prevalence (Cases every 10.000 residents <18)	Average age
Bologna	170	10,81	8,17
Forlì-Cesena	21	3,31	11,38
Ferrara	20	4,31	13,00
Modena	100	8,41	8,93
Parma	67	9,39	10,99
Piacenza	38	8,65	11,82
Ravenna	70	11,61	9,10
Reggio-Emilia	70	7,39	8,19
Rimini	45	8,12	9,91
**Total**	**601**	**8,44**	**9,35**

**Fig. 1 (abstract A2).**
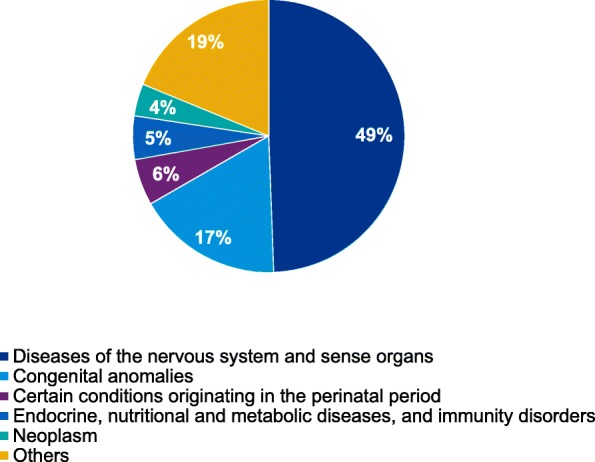
Main observed pathologies

## A3 IgE-mediated food allergies: from the traditional diagnostics to a molecular approach and new frontiers of treatment

### Stefania Arasi^1^, Giovanni B Pajno^2^, Alessandro G Fiocchi^1^

#### ^1^ Allergy Unit, Bambino Gesù Children's Hospital, IRCCS, Rome, Italy; ^2^Department of Pediatrics, Allergy Unit, University of Messina, Messina, Italy

##### **Correspondence:** Stefania Arasi (stefania.arasi@opbg.net)

IgE-mediated food allergy (FA) has been emerging as a significant health issue which may impair the disease-related quality of life, both of patients and their family members. It represents a major cause of life-threatening hypersensitivity reactions whose prevalence is increasing significantly. The diagnostic work-up of FA is traditionally based on a meticolous and detailed medical history collected with the parent and/or patient and cutaneous tests. As an alternative to the latter, it is possible to search the serum for specific IgE with commercial extract. However, these investigations are limited by the risk of false positive responses mainly due to the presence of cross-reactive allergens. The current gold standard for FA diagnosis is the oral food challenge, i.e. the administration of the suspected culprit food in clinical setting. It is highly demanding as risky for severe anaphylaxis and time- consuming. In some cases, to detect serum IgE against specific molecular allergenic components (component-resolved diagnosis, CRD) could be useful in order: to examine the possibility of cross-reactions or primary co-sensitizations; to determine the risk of clinical severity after allergen exposure; to investigate the risk of false positive results with extracts containing ubiquitous allergens ("Pan-alllergens"); to analyze cross-reactivity between pollens and food; and to evaluate the opportunity to perform oral food challenge(s) [1].

In terms of treatment, the current standard approach is relegated to strict dietary avoidance along with education on prompt recognition of symptoms, and emergency treatment of adverse reactions. However, several allergen specific- and nonspecific-therapies are under investigation. Allergen immunotherapy (AIT) is the only currently available treatment that targets the etio-pathophysiology and may modulate the natural history of IgE-mediated diseases. Based on a systematic review and metaanalysis [2], the European Allergy and Clinical Immunology Academy (EAACI) has recently stated that OIT can be recommended as a treatment option to increase the threshold of reaction in children with persistent cow’s milk, egg or peanut allergy [3]. However, there are still several gaps to be filled in the current body of evidence, including safety issues. In this perspective, omalizumab -an anti-IgE monoclonal antibody-, alone or combined with oral immunotherapy, might represent a promising treatment option [4,5]. In the near future, well-designed studies are awaited in order to overcome the current gaps in the evidence and furtherly promote implementation strategies with the final goal of a treatment tailored on each specific clinical sub-group.

**References**

1. Matricardi PM, Kleine-Tebbe J, Hoffmann HJ, Valenta R, Hilger C, Hofmaier , et al. EAACI Molecular Allergology User's Guide. Pediatr Allergy Immunol. 2016;27 Suppl 23:1-250.

2. Pajno GB, Fernandez-Rivas M, Arasi S, Roberts G, Akdis CA, Alvaro-Lozano M, et al. EAACI Guidelines on allergen immunotherapy: IgE-mediated food allergy. Allergy. 2018; 73(4):799-815.

3. Nurmatov U, Dhami S, Arasi S, Pajno GB, Fernandez-Rivas M, Muraro A, et al. Allergen immunotherapy for IgE-mediated food allergy: a systematic review and meta-analysis. Allergy. 2017;72:1133-1147.

4. Wood RA, Kim JS, Lindblad R, Nadeau K, Henning AK, Dawson P, et al. A randomized, double-blind, placebo-controlled study of omalizumab combined with oral immunotherapy for the treatment of cow's milk allergy. J Allergy Clin Immunol. 2016;137:1103-1110.

5. Fiocchi A, Artesani MC, Riccardi C, Mennini M, Pecora V, Fierro V, et al. Impact of omalizumab on food allergy in patients treated for asthma: a real-life study. J Allergy Clin Immunol Pract. 2019;7:1901-1909.

## A4 The pediatrician and the digital clinic

### Paolo Becherucci^1^, Marco Masoni^2^

#### ^1^Primary Pediatric Care Italian Society, Florence, Italy; ^2^Department of Experimental and Clinical Medicine, University of Florence, Florence, Italy

##### **Correspondence:** Paolo Becherucci (m.masoni@med.unifi.it)

Although Internet offers many opportunities to improve the provision of health services to citizens and at the same time to reduce costs, there are risks that physicians should know and not underestimate. When faced with difficulties from the use of Internet applications, the healthcare professionals adopt a spontaneous and empirical approach, often resorting to the help of colleagues if non-routine and unusual methods of use and situations emerge. To avoid simplistic behaviors that can put patients' health at risk, international organizations, such as the American Medical Association (AMA), the American College of Physician (ACP) and the British Medical Association (BMA), have produced guidelines/directives that provide a reference base to use. Among the many services offered by the Internet, the course focuses on e-mail and social media. Even if e-mail is a routine tool in healthcare, many don’t know how to manage it in a correct way. The use of e-mail will be explained distinguishing between two types of patient-physician communication: the first characterized by the absence of a preexisting face-to-face interaction, the other characterized by the presence of a preexisting contractual relationship between patient and physician. Physicians increasingly use social media to communicate with patients. In these interactions, healthcare professionals forget that Internet is a public space where it is important to not share patient’s confidential data and to have behaviors in line with a correct professional ethic. The features that hinder the use of WhatsApp in the communication with patients will also be discussed. The use of computer and electronic medical report are mandatory for the pediatrician of primary care. But some attentions are required in their use, principally for privacy problem. Also, we examine the resources for printing, scanning, storage in ambulatory work setting. The aim of the course is to help physicians to manage e-mail and social media in a suitable way; on the other hand, we will give advice so that the computer technology represents an aid and not a hindrance during the daily ambulatory work.

## A5 Telemedicine’s role in monitoring patients with rare and chronic disease: the Cystic Fibrosis model

### Sergio Bella, Clarissa Paglia

#### Cystic Fibrosis Unit, Bambino Gesù Children’s Hospital Rome, Italy

##### **Correspondence:** Sergio Bella (sergio.bella@opbg.net)

**Background**

The improvement of health care has led to a transformation of some acute to chronic diseases. The consequence was the appearance of co-morbidities not previously evident. This has increased the complexity of the follow-up with even an increase in accesses to the hospital. This has created the need, given the reduction of resources and therefore of beds to find an alternative system that would allow the patient to be able to remain "safe" at home. Telemonitoring is therefore developed, a branch of telemedicine to monitor vital parameters for a pathologist and subsequently also those concerning co-morbidities.

**Material and method**

Let us then move on to the Oxitel tool, which allowed a rudimentary remote monitoring of platforms that allow us to know the patient's state of health in a precise manner. The next step was to make the platforms easier to use and portable. The platform of which I speak to you today is precisely the last born. It allows both telemonitoring and remote diagnostics to be performed. All devices are CE certified.

**Results**

We expect, as in other projects carried out by us, a rationalization of access to the hospital, less decay of vital parameters and in particular of lung function, and finally an empowerment of the patient who will learn to better manage his pathology.

**Conclusion**

Patients will take measurements from their home that will be downloaded and interpreted by the attending physician, or by the doctor of the reference hospital center. There is the possibility of using the active 24/24 operations center which aims to carry out the triage and then eventually alert the attending physician. This type of platform highlights the sudden evolution of technology that, in addition to leading to more appropriate and precise measurements, also leads to greater patient adherence to the home telemonitoring program for ease of use. Lastly, the decrease in incongruous accesses is no less important

## A6 eHealth and Nursing: the role of the nurse in the technological and cultural transformation of medicine

### Giada Bertaina, Alberto Lazzero

#### University of Turin, Italy

##### **Correspondence:** Giada Bertaina (giada.bertaina952@gmail.com)

This paper has been developed as an analysis and deployment of an innovative technology devoted to the support in nursing and medicine for patients. By means of an analysis of the Italian (North western area of the country) and French (ProvenceAlpes- Côte d'Azur region) hospitals, the research goal has been to explore how new technologies can be applied to medicine. The activity included the analysis of the knowledge and the education of nurses referring to the implementation and application of telemedicine and telenursing technologies, to better support patients in their treatment. It is highlighted how the cultural and traditional aspects of resources’ education plays an important role to the application of eHealth as a whole. The report includes a presentation of how the telemedicine is applied in foreign countries, like United Kingdom. There the General Practice centers are adopting new technology (Apps and digitized medical records) for supporting the full treatment cycle of patients.

## A7 Lung ultrasound in infants with acute bronchiolitis

### Carlotta Biagi, Anna Mingozzi, Luca Pierantoni, Marcello Lanari

#### Pediatric Emergency Unit, Department of Medical and Surgical Sciences (DIMEC), St. Orsola-Malpighi Hospital, University of Bologna, Bilogna, Italy

##### **Correspondence:** Carlotta Biagi (carlottabiagi@yahoo.it)

**Background:** The variable course of acute bronchiolitis (AB) and the lack of current scoring systems that could highly predict the progression of this disease result in frequent hospital admission and misuse of diagnostic tests. Chest X-ray (CXR) is still performed in a high percentage of infants with AB, mainly to rule out concomitant pneumonia. In this context, lung ultrasound (LUS) is recently emerging like a point-of-care tool that could help the clinician in the management of multiple pulmonary diseases. The aim of this study was to assess the accuracy and reliability of LUS for the diagnosis of concomitant pneumonia in infants with AB and to evaluate the correlation between LUS findings and clinical severity of bronchiolitis. Moreover we calculate the inter-observer agreement between sonographers of different experience levels.

**Material and methods:** We enrolled infants admitted to our hospital in 2016-2018 with a diagnosis of AB and undergone CXR because of clinical suspicion of concomitant pneumonia. LUS was performed in each child by a novice pediatrician sonographer blinded to the patient's clinical, laboratory and CXR findings. Moreover, an exploratory analysis was done to calculate the inter-observer agreement between two sonographers (experienced radiologist and pediatrician) who independently performed LUS. The diagnosis of pneumonia was established by an expert clinician on the basis of the British Thoracic Society guidelines. The study was approved by the ethics committee of our institution, approval number 13/2016/O/Sper. Parents of all the eligible patients gave informed written consent.

**Results:** Ninety six hospitalized infants with AB were enrolled. A final diagnosis of concomitant pneumonia was made in 27 patients. Sensitivity and specificity of LUS for the diagnosis of pneumonia were 96.3% and 85.5% respectively, while CXR showed a sensitivity of 96.3% and specificity of 76.8%. Considering the patients with uncomplicated AB, a significant correlation emerged between the detection of subpleural consolidations and the need for oxygen therapy during the hospital stay. There was good to excellent agreement between the novice and expert sonographer.

**Conclusions:** This study shows the good accuracy of LUS in diagnosing pneumonia in infants with AB. Added benefit of LUS included high inter-observer agreement. Moreover, if confirmed in future studies, LUS may emerge as a useful technique to predict the need of supplemental oxygen in infants with bronchiolitis and to help clinician in the management of this disease.

## A8 Management of severe respiratory syncytial virus infections in the pediatric intensive care unit

### Paolo Biban, Marcella Gaffuri, Stefania Spaggiari, Laura Andaloro, Francesco Sacco, Rossella Frassoldati, Giuseppe Pagano, Alessandra Serra, Paolo Bonetti, Pierantonio Santuz

#### Department of Neonatal and Paediatric Critical Care, Verona University Hospital, Verona, Italy

##### **Correspondence:** Paolo Biban (paolo.biban@aovr.veneto.it)

Respiratory syncytial virus (RSV) is a common cause of hospital admission for respiratory tract infections [1]. Most frequently RSV may cause bronchiolitis, which could precipitate acute respiratory failure requiring intensive care and ventilatory support. According to a recent study, in about 140.000 children hospitalized for RSV-associated acute respiratory illness, up to 22% of these patients required admission to a pediatric intensive care unit (PICU).[2] Co-morbidities, such as prematurity, congenital heart disease, genetic abnormalities, chronic lung disease, sepsis or neuromuscular diseases, were important contributors for worse outcomes [2]. In a large study on children with severe community-acquired RSV infections, one-third of 604 patients were intubated and mechanically ventilated.[3] Co-morbidities and transfer from a peripheral hospital were associated with a higher rate of mechanical ventilation (MV) [3]. Interestingly, the increasing use of humidified high-flow nasal cannula (HFNC) oxygen and continuous positive airway pressure (CPAP) is changing the admission patterns and ventilation requirements for bronchiolitis [4,5]. Non-invasive ventilation (NIV) seems to be the first treatment choice in most units, with CPAP seemingly more effective than HFNC in reducing escalation toward invasive MV [6]. However, several patients fail to respond to NIV, requiring tracheal intubation and MV. Beside conventional MV and high-frequency ventilation (HFV), new ventilatory modalities have been tried in RSV bronchiolitis, showing promising results both in invasive and non-invasive scenarios. Interesting preliminary data have been reported on neurally adjusted ventilatory assist (NAVA), an unique mode of assisted MV that delivers inspiratory pressure proportionally to the electrical diaphragmatic activity [7]. In 11 children with severe RSV bronchiolitis failing CPAP, non-invasive intermittent positive-pressure ventilation was provided using NAVA, comparing asynchrony and ventilator response time with standard pressure support (PS) mode. Patient-ventilator inspiratory asynchronies and trigger delay were significantly lower in NAVA than in PS mode [8]. In another study in infants with severe bronchiolitis, NAVA NIV was associated with respiratory unloading during the transition from nasal CPAP to NAVA, showing a marked improvement of several work of breathing indices [9]. Finally, in 23 infants treated with invasive MV for severe bronchiolitis, NAVA had less trigger delay, improved ventilator response time and reduced work of breathing when compared with pneumatically triggered breaths [10]. In summary, RSV-associated respiratory illnesses still carry a high burden in the PICUs, in terms of morbility, bed-occupancy and costs. Further research is needed to validate the role of NAVA and other new ventilatory modes to reduce invasive MV in these patients, as well as lenght of PICU and hospital stay.

**References**

1. Florin TA, Plint AC, Zorc JJ. Viral bronchiolitis. Lancet. 2017;389:211–24.

2. Gupta P, Beam BW, Rettiganti M. Temporal trends of respiratory syncytial virus–associated hospital and ICU admissions across the United States. Pediatr Crit Care Med 2016;17:e343–e351.

3. Pham H, Thompson J, Wurzel D, Duke T. Ten years of severe respiratory syncytial virus infections in a tertiary paediatric intensive care unit. J Paediatr Child Health. 2019. [Epub ahead of print]

4. Franklin D, Fraser JF, Schibler A. Respiratory support for infants with bronchiolitis, a narrative review of the literature. Paediatr Respir Rev. 2010;30:16-24.

5. Goh CT, Kirby LJ, Schell DN, Egan JR. Humidified high-flow nasal cannula oxygen in bronchiolitis reduces need for invasive ventilation but not intensive care admission. J Paediatr Child Health. 2017;53:897-902.

6. Essouri S, Laurent M, Chevret L, Durand P, Ecochard E, Gajdos V, et al. Improved clinical and economic outcomes in severe bronchiolitis with pre-emptive nCPAP ventilatory strategy. Intensive Care Med. 2014;40:84–91.

7. Biban P, Serra A, Polese G, Soffiati M, Santuz P. Neurally adjusted ventilatory assist: a new approach to mechanically ventilated infants. J Matern Fetal Neonatal Med. 2010;23 Suppl 3: 38-40.

8. Baudin, F, Pouyau R, Cour-Andlauer F, Berthiller J, Robert D, Javouhey E. Neurally Adjusted Ventilator Assist (NAVA) reduces asynchrony during non-invasive ventilation for severe bronchiolitis. Pediatr Pulmonol. 2015;50:1320-7.

9. Baudin F, Emeriaud G, Essouri S, Beck J, Javouhey E, Guerin C. Neurally adjusted ventilatory assist decreases work of breathing during non-invasive ventilation in infants with severe bronchiolitis. Crit Care. 2019;23:120.

10. Clement KC, Thurman TL, Holt SJ, Heulitt MJ. Neurally triggered breaths reduce trigger delay and improve ventilator response times in ventilated infants with bronchiolitis. Intensive Care Med. 2011;37:1826–1832.

## A9 E-learning for hip ultrasound interpretation

### Salvatore Bonforte^1^, Francesco Guarnera^2^, Giuseppe Pappalardo^2^, Giuseppe Atti^1^

#### ^1^Ultrasound Study Group of the Italian Society of Pediatrics; ^2^Department of Mathematics and Computer Science, University of Catania, Catania, Italy

##### **Correspondence:** Salvatore Bonforte (salvo.bonforte@gmail.com)

We present a web application (URL: http://ecoanche.dmi.unict.it), implemented on the AMP (Apache/MySql/PHP https://en.wikipedia.org/wiki/LAMP_(software_bundle) platform and the MVC PHP Laravel framework (http://laravel.com), devised to carry out a remote continuing education programme, aimed at offering training in the reading and interpretation of neonatal ultrasound hip images. Developmental dysplasia of the hip (DDH) is the most frequent pathology of the musculoskeletal system in infants. Thus, screening for DDH is a recommended practice for all newborns, because early diagnosis and treatment are paramount to guarantee the best possible outcome for this pathology [1]. Known available tests for DDH screening are the clinical examination (Ortolani-Barrow maneuver) and the ultrasound examination. Execution of the latter, which is more sensitive than the former, is mandatory before DDH therapy is prescribed. For an ultrasound study of the hip to be reliable, it must be carried out by expert and well-trained operators [2]. In Italy, to date, hip ultrasound training courses are few and mainly theoretically oriented. In order to obviate, at least partially, to this lack of training opportunities, we present a novel, remote training, e-learning programme, aimed at enabling trainees to comfortably perform remote reading and reporting on a set of ultrasound images of both normal and pathological hips. The e-learning experience is organized as follows. There are two categories of users: administrators (domain experts) and trainees. Platform administrators load and maintain a dataset of normal and pathological hip ultrasound images. Some of these will have been obtained through incorrect scanning and trainees should acquire the ability to detect and discard them. With a correctly acquired hip image (i.e., one obtained in the “standard plane”), the trainee will be able to draw lines and circles on a canvas having the image itself as the background. For this purpose, the HTML5 Canvas library is employed on a bitmap, or “canvas”, modelled as a x-y plane [3]. The system analyzes points, lines and circles drawn by the trainee user and, through the appropriate mathematical formulae, computes the relevant intersection coordinates, angles and diameters, and automatically produces a report based on computed data, in accordance with the interpretation standards chosen by administrators. All images annotated by trainees will be checked and corrected by administrators, so as to provide the former with useful feedback. Finally, the platform will be made available to the scientific community as a very effective means to agree shared interpretation and reporting standards for neonatal hip ultrasound imagery.

**References**

1. Quader N, Schaeffer EK,. Hodgson AJ. A systematic review and meta-analysis on the reproducibility of ultrasound-based metrics for assessing developmental dysplasia of the hip. J Pediatr Orthop. 2018; 38: 305-311.

2. Choudry QA, Paton RW. Neonatal screening and selective sonographic imaging in the diagnosis of developmental dysplasia of the hip. Bone Joint J. 2018; 100-B: 806-10.

3. World Wide Web Consortium W3C. HTML Canvas 2D Context. Available from: https://www.w3.org/TR/2dcontext/ [Accessed 15 July 2019].

## A10 Adolescence, smartphone and tablets: a review of the literature

### Elena Bozzola^1^, Giulia Spina^1^, Margherita Ruggiero^1^, Davide Vecchio^2^, Mauro Bozzola^3^, Alberto Villani^1^

#### ^1^Pediatric and Infectious Diseases Unit, Bambino Gesù Children Hospital, Rome, Italy; ^2^ Medical Genetics Unit, “V. Cervello” Hospital, Palermo, Italy; ^3^Unit of Pediatric and Adolescence, Department of Internal Medicine and therapeutics, University of Pavia, Italy

##### **Correspondence:** Elena Bozzola (elena.bozzola@opbg.net)

**Background**

The use of media device, such as smartphone and tablet, is currently increasing, especially among the youngest [1]. The youth spend more and more time with their smartphones consulting social media, mainly Facebook, Instagram and Twitter because of the necessity to use it for the adolescents as means to construct a social identity and express themselves. On average, children are getting their first smartphones youger than in the previous years, around age ten. For some children, smartphone ownership starts even sooner as young as seven years, according to internet safety experts. Also in Italy, twelve-thirteen years is the the age of first access to nowadays [2, 3].

**Materials and methods.**

We reviewed the last 10 years literature to find out the possible consequences of tablets and smartphone use in adolescence.

**Results.**

In literature, smartphones and tablets use is associated to potential benefits, such as the availability of education tools for studying, chat apps for connecting with friends and the wealth of information on the web. But there are also negative outcomes of media use by adolescents on:

- learning. Social media use correlates to lower academic performance and a reduced concentration [4];
- sleep. An excessive use of smartphones is associated to a poor quality of sleep and a shorter sleep time [5,6];
- sigh. The increased use of smartphones may result in dry eye disease, eye irritation and fatigue, burning sensation, conjunctival injection, decreased visual acuity, strain, and fatigue acute acquired comitant esotropia [7,8];
- addiction. Many adolescents use compulsively their smartphones to listen music, check emails and social apps, even during a face-to-face communication [9]. Smartphones addiction has been linked to psychological disorders, such as low self-esteem, anxiety, depression mood, and loneliness [10];
- physical symptoms. Musculoskeletal disorders, such as neck or shoulder pain, and sedentary lifestyle, with consequently risk of obesity, are associated to a prolonged use of tablets in adolescents [11,12];
- cyberbullying and online addictions. Research has consistently shown that time spent on line is correlated with cyberbulling involvments [13];
- Hikikomori**.** This phenomenon of social withdrawal is becoming widespreading in sever countries [14].

**Conclusions.**

Smartphones and social media are increasing used by adolescents, influencing their mood and their style of life. Clinicians and parents must be aware of the possible neuropsychological side effects of media device use among adolescents.

**References**

1. Bozzola E, Spina G, Ruggiero M, Memo L, Agostiniani R, Bozzola M, et al. Media devices in pre-school children: the recommendations of the Italian pediatric society. Ital J Pediatr. 2018;44:69.

2. The statistics portal. http://www.statista.com Accessed 5 September May 2019.

3. Oberst U, Renau V, Chamarro A, Carbonell X. Gender stereotypes in Facebook profiles: are women more female online? Comput Hum Behav. 2016;60:559-564.

4. Dewitte S, Schouwenburg HC. Procrastination, temptations, and incentives: The struggle between the present and the future in procrastinators and the punctual. Eur J Personal. 2002;16:469–489.

5. Lanaj K, Johnson RE, Barnes CM. Beginning the workday yet already depleted? Consequences of late-night smartphone use and sleep. Organ Behav Hum Decis Process. 2014;124:11–23.

6. Lemola S, Perkinson-Gloor N, Brand S, Dewald-Kaufmann JF, Grob A. Adolescents’ electronic media use at night, sleep disturbance, and depressive symptoms in the smartphone age. J Youth Adolesc. 2015;44:405–418.

7. Smick K. Guarding your patient’s eyes for harmful light: Part one: The importance of education. Rev Optometry. 2014;151:26–28.

8. Bergqvist UO, Knave BG. Eye discomfort and work with visual display terminals. Scand J Work Environ Health. 1994;20:27–33.

9. Chotpitayasunondh V, Douglas KM. How “phubbing” becomes the norm: The antecedents and consequences of snubbing via smartphone. Comput Hum Behav. 2016;63:9-18.

10. Caplan SE. Relations among loneliness, social anxiety, and problematic Internet use. CyberPsych & Behav. 2006;10:234–242.

11. Ko K, Kim HS, Woo JH. The study of muscle fatigue and risks of musculoskeletal system disorders from text inputting on a smartphone. J Ergon Soc Korea. 2013;32: 273–278.

12. Cao H, Sun Y, Wan Y, Hao J, Tao F. Problematic Internet use in Chinese adolescents and its relation to psychosomatic symptoms and life satisfaction. BMC Public Health. 2011;11:802.

13. Kowalski RM, Giumetti GW, Schroeder AN, Lattanner MR. Bullying in the digital age: a critical review and meta-analysis of cyberbullying research among youth. Psychol Bull. 2014;140:1073-137.

14. Kato TA, Kanba S, Teo AR. Hikikomori: experience in Japan and international relevance. World Psychiatry. 2018;17: 05.

## A11 Media device addiction

### Elena Bozzola^1^, Margherita Ruggiero^1^, Giulia Spina^1^, Davide Vecchio^2^, Mauro Bozzola^3^, Alberto Villani^1^

#### ^1^Pediatric and Infectious Diseases Unit, Bambino Gesù Children Hospital, Rome, Italy; ^2^ Medical Genetics Unit, “V. Cervello” Hospital, Palermo, Italy; ^3^Unit of Pediatric and Adolescence, Department of Internal Medicine and therapeutics, University of Pavia, Italy

##### **Correspondence:** Elena Bozzola (elena.bozzola@opbg.net)

**Background**

Media device, such as smartphone, tablets and other mobile phones are actually overused, especially by adolescents and young adults. Overuse of mobile phones can affect social and psychological well-being and health and may let to addiction Addiction is defined as a obsession with a certain activity causing a disturbance of dailies activities [1]. In a recent survey on media device use, 70% of responders admit that they check their phones in the morning within an hour of getting up and 56% that they check their phones before going to bed. Moreover, 44% of responders stated that they would feel very anxious and irritable if they did not interact with their phones within a week [2].

**Materials and methods.**

We reviewed the last 10 years literature to find out the possible consequences of media device addiction in adolescence.

**Results.**

Media device addiction have been related to to excessive money on mobile phones, use of media device in socially or physically inappropriate situations such as while driving or during a meal. Many adolescents use compulsively their smartphones to check emails and social apps, even during a face-to-face communication [3]. Smartphones addiction has been linked to psychological disorders, such as low self-esteem, anxiety, depression mood, and loneliness [4]. In fact, a prolonged used of mobile devices may led to adverse effects on relationships, as well as to anxiety if separated from a mobile phone or if there is not a sufficient signal. Addiction may also led to phenomenon of social withdrawal, called Hikikomori [5]. The appearance of people, especially teenagers and young men, who stopped going to school or the workplace and spent most of the time withdrawn into their homes for months or years, appeared in Japan by the late 1990s. One decade later, in 2010, it has been definited as “a situation where a person without psychosis is withdrawn into his/her home for more than six months and does not participate in society such as attending school and/or work”. In the last years, following further advances in Internet society, hikikomori has become increasingly recognized in several Western Countries [6]^..^

**Conclusions.**

Smartphones and social media, mainly Facebook, Instagram and Twitter, are increasing used by adolescents, influencing their mood and their style of life. Clinicians and parents must be aware of the possible neuropsychological side effects of media device use among adolescents and in particular of the risk of addiction.

**References**

1. Kwon M, Kim DJ, Cho H, Yang S. The smartphone addiction scale: development and validation of a short version for adolescents. PLoS ONE. 2013;8;e83558.

2. Perlow LA. Sleeping with your smartphone: how to break the 24/7 habit and change the way you work. Harvard Business Review. 2012. p.15-35.

3. Chotpitayasunondh V, Douglas KM. How “phubbing” becomes the norm: The antecedents and consequences of snubbing via smartphone. Comput Hum Behav. 2016;63:9-18.

4. Caplan SE. Relations among loneliness, social anxiety, and problematic Internet use. Cyber Psychology Behav. 2006;10:234–242.

5. Kato TA, Kanba S, Teo AR. Hikikomori: experience in Japan and international relevance. World Psychiatry. 2018;17:105-106.

6. Teo AR, Fetters MD, Stufflebam K, Tateno M, Balhara Y, Young Choi T, et al. Identification of the Hikikomori syndrome of social withdrawal: psychosocial features and treatment preferences in four countries. Int J Soc Psychiatry. 2015;61:64-72.

## A12 Acute central nervous system infections: how to protect

### Elena Bozzola, Andrzej Krzystofiak, Laura Lancella, Anna Quondamcarlo, Laura Cursi, Giuseppina Gennarini, Sabrina Falasca, Alberto Villani

#### Pediatric and Infectious Diseases Unit, Bambino Gesù Children Hospital, Rome, Italy

##### **Correspondence:** Elena Bozzola (elena.bozzola@opbg.net)

**Background**

Pediatric bacterial meningitis is a real neurologic emergency. An early diagnosis and a prompt start of empiric antimicrobial therapy are essentual, but in some cases they can’t prevent the death or the neurologial sequelae. Moreover, clinical symptoms such as fever, headache, meningismus, and an altered level of consciousness are suggestive of meningitis. However they may be absent, expecially in the very young children, leading to a delay in both diagnosis and therapy [1].

**Materials and methods**

We reviewed the last 10 years literature to find out the possible way to prevent bacterial meningitis.

**Results**

In literature a number of risk factors of meningitis are reported, including socioeconomic factors, age, genetic variation of the host and underlying medical conditions. They are associated with increased susceptibility to invasive bacterial infections in both children and adults [2,3] . Widespread implementation of vaccination policies against meningococcal, pneumococcal and *Haemophilus influenzae* type b diseases has led to a significant decline in the frequency of bacterial meningitis over the past years [4]. Anyway, prevention still remains a priority because of the devastating outcomes and risk for outbreaks. Moreover, vaccination may provide direct and indirect protection and reduce the incidence of bacterial meningitis. For example, in England, within three years, the vaccination campaign against Neisseria meningitidis C resulted in a reduction in incidence of both vaccinated and unvaccinated children [5].

**Conclusions.**

Vaccination against common pathogens has decreased the burden of disease and is actually the only effective way to prevent some of bacterial meningitis.

**References**

1. Posadas E, Fisher J. Pediatric bacterial meningitis: an update on early identification and management. Pediatr Emerg Med Pract. 2018;15:1-20.

2. Pace D, Pollard AJ. Meningococcal disease: clinical presentation and sequelae. Vaccine. 2012;30 Suppl 2:B3-9.

3. Lundbo LF, Benfield T. Risk factors for community-acquired bacterial meningitis. Infect Dis (Lond). 2017;49:433-444.

4. McIntyre PB, O'Brien KL, Greenwood B, van de Beek D. Effect of vaccines on bacterial meningitis worldwide. *Lancet.* 2012;380:1703-1711.

5. Ramsay ME, Andrews NJ, Trotter CL, Kaczmarski EB, Miller E. Herd immunity from meningococcal serogroup C conjugate vaccination in England: database analysis. BMJ. 2003;326:365-6.

## A13 Endocrinological complications in celiac children

### Mauro Bozzola^1^, Chiara Montalbano^1^, Elena Bozzola^2^, Pietro Ferrara^3,4^, Alberto Villani^2^

#### ^1^Department of Internal Medicine and Therapeutics, Unit of Pediatrics and Adolescentology, University of Pavia, Pavia, Italy; ^2^Department of Pediatrics, Pediatric and Infectious Diseases Unit, Bambino Gesù Children Hospital IRCCS, Rome, Italy; ^3^Institute of Pediatrics, Catholic University and ^4^Campus Bio-Medico University, Rome, Italy

##### **Correspondence:** Mauro Bozzola (mauro.bozzola@unipv.it)

Celiac disease (CD) is an immune-mediated enteropathy triggered by dietary gluten-containing cereals in genetically susceptible individuals and results in a wide-range of intestinal and extraintestinal manifestations. Short stature is the most common extraintestinal feature of CD and may be the only symptom of the disease. The pathogenesis of growth failure is probably due to malabsorption or abnormality in the endocrine growth axis. A lack of adequate nutrient availability seems to inhibit regular hormone generation, like in patients with undernutrition. In celiac children, the introduction of a gluten-free diet (GFD) increases the intestinal absorption of both macro and micronutrients leading to an increase in both height and weight. However, some celiac children with short stature fail to display catch-up growth despite a strict GFD as the growth failure in CD patients is not completely due to undernutrition. This finding is confirmed by reports of overweight or obese children at the time of CD diagnosis. After starting a GFD, the celiac child usually returns to the normal growth curve for weight and height. If the catch-up growth does not occur, after 1-2 years of GFD, in the presence of seronegativity for specific celiac antibodies, an evaluation of GH secretion in response to two pharmacological stimuli is mandatory. However, only 0.23% of patients with short stature show an association between CD and GH deficiency (GHD) suggesting that this association is uncommon [1]. In CD patients with GHD, substitutive therapy should be promptly started at standard doses to achieve complete catch-up growth. The long-term effects of GH treatment in patients who follow a strict GFD are similar to those observed in children with idiopathic GHD [2]. The association between CD and autoimmune diseases is due to the common genetic predisposition such as HLA-DQ2 or DQ8 haplotypes and, the most frequent is between CD and Hashimoto thyroiditis. Therefore, in celiac patients anti-thyroid antibodies should be monitored annually and in the presence of seropositivity, is mandatory a thyroid echography. More rare is Graves disease characterized by the presence of antibodies anti-TSH and thyroid parenchyma disomogenous and vascularized at echography. Another widely that between CD and Diabetes mellitus type 1 (T1DM). In most documented association is cases, CD is diagnosed months or years after the onset of T1DM. Finally, anti-pituitary and anti-hypothalamus autoantibodies have been detected in celiac GHD children without catch-up growth after GFD, suggesting the onset of autoimmune hypophysitis [3].

**References**

1. Bozzola M, Giovenale D, Bozzola E, Meazza C, Martinetti M, Tinelli C, et al. Growth hormone deficiency and coeliac disease: An unusual association? Clin Endocrinol. 2005;62:372–375.

2. Meazza C, Pagani S, Messini B, Cardinale GM, Mastrangelo C, Citro G, et al. Celiac children treated for growth hormone deficiency reach normal final height. Clin Endocrinol. 2011;74:791–792.

3. Delvecchio M, de Bellis A, Francavilla R, Rutigliano V, Predieri B, Indrio F, et al. Anti-pituitary antibodies in children with newly diagnosed celiac disease: A novel finding contributing to linear-growth impairment. Am J Gastroenterol. 2010;105:691–696.

## A14 Biologic drugs in macrophage activation syndrome

### Claudia Bracaglia (claudia.bracaglia@opbg.net)

#### Division of Rheumatology, IRCCS Ospedale Pediatrico Bambino Gesù, Rome, Italy

Macrophage activation syndrome (MAS) is a potentially fatal complication of rheumatic diseases, particularly of systemic juvenile idiopathic arthritis (sJIA). MAS is caused by excessive activation and proliferation of T lymphocytes and of macrophages with overproduction of cytokines. MAS is a life-threatening condition associated with high mortality rates. Therefore, early recognition and immediate therapeutic intervention are critical [1].

Actually, the mainstay of treatment of MAS is high-dose glucocorticoids, cyclosporine, and in more difficult instances, etoposide. The utility of biologic drugs in MAS treatment remains unclear.

Although TNFα inhibiting agents have been reported to be effective in occasional MAS patients, other reports describe patients in whom MAS developed while they were on TNFα-inhibiting agents.

Since in sJIA MAS episodes are often triggered by the disease flare, biologics that neutralize IL-1, a cytokine that plays a pivotal role in sJIA pathogenesis, have been tried by some authors. There are several reports of sJIA-associated MAS dramatically benefiting from anakinra, a recombinant IL-1 receptor antagonist, however, are also reported patients who developed MAS while being treated with anakinra. Canakinumab, a monoclonal antibody directed against IL-1β, is an effective treatment in sJIA, it does not appear to have a significant effect on reducing the risk of MAS [2-3].

IL-6 blockade, via the anti-IL-6 receptor monoclonal antibody tocilizumab, has proven highly efficacious in treating sJIA. There are no reports of MAS treated specifically with tocilizumab. In a phase III clinical trial in sJIA, MAS was observed in some patients receiving tocilizumab [2-3].

High serum levels of IL-18 have been observed in sJIA and patients with higher levels of IL-18 also appear more likely to develop MAS. However, in many sJIA patients, plasma IL-18 levels remain elevated even in clinical remission. A phase II clinical trial in patients with Adult Onset Still Disease (AOSD) with tadekinig, the recombinant IL-18 binding protein, showed that approximately 50% of the recruited patients met the primary outcome [4].

IFN-γ and IFN-γ-induced chemokines, CXCL9 and CXCL10, are significantly elevated in MAS patients compared to sJIA patients without MAS. Emapalumab, an anti-IFN-γ monoclonal antibody, is already approved for the treatment of primary HLH patients. A clinical trial in patients with MAS in the context of sJIA is ongoing and preliminary results are highly promising [5].

Currently, the mainstay of treatment of MAS remains immunosuppressive therapy. However, in the immediate future, new target therapy will allow a better disease control.

**References**

1. Ravelli A, Grom AA, Behrens EM, Cron RQ. Macrophage activation syndrome as part of systemic juvenile idiopathic arthritis: diagnosis, genetics, pathophysiology and treatment. Genes Immun. 2012;13:289–98.

2. Schulert GS, Grom AA. Macrophage activation syndrome and cytokine directed therapies. Best Pract Res Clin Rheumatol. 2014; 28: 277–292.

3. Grom AA, Horne A, De Benedetti F. Macrophage activation syndrome in the era of biologic therapy. Nat Rev Rheumatol. 2016;12:259-68.

4. Prencipe G, Bracaglia C, De Benedetti F. Interleukin-18 in pediatric rheumatic diseases. Curr Opin Rheumatol. 2019. [Epub ahead of print]

5. De Benedetti F, Brogan P, Grom A, Quartier P, Schneider R, De Graaf K et al. Emapalumab, an interferon gamma (IFN-y)-blocking monoclonal antibody, in patients with macrophage activation syndrome (MAS) complicating systemic juvenile idiopathic arthritis (sJIA). Ann Rheum Dis. 2019; 78 (S2):178.

## A15 Congenital anomalies of the coronary arteries: the experience of a high-volume tertiary center

### Maurizio Brighenti^1^, Gabriele Bronzetti^1^ , Andrea Pession^3^ , Gaetano D. Gargiulo^2^ , Andrea Donti^1^

#### ^1^Pediatric Cardiology & GUCH Unit, Cardiothoracic-Vascular Department, S. Orsola-Malpighi Hospital, University of Bologna, Bologna, Italy; ^2^Pediatric & Grown-up Congenital Cardiac Surgery Unit, Cardiothoracic-Vascular Department, S. Orsola-Malpighi Hospital, University of Bologna, Bologna, Italy; ^3^Department of Pediatrics, S. Orsola-Malpighi Hospital, University of Bologna, Bologna, Italy

##### **Correspondence:** Maurizio Brighenti (maurizio.brighenti@yahoo.it)

**Background**

The prevalence of coronary arteries (CA) congenital anomalies is 1-2% in the general population with a wide spectrum of clinical manifestations from total innocuity to sudden cardiac death. Since medical, surgical and transcatheter therapies are always developing there is still lack of knowledge about long–term outcome for each anatomic subtype.

**Materials and methods**

Clinical charts of 53 patients (25 M, mean age 14.3 ± 8.6 years) with isolated CA anomalies or associated with minor congenital heart defects (CHD) in the period 1/01/2004 - 12/31/2018 were retrospectively reviewed in order to analyze risk factors for poor outcome.

**Results**

Eight patients (15%) had an associated CHD. Twenty-one patients (39.6%) had an abnormal coronary origin from the pulmonary artery or its branches (17 with anomalous origin of the left CA from the pulmonary artery - ALCAPA), 18 patients (34%) had single or multiple CA fistulae, 12 patients (22.6%) had an anomalous CA from the opposite sinus (ACAOS). The most used preprocedural diagnostic technique was CA computed tomography (42 % of the patients). Thirty three patients underwent surgical interventions aimed at restoring physiological coronary circulation, 16 (48.5%) of them for ALCAPA repair. On the other side 34 (64%) patients underwent at least one cardiac catheterization; 13 (38.2%) of them for interventional purpose (mostly embolization of CA fistulae). ALCAPA repair was safe and effective in the medium to long-term follow-up as above 75% of the patients had a complete normalization of left ventricular function at > 6 months of follow-up after surgery. We recorded 7 cases of post-operative significant pericardial effusion; 6 of them had a diagnosis of anomalous origin of a CA from the pulmonary artery and one with left-ACAOS. One other ALCAPA patient was diagnosed with intracardiac thrombosis. There were no adverse events after diagnostic or interventional cardiac catheterizations. Only 3 patients with ACAOS and one with ALCAPA achieved the combined end-point of death-cardiac transplantation showing a cumulative rate of 7.5%. All the patients with left-ACAOS mentioned above required a variable run of extracorporeal membrane oxygenation during the hospitalization beacause of intractable low cardiac output syndrome.

**Conclusions**

In skilled hands surgical and catheterization procedures are safe and able to restore phisiological CA circulation. Overall long-term survival for CA anomalies is excellent but some anatomic subtypes like left-ACAOS carry a high risk of sudden death. A further piece in the puzzle is required in the modern era of preparticipation screening.

## A16 Complement system in kidney diseases: not yet fully understood

### Valentina Bruno^1^, Serena Ascione^1^, Francesca Nuzzi^1^, Maria Rosaria D’Armiento^2^, Giuseppina Marino Marsilia^3^, Elena Bresin^4^, Carmine Pecoraro^1^

#### ^1^Division of Paediatric Nephrology and Dialysis, Santobono-Pausilipon Children’s Hospital, Naples, Italy; ^2^ Department of Biomorphological and Functional Sciences, Section of Anatomical Pathology, Federico II University, Naples, Italy; ^3^ Unit of Anatomical Pathology, A. Cardarelli Hospital, Naples, Italy; ^4^ Mario Negri Institute for Pharmacological Research, Bergamo, Italy

##### Correspondence: Valentina Bruno

**Background**

The complement system is recognized as a functional bridge between innate and adaptive immune responses, leading to integrated host defense against infections.

The pathogenetic role of hyperactivation/dysregulation of complement has been evaluated in several kidney diseases. Traditionally, genetic dysregulation of complement alternative pathway (CAP) is described as the main pathogenetic mechanism of atypical hemolytic uremic syndrome (aHUS) and C3 glomerulopathy (C3G) [1]. Growing evidence suggests the pathogenetic role of genetic dysregulation of CAP in other renal diseases, such as (Shiga toxin-producing Escherichia coli) STEC-HUS or atypical post-infectious glomerulonephritis (PIGN) [2].

**Materials and methods**

201 PIGN patients (121 M, mean age: 5.6 yrs) were retrospectively studied from 2001 to 2017; atypical cases underwent a kidney biopsy. In selected patients, molecular analysis of complement genes (Next-generation sequencing) was performed. The following variables were analysed at the onset in both typical and atypical cases: sex, age, glomerular filtration rate (GFR), proteinuria, serum C3 and C4 levels.

**Results**

35/201 patients (17.4%) underwent a kidney biopsy; 16/35 (45.71%) showed a morphological pattern of C3 glomerulopathy (C3G). In 16/16 children molecular analysis of complement-related genes was performed revealing polymorphisms and/or gene mutations in 12/16 cases (5 p.V62I in CFH gene; 3 homozygous polymorphism c.332C>T in CFH gene; 3 allelic variants V62 and H402 in CFH gene; 1 heterozygous gene mutation in CFH - p.G133R; 2 p.A473V in THBD gene; 1 new splice variant c.4851-1G>A - C3 gene; 1 p.R102G and p.P314L in C3 gene, 1 c.897 T>C in MCP gene). Serum C3 levels were significantly lower in typical PIGN compared to atypical cases (0.34 +/- 0.02 vs 0.58 +/-0.08; p < 0.05 (Figure 1). Glomerular filtration rate at the onset was lower in atypical PIGN, even if not statistically significant. No differences observed in age of onset, proteinuria and serum C4 levels (Table1).

**Conclusions**

The role of complement system in aHUS and C3G has been widely studied over the last years. Nevertheless, genetic dysregulation of CAP has been suggested as possible pathogenetic mechanism in other kidney diseases such as PIGN, a common form of glomerulonephritis in paediatric age. Prognosis is usually good; however, a few patients present with atypical features, such as persistent low C3 and/or persistent proteinuria and/or decline in renal function. In our experience, these patients can reveal a genetic defect in the regulation of CAP, suggesting PIGN and C3G as a spectrum of multifactorial glomerular disease with either primary or secondary complement activation triggered by infection [3].

**References**

1. Noris M, Remuzzi G. Genetics and genetic testing in hemolytic uremic syndrome/thrombotic thrombocytopenic purpura. Semin Nephrol. 2010;30:395-408.

2. De Vriese AS, Sethi S, Van Praet J, Nath KA, Fervenza FC. Kidney Disease Caused by Dysregulation of the Complement Alternative Pathway: An Etiologic Approach. J Am Soc Nephrol. 2015;26:2917-29.

3. Al-Ghaithi B, Chanchlani R, Riedl M, Thorner P, Licht C. C3 Glomerulopathy and post-infectious glomerulonephritis define a disease spectrum. Pediatr Nephrol. 2016;31:2079-86.

**Fig. 1 (abstract A16).**
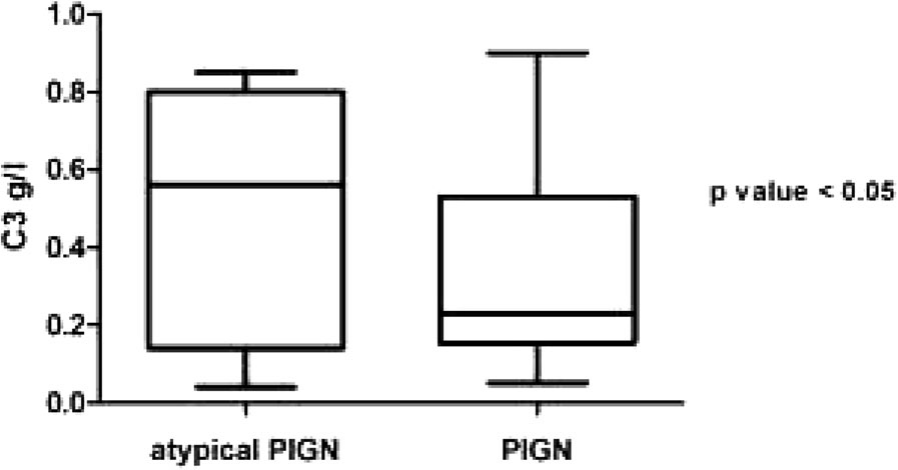
Serum C3 levels at the onset

**Table 1 (abstract A16).** Variables analysed at the onset

	Atypical PIGN	Typical PIGN	*P* value
Age of onset (years)	5.84 ± 0.514	5.054 ± 0.261	n.s.
eGFR ml/min/1.73 m^2^	79.71 ± 7.354	92.45 ± 2.888	n.s.
Proteinuria mg/24 hours	1033 ± 253.9	1095 ± 128.1	n.s.
Serum C3 levels g/l	0.580 ± 0.081	0.340 ± 0.023	< 0.05
Serum C4 levels g/l	0.216 ± 0.013	0.219 ± 0.011	n.s.

## A17 Asthma and sport in children

### Carlo Caffarelli, Angelica Santoro, Laura Nicoletti, Luca Bernardi, Giulia Dal Canto

#### Clinica Pediatrica, Azienda Ospedaliero-Universitaria, Dipartimento di Medicina e Chirurgia University of Parma, 43100 Parma, Italy

##### **Correspondence:** Carlo Caffarelli (carlo.caffarelli@unipr.it)

The term exercise-induced-bronchoconstriction (EIB) defines the acute and transient airways narrowing occurring during or immediately after exercise. EIB may occur both in asthmatic and non-asthmatic patients. EIB affects from 40 to 90% of individuals with asthma [1] and approximately from 8 to 10% of children without asthma. Children with EIB typically experience chest tightness, shortness of breath, cough, wheeze, due to bronchoconstriction which generally begins 3 minutes after exercise, peaks in 10-15 minutes and resolves by 60 minutes. Patients with atopic asthma may present EIB more likely when exercise includes exposure to the relevant allergen or when asthma is not optimally controlled. The diagnosis of EIB is based on the combination of clinical symptoms and documented dynamic changes in airway function during or after exercise. Exercise testing necessary for the diagnosis of EIB and to differentiate EIB from other causes of exercise-induced dyspnoea. The standard test is represented by cycle ergometer or treadmill test. However, to prevent poor collaboration, children can perform a free-runing test which is easy, reproducible, inexpensive and improve the compliance [2,3]. The test is performed under surveillance in a level corridor or outdoor, the child must be encouraged to run as fast as possible for 6 minutes, enough to rise the heart rate to 80 to 90% of the predicted maximum (calculated by 220-age). FEV1 (forced expiratory volume in one second) should be recorded at baseline and following exercise for at least 15 minutes. A test is positive if FEV 1 decreases by 15% or more [1]. Children who present bronchoconstriction should stop practicing, use short-acting-beta2-agonists and rest until his/her respiration has normalized. The optimal treatment for EIB is to prevent the onset of bronchoconstriction. The pharmacological measures include pre-exercise administration (about 15 minutes before) of inhaled short-acting beta-agonists (SABA). When SABA therapy is ineffective, or patients practice intermittently in an unpredictable way during the day, like children usually do, the administration (at least two hours before) of a leukotriene receptor antagonist may be considered [4]. Among nonpharmacological measures that may help to reduce EIB we mention a warm-up before exercise, practice where the air is warmer, and the humidity is higher. Education should focus on recognizing of symptoms, having a correct inhaler technique, controlling the underlying and contributing factors. The aim of the management is to ensure that exercise is not avoided in children with EIB.

**References**

1. Caffarelli C, Bacchini PL, Gruppi L, Bernasconi S. Exercise-induced bronchoconstriction in children with atopic eczema. Pediatr Allergy Immunol. 2005:16:655-661.

2. Pierson WE. Exercise-induced bronchospasm in children and adolescents. Pediatr Clin North Am. 1988:35:112-30.

3. Shapiro GG, Pearson WE, Furukama CT. A comparison of effectiveness of free-running and treadmill exercise for assessing exercise-induced bronchospasm in clinical practice. J Allergy Clin Immunol. 1979:64:609-12.

4. Parsons JP, Hallstrand TS, Mastronarde JG, Kaminsky DA, Rundell KW, Hull JH, et al. An official American Thoracic Society clinical practice guideline: exercise-induced bronchoconstriction. Am J Respir Crit Care Med. 2013;187:1016-27.

## A18 The transition in diabetic patient

### Francesca Cardella (cardellafrancesca@gmail.com)

#### Pediatric Diabetology Regional Reference Center, Department of Pediatric, Children Hospital “G. Di Cristina”, Palermo, Italy

The transition from pediatrics to adult clinics represents a difficult moment in the management of any chronic pediatric onset disease, particularly in patients with Type 1 diabetes mellitus.

It is necessary to start a structured care pathway that can facilitate the passage of the management of the diabetic young person from the Pediatric to the Adult structure, avoiding the drop-out of patients (around 30%) in this critical phase of life. The transition must be scheduled around the age of 18, except for specific needs motivated by the individual patient. The transfer must be gradual and not traumatic with the involvement of both care teams . The Pediatric Diabetology Reference Center will organize annual meetings (or school camps) together with diabetologists and patients who are considered ready to pass, in order to present the new care team. In each Adult Center a dedicated structure must be expected in accordance with the recommendations of the National and International Scientific Societies (SIEDP-SID-AMD and ISPAD). In addition to the Diabetologist, the team should include other professional figures such as the dedicated nurse, dietitian and, where possible, the psychologist.

Tasks of the Pediatric Diabetologist:

- Identify the young people "ready" for the transition, explaining the reasons, and adapting the care modalities to the characteristics of the adult's structure (cadence of the visits, education for goals of care) .
- Identify together with the young person the Adult Diabetology Center to which to refer (according to logistic needs or motivated by lifestyle).
- Provide an information booklet (service card) drawn up by the adult center .
- Prepare a detailed clinical sheet with all the characteristics of the patient (History, onset modality, comorbidity, possible complications, current therapy, compliance with self-management)
- Present the new care team.
- Inform the family doctor.

Tasks of the Adult Diabetologist:

- Provide an information booklet (service card)
- Establish the headquarters of the transition structure which must have a dedicated space and visiting hours.
- Identify the professional figures that are part of the Transition Team.

During the follow-up it’s necessary the connection between the teams, for the verification of the continuity of care. The patient after six months / 1 year should complete a satisfaction questionnaire.

Only by the collaboration of all the structures provided for the protection of healt we can get the best metabolic control, ensuring the therapeutic goals of a potentially disabling chronic disease.

## A19 Complementary feeding: which model?

### Margherita Caroli (margheritacaroli53@gmail.com)

#### Paediatrician and nutritionist on private practice, Francavilla Fontana (BR), Italy

It has been recently recognized that the nutrition of the first 1000 days of life can influence health up to adulthood. Around 6 months of age all infants start to consume solid foods. This period of time, previously called “weaning”, is now defined as “complementary feeding”, thus acknowledging that the main source of energy in the first year of life is human milk or formula. Even though the industry is committing itself to improve the quality of formulas, their nutritional content is quite different from that of human milk. Up to the end of the first year of life, i.e. in the lapse when milk is still the main source of energy, breast- and formula-fed infants should be treated differently when they start to introduce solid foods, according to the different nutritional contents of human milk and formula. Although complementary feeding is heavily influenced by local habits and culture, new scientific information should be the basis of anybody’s choices, so as to respect the child’s right to health. In order to offer a healthy and adequate diet to infants we must know what, when and how much food we must offer, according to whether they take human milk or formula. When – For many years children have been weaned too early, in contrast with WHO recommendations to start solid foods at 6 months of age, i.e. 180 days of life. What – Breastfed infants should be offered protein- and iron-rich foods, such as meat and fish, whereas formula-fed infants should be offered vegetables and cereals, in order to stimulate their taste for new flavours and to not increase the proteins’ intake. How much – Mothers frequently overestimate portions to be fed to their infants, so paediatricians need to inform them about the right amounts of food to offer, so that they don’t overfeed them. In conclusion, healthy complementary feeding models should:

1. 1 start solid foods at 6 full months: 180 days, according to WHO recommendations.
2. 2 start with different solid foods considering whether the baby is breast or formula-fed.
3. 3 remember that what the baby eats has the same importance as the way the baby is fed.
4. 4 consider that this is the time when most of the future health is programmed.

## A20 Common pediatric drugs: “wise” use and off-label prescription in out-patient settings

### Romeo Carrozzo (romeo.carrozzo@virgilio.it)

#### Family pediatrician, ATS Bergamo, Bergamo, Italy

“Off-label use” refers to the use of pharmaceutical drugs for an unapproved indication or in an unapproved age group, dosage, or route of administration. In pediatric practice, off-label prescription is widely employed both in in-patients and out-patients settings. However, this misuse may lead to inefficient treatments and adverse drug reactions. The need for a regulation of the market for pediatric drugs has moved in the 2000s both the Food and Drug Administration and the European Medical Agency to set systems of requirements, rewards and incentives to ensure high quality standards for drug research in pediatrics. Under those rules, the drug market has produced a larger amount of safe drugs for children. In Italy, several laws have faced the off-label use, stating that the off-label prescription might be appropriate in specific scenarios, such as the efficacy of the off-label drug demonstrated in scientific reports, and the lack of suitable alternatives among approved medications. In such cases, the inform consent to the patient has to be provided. Clinical guidelines and literature reviews seldom recommend the employment of off-label drugs in pediatric practice. Nonetheless, the availability of new drugs and constant reviews of the information for medical products challenge pediatricians for their practice, and underline the importance of a periodical update for physicians dealing with children in out-patients settings.

## A21 Intracranial bleeding and widespread ecchymoses in a newborn

### Roberto Carta^1^, Antonella Nonnis^1^, Maria Bonaria Piludu^1^, Lorenza Cara^1^, Catia Atzeni^1^, Antonio Cualbu^2^, Rossella Mura^1^, Anna Brigida Aru^1^

#### ^1^ Pediatric Haematology/Oncology Unit, Ospedale Pediatrico Microcitemico “A. Cao”, Cagliari, Italy; ^2^ Pediatric Unit , Ospedale San Francesco, Nuoro, Italy

##### **Correspondence:** Roberto Carta (roberto.carta.md@icloud.com)

**BACKGROUND**

Glanzmann Thrombasthenia (GT) is a rare inherited condition characterized by a platelet aggregation defect caused by mutation of genes encoding for integrin αIIb e β3 that constitute the fibrinogen platelet receptor. GT is clinically characterized by early onset bleeding disorders; diagnosis is based on absent/reduced platelet aggregation following the stimulation of physiological agonists, prolonged bleeding time or PFA-100 closing time and abnormal clot retraction time[1-7]. The standard treatment for GT consists of local measures for mucocutaneous bleeding, anti-fibrinolytic therapy and platelet transfusion. Recombinant activated factor VII has been shown to be effective in patients with platelet antibodies and refractoriness to platelet transfusions. Allogenic bone marrow transplantation, the only curative treatment, is reserved for patients not responsive to common therapies. Gene therapy is currently under investigation[7-11].

**CASE REPORT**

DG was admitted to neonatal intensive care unit in the first day of life due to widespread ecchymoses and desaturation. Oxygen therapy, broad-spectrum intravenous antibiotic therapy and repeated intramuscular therapy with vitamin K_1_ were undertaken. Blood tests showed anemia, platelet count and coagulation indexes were normal. Infectious examinations, performed on blood and cerebrospinal fluid, were negative. Cranial ultrasound showed areas of hyperechogenicity in thalami and in parietal lobes, brain computed tomography and magnetic resonance confirmed the presence of parenchymal, subarachnoid and subdural hemorrhagic foci in the right temporal lobe and parietal-occipital regions associated with edema. Due to this finding antiedema therapy with mannitol was started. Once the acute phase has been resolved, the child arrived at our Center at the age of 3.5 months for the persistence of ecchymoses. The coagulation tests (PT, aPTT, fibrinogen, Von-Willebrand factor, ristocetine cofactor, factor VIII) were confirmed as normal, the platelet aggregation test showed severe defect in response to ADP, collagen, epinephrine and reduced to ristocetin. Further evaluation showed total GpIIb deficiency and reduction of GpIIIa (25%) and GpIb (75%); GpIIb/IIIa receptor expression was 0.3% after activation with ADP and PAR1-AP whereas p-selectin exposure was 59.4% with PAR1-AP and 20% with ADP. A serious defect of platelet function compatible with the diagnosis of GT is thus identified. Platelet function tests were extended to the family and the asymptomatic father was identified as a carrier of the defect.

**CONCLUSION**

This case report underlines the importance of an accurate assessment of hemorrhagic signs and symptoms that associated to a detailed anamnesis and appropriate laboratory diagnostics allow to identify rare coagulation disorders, even in cases with unusual clinical expression as intracranial haemorrhage in neonatal age.

Informed consent to publish has been obtained from parents of this patient.

**References**

1. Orkin SH, Nathan DG, Ginsburg D. Approach to the child with a suspected bleeding disorder. In: Orkin SH, Nathan DG, Ginsburg D, Lock AT, Fisher DE, Lux SE, editors. Nathan and Oski’s Hematology and oncology of infancy and childhood. 8th edition. Philadelphia: Elsevier; 2015. p. 999-1009.

2. Sharma R, Perez Botero J, Jobe SM. Congenital disorders of platelet function and number. Pediatr Clin North Am. 2018; 65:561-578.

3. Nurden AT, Nurden P. Congenital platelet disorders and understanding of platelet function. Br J Haematol. 2014;165:165-78.

4. Nurden AT, Freson K, Seligsohn U. Inherited platelet disorders. Haemophilia. 2012;18.154-60.

5. Tabibian S, Motlagh H, Naderi M, Dorgalaleh A. Intracranial hemorrhage in congenital bleeding disorders. Blood Coagul Fibrinolysis. 2018;29:1-11.

6. Gresele P, Harrison P, Bury L, Falcinelli E, Gachet C, Hayward CP, et al. Diagnosis of suspected inherited platelet function disorders: results of a worldwide survey. J Thromb Haemost. 2014;12:1562-9.

7. Solh T, Botsford A, Solh M. Glanzmann's thrombasthenia: pathogenesis, diagnosis, and current and emerging treatment options. J Blood Med. 2015; 8:219-27.

8. Poon MC, Di Minno G, d'Oiron R, et al. New insights into the treatment of Glanzmann Thrombasthenia. Transfus Med Rev. 2016;30:92-9.

9. Grainger JD, Thachil J, Will AM. How we treat the platelet glycoprotein defects; Glanzmann thrombasthenia and Bernard Soulier syndrome in children and adults. Br J Haematol. 2018;182:621-632.

10. Nurden AT. Acquired Antibodies to αIIbβ3 in Glanzmann Thrombasthenia: From Transfusion and Pregnancy to Bone Marrow Transplants and Beyond. Transfus Med Rev. 2018;32:155-164.

11. Wilcox DA. Megakaryocyte- and megakaryocyte precursor-related gene therapies. Blood. 2016;127:1260-8.

## A22 The Italian approach to sickle cell disease in childhood: guidelines and national initiatives

### Maddalena Casale, Silverio Perrotta

#### Dipartimento della Donna, del bambino e di Chirurgia Generale e Specialistica, Università degli Studi della Campania Luigi Vanvitelli, Naples, Italy

##### **Correspondence:** Maddalena Casale (maddalena.casale@unicampania.it)

Sickle cell disease (SCD) is the most common genetic disorder worldwide, mostly prevalent in some areas affected by crushing humanitarian crisis. The high refugee influx across Mediterranean sea has increased the prevalence of this disorder, previously very low, in some Southern European countries, such as Italy [1].The World Health Organization has recognized SCD as a global public health problem and the nations greatly involved in the European refugee crisis are required to develop accessible health care to these vulnerable populations, especially children [2]. The Italian Society of Thalassemia and Hemoglobinopathies (SITE) and Italian Association of Pediatric Hematology Oncology (AIEOP) responded to these needs with the following initiatives: the creation of a national working group focused on SCD, the development of tailored guidelines to the Italian health care system, the training to emergency department and frontline health care physicians in diagnosis and treatment of acute events with a specifically designed algorithm, the implementation of screening for SCD in refugees by a point-of-care screening devices [1-3].

Pediatric hematologists representing 54 different centers across Italy produced a document consisting of 27 chapters and 242 recommendations, tailored to the Italian health care system which greatly differs US, UK and France where previous recommendations for standard of care in childhood SCD have been produced [2]. Management of pain, severe infections, cerebral stroke, acute chest syndrome and pulmonary disorders, spleen sequestration and splenectomy, transfusion and hydroxyurea therapy were some of the most relevant issues addressed in the document, available at the AIEOP website [http://www.aieop.org/web/]. In order to offer a faster and more feasible document to emergency providers and paediatricians, an algorithm with interactive windows was developed to guide physicians in the management of acute and often life-threatening complications due to SCD [4]. In the absence of a nationwide screening, the majority of SCD patients are diagnosed because of acute symptoms which cause frequent accesses to the emergency departments [3]. The principal aim of the algorithm was the training of operators and front line paediatricians in a fast and appropriate approach to the acute events, in order to ensure the right priority access to the evaluation and subsequent diagnosis and therapy [3]. The document can be downloaded from the SITE webpage [http://www.site-italia.org/]. Further documents were focused on the management of severe infections in SCD and splenectomised patients who are at high risk of this life-threatening complication [5] and on hydroxyurea therapy in SCD children [6].

**Acknowledgements**

We thank SITE and AIEOP and all the colleagues who supported the initiatives presented in this abstract.

**References**

1. De Franceschi L, Lux C, Piel FB, Gianesin B, Bonetti F, Casale M, et al. Access to emergency department for acute events and identification of sickle cell disease in refugees. Blood. 2019;133:2100-2103.

2. Colombatti R, Perrotta S, Samperi P, Casale M, Masera N, Palazzi G, et al. Organizing national responses for rare blood disorders: the Italian experience with sickle cell disease in childhood. Orphanet J Rare Dis. 2013;8:169.

3. Forni GL, Finco G, Graziadei G, Balocco M, Rigano P, Perrotta S, et al. Development of interactive algorithm for clinical management of acute events related to sickle cell disease in emergency department. Orphanet J Rare Dis. 2014;9:91.

4. Inusa B, Casale M, Booth C, Lucas S. Subarachnoid haemorrhage and cerebral vasculopathy in a child with sickle cell anaemia. BMJ Case Rep. 2014;2014.

5. Casale M, Cinque P, Ricchi P, Costantini S, Spasiano A, Prossomariti L, et al. Effect of splenectomy on iron balance in patients with β-thalassemia major: a long-term follow-up. Eur J Haematol. 2013;91:69-73.

6. Colombatti R, Palazzi G, Masera N, Notarangelo LD, Bonetti E, Samperi P, et al. Hydroxyurea prescription, availability and use for children with sickle cell disease in Italy: Results of a national multicenter survey. Pediatr Blood Cancer. 2018;65: e26774.

## A23 Managing pain: the Hospital setting

### Emanuele Castagno (ecastagno@cittadellasalute.to.it)

#### S.C. Pediatria d’Urgenza, Ospedale Infantile Regina Margherita - A.O.U. Città della Salute e della Scienza di Torino, Turin, Italy

Pain is a major cause of admission to the Emergency Department and represents an everyday matter of concern for hospitalized children, but its management is often inappropriate. Though a plenty of guidelines, recommendations and training strategies are available, pain evaluation, measurement and treatment is still lacking in all age groups and in different situations. Different factors could explain it, namely the false believes that pain is not a priority, in particular in the emergency setting, that children do not feel pain as much as adults, and that analgesics might delay correct diagnosis. On the contrary, prompt and adequate control of acute and procedural pain is well known to enhance the prognosis significantly, and represent a strict ethical, deontological and juridical duty for all the healthcare professionals. Cornerstones of adequate pain management are: accurate evaluation and measurement with validated scales; early treatment; and wise use of non-pharmacological and pharmacological strategies. First, pain should be evaluated and recorded throughout all the permanence of children in the Hospital, since admission to Emergency Departments to discharge home. Standardized validated scales should be known and used by nurses and doctors regularly, both to define the best strategy to control pain for each child and to monitor its efficacy.

Second, non-pharmacological techniques should be always used, ranging from the Hospital environment features to behavioral and relating aspects. This is very important to obtain children and their families’ alliance to defeat pain and to reduce drugs doses, in particular during procedures. Third, available pharmacological strategies should be well known and drugs should be administered properly in terms of indication, time and dosage. Adequate pain control should be guaranteed also on discharge, in alliance with outpatient settings and general practitioners. Adequate pain management should be guaranteed for all the children accessing Hospitals, in particular those who experience chronic pain and those more vulnerable because of neurological impairment and other special needs. Better management can be achieved through deep collaboration between pediatricians, intensive care specialists and nurses, and supporting specific skills development in pediatric training programs.

## A24 How to recognise signs and signals of discomfort in adolescent age

### Serenella Castronuovo (serenella.castronuovo@gmail.com)

#### Family pediatrician, Gruppo di Studio Nazionale Adolescenza della SIP, Nettuno (RM), 00048, Italy

Adolescence is the transitional phase of growth and development between childhood and adulthood.

Adolescence is the period in the life of a person during which many physical and psychological changes take place: adolescents need to adapt to them, they may have to solve issues with their personal, social and sexual identity. The Word Health Organization (WHO) defines an adolescent as any person between ages 10 and 19. This age range falls within WHO’s definition of *young people*, which refers to individuals between ages 10 and 24. The onset of the majority of the psychological diseases take place between 12 and 19 years of age [1]. Pediatricians "should be" the first operators to intercept, in addition to the somatic pathologies, also the initial stages of emotional and social discomforts. Only 13% of the pediatricians feel they are prepared and adequate for the management of adolescents [2].

In accordance with the preliminary results of a SIP survey [3]:

- 80% of the adolescents experiment emotional discomfort
- 50% feel the need of a psychological support (but 84,2% of them have not asked for any help)
- 15% of self-harm cases

For this reasons, in Italy the periodical "Health Evaluations" made by family paediatricians are very important. Prevention is a key objective of the evaluations: it is realized through a plan of filter medical examinations which take place each at the appropriate age in which the various issues can be detected. Adolescents do not love to go to the paediatrician, and for this reason the Health Evaluation is a good occasion for the doctor to put in evidence potential physical and psychological discomforts and, above all, having a talk with the patient. It is necessary to organise the medical practice in order to guarantee the right environment and time to adolescents. It is also necessary to plan the evaluation in order to safeguard the patient privacy. What to look for:

- ▪ Difficulty or inability having relations with peers
- ▪ Clearly inappropriate or potentially dangerous behaviours
- ▪ Abuse of alcohol, tobacco, drugs
- ▪ Inexplicable reduction of school performances
- ▪ Latent feeling of unhappiness and melancholy
- ▪ Low self-esteem
- ▪ Depression or free floating anxiety
- ▪ Isolation and Internet dependency
- ▪ Food behaviour disorders and/or alteration of the body image
- ▪ Signs of self-harm

The following tests (available for free) help to identify signs of discomfort:

- Questionnaire SDQ-ITA from 4 to 16 years-of-age, used internationally [4] [5]
- Questionnaire Child & Adolescent Behaviour Inventory [6][7]

**References**

1. Paus T, Keshavan M, Giedd JN.Why do many psychiatric disorders emerge during adoloscence? Nat Rev Neurosci. 2008;9:947-57.

2. Weitzman C, Wegner L. Promoting optimal development: Screening for behavioral and emotional problems. Pediatrics. 2015;135:384-395.

3. Società Italiana di Pediatria. Adolescenti, 80% ha sperimentato disagio emotivo. https://www.sip.it/2017/05/30/adolescenti-80-ha-sperimentato-disagio-emotivo/. Accessed 6 September 2019.

4. Goodman R. The Strengths and Difficulties Questionnaire: A research note. J Child Psychol Psychiatr.1997;38:581-586.

5. Goodman R. Psychometric properties of the Strengths and Difficulties Questionnaire (SDQ). J Am Acad Child Adolesc Psychiatr. 2001;40: 1337-1345.

6. Cianchetti C, Pittau A, Carta V, Campus G, Littarru R, Ledda MG, et al. Child and Adolescent Behavior Inventory (CABI): A New Instrument for Epidemiological Studies and Pre-Clinical Evaluation. Clin Pract Epidemiol Ment Health. 2013;9:51-61..

7. Cianchetti C, Marino M, Riccio MP, Craig F, Matera E, Ledda MG,et al., Child and adolescent behavior inventory (CABI): a new alternative to CBCL. Eur Child Adolesc Psychiatr. 2015;24(suppl.1):S22

## A25 Management of chronic lung disease in children

### Salvatore Cazzato, Federica Zallocco, Alessia Omenetti

#### Pediatrics Unit, Department of Mother and Child Health, G. Salesi Children’s Hospital, Ancona, Italy

##### **Correspondence:** Salvatore Cazzato (salvatore.cazzato@ospedaliriuniti.marche.it)

Among chronic lung diseases, non-cystic fibrosis bronchiectasis (NCFB) has recently received growing attention, mainly due to its increase prevalence worldwide, its impact on quality of life, and the current poor outcomes. An inappropriate medical approach to bronchiectasis may actually be associated with rapid decline in lung function and worse prognosis. Therefore, an early diagnosis and prompt optimal management is crucial, especially in children where disease progression may be delayed or even resolved in some cases [1,2]. Disease monitoring, acute exacerbation prompt identification and treatment and prevention of infections in the clinical management of NCFB will be reviewed. Unfortunately, the current treatment strategies have been extrapolated from those used for children with cystic fibrosis or adults with NCFB. The proposed methods used to monitor disease progression include 1) clinical signs and symptoms (i.e. change on cough quality and frequency, anthropological measurements), 2) lung function (i.e. pulmonary functions tests, PFTs), 3) imaging and sputum characteristics (i.e. color, volume, microbiology). However, PTFs may be hampered in youngest children, and bronchoalveolar lavage (BAL) may be required in those uncapable of expectorating [3,4]. Early identification and treatment of acute exacerbations is an integral part of clinical surveillance. Systemic antibiotics treatment should be actively administered to control airway infection during acute exacerbation [5]. At the same time, given the fact that NCFB children are at higher risk of more severe infectious complications and proned to new infections, prevention is an increasing concern in order to avoid further lung damage due to infections. NCFB children should fulfill the national immunization plan for high-risk groups [6]. Airway clearance therapy can be helpful in improving respiratory function and quality of life. Treatment options depend on the child’s age and ability to cooperate in treatment. Given the wide variety of airway clearance techniques, the best effective and patient-tailored physiotherapy techniques and devices should be chosen [7-9]. Airway hyper-responsiveness can be found in NCFB children. However, there is insufficient evidence to suggest the routine use of inhaled corticosteroids or ICS-LABA combination [10]. Inhalation of hypertonic saline can be helpful in enhancing mucociliary clearance or reducing cough in children, whereas evidence for effectiveness or inhalation of other mucoactive agents in children with bronchiectasis is lacking [11]. Finally, nutritional aspects is another concern in the management of children with NCFB. To date, limited studies on nutritional data are available, but failure to thrive and lower BMI are associated with poor prognosis [12].

**References**

1) Chang AB, Bush A, Grimwood K. Bronchiectasis in children: diagnosis and treatment. Lancet. 2018;392:866-79.

2 )Quint JK, Millett ER, Joshi M, Navaratnam V, Thomas SL, Hurst JR, et al. Changes in the incidence, prevalence and mortality of bronchiectasis in the UK from 2004 to 2013: a population-based cohort study. Eur Respir J. 2016;47:186-93.

3) Kapur N, Masters IB, Morris PS, Galligan J, Ware R, Chang AB. Defining pulmonary exacerbation in children with non‐cystic fibrosis bronchiectasis. Pediatr Pulmonol. 2012;47:68-75.

4) McCallum GB, Binks MJ. The epidemiology of chronic suppurative lung disease and bronchiectasis in children and adolescents. Front pediatr. 2017;20:5-27.

5) Kapur N, Masters IB, Newcombe P, Chang AB. The burden of disease in pediatric non-cystic fibrosis bronchiectasis. Chest. 2012;141:1018-24.

6) O'Grady KA, Cripps AW, Grimwood K. Paediatric and adult bronchiectasis: Vaccination in prevention and management. Respirology. 2019;24:107-14.

7) Main E, Grillo L, Rand S. Airway clearance strategies in cystic fibrosis and non-cystic fibrosis bronchiectasis. Sem Respir Crit Care Med. 2015; 36:251-266.

8) Indinnimeo L, Tancredi G, Barreto M, De Castro G, Zicari AM, Monaco F, et al. Effects of a program of hospital-supervised chest physical therapy on lung function tests in children with chronic respiratory disease: 1-year follow-up. Int J Immunopathol Pharmacol. 2007;20:841-5.

9) Lee AL, Burge AT, Holland AE. Airway clearance techniques for bronchiectasis. Cochrane Database Syst Rev. 2015;11:CD008351.

10) Lee E, Hong SJ. Pharmacotherapeutic strategies for treating bronchiectasis in pediatric patients. Expert Opin Pharmacother. 2019;22:1-2.

11) Elkins MR, Robinson M, Rose BR, Harbour C, Moriarty CP, Marks GB, et al. A controlled trial of long-term inhaled hypertonic saline in patients with cystic fibrosis. N Engl J Med. 2006;354:229-40.

12) Bastardo CM, Sonnappa S, Stanojevic S, Navarro A, Lopez PM, Jaffe A, et al. Non-cystic fibrosis bronchiectasis in childhood: longitudinal growth and lung function. Thorax. 2009;64:246-51.

## A26 Alcohol addiction during pregnancy

### Mauro Ceccanti^1^, Gemma Battagliese ^1^, Simona Gencarelli^1^, Fabiola Pisciotta^1^, Roberta Ledda^1^, Marisa P. Messina^1^, Rosaria Ciccarelli^1^, Giovanna Coriale^1^, Marco Fiore^2^

#### ^1^Sapienza University, Rome, Italy; ^2^IBCN-CNR, Rome, Italy

##### **Correspondence:** Mauro Ceccanti (mauro.ceccanti@uniroma1.it)

**Background**

The prevalence of FAS in the European Region has been estimated at 37.4 per 10.000 (Italy 82.0 per 10.000); while the prevalence of alcohol consumption during pregnancy in the same region is 25.2% (Italy 33.1%). Style of alcohol consumption in the young female population increased the risk for having a child with FASD. Safe amounts of alcohol during gestation have not been established. Therefore, medical authorities recommend no alcohol during pregnancy.

Small amounts of alcohol do not cause gross morphological abnormalities but may induce behavioural and memory impairments and reduced brain volume. Alcohol cross the blood-brain barrier disrupting the brain developing and potentiating oxidative stress. FASD diagnosis based on several signs and symptoms is a permanent condition.

**Materials and methods**

Studies:

1. The first was conducted on 96 pregnant women in follow-up at the ambulatory of Gynaecology and Obstetrics (Sapienza University of Rome);
2. the second study investigated 44 pregnant women during the second trimester of gestation in follow-up to the ambulatory of the same hospital.

Gynaecologists assessed drinking habits during pregnancy through a food diary. Then, pregnant women fill out the three structured questionnaires (AUDIT‐C, T‐ACE, and TWEAK). After the interviews, pregnant women were asked to provide a sample of their urine to assess the concentration of EtG. Following in time the morphologic ultrasound was carried out to all participants of study 2. EtG determinations were performed with a cut-off established at 100 ng/mL, a value indicating occasional alcohol drinking.

**Results**

Study 1: 34.28% of pregnant women overcame EtG cut-off. No direct correlation was found between EtG value and the alcohol screening interviews showing lower levels of alcohol consumption, although T-ACE revealed the same at-risk percentage.

Study 2: Fifteen of the enrolled pregnant women had EtG value higher than 100 ng/mL (34.09%). ANOVA revealed that the fetuses exposed to alcohol had significantly longer interorbital distance and also increased front-thalamic distance (P's < 0.02) (Ferraguti et al., 2019).

**Conclusions**

Findings show that assessed maternal alcohol consumption during pregnancy only with screening instruments may significantly underestimate alcohol use. The evaluation of maternal alcohol consumption during pregnancy by urine EtG may identify at-risk mothers and disclose FASD-related damage in the fetus.

## A27 Guidelines in pediatric dermatology

### Leonardo Celleno (leonardo.celleno@unicatt.it)

#### Fondazione Policlinico Gemelli, Rome, Italy

Especially in pediatric dermocosmetology the safety of use is the fundamental parameter that must be respected in the formulation of cosmetic products that are formulated for children.

Safety is based, even before that on the data of the finished product, on the toxicological characteristics of each ingredient that is used. It is up to the formulator and the safety assessor to choose adequate raw materials respect to purity, hypoallergenic charatheristic, low irritant power and a toxicological profile that allows to establish a very high MOS (Margity of Safety). Other fundamental points in the evaluation of safety are the presence of perfumes and preservatives and studies on the finished product conducted with scientifically acceptable methods. When creating products for children, it is necessary to consider how the child's skin presents significant morphological differences with that of the adult: high TEWL (trans epidermal water loss), slightly alkaline pH, poor buffer capacity, immature melanocytes with lower melanin content, thickness lower dermis and epidermis, saprophytic flora (microbiome) in development, quiescent sebaceous production, poorly developed hydrolipidic film, low concentration of NMF (skin hydration factor), desquamation, high cell turnover. During pediatric age, in various conditions both pathological and paraphysiologicalthe the function of the epidermal barrier can be inefficient, making the baby's skin susceptible to the onset of disease and vulnerable to chemical agents and microbial aggression. The cosmetics intended for children must therefore be designed and formulated in such a way as to preserve and respect the delicate skin balance typical of the little ones' skin, which is not yet completely mature and developed. In summary the dermo-cosmetological support in pediatric age is of primary importance for:

- Maintain good skin physiological status

- Soothe any irritative effects of both topical and systemic medical therapies

- Complete medical treatment

- Maintenance or rebalancing of the microbiome.

## A28 Pediatrics in Emilia-Romagna

### Giancarlo Cerasoli (giancarlo.cerasoli@libero.it)

#### pediatrician ASL Romagna, Italy

Emilia-Romagna was one of the first regions of Italy to promote the construction of childcare facilities.In the second half of the nineteenth century, settlements of shelter and marine colonies for children suffering from rickets and scrofula spread. At the beginning of the twentieth century the first pediatric hospitals were established (in Parma and Modena), the three pediatric clinics of Bologna, Parma and Modena and the pediatric divisions of the civil hospitals of Parma, Ferrara and Reggio-Emilia. As in the other regions of Italy, in the smaller cities of Emilia-Romagna, the creation of the first departments for the shelter and care of sick children took place inside the “Brefotrofi” e “Istituti Provinciali per l’infanzia”, while the clinics and dispensaries became part of the "Aiuti Materni" and spread especially after 1925, following the rules establishing the National Opera for Maternity and Childhood. The scientific activity of the first pediatricians from Emilia-Romagna is evident above all in the pages of national pediatric journals, four of which are published in this region, and in congresses organized by the Italian Society of Pediatrics, starting from the one held in 1890 in Rome.

## A29 Corticosteroid Inhalers: how we are using them

### Giovanni Cerimoniale (giovanni.cerimoniale@gmail.com)

#### Pediatra di Famiglia, Minturno, Italy

**Background**

This survey aimed at collecting information related to the therapy and management of respiratory diseases requiring the use of ICS, in view of a Consensus document endorsed by the main Pediatric Scientific Society soon to be drafted.

**Methods**

All the members of the Italian Society of Pediatrics (SIP) were submitted this on – line survey , collecting data about the use of ICS for the treatment of respiratory diseases

**Results**

One thousand-two pediatricians were involved in this survey, subdivided as follows: 392 (39%) primary care pediatricians, 388 (38%) hospital pediatricians, 31(3%) university pediatricians, 164 (16%) professional pediatricians, and 27 (3%) post-graduate students in Pediatrics.Good adherence to recommendations in literature for the treatment of allergic rhinitis was found: oral antihistamines are used by 607 (61%) of the interviewed in case of secretory allergic rhinitis while 649 (65%) use spray nasal corticoids for the obstructive type.For the treatment of the asthma attack ICS are not used by 54% of the interviewed sample, high dosage is used by 28% , low doses are used by 18% ICS are not used as a maintenance therapy for low recurret forms by 54% of the sample, low doses are used by 44% while high dosage is used by 2% . In case of frequent recurrence these percentages change: ICS are recommended in high doses by 67% of the sample and a low dosage is indicated by 31% of the interwieved.For recurrent wheezing, percentages were very similar for treatment only in case of need (49%) or as a maintenance therapy. Both for asthma and wheezing ,treatment time before control appeared affected by participants’ work setting. Closer controls were preferred by primary care, hospital or professionals if compared to university pediatricians. Preference for the use of some specific steroid molecules according to the different pathologies also emerged. Parents’influence on these choices resulted of no relevance.

**Conclusions**

Over 10% of S.I.P members were involved in this survey. A good management of the child affected by recurrent asthma or wheezing is shown as well as the choice of close control visits accounting for more accurate treatment adjustments to the small patient’s needs. An excessive use of corticosteroids for the treatment of asthma was found, expecially for the acute asthma attack as well as for the dosages used in the prevention of recurrences.

As a result of this survey, improvements in the implementation of good clinical practice is to be expected , triggering the organization of specific training courses.

## A30 Controversies in the tuberculosis screening in internationally adopted children

### Elena Chiappini, Barba Bortone, Luisa Galli

#### Meyer University Hospital, Department of Health Sciences, University of Florence, Florence, Italy

##### **Correspondence:** Elena Chiappini (elena.chiappini@unifi.it)

Infectious diseases are commonly reported in internationally adopted children (IAC). In this population, the estimated prevalence of tubercular disease is <1.0%, while the prevalence of latent tubercular infection (LTBI) is about 15%. The published screening protocols for tuberculosis in IAC are discordant and the use of tuberculin skin test (TST), Interferon Gamma Release Assay (IGRA), or both is still under debate. Several studies have been conducted to evaluate the performance of these tests, but in the absence of a gold standard test for the diagnosis of LTBI, no conclusive data are available. When both tests are performed, discordant results are difficultly interpreted. The possibility of discrepant results (mainly TST + / IGRA-) may be due to a lower specificity of TST compared to IGRA (which is not or minimally influenced by previous BCG vaccination, nor non-tuberculous mycobacterial infections, or repeated TSTs). However, although IGRAs showed overall higher specificity than TST, they may display a lower sensitivity in children under 5 years of age. In Italy, the national guidelines for the diagnosis of tuberculosis in immigrants, published in 2018, suggest the possible use of both TST or IGRA, even sequentially, but recommend the use of TST in immigrant children aged less than 5 years. Other experts, and the most recent US guidelines, suggest the use of IGRA even in children aged from 2 to 5 years, if a good follow-up can be assured. In young children. However, IGRA are the preferred assay when the tested children are unlikely to return for TST reading. Some experts suggest that an initial negative TST/IGRA should be repeated after 3 or 6 months from the child’s arrival, since the first test may be falsely negative, especially in recently infected and/or malnourished children.

## A31 Sarcomas in adolescents

### Stefano Chiaravalli (stefano.chiaravalli@istitutotumori.mi.it)

#### Pediatric Oncology Unit, Medical Oncology and Hematology Department, Fondazione IRCCS Istituto, Nazionale Tumori, Milan, Italy

Sarcomas represent a very heterogeneous group of mesenchymal malignant tumors that are relatively frequent in adolescents. Survival outcomes for adolescent with sarcomas lag behind those of children diagnosed with histologically similar tumours: for most histotypes, the survival

decreases with advancing age. The clinical management of these tumors may be complex in this age group, partly due to tumor associated factors, but also because of the management of these patients: delays in diagnosis, trial availability and participation, aspects of the organisation of care (with an emphasis on age-specific needs), national centralisation of sarcoma care, international consortia. As a matter of fact, different approaches have been adopted by pediatric and adult medical oncologists dealing with the same disease. However, nowadays constant efforts from international collaborations between pediatric and adult oncologists of sarcoma groups have optioned in converging towards a common therapeutic strategy, while improving quality of treatment, as well as research advances dedicated to this at-risk age group of patients with sarcomas. Two aspects may be of particular interest, in more details. First, the outcome of adolescents and young adults with rhabdomyosarcoma is decidedly worse than that generally seen in childhood. It is supposed that rhabdomyosarcoma patients, regardless of their age, would receive better treatment when following guidelines derived from the large pediatric experience. Second, the impact of new targeted agents in the pediatric population has not paralleled the progress seen in adult sarcoma patients. Increased international collaboration between pediatric and adult sarcoma groups is of critical importance to facilitate the transfer of potentially effective new agents from adults to children and adolescents, and to improve research programs dedicated to young patients.

## A32 Recent advances in precocious puberty

### Francesco Chiarelli, Nella Polidori

#### Department of Pediatrics, University of Chieti, Chieti, Italy

##### **Correspondence:** Francesco Chiarelli (chiarelli@unich.it)

Puberty is a period of transition characterized by the development of secondary sexual characteristics, maturation of the gonads and achievement of reproductive capacity. Precocious puberty has been defined as the onset of breast stage II development before the age of 8 years in girls and genital stage 2 development before 9 years in boys. The timing of onset of puberty in girls has been decreasing all over the world. In fact, although in the male the classic chronological limit of 9 years is more widely accepted, there has been a decrease of at least one year of the age of appearance of physiological puberty in the female determining a relevant discussion on the possible similar reduction of the cut-off for age of definition of precocious puberty in girls from 8 to 7 years. This secular trend, first recognized in the developed countries, has been noted in developing countries as well. This has been attributed to improved nutrition and effects of environmental endocrine disrupters. Based on the origin of pubertal activation, precocious puberty can be classified as gonadotropin-dependent (PPC), due to the early activation of the hypothalamic-pituitary-gonadal axis with consequent increase in the pulsatile secretion of LH and subsequent gonadal activation; or gonadotropin-independent (PPP), as expression of excessive production of sex hormones, without activation of the hypothalamic-pituitary-gonadal axis. Many factors are involved in pubertal initiation, but the understanding of pubertal regulation has undergone a sea change with the discovery of the kisspeptin system. Currently, mutations in the kisspeptin system, as MKRN3 and DLK1, have been identified in sporadic and familial cases of central precocious puberty. The diagnosis is based on physical exam findings indicating advancing puberty and on laboratory test. The gold therapeutic standard for children with PPC consists of agonists of hormones that release gonadotropin, used with the goal of regression or stabilization of the pubertal signs, decreases the growth rate, the progression of bone age and height preservation. To date, new pharmaceutical formulations have been developed in order to improve the adherence to treatment and its therapeutic efficacy that will soon change the approach to subjects with PPC in the coming years. Instead, PPP treatment is influenced by the causes determining the activation of puberty.However, many areas remain to be explored such as targeted therapies and aspects of clinical management. Further investigation into psychological effects and additional data regarding long-term outcomes, is needed.

## A33 Italian validation of the “Humpty Dumpty Fall Scale” and realization of a modified version. Simulation of a possible new scale

### Ciofi Daniele^1^, Biermann Klaus P^2^, Gabriele Frangioni^2^, Midea Ilaria E.^2^ , Albolino Sara^3^, Dagliana Giulia^3^, Ciardelli Alessia^4^ Filippo Festini^5^

#### ^1^ University of Tor Vergata, Roma, Italy; ^2^ Meyer Children Hospital, Florence, Italy; ^3^ Center for the Management of Clinical Risk, Tuscan government, Florence, Italy; ^4^ IRCSS Fondazione Gabriele Monasterio - Ospedale Pediatrico Apuano, Massa, Italy; ^5^ University of Florence, Italy

##### **Correspondence:** Ciofi Daniele (daniele.ciofi@meyer.it)

**Introduction**

Falls are a relevant problem in regards to frequency and severity in paediatric settings [1-7]. The prevention of Risk for Falls is a goal that Health Institutions and Health Professionals have to aim to (8,9). The prevention is only possible with an appropriate falls risk assessment instrument that identifies patients that need specific prevention measures whit specifics tools like as Pediatric Fall Risk Assessment Scales (PFRAS) [10-18]. The only existing document, the “Humpty Dumpty Fall Scale” (HDFS), is validated only in English [19].

**Materials and methods**

The study consists of 4 steps. First step: linguistic and cultural validation [20]. Second step: evaluation of the predictive properties. Third step: modifications of the scale to improve its performance. Fourth step: creation and simulation of a new scale with a better predictive performance, using epidemiological and clinical data gathered in step 2. For the validation we have used the forward-backward translation and a pilot test with nurses and a convenience sample of 100 paediatric inpatients for the evaluation of clarity, validity and reliability. During step 2 the validated Italian HDFS was used with 1500 hospitalized children in Tuscany [21, 22].

**Results**

The Italian version, HDFS-ita, had a validity of SICV=0,92 and an inter- rater reliability of K di Cohen=0,965. The predictive performance was poor (sensibility 77,8%, specificity 36,6%, AUC of the ROC curve: 0.593). A new version of the HDFS-ita, the HDFS-ita-M with only three items and a cut off of 7 was created to be used in subjects between 1 and 16 years. This scale had a better but still not satisfying performance in regards to predictivity (sensibility 77.8%, specificity 53.3% AUC of the ROC curve:0.670). With the data collected during step 2 a new scale was developed with satisfying statistical and clinical performances (sensibility 88.9%, specificity 41.9% AUC of the ROC curve:0.820). This 6 item scale with a cut off of 7 can only be used for subjects from 1 to 16 years. Its temporary name is “Meyer-Tuscany Fall Scale”.

**Conclusion.**

The HDFS-ita-M can immediately be implemented cancelling four of the 7 items of the HDFS-ita. The “Meyer-Tuscany Fall Scale” can be introduced after a test period and validity and stability inter-rater reliability measures, possibly with similar methods as in step 2 of this study.

**Acknowledgements.** We thank the members of the Tuscan Pediatric Fall Scale Study Group.

**References**

1. NHS Improvement. The incidence and costs of inpatient falls in hospitals. London; National Health System. https://improvement.nhs.uk/uploads/documents/Falls_report_July2017.v2.pdf . Accessed 15 July 2019.

2. Eventi sentinella nelle strutture del SSN: quarto rapporto. http://www.salute.gov.it/portale/news/p3_2_1_1_1.jsp?lingua=italiano&menu=notizie&p=dalministero&id=999 . Accessed 15 July 2019.

3. Nimityongskul P, Anderson LD. The likelihood of injuries when children fall out of bed. J Pediatr Orthoped. 1987;7:184-186.

4. Cooper CL, Nolt JD. Development of an evidence-based pediatric fall prevention program. Journal Nursing Quality Care. 2007;22:107-112.

5. Yu H, Wier LM, Elixhauser A. Hospital Stays for Children, 2009. HCUP Statistical Brief #118. Agency for Healthcare Research and Quality. 2011.

6. Hill-Rodriguez D, Messmer PR, Williams PD, Zeller RA, Williams AR, Wood M, et al. The Humpty Dumpty Falls Scale: a case-control study. J Spec Pediatr Nurs. 2009;14:22-32.

7. Schaffer PL, Daraiseh NM, Daum L, Mendez E, Lin L, Huth MM. Pediatric inpatient falls and injuries: a descriptive analysis of risk factors. J Spec Pediatr Nurs. 2012;17:10-8.

8. Pomerantz WJ, Gittelman MA, Hornung R, Husseinzadeh H. Falls in children birth to 5 years: different mechanisms lead to different injuries. J Trauma Acute Care Surg. 2012;73:254-7.

9. The Joint Commission International. JCI Accreditation Standards for Hospitals. 4^th^ edition. U.S. Joint Commission International. 2010.

10. Razmus I, Wilson D, Smith R, Newman E. Falls in hospitalized children. Pediatr Nurs. 2006;32:568–572.

11. Cummings R. An Evidence-based Approach to Fall Risk Assessment. Presented at 17th International Nursing Research Congress Focusing on Evidence-Based Practice. Montreal, Quebec, Canada. 2006.

12. Razmus I, Davis D. The epidemiology of falls in Hospitalized children. Pediatr Nurs. 2012;38:31-5.

13. Graf E. Pediatric hospital falls: Development of a predictor model to guide pediatric clinical practice. Presented at 38th Sigma Theta Tau International Biennial Convention, Indianapolis, US. 2005.

14. Graf E. Identifying Predictor Variables Associated with Pediatric In-patient Fall Risk Assessments. Presented at 5th National Conference evidence-based Fall Prevention Conference. Clearwater, Florida, Us. 2006.

15. Graf E. Inpatient Pediatric Fall Assessment & Interventions: What We Know so Far. Presented at 8th Annual Transforming Fall Prevention Practices. Clearwater, Florida, US. 2007.

16. Harvey K, Kramlich D, Chapman J, Parker J, Blades E. Exploring and evaluating five paediatric falls assessment instruments and injury risk indicators: an ambispective study in a tertiary care setting. J Nurs Manag. 2010;18:531-41.

17. Neiman J, Rannie M, Thrasher J, Terry K, Kahn MG. Development, implementation and evaluation of a comprehensive fall risk program. J Spec Pediatr Nurs. 2011;16:130–139.

18. Ryan-Wenger NA, Kimchi-Woods J, Erbaugh MA, LaFolette L, Lathrop J. Challenges and conundrums in the validation of pediatric fall risk assessment tools. Ped Nurs. 2012;38:159–167.

19. Wood M, Hill-Rodriguez D, Messmer P, et al. Implementing a Humpty Dumpty Falls Scale for Pediatric Patients. 17th International Nursing Research Congress Focusing on Evidence-Based Practice. Montreal, Quebec, Canada. 2006.

20. Sousa V, Rojjanasrirat W. Translation, adaptation and validation of instruments or scales for use in cross-cultural health care research: a clear and user friendly guideline. Journal of evaluation in clinical practice. 2011;17:268-274.

21. Polit DF, Tatano Beck C. The Content Validity Index: are you sure you know what’s being reported? Critique and recommendations. Res Nurs Health. 2006;29:489-497.

22. Abramson JH. WINPEPI (PEPI-for-Windows): computer programs for epidemiologists. Epidemiol Persp Innov. 2004;1:6.

## A34 Diagnosis of recurrent respiratory infections

### Carlo Concato^1^, Stefania Ranno^1^, Livia Piccioni^1^

#### Virology Unit, Department of Laboratories, Bambino Gesù Children’s Hospital – IRCCS, Rome, Italy

##### **Correspondence:** Carlo Concato (carlo.concato@opbg.net)

Acute respiratory infections (ARI) represent the main cause of morbidity and hospitalization in pediatric patients and in 2016 were the second leading cause of death in children between 1-5 years after prematurity, with an etiology attributable (95% IU) to Streptococcus pneumoniae (52.25%), Haemophilus influenzae B (7.36%), RSV (6.29%) and influenza virus (1.28%) [1, 2, 3].

The Italian Society of Pediatrics, among the criteria to define a child suffering from recurrent respiratory infections, indicates the following conditions:

- more than six respiratory infections/year;

- more than a respiratory infection of the upper airways per month between September and April;

- more than three respiratory infections of the lower airways/year.

The etiological agents vary depending on the respiratory tract affected and in 80% of the cases it is because of a virus, a percentage that rises up to 95% in infections of the upper airways. In the lower respiratory tract from 4.5 to 40% of cases there is a positive for bacteria, even in these cases up to 50% of patients have a concomitant or previous viral infection. The engraftment of respiratory viruses is favored by a genetic predisposition or by exposure to environmental factors and determines a situation of local inflammation, which in turn favors the entry of other viruses and respiratory bacteria, shaping a real infection of the respiratory tract. The main microorganisms responsible for respiratory infections are: RSV, Influenza A/B virus, Parainfluenza virus, Adenovirus, Enterovirus, Metapneumovirus and Bocavirus. As for the bacteria Streptococco pyogenes, Pneumococcus, Haemophilus Influenzae, Mycoplasma pneumoniae and Chlamydia pneumoniae. Although with a non-predominant pathogenic significance the most detected virus is the Rhinovirus, present in 45% of infections involving the upper airways, often in co-infection with other microorganisms [4, 5].

An accurate medical history is essential for the selection of suitable diagnostic tests. The Laboratory offers the possibility of performing first-level tests (rapid tests for antigen research) useful for immediate screening of a single pathogen (VRS, Influenza, SGA). Since the symptoms associated with ARI are common in viral, bacterial and fungal infections, it is particularly useful to use tests that allow a wide-ranging investigation to be performed for the differential diagnosis between different respiratory infections and other diseases. Molecular assays (real-time multiplex PCR) respond to this need as they allow to quickly distinguish between the different etiologies favoring a correct management of the therapy and where necessary the isolation of the patient [6].

**References**

1) Williams BG, Gouws E, Boschi-Pinto C, Bryce J, Dye C. Estimates of world-wide distribution of child deaths from acute respiratory infections. Lancet Infect Dis. 2002;2:25-32.

2) Walker CLF, Rudan I, Liu L, Nair H, Theodoratou E, Bhutta ZA, et al. Global burden of childhood pneumonia and diarrhoea. Lancet. 2013;381:1405-1416.

3) GBD 2016 Lower Respiratory Infections Collaborators. Estimates of the global, regional, and national morbidity, mortality, and aetiologies of lower respiratory infections in 195 countries, 1990-2016: a systematic analysis for the Global Burden of Disease Study 2016. Lancet Infect Dis. 2018;18:1191-1210.

4) Gaunt ER, Harvala H, McIntyre C, Templeton KE, Simmonds P. Disease burden of the most commonly detected respiratory viruses in hospitalized patients calculated using the disability adjusted life year (DALY) model. J Clin Virol. 2011;52:215-21.

5) de Benedictis FM, Bush A. Recurrent lower respiratory tract infections in children. BMJ. 2018;362.

6) Reddington K, Tuite N, Barry T, O'Grady J, Zumla A. Advances in multiparametric molecular diagnostics technologies for respiratory tract infections. Curr Opin Pulm Med. 2013;19:298-304.

## A35 Vaccination in healthcare workers: who, what, when

### Michele Conversano, Tatiana Battista, Carmela Russo

#### Dipartimento di Prevenzione ASL TA, Taranto, Italy

##### **Correspondence:** Michele Conversano (michele.conversano@asl.taranto.it)

Healthcare professionals represent important behavior patterns and a reliable source of scientific knowledge that can provide accurate and accurate information to patients and the community. The numerous professional figures involved and the multiplicity of procedures and tasks carried out make the definition of risks for personnel working in the health sector very complex. Among the risks associated with socio-health care, the infectious one, occupies a special place due to the complexity of the determinants, the increasing epidemiological trend due to the conditions of coexistence in closed environments, the increase in the proportion of immunocompromised, fragile and / or elderly, the increased frequency of invasive therapeutic diagnostic procedures, the spread of antibiotic-resistance phenomenon. In the health work context, in particular, the vaccination protection of the operator is accompanied by the maintenance of a functioning health system and the protection of patients, especially at high risk, by limiting the transmission of the disease in a semi-open population. An assessment of the adhesion determinants shows that health professionals are willing to get vaccinated if they believe in the protection given by the vaccine, if they are worried about their patients and their families, if they have easy access to vaccines, if the vaccines are provided for free.

According to health professionals it is fundamental: a clear institutional vaccination strategy, on all prevention issues and which regularly involves competent doctors, hygienists and hospital infection control personnel.

To encourage adherence to vaccination in the health sector, necessary actions are required: sending an invitation letter, availability / dissemination of a vaccination schedule, offering incentives, such as: vacation days / free hours, promotional material ( pens, pins, stickers, screen savers), repeat vaccination campaigns regularly, contact all new employees and medical, nursing and other health professions students.

It is important to favor an active role of the competent doctor in guiding workers towards choices and behaviors favorable to health, including vaccinations. Through the computerized vaccination registry, the competent doctor will be able to know the vaccination status of each health worker for the correct management of the programs and the improvement of vaccination coverage. In this process it is also important to disclose the consequences of non-vaccination, through prevention campaigns that include elements such as the refusal form or the obligation to wear masks for unvaccinated workers, which has led to an increase in the use of vaccines in the field hospital. To ensure a policy of disseminating vaccination compliance in operators in the various health facilities, it is necessary to invest in their technical and scientific training on vaccinations and on the ability to communicate and interact with users on prevention issues. A good knowledge of all aspects (eg use of vaccines, medical legal aspects of vaccination or lack of vaccination) ensures the collaboration of health personnel with adequate training in the various medical, surgical and emergency branches, raising awareness on the rationale of vaccination for subjects at risk for condition and pathology.

**References**

1. Center for Disease Control and Prevention, Immunization of Health--Care Personnel. Recommendations of the Advisory Committee on the Immunization Practices (ACIP). MMWR 2011;60:1- 46.

2. La Torre G, Scalingi S, Garruto V, Siclari M, Chiarini M, Mannocci A. Knowledge attitude and behaviours towards recommended vaccinations among healthcare workers. Healthcare (Basel). 2017;7:5.

3. Fortunato F, Tafuri S, Cozza V, Martinelli D, Prato R. Low vaccination coverage among italian healthcare workers in 2013. Hum Vaccin Immunother. 2015;11:133-9.

## A36 Child protection unit “bambi”: an over ten years’ experience

### Coppo E^1^, Racalbuto S^1^, Cavecchia I^1^ , Malabaila A^1^ , Urbino FA^2^

#### ^1^Department of Pediatric Emergency, “Bambi” Unit, A.O.U. “Città della Salute e della Scienza”, Turin, Italy; ^2^ Department of Pediatric Emergency, A.O.U. “Città della Salute e della Scienza”, Turin, Italy

##### **Correspondence:** Coppo E (ecoppo@cittadellasalute.to.it)

Child Abuse (CA) represents a diagnostic challenge due to its complex presentation. Although the consequences of failing to diagnose CA may be life-threatening, a wrong diagnosis may have devastating sequelae too [1,2,3]. The Child protection unit “Bambi” is located at “Regina Margherita” Children Hospital in Turin and it is a multidisciplinary team composed by pediatricians, psychologists, nurses, social workers and medical examiners. The aim of the Bambi unit is to recognize and diagnose child abuse in all its forms (sexual abuse, maltreatment, neglect). Every year, in the last years, above 200 children are visited in the unit. This number is increasing constantly; we think this is not related to the increasing of the phenomenon but a result of a better detection of cases from social workers, prosecutors, doctors, teachers and police. CA diagnosis is always a multidisciplinary diagnosis, since a single element is not enough to detect abuse, and multiple evaluations are required [4,5,6]. From 2012 to 2017, the Bambi unit visited 1294 children from 0 to 18 years old suspected to be abused: 65% children were females. Among this children 43% were evaluated because of suspicion of sexual abuse, 38% because of maltreatment, 6% neglect, 7% more than one kind. Regarding sexual abuse, the incidence is higher in females (82%), while maltreatment is equally experienced by both genders (50%). Mean age for children suspected to be abused was 7y5m, for maltreatment 7y4m, for neglect 6y6m. The median age of patients was 7y5m. From 2015 to 2018, we saw 719 children, among whom 37,8% reported intra-familiar physical maltreatment, 3% extra-familiar physical maltreatment, 12,1% extra-familiar sexual abuse, 31,2% intra-familiar sexual abuse, 14,6% neglect, 2,2% intoxication.

About 80% of the children seen in the unit are reported to the authorities due to suspicion of any form of abuse[7].

**References**

1.Offiah A, Van rijn RR, Perez-Rossello JM, Kleinman PK. Skeletal imaging of child abuse (non-accidental injury). Pediatr Radiol. 2009;39:461-70.

2. Moody G, Cannings-John R, Hood K, Kemp A, Robling M. Establishing the international prevalence of self-reported child maltreatment: a systematic review by maltreatment type and gender. BMC Public Health. 2018;18:1164.

3. Preventing child maltreatment: a guide to taking action and generating evidence. World health organization. 2006. https://www.who.int/violence_injury_prevention/publications/violence/child_maltreatment/en/

Accessed 8 August 2019.

4. World Health organization. Management of substance abuse unit. Global status report on alcohol and health, 2014. World Health Organization, 2014. https://www.who.int/substance_abuse/publications/alcohol_2014/en/ Accessd 8 August 2019.

5. Bellis MA, Hughes K, Leckenby N, Perkins C, Lowey H. National household survey of adverse childhood experiences and their relationship with resilience to health-harming behaviors in England. BMC Med. 2014;12:72.

6. Arias I. Report from the CDC. The legacy of child maltreatment: Long-term health consequences for women. Journal of Women's Health. 2004; 13: 468-473.

7. Autorità Garante per l’Infanzia e l’Adolescenza, CISMAI & Fondazione Terre des Hommes Italia. Indagine nazionale sul maltrattamento dei bambini e degli adolescenti in Italia. 2015. www.terredeshommes.it/dnload/Indagine-Maltrattamento-bambini-TDH-Cismai-Garante.pdf Accessed 8 August 2019.

## A37 Structuring a questionnaire for a comprehensive needs assessment tool of unaccompanied minors migrants hosted in Italian reception facilities

### Leuconoe Grazia Sisti^1,2^, Emanuela Maria Frisicale ^1,2,3^, Alice Corsaro ^2,5^, Drieda Zace ^1,2^, Andrea Gentili ^1,2^, Luca Giraldi ^1^, Stefania Bruno^1,2^, Simona La PLaca ^4,5^ Maria Luisa Di Pietro ^1,2^, Stefania Boccia ^1,6^

#### ^1^Università Cattolica del Sacro Cuore, Department of Public Health, Rome, Italy; ^2^Center of research and study on Global Health- Università Cattolica del Sacro Cuore - Rome, Italy; ^3^Local Health Authority - ASL Roma 1, Rome, Italy; ^4^University Hospital “Policlinico P.Giaccone”, Palermo, Italy; ^5^National Working Group for Migrant Children, Italian Society of Paediatrics; ^6^Fondazione Policlinico Universitario “A.Gemelli” IRCCS, UOC Igiene Ospedaliera, Rome, Italy

##### **Correspondence:** Leuconoe Grazia Sisti (leuconoe.sisti@gmail.com)

**Background**

The unaccompanied minors migrants (UMMs) flow in Italy has increased significantly since 2014 with 12,212 UMMs’ arrivals reported in 2018. The Italian ministerial guidelines on reception of foreigners recognize UMMs particular vulnerability, due both to developmental age and migrant status, and define the areas of need to be investigated during the stay of minors in reception facilities.However, they do not suggest clear and shared tools to use.

**Objective**

Based on the socio-economic determinants of the health paradigm, the aim of our study was to structure a shared and comprehensive tool for evaluating UMMs’ needs, in order to support children assessment in reception facilities.

**Methods**

Results of a systematic review on need assessment tools for UMMs’, performed on Pubmed, Scopus and grey literature, were discussed with the National Working Group for Migrant Children of the Italian Society of Paediatrics in order to develop an original tool. This was successively submitted to a panel of experts (Paediatrician, Neuropsychiatrist, Social Operators, Bioethicist) using the Delphi methodology in two rounds.

**Results**

Most of the questionnaires found in the literature focused only on aspects related to psychological health (80% of the 27 studies included in the analysis). On the contrary, the questionnaire produced by our working group investigates physical, mental, spiritual health and legal, educational and social needs, focusing on the subjective perception of well-being and needs satisfaction. This questionnaire, still undergoing the validation phase, has been planned to be self-administered with the support of cultural mediators and/or operators of the reception facilities.

**Conclusions**

Given the absence of validated and shared tools, the validation of a questionnaire that evaluates the overall needs of UMMs is necessary both to improve and standardize the assessment, and to evaluate indirectly the hosting country ability to meet and to respond adequately to the detected needs.

## A38 Feeding issues in late preterm infants

### Luigi Corvaglia (luigi.corvaglia@unibo.it)

#### Department of Medical and Surgical Sciences, University of Bologna, Bologna, Italy

Neonatal Intensive Care Unit, S.Orsola-Malpighi Hospital, Bologna, Italy

Moderate (gestational age [GA] 32-33^+6^ weeks) and late (GA 34-36^+6^) preterm infants (LPIs) account for over 80% of preterm infants born worldwide, with a 20% increase in LP births documented in the US over a ten years period. Even if many LPIs are similar in size to term infants, they are physiologically immature and have higher risk of developing medical complications which result in higher mortality, morbidity, and hospital readmission. Until recently, most of the scientific literature has focused on more immature and higher-risk infants, such as very-low-birth-weight infants, and very little attention has been paid to respiratory and nutritional issues affecting LPIs.

Many LPIs require early nutritional support, as, for these infants, gestation is interrupted during a period of intense gastrointestinal (GI) development. For this reason, LPIs present with a variable degree of GI immaturity, including inability to coordinate suck, swallow and breathing, which makes oral feeding quite challenging, and alterations in GI motility and absorption. Difficulties in achieving an adequate nutrition translate into faltering growth during the neonatal period and into a higher risk of short stature in the first years of life. Furthermore, they have potential negative consequences in terms of neurodevelopment, because brain structural and functional development are not fully completed when LP birth occurs. Nutritional requirements of LPIs have been shown to be greater than those of full-term infants. Breastfeeding represents the optimal nutritional choice also for LPIs, but effective milk transfer is often impaired by GI immaturity of these infants. Therefore, LPIs and their mothers require additional and specific breastfeeding support, which should be provided by adequately trained personnel. However, when breastfeeding alone is insufficient to satisfy the elevated nutritional requirements of LPIs, additional milk intake should be provided, when possible, with supplemental expressed own mother’s milk. When own mother’s milk is lacking, the choice of an adequate infant formula is fundamental, and should be individualized considering the infant’s characteristics and specific nutritional needs. During hospital stay, the provision of an infant formula containing long chain polyunsaturated fatty acids is recommended, while, after hospital discharge, the use of a post-discharge formula, which has a higher calorie and protein content than a term formula, should be encouraged especially in infants small for gestational age or with significant extrauterine growth retardation.

## A39 Diabetes and its oral complications

### Micaela Costacurta, Raffaella Docimo

#### Department of Surgical Sciences, University of Rome Tor Vergata, Rome, Italy

##### **Correspondence:** Micaela Costacurta (micaela.costacurta@uniroma2.it)

**Background**

Diabetes mellitus is associated with various oral manifestations in paediatric subjects too. Some oral complications, such as periodontal disease, may be a possible risk factor for poor metabolic control in subjects with diabetes and can lead to a two-way relationship.

**Materials and methods**

The authors reviewed the literature to classify oral conditions related to diabetes mellitus in paediatric age. Studies that had low or no association between diabetes mellitus and oral manifestations were included too.

**Results**

In paediatric subjects, diabetes mellitus is associated with different frequency to the following oral clinical manifestations: periodontal disease (increase of *Plaque Index*, *Gingival Index*, *Bleeding on Probing*), dental caries (increase of *dmft /DMFT*), salivary changes (decreased of salivary flow), alterations of the oral microbiome, pulp inflammation, periapical pathology, taste alterations, neuro-sensory alterations, alteration of dental eruption times. The frequency of some oral manifestations is related to the time of diagnosis and the glycaemic control of diabetes mellitus.

Particular attention was paid to salivary changes in the diabetic subject and the two-way relationship between diabetes mellitus /periodontal disease and diabetes mellitus /periapical pathology, in particular for preventive and interceptive purposes.

Some studies present conflicting results on the association between diabetes mellitus and dental caries.

**Conclusions**

The prevention and interception of some oral complications of diabetes mellitus are fundamental both for oral health, but also for the general health of the subject, influencing positively glycaemic control.

## A40 Development of infants oral feeding: towards safe nutritive sucking

### Francesco Cresi, Vito Andrea Dell’Anna, Elena Maggiora, Elena Grosso, Federica Logrippo, Enrico Bertino, Alessandra Coscia

#### Neonatology and Neonatal Intensive Care Unit, Sant’Anna Hospital, Città della Salute e della Scienza, University of Turin, Turin, Italy

##### **Correspondence:** Francesco Cresi (francesco.cresi@unito.it)

Newborn feeding is a complex and dynamical system that requires the proper integration of physical and neurophysiologic functions involved in the sucking pathway [1]. Sucking may be nutritive or nonnutritive as it involves milk transport or not, respectively. Mature sucking includes two components, suction and expression. Suction corresponds to the negative intraoral pressure that draws milk into the mouth. Expression corresponds to the compression and/or stripping of the tongue against the hard palate to eject liquid into the mouth [2]. During nutritive sucking, the correct synchrony of sucking, swallowing, breathing, and esophageal activities is critical for safety in order to prevent simultaneous milk entry into the trachea and esophagus. Efficient nutritive sucking leads to increased transition from tube to breast feeding, shorter hospital stays and safe discharge. On the other hand, nonnutritive sucking matures earlier and occurs at a faster frequency (2 cycles/s) than nutritive sucking (1 cycle/s). It is an activity confined within the oral cavity that is independent of the swallow, respiratory, and esophageal functions. Swallows are minimally involved and no milk is ingested as the pharyngeal phase of swallowing is not activated, allowing sucking and respiration to function at a more rapid pace. Therefore, nonnutritive sucking is a good marker for sucking itself but is not predictive of nutritive sucking competency and readiness to oral feeding [3]. Healthy premature infants typically achieve independent oral feeding skills by 36–38 weeks of postmenstrual age while extremely preterm and infants who have neurodevelopmental problems or other comorbidities (pulmonary bronchodysplasia, cardiopathies, GERD) often require longer time. The prevalence of dysphagia in premature infants born at <37 weeks of gestation and preterm with a very low birth weight (<1500 g) is around 10.5% and 24.5%, respectively. [4] The American Academy of Pediatrics stated that oral feeding sufficient to support appropriate growth together with the ability to maintain normal body temperature and sufficiently mature respiratory control are the essential physiologic competencies before hospital discharge of the preterm infant [5]. Consequently, neonatal dysphagia or abnormalities of swallowing are associated with delayed full oral feeding, longer hospital stays and tube feeding at discharge.

**References**

1. Engstler K, Perez J, Goldfield E. Neonatal Feeding Behavior as a Complex Dynamical System. Semin Speech Lang. 2017;38:77-86.

2. Lau C. Development of Suck and Swallow Mechanisms in Infants. Ann Nutr Metab. 2015;66:7-14.

3. Lau C. Development of infant oral feeding skills: what do we know? Am J Clin Nutr. 2016;103:616S-621S.

4. Mizuno K, Ueda A. The maturation and coordination of sucking, swallowing, and respiration in preterm infants. J Pediatr. 2003;142:36-40.

5. Committee on Fetus and Newborn. Hospital Discharge of the High-Risk Neonate. Pediatrics. 2008;122:1119-1126.

## A41 Outcomes of conservative treatment in children with acute appendicitis

### Carolina D’Anna^1^, Letizia Zenzeri^1^, Maria Concetta Lonardo^2^

#### ^1^Emergency Department, Santobono Pediatric Hospital, Naples, Italy; ^2^Department of Translational Medical Science, Section of Pediatrics, University of Naples "Federico II," Naples, Italy

##### **Correspondence:** Carolina D’Anna (dannacarol@alice.it)

**Introduction**

Acute appendicitis (AA) is still the most common indication as surgical emergency in children [1]. For over 100 years, surgical removal of the appendix has been considered a dogma [2]. 2 Nowadays there is a growing consensus in literature to suggest that a conservative treatment using antibiotics may be effective as well as surgical treatment in adult patients with diagnosis of AA [3]. Data in pediatric population are scarce [4]. The main aim of our retrospective study was to evaluate if abdominal ultrasonographic specific findings associated to clinical and laboratory examination improve diagnostic accuracy of acute uncomplicated appendicitis (AUA). As second aim we evaluated the efficacy of conservative treatment of AUA.

**Methods**

In this retrospective study all patients with diagnosis of AUA who received a conservative treatment were enrolled. Data collected were: duration of symptoms, sign and symptoms at the diagnosis, findings at abdominal ultrasonography, type of treatment performed (conservative vs surgical treatment), follow-up at 24/48 hours and follow-up at 6 and 12 months.

**Results**

Of a total of 1131 admissions for abdominal pain, only 551 were AA (412 complicated vs 139 uncomplicated). All of the AUA (139 patients) at the diagnosis presented the typical findings at abdominal ultrasonography, and every patient were treated with antibiotics in the first 24 hours. After 24/48 h only the 22% underwent surgical treatment while the 78% (109) were responder-to-treatment and consequently dismissed continuing the antibiotics at home. Only 6 of 109 patients during the long-term follow-up were admitted for relapsing symptoms. 2(33%) of them after 6 months newly started antibiotic therapy while 1(16%) at twelve months of follow-up underwent surgical treatment for complicated appendicitis and 3(50%) during the long-term follow-up didn’t received any treatment.

**Conclusions**

Clinical and laboratory examinations are not sufficient for the diagnosis and they can only suggest the diagnosis of AA. Nowadays ultrasonography is the gold standard for diagnosis of AA and especially in order to distinguish if appendicitis is complicated or not. We observed in our study that the severity of appendicitis correlates with decreased submucosal integrity rather than with appendiceal diameter. Moreover, US is useful to discriminate between patients responding or not responding to conservative treatment. The high rate of responder to antibiotic treatment showed how conservative treatment is safe and effective in patients with AUA. Therefore, for a better therapeutic outcome, it is essential to make a proper selection of patients who can be treated conservatively.

**References**

1.St Peter SD, Sharp SW, Holcomb GW, Ostile DJ. An evidence-based definition for perforated appendicitis derived from a prospective randomized trial. J Pediatr Surg. 2008;43:2242–5.

2. Emil S, Laberge JM, Mikhail P, Baican L, Flageole H, Nguyen L, Shaw K. Appendicitis in children: a ten-year update of therapeutic recommendations. J Pediatr Surg. 2003;38:236–42.

3. Georgiou R, Eaton S, Stanton MP, Pierro A, Hall NJ. Efficacy and safety of Nonoperative treatment for acute appendicitis: a Meta-analysis. Pediatrics. 2017;139: pii: e20163003.

4. Fugazzola P, Coccolini F, Tomasoni M, Stella M, Ansaloni L. Early appendectomy vs. conservative management in complicated acute appendicitis in children: A meta-analysis. J Pediatr Surg. 2019; pii: S0022-3468(19)30125-3.

## A42 A problem at the Emergency Department: hyperglycemia in diabetic child

### Giuseppe d’Annunzio, Nicola Minuto, Mohamad Maghnie

#### Pediatric Clinic, Regional Center for Pediatric Diabetes, IRCCS Istituto Giannina Gaslini, Genoa, Italy

##### **Correspondence:** Giuseppe d’Annunzio (giuseppedannunzio@gaslini.org)

Despite increasing knowledge about the disease pathogenesis, diabetic ketoacidosis (DKA) is frequently observed in newly-diagnosed Type 1 Diabetes Mellitus (T1DM), requiring hospitalization and strict management, sometimes in Intensive Care Unit. After DKA recovery and educational interventions, patients are regularly followed in the outpatient section. Otherwise several conditions, sometimes life-threatening, cause acute metabolic decompensation in established diabetes, requiring hospitalization. The incidence of recurrent DKA in the US has been estimated at 8 per 100 person-years. As regards insulin treatment, one problem frequently encountered is Continuous Subcutaneous Insulin Infusion (CSII) malfunctioning, responsible for acute DKA. In 2013 among 13.9% Italian children and adolescents with T1DM on CSII a rate of pump failure 0.165 failure patient-year has been reported. When infusion is interrupted the lack of subcutaneous long-acting analogue insulin reserve, available in patients on basal-bolus regimen, is responsible for metabolic imbalance quickly leading to hyperglycemia. Frozen insulin, due to inappropriate vials storage, is another cause of acute DKA, especially in CSII users. Insulin allergy, otherwise rarely encountered after availability of human insulin preparations, is another serious adverse event requiring hospitalization, with a prevalence of 2.4%. Insulin allergy could elicit immediate reactions, usually induced by an IgE-mediated mechanism. Clinical presentation includes erythema and swelling at site of injection, urticaria, angioedema, rhinitis, bronchospasm and anaphylaxis, requiring urgent desensitization treatment. Another cause of acute metabolic decompensation is intentional insulin omission as inappropriate compensatory behaviour, mostly in females, for weight loss as expression of eating disorder including “diabulimia”, or other severe psychological disturbances. Other acute complications leading to hospitalization in long-standing patients include acute appendicitis, pancreatitis and kidney injuries. As regards neurological involvement acute chorea and central venous thrombosis have been described. Patients with T1DM with adequate metabolic control are not more susceptible to infections as compared with peers, otherwise infectious diseases may lead to DKA up to 32% of cases. The infections include bacteria (49%), viruses (49%), and tuberculosis (2%). DKA with tuberculous meningitis, herpes simplex type 2 encephalitis and group B streptococcal meningitis have also been reported. Ileo psoas abscesses, rhinocerebral mucormycosis have been described. A retrospective analysis using the Pediatric Health Information System (PHIS) database on youth with DM who presented to the Emergency Department (ED) or were hospitalized for infection management from 2008 to 2014 reported respiratory infections were the most common type of infection followed by skin and soft tissue infections for both ED care and inpatient hospitalizations. Hospital admission for DKA may be influenced by a wide spectrum of risk factors. Identification of valid predictors for DKA may offer the opportunity to prevent both acute complications of type 1 diabetes in high-risk individuals.

## A43 The revolutions of genetic prenatal diagnosis

### Bruno Dallapiccola (bruno.dallapiccola@opbg.net)

#### Scientific Directorate, Bambino Gesù Children Hospital, Rome, Italy

The declining birth rates in all Mediterranean countries, with Italy bringing up the rear, is paralleled by an increasing demand of prenatal testing aiming at monitoring the embryo’s and fetus’s wellness. In general, these tests are analyzing either the estimated 3% risk of defects or genetic diseases’ pending in each pregnancy, or the risks related to family history or maternal lifestyle and pregnancy history. Notable risk factors include advanced parental age, which increases the chance of de novo mutations, and segregation of distinct mutations from parents. A self-evaluation assessment based on family and maternal/pregnancy history is adequate for establishing if a given pregnancy has a standard or an increased genetic risk, thus demanding targeted monitoring. Advances in genetics, in particular increased availability of next generation sequencing (NGS) techniques, has widened the possibility to investigate in deep the genetic make-up of embryo and fetus, thus changing the traditional prenatal testing protocols. The main revolutions in prenatal diagnosis include the following: 1. Anticipation of diagnosis, from second to first trimester to preimplantation stage. Technical miniaturizing allows investigation of the genome based on single cells’ analysis. Researches are attempting to edit the diseased embryos, rising ethical concerns about precision and safeness of this experimental approach. 2. The shift from invasive to non-invasive diagnosis, based on the screening of biochemical markers, and, in recent years, of free fetal DNA in maternal blood. These tests predict with variable accuracy the risk of aneuploidy, or the presence of some genomic imbalances and Mendelian disorders. 3. The shift from a risk-guided to a technology-driven prenatal diagnosis. Availability at reduced costs of NGS has increased the demand of exome and genome sequencing often overlooking the limits of these techniques in term of incomplete coverage of critical genes and inability to interpret many variations of uncertain significance. 4. The shift from “prevention” of birth of an affected newborn based on pregnancy termination, to disease’s cure based on new drugs and gene therapy. For several diseases target of prenatal diagnosis, including cystic fibrosis, spinal muscular atrophy, thalassemia and others, promising precision medicine protocols are becoming available, arguing for the need of continuous update of the prenatal genetic testing scenarios, in order to offer to prospective parents the best opportunities.

## A44 Fragile X syndrome

### Stefano D’Arrigo, Claudia Ciaccio, Valeria Tessarollo, Chiara Pantaleoni

#### Developmental Neurology Department, IRCCS Fondazione Istituto Neurologico “C. Besta”, Milan, Italy

##### **Correspondence:** Stefano D’Arrigo (stefano.darrigo@istituto-besta.it)

Fragile-X syndrome (FXS) is one of the most frequent inherited cause of intellectual disability with a prevalence of about 1:5,000-7000 in men and 1:4,000-6,000 in women. FXS is caused by an alteration of the Fragile X Mental Retardation-1 gene (FMR1) on Xq27.3 band. More than 99% of individuals with FXS have a FMR1 loss- of-function, caused by an increased number of CGG repeats in the 5'untraslated region (typically >200). This “full mutation” determines a hypermethylated state of the deoxycytosine residues located at the FMR1 promoter. This results in loss or heavy reduction of the protein product (FMRP) and gives rise to the expression of the cytogenetic fragile site (FRAXA). The disease is caused by the absence of the FMRP protein. Different kinds of FMR1 alterations (deletions encompassing the gene, intragenic deletions/duplications, single-nucleotide variants) are responsible for the remaining molecular diagnosis of FXS. Genetic tests generally focus on the number of CGG repeats and the FMR1 alleles are usually categorised according to this number in Normal (5-44 repeats), Intermediate (45-58 repeats) and Pre-mutation (59-200 repeats) alleles. The probability of affected offspring is greater the more repeats are.

Fragile-X syndrome shows a clear genotype-phenotype correlation. In male full mutation children (hemizygotes) clinical picture is characterised by moderate development delay/intellectual disability, prevalently involving language, typical cranio-facial dysmorphisms and an high prevalence of neuropsychiatric disorders [1]. Particularly social anxiety is frequent and often associated to other autistic-like features with about 60% of affected males reaching diagnostic criteria for autism spectrum disorder [2]. Anyway fragile-X children maintain an adequate preferential relationship with his main caregivers and show milder social impairment than autistic children without the syndrome. About 20% of patients are affected by epileptic seizures with onset in early infancy. Different kind of seizures are reported and these are usually pharmacoresponsive and self‐limited, disappearing in adolescence.

The syndrome maintains typical post-pubertal phenotypic characteristics. It is possible to observe an increase of the hyperactivity and social anxiety, onset of obsessive-compulsive and, in the most severe cases, psychotic symptoms. Mitral valve prolapse has been observed in 50% of adult patients. Female full mutation patients (heterozygotes) show a mild physical and behavioural phenotype. Premutation patients generally have a normal appearance and normal intelligence but half of them shows neuropsychiatric disorders [3]. Female carriers can have a premature ovarian failure, while a late-onset ataxic syndrome with tremor has been reported in premutation male carriers.

**References**

1. Lubala TK, Lumaka A, Kanteng G, Mutesa L, Mukuku O, Wembonyama S, et al. Fragile X checklists: A meta-analysis and development of a simplified universal clinical checklist. Mol Genet Genomic Med. 2018. doi: 10.1002/mgg3.398.
2. Niu M, Han Y, Dy ABC, Du J, Jin H, Qin J, et al. Autism symptoms in Fragile X Syndrome. J Child Neurol. 2017; 2:903-909.
3. Hagerman RJ, Protic D, Rajaratnam A, Salcedo-Arellano MJ, Aydin EY, Schneider A. Fragile X-Associated Neuropsychiatric Disorders (FXAND). Front Psychiatry. 2018;9:564.

## A45 Born in Italy today

### Mario De Curtis, Daniela Regoli, Lucia Dito

#### Dipartimento Materno Infantile, Università di Roma La Sapienza, Policlinico Umberto I, Roma, Italy

##### **Correspondence:** Mario De Curtis (mario.decurtis@uniroma1.it)

**Background**

in Italy in the last century there have been significant demographic changes.

The aim of the study is to analyse the changes of birth rates and neonatal and infant mortality in Italy.

**Materials and Methods**

The presented data was obtained from the most recent data from the Italian Statistics Bureau (ISTAT). Neonatal and infant mortality rates were calculated as the number of deaths occurring before 28 days of age and the first year of life respectively for every 1000 live births. Children were considered Italian citizens if at least one of their parents had Italian citizenship; they were considered immigrant residents living in Italy if neither parent had Italian citizenship.

**Results**

During 2018, 449,000 children were born. This was 128,000 less than that of 2008, at the beginning of economic crisis, and less than half of those born in the 60s.

The mean age of women at childbirth was 32 years and 9% of births in Italy occur in women over 40 years old. The number of those born with medically assisted procreation techniques has increased and in 2016 they were 13,582, or about 3% of total births. In 2018, foreign parents, who represent only 8.7% of the all population, contributed significantly to the birth rate with 67,000 births (14.9% of all births).

In 2016 mean neonatal and infant mortality was 2 and 2.8 per thousand live births respectively but varied across the different regions of Italy. Children born in the southern regions have a 36% higher risk of mortality compared to those born in the northern regions. Infants born to immigrant parents have an infant mortality rate that was 56% higher than those born to Italian parents.

**Discussion**

The decline in birth rates in Italy is linked to the reduction of the number of potential parents, due to the recent economic crisis and to the frequent postponing of the age of procreation by many working women. The possible interventions to improve the birth rate should include a series of fiscal policies in favor of families with children, interventions to help working parents and, above all, the availability - at low costs - of childcare services.

The main causes of neonatal and infant mortality are linked not only to economic and social factors, but also to organizational problems concerning the perinatal care.

**Conclusions**

A political and social project that puts children at the center of attention and good governance is indispensable, because children represent our future.

## A46 Prevention of chronic malnutrition in “special” patients: PEG and PEJ

### Paola De Angelis (paola.deangelis@opbg.net)

#### Endoscopy Unit, Digestive Diseases Unit Bambino Gesù Children Hospital, Rome

In order to prevent chronic malnutrition, artificial feeding plays a great role for some “special” patients. Enteral nutrition by percutaneous gastrostomy is recommended in patients with “normal” bowel function and long term artificial feeding requirement (encephalopathy, cerebral palsy, repeated aspiration pneumonia, etc). Percutaneous endoscopic gastrostomy (PEG) is easily and safety performed in deep sedation/general anaesthesia, with consequent early enteral nutrition and drug administration, and a related low incidence of immediate complications. The avoidance of nasogastric tube and its side effects and a good compliance of patients and caregivers and nurses are a real advantage of this technique. In anatomy difficulties a surgical gastrostomy should be performed. A percutaneous endoscopic gastrojejunostomy (PEJ) could be important for patients affected by chronic vomit. The style-guided J-tube through the pyloric ring is endoscopically passed and advanced it to the jejunum (jejunal feeding instead of gastric one), without sedation in endoscopic suite. A multidisciplinary approach of patients with enteral feding is necessary to share indications, timing and management of PEG/PEJ. GERD and nutritional intakes must be considered in patients’ follow up.

## A47 Health mobile for rehabilitation: what prospects?

### Matteo De Marchis, Beniamino Di Giacomodonato, Irene Piermarini, Paola Leone, Sergio Bella

#### ^1^Dep. of specialized Pediatrics , Bambino Gesù Children’s Hospital Rome, Rome, Italy

##### **Correspondence:** Matteo De Marchis (matteo.demarchis@tiscali.it )

**Background**

Recently, the category of health-related apps has seen significant growth. An increasing number of health care organizations are using Mobile Health (mHealth) apps with an understanding of the potential benefit for monitoring and treating diseases and chronic conditions and facilitating diagnoses. We analized Cystic fibrosis (CF) that represents a multi-organ genetic disorder, but its clinical outcome involves mostly the respiratory system. Respiratory physiotherapy is a fondamental tool for the cure of cystic fibrosis. Viscous mucus secretions obstruct airways, leading to recurrent and often subsequent chronic infections. The course of the disease is marked by periodic exacerbations with decline in pulmonary function. Adherence is generally poor and has been estimated at 50% or less for pulmonary medications. Telehealth could be useful in improving the adherence to PR (pulmonary rehabilitation), as a continuous information exchange between rehabilitation team/caregivers and patient/caregivers. The Telehealth system allows communicating in real-time health issues of the patient to the rehabilitation team and improves the collection of patient’s data from a retrospective point of view of the majority of chronic respiratory diseases.

**Material and Methods**

We realized a survey to evaluate the possible use and the utility of a future digital application for respiratory rehabilitation in patients followed by Telemedicine-service in a new future way to practise rehabilitation. 24 CF patients (age 27,04±8,13) are enrolled and then their parents (age 40,45±7,26); we asked them to answer ten questions about for example the use of mobile-phone, the internet connection, which app they usually use, and also if they want to receive a communication about respiratory rehabilitation. So, we thought a practice application with the aim to manage the current personal PR program at home, enabling to administer aerosol therapies protocol, bronchial clearance techniques, physical exercises, and have access to personal documents and certifications.

**Results**

All adult patients and their parents (100%) expressed an excellent opinion about a possible way of digital application for physiotherapy, and they want to use it in their daily life.

**Conclusions**

This future app for respiratory rehabilitation will be a good tool, specially to improve adherence in medical treatment in patients with Cystic Fibrosis.

## A48 Mechanisms of fever and inflammation

### Arianna De Matteis (ari.dematteis@gmail.com)

#### Department of Translational Medical Science, Section of Pediatrics, University of Naples Federico II, Naples, Italy

**Background** Inflammation is a physiologic process aiming to defend the integrity of organisms against dangerous insults. Fever is a cardinal response of inflammation which depends on synergism between immunological and neurological activity [1,2].

**Materials and methods** Literature review has been performed about mechanisms of fever and inflammation.

**Results** Endogenous pyrogens (TNF, IL-1 and IL-6) are the bridge leading inflammatory and neurological response. They act both in peripheric tissue, causing the activation of innate and adaptive immune response, and in central nervous system promoting production of PGE2 (prostaglandin E2) by COX-2. Cytokine synergy is critical for fever, but not the all molecules involved in the inflammatory response are responsible of it. Fever is the main symptom of autoinflammatory disorders, in which uncontrolled activation of innate immune system cause an over-production of inflammatory mediators.

**Conclusions** Fever is an ancestral defence response, which mechanism includes the activation of both immune and neurological system. Peripheral and central activity of cytokines is essential for organising homeostasis of fever and the alteration of immunological functionality is responsible for autoinflammatory disorders.

**Acknowledgements** I wish to acknowledge the help provided by Dr. Fabrizio De Benedetti and Dr. Claudia Bracaglia.

**References**

1. Evans SS, Repasky EA, Fisher DT. Fever and the thermal regulation of immunity: the immune system feels the heat. Nat Rev Immunol. 2015;15:335-49.

2. Gül A. Dynamics of Inflammatory Response in Autoinflammatory Disorders: Autonomous and Hyperinflammatory States. Front Immunol. 2018 ;9:2422.

## A49 Early treatment of DDH significantly influences the acetabulum’s growth in newborns

### Maurizio De Pellegrin, Lorenzo Marcucci, Désirée Moharamzadeh, Dario Fracassetti

#### Pediatric Orthopedic Department IRCCS San Raffaele Hospital, Milan, Italy

##### **Correspondence:** Maurizio De Pellegrin (depellegrin.maurizio@hsr.it)

**Background.** Early treatment of developmental dysplasia of the hip (DDH) is more effective if started at an early stage [1-4]. Ramsey reported in 1976 [5] that if treated by the 1^st^ month of life, the treatment duration lasted in average 3.6 months; if between the 1^st^ and 3^rd^ month, it increased to 7 months; if between the 3^rd^ and 6^th^ month, it increased to 9.3 months. Therefore, the importance of anticipating DDH diagnosis, possibly by the 6th week of life, is obvious [6]. Early DDH diagnosis not only facilitates early treatment, but it also reduces the likelihood of avascular necrosis of the femoral head and early osteoarthritis of the hip and leads to better results [7,8]. Aim of this study was to evaluate the effectiveness of early treatment in severe DDH in newborns.

**Materials and methods.** For this study 72 neonates (66 females, 6 males) with 93 type III hips according to Graf’s ultrasonographic classification were examined. Out of 72 patients 21 (0 males, 21 females) had bilateral presentation, 21 (3 males, 18 females) had monolateral presentation with the left side affected and 30 (3 males, 27 females) with the right side affected. Mean age at diagnosis was 35 days (range 1-202). The treatment was always performed with the hips in the “squatting” position (100-110° flexion, 50° abduction). The acetabulum’s growth was ultrasonographycally monitored and the alpha-angle assessed by two subsequent evaluations with an average follow up of 109 days (range 39-347). The sample was then divided into three groups based upon patient’s age at diagnosis and treatment beginning: Group 1 (age < 11 days), Group 2 (age ≥ 11days <42 days), Group 3 (age ≥ 42 days).

**Results** The statistical analysis showed a strong dependency (*p*<0,001) between the alpha-angle gain (acetabular growth) and the time of diagnosis and found no statistically significant correlation between sex (p-value 0,81), affected side (p-value 0,72) and treatment length (p-value 0,864). At follow up in Group 1 91,31 % of the severe dysplastic hips showed normal values of the alpha angle (> 60°) while in Group 2 and in Group 3 62.5 % and 48.7 % respectively (Figure 1).

**Fig. 1 (abstract A49).**
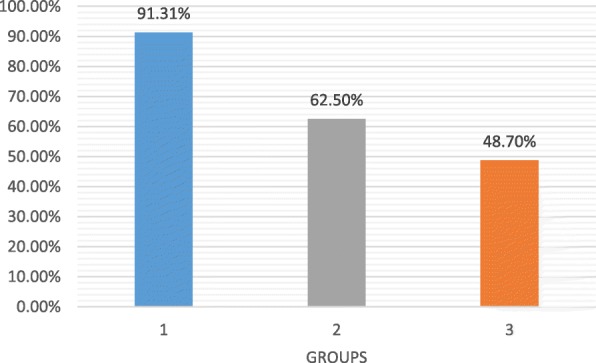
The histograms show the percentage of hips affected by severe DDH reaching a normal alpha-angle (>60°) at the second Ultrasound-FU in the three groups of the sample. (Sample based upon patient’s age at diagnosis and treatment beginning: Group 1 age < 11 days, Group 2 age ≥ 11days <42 days, Group 3 age ≥ 42 days)

**Conclusions** The results highlight the mandatory need for an early treatment of severe hip dysplasia, which, if not started shortly after birth (4-6 weeks) is considerably less effective and has less predictable results. The need of a DDH screening for early diagnosis has been confirmed [9,10].

**References**

1. Catterall A. The early diagnosis of congenital dislocation of the hip (editorial). J Bone Joint Surg Br. 1994;76:515-6.

2. American Academy of Pediatrics. La displasia evolutiva dell’anca: linee guida per la diagnosi precoce. Pediatr. 2000;12: 351-362.

3. De Pellegrin M, Tessari L. L’ecografia dell’anca infantile. Significato e ruolo nella diagnosi precoce di displasia congenita. Medico e Bambino. 1993;1:25-29.

4. De Pellegrin M, Moharamzadeh D, Fraschini G. Early diagnosis and treatment of DDH: a sonographic approach. Hip Int. 2007;17 Suppl 5:S15-21.

5. Ramsey PL. Congenital hip dislocation: before and after walking age. Postgrad Med. 1976;60:114-120.

6. De Pellegrin M, Boero S, Origo C, Farsetti P. La displasia congenita dell’anca (DCA). Terminologia, diagnosi precoce, screening, raccomandazioni. Giornale Italiano di Ortopedia e Traumatologia. 2019;45:15-20.

**7.** Senaran H, Bowen JR, Harcke HT. Avascular necrosis rate in early reduction after failed Pavlik harness treatment of developmental dysplasia of the hip. J Pediatr Orthop. 2007;27:192-7.

8. Kotlarsky P, Haber R, Bialik V & Eidelman M. Developmental dysplasia of the hip: What has changed in the last 20 years? World Journal of Orthopedics. 2015;6:886-901.

9. Mahan ST, Katz JN, Kim YJ. To screen or not to screen? A decision analysis of the utility of screening for developmental dysplasia of the hip. J Bone Joint Surg Am. 2009;91:1705-19.

10. Chiara A, De Pellegrin M. Developmental dysplasia of the hip: to screen or not to screen with ultrasound. Early Hum Dev. 2013;89S4:S102-S103.

## A50 The Emilia-Romagna experience with ProBA project (Progetto Bambini e Antibiotici): a multilevel intervention to reduce inappropriate antibiotic prescription in pediatric care

### Simona Di Mario^1^, Carlo Gagliotti^2^, Rossella Buttazzi^2^, Luca Cisbani^3^, Chiara Di Girolamo^4^, Antonio Brambilla^5^, Maria Luisa Moro^2^

#### ^1^ Regional Health Authority of Emilia-Romagna, Primary Care Service and SaPeRiDoc- Centro di Documentazione sulla Salute Perinatale e Riproduttiva, Bologna, Italy; ^2^ Regional Health and Social Agency of Emilia-Romagna, Bologna, Italy; ^3^ Information Technology Service, Regional Health Authority of Emilia-Romagna, Bologna, Italy; ^4^ Department of Medical and Surgical Sciences, Alma Mater Studiorum - University of Bologna, Bologna, Italy; ^5^ Formerly Regional Health Authority of Emilia-Romagna, Bologna, Italy

##### **Correspondence:** Simona Di Mario (simona.dimario@regione.emilia-romagna.it)

**Background**

To describe the components and the achievements of the ProBA project (Progetto Bambini e Antibiotici), a long-lasting multilevel intervention aimed at promoting appropriate use of antibiotics in Emilia-Romagna Region (Italy).

**Method**

The project started in 2003 with a KAP survey involving pediatricians and parents on reasons for antibiotic prescription. Guidelines development on acute otitis media and sore throat followed in 2007, a monitoring system on antibiotic prescriptions with annual report was implemented, and information for parents were provided in the first phase of the project. Starting from 2015, a second phase of ProBA project was implemented based on previous experience: guidelines were updated, evidence was disseminated, new tools to monitor results and to provide feedback were developed. Moreover, an annual public information campaign was launched and progressively enriched with new products. Regional commitment was ensured through clear identification of objectives and pay for performance incentives were provided to health professionals. A quantitative and qualitative assessment of antibiotic use in children 0-13 years was performed. According to the European Commission recommendation, assessment was based on the estimation of two principal indicators: the total antibiotic prescription rate and the ratio of amoxicillin versus amoxicillin-clavulanic acid. Throughout the project, papers both in Italian and in English language were published to share findings and experiences.

**Results**

A substantial decrease of antibiotic prescription rates was registered in the time period 2005-2017 (from 1307 to 777 prescriptions per 1000 children), along with a relative growth of the use of amoxicillin (the ratio of amoxicillin to amoxicillin-clavulanic acid increased from 0.6 to 1.3 in the same population). The Italian Medicine Agency AIFA recently issued a national report on antibiotic use that applies the same indicators used in ProBA. Figures at national level were: total antibiotic prescription rate 1047 prescriptions per 1000 children (quite higher than the one in Emilia-Romagna), and ratio of amoxicillin to amoxicillin-clavulanic acid 0.3 (much lower than the one in our Region).

**Conclusions**

The ProBA project is associated with a containment of antibiotic over-prescription for pediatric care and a relative increase of narrow spectrum antibiotics use. A future development of the project could be the establishment of a collaborative network with other Italian regions to define a common set of indicators, to compare attitudes and practices, to share products and tools developed within ProBA, and to outline joint objectives of improvement.

## A51 New conception scenarios and maternal dimension

### Magda Di Renzo, Federico Bianchi di Castelbianco

#### Istituto di Ortofonologia, Roma, Italy

##### **Correspondence:** Magda Di Renzo (m.direnzo@ortofonologia.it)

The advancement of medically assisted procreation technology has generated radical changes in issues relating to maternal dimension and in the psychic structure associated with the child birth, but it was not accompained by necessary ethical and psychic considerations.

The child of the new conception scenarios seems to be scheduled rather then desired, even regardless of the fact that the implementation of the medically assisted procreation tecnique, sometimes creating negative effects on future mothers and babies, quiets “the manifestation of most carnal symbols detachs eroticism from sexuality and breaks the symbolic chain sexual desire-desire for a child” [1], making it subjectively sterilizing. The coercion of technique does not allow to experience the fascination of intimacy and it impacts the course of pregnancy. The choice of “a baby not matter what”, namely cost what it may, preclude the relationality and it does not permit the slow process of mentalizing a child, that can take place only after the process of infertility grief. The child of the new conception scenarios is not a child desired for him/herself but “above all it represents a child having a duty to be conforming to the desire of those who gave him/her birth” [2].

The speed of these changes did not allow for sufficient meditation and depressive episode or significant couple conflicts which may lead to separation often occur, once the child was born (that is once reached thei narcissistic desire). Mathers and fathers who did not take part to the generation of the child, who did not take part to the conception scene (for egg and sperm donation), are often overwhelmed by alienation and strangeness and this may negatively affects the child’s development or future. Or, the child may face with aging parents (thanks to technology you may have a baby even at age 60) who are unable to guarantee an appropriate physical and psychological avalaibility in his/her life and they make him/her ashamed of having granparents rather then parents. There is also an issue about foetal reduction (that is prohibited but possible for medical reason) that may lead guilt over eliminating one of the many coveted children.

**References**

1. Chatel M M, Malaise dans la procrèation, Albin Michel. Paris; 1993.
2. Bayle B, L’embryon sur le divan. Psycopathologie de la conception humain, Masson. Paris; 2003.

## A52 Primary Ciliary Dyskinesia: a rare cause for Bronchiectasis in Childhood

### Maria E. Di Cicco ^1,2^, Diego Peroni ^1,2^, Massimo Pifferi ^2^

#### ^1^ Department of Clinical and Experimental Medicine, University of Pisa, Pisa, Italy; ^2^ Pediatrics Unit, Pisa University Hospital, Pisa, Italy

##### **Correspondence:** Maria E. Di Cicco (maria.dicicco@unipi.it)

Primary Ciliary Dyskinesia (PCD) is a rare congenital, clinically and genetically heterogeneous disease caused by abnormal structure and/or function of respiratory cilia, with impaired mucociliary transport leading to recurrent respiratory infections and progressive loss of lung function. PCD diagnosis is not easy, since signs and symptoms of this condition are nonspecific and may vary according to the age of the patients. Moreover, diagnostic tests are complex, time consuming and expensive [1]. However, some pointers of the disease, such as laterality defects, daily wet cough and persistent rhinorrhea, may be noticed in the perinatal period and should suggest to refer the patient to the specialist or at the reference center for PCD, in order to perform the diagnostic tests and start the related treatments as soon as possible [1]. In PCD, the recurrence of respiratory infections causes a continuous and exaggerated neutrophilic response in the bronchial mucosa, with consequent release of tissue-damaging enzymes that, in time, lead to progressive bronchial wall damage and dilatation, known as bronchiectasis. In bronchiectasis mucus is even more stuck, which make infections and inflammation more and more frequent and severe, causing worsening of the lesions and symptoms, with onset of sputum production and eventually bronchorrea. As a consequence, bronchiectasis severity correlates with worse pulmonary function [2]. Bronchiectasis is a consistent finding in adult PCD patients, but often appears in childhood [3] by 8 years of age in 50% of cases [4]. Bronchiectasis distribution in PCD has been shown to have a basal predilection: the most involved pulmonary lobes are the middle lobe, the lingula and the inferior lobes, while, unlike cystic fibrosis, upper lobes involvement can be found in most severe cases and later disease stage. [4]. In children, bronchiectasis involves firstly the middle lobe, since the middle lobe bronchus is the longest, narrowest and most horizontal of the lobar bronchi, facilitating mucus stagnation and plugging. High Resolution Chest Tomography is still the gold standard test to diagnose bronchiectasis and assess its extent and severity. Recently, some studies have shown that also chest Magnetic Resonance Imaging is an accurate and reliable method to evaluate bronchiectasis in PCD patients [5]. Treatment of bronchiectasis is mainly based on respiratory physiotherapy and airway clearance techniques, early administration of antibiotics on the occasion of respiratory infections and eradication or chronic treatment of Pseudomonas aeruginosa infection when present [6].

**References**

1. Shapiro AJ, Davis SD, Polineni D, Manion M, Rosenfeld M, Dell SD, et al. American Thoracic Society Assembly on Pediatrics. Diagnosis of Primary Ciliary Dyskinesia. An Official American Thoracic Society Clinical Practice Guideline. Am J Respir Crit Care Med. 2018;197:e24-e39.

2. Pifferi M, Bush A, Pioggia G, Caramella D, Tartarisco G, Di Cicco M, et al. Evaluation of pulmonary disease using static lung volumes in primary ciliary dyskinesia. Thorax. 2012;67:993-9.

3. Hosie PH, Fitzgerald DA, Jaffe A, Birman CS, Rutland J, Morgan LC. Presentation of primary ciliary dyskinesia in children: 30 years’ experience. J Paediatr Child Health. 2015;51:722-6.

4. Kennedy MP, Noone PG, Leigh MW, Zariwala MA, Minnix SL, Knowles MR, et al. High-resolution CT of patients with primary ciliary dyskinesia. Am J Roentgenol. 2007;188:1232-8.

5. Montella S, Santamaria F, Salvatore M, Maglione M, Iacotucci P, De Santi MM, et al. Lung disease assessment in primary ciliary dyskinesia: a comparison between chest high-field magnetic resonance imaging and high-resolution computed tomography findings. Ital J Pediatr. 2009;35:24.

6. Polineni D, Davis SD, Dell SD. Treatment recommendations in Primary Ciliary Dyskinesia Paediatr Respir Rev. 2016;18:39-45.

## A53 Lung microbiome and asthma in children

### Maria E Di Cicco ^1,2^, Mauro Pistello ^3^, Pasquale Comberiati ^2^, Massimo Pifferi ^2^, Diego Peroni ^1,2^

#### ^1^ Department of Clinical and Experimental Medicine, University of Pisa, Pisa, Italy; ^2^ Pediatrics Unit, Pisa University Hospital, Pisa, Italy; ^3^ Retrovirus Center and Virology Section, Department of Translational Research and New Technologies in Medicine and Surgery, University of Pisa, Pisa, Italy

##### **Correspondence:** Maria E. Di Cicco (maria.dicicco@unipi.it)

Healthy lungs have been historically considered to be sterile based on the results of standard microbiological culture techniques. However, starting from the 2010s, next generation sequencing has been used on lower airways samples allowing the identification of low-density microbial communities in a culture-independent way [1]: the lung microbiome composition is now well known and described as to be characterized by the prevalence of bacteria belonging to phyla *Firmicutes* and *Bacteroidetes* (mostly Prevotella and Veilonella spp), reflecting the composition of the microbiome of the oropharynx, since bacteria arrive to the lungs mostly through micro-aspiration of secretions from the naso-oropharynx [2]. In the healthy subjects bacteria are rapidly eliminated from the lower airways, in a continuous and delicate balancing of immigration and elimination, which can be acutely altered during respiratory infections or be modified in chronic respiratory diseases, especially in advanced stages [3]. Asthma is the most common chronic disease in childhood: its pathogenesis is multifactorial and includes environmental factors interacting with susceptibility genes during pregnancy and in the first years of life. In asthma, as in other respiratory diseases, the lung microbiome composition changes: as a matter of fact, many studies have demonstrated that both in children and in adult asthmatics the lung microbiome is characterized by the prevalence of the phyla *Proteobacteria* (mostly Haemophilus, Moraxella, and Neisseria spp) and reduced biodiversity [4]. However, it is not clear whether this change is secondary to the underlying disease or plays a primary role in its development. Considering what is already known about the role of early atopy onset and early viral infections in the development of asthma, with RSV and rhinovirus as major risk factors, a primary role of the microbiome seems likely [5]. For example, in infancy, respiratory viral infections can induce the emergence of Proteobacteria, which are also known to be linked to a higher risk of asthma at 5 years in children born to asthmatic mothers. Moreover, the phylum Proteobacteria is linked to severity of asthma and bronchial hyperreactivity. The lung microbiome may also interact with the immune system, modulating inflammation. Antibiotics may alter the lung microbiome and possibly interfere with all these processes: as a consequence, they should be prescribed exclusively when necessary, especially in the first years of life. The potential role of probiotics and prebiotics on lung microbiome has not been yet evaluated.

**References**

1. Hilty M, Burke C, Pedro H, Cardenas P, Bush A, Bossley C, et al. Disordered microbial communities in asthmatic airways. PLoS One. 2010; 5: e8578.

2. Dickson RP, Erb-Downward JR, Freeman CM, McCloskey L, Beck JM, Huffnagle GB, et al. Spatial variation in the healthy human lung microbiome and the adapted island model of lung biogeography. Ann Am Thorac Soc. 2015;12:821-30.

3. Dickson RP, Martinez FJ, Huffnagle GB. The role of the microbiome in exacerbations of chronic lung diseases. Lancet. 2014;384:691-702.

4. Di Cicco M, Pistello M, Jacinto T, Ragazzo V, Piras M, Freer G, et al. Does lung microbiome play a causal or casual role in asthma? Pediatr Pulmonol. 2018;53:1340-5.

5. Sullivan A, Hunt E, MacSharry J, Murphy DM. The microbiome and the pathophysiology of asthma. Respir Res. 2016;17:163.

## A54 Interventions for improving vaccine literacy in Neonatal Intensive Care Unit. A single centre experience and future perspective

### Antonio Di Mauro^1^, Maria Elisabetta Baldassarre^1^, Federico Schettini^1^, Manuela Capozza^1^, Raffaella Panza^1^, Federica Di Mauro^2^, Francesco Paolo Bianchi^2^, Silvio Tafuri^2^, Nicola Laforgia^1^

#### ^1^ Neonatology and Neonatal Intensive Care Unit Section, Department of Biomedical Science and Human Oncology, “Aldo Moro” University of Bari, P.za Giulio Cesare 11, 70124 Bari, Italy; ^2^ Hygiene and Preventive Medicine Section, Department of Biomedical Science and Human Oncology, “Aldo Moro” University of Bari

##### **Correspondence:** Antonio Di Mauro (antonio.dimauro@uniba.it)

**Background**

Despite preterm infants are at high risk for infectious diseases and World Health Organization recommends that they receive immunization according to routine schedules for at term neonates, completeness and timeliness of vaccinations in this special population is worldwide still lower compared to general population.

**Materials and methods**

In our Neonatal Intensive Care Unit a specific program for parents of preterm infants has been implemented to improve their vaccine literacy, by in-hospital vaccine administration, outpatient follow-up vaccination counselling, vaccine-related information through social media. We have evaluated the efficacy of this program, by the analysis of coverage and timeliness of first two doses of vaccine (both Hexavalent and PCV13) in our patients (birth cohort 2016/17), comparing their data with data from general population of the same birth cohort extracted from the 2019 Apulian Regional Vaccination Register (GIAVA).

**Results**

We studied 170 preterm infants with average gestational age and weight of, respectively, 30.1±2.9 weeks (range: 23-33) and 1,456.0±504.9 grams (range: 400-2,590).

Table 1 and 2 show coverage and timeless of vaccination.

Coverage resulted similar to general population. This result, confirming our previous study [1], highlights how our interventions are consistent with the purpose of overcoming vaccination hesitancy, especially in parents of infants who have experienced health problems. Furthermore, our results confirms the efficacy of the active vaccinations administration during hospitalization as a powerful tool to improve coverage.

In preterm infants, the average age of vaccinations is similar to data previously published [1] and significant delayed compare to the recommended timeline (Table 2).

The linear regression analysis shows no association between time delay with birth-weight and/or gestational age (p>0.05).

**Conclusions**

Our easy, inexpensive and highly reproducible interventions seem to increase vaccination coverage in preterm infants. Timeliness remains still inadequate and carries significant worry in this high-risk population for preventable infectious disease-related complications.

The “medical” use of web 2.0-based social networks, because of their widespread distribution, could be a very significant tool, if adequately monitored, to positively influence parents’ decision making about vaccination and counteract vaccine hesitancy.

**Table 1 (abstract A54).** Vaccine coverage (VC) in preterm newborns and general population. Birth cohort 2016-2017

Vaccine type	VC	Preterm	General population	z	p
	n	% (95%CI)	%		
DTaP-IPV-HBV-Hib 1 dose	169	99.4 (96.7-99.9)	99.0	0.5	0.601
DTaP-IPV-HBV-Hib 2 dose	164	96.5 (92.5-98.7)	94.8	1.0	0.319
PCV 1 dose	167	98.2 (94.9-99.6)	98.4	0.2	0.836
PCV 2 dose	161	94.7 (90.2-97.8)	94.1	0.3	0.740

**Table 2 (abstract A54).** Average age of vaccine administration (day: mean±SD) in preterm and general population. Birth cohort 2016-2017

Vaccine type	preterm	general population	t	p
DTaP-IPV-HBV-Hib 1 dose	119.3±81.9	61.0	9.3	0.000
DTaP-IPV-HBV-Hib 2 dose	214.6±105.0	121.0	11.6	0.000
PCV 1 dose	119.0±66.8	61.0	11.3	0.000
PCV 2 dose	209.8±81.8	121.0	14.2	0.000

**Reference**

1. Laforgia N, Di Mauro A, Bianchi FP, et al. Are pre-terms born timely and right immunized? Results of an Italian cohort study. Hum Vaccin Immunother. 2018;14:1398–1402.

## A55 New therapeutic options in children with chronic intestinal pseudo-obstruction

### Giovanni Di Nardo (giovanni.dinardo@icloud.com)

#### Chair of Pediatrics, NESMOS Department, School of Medicine and Psychology, Sapienza University of Rome, Sant’Andrea University Hospital, Rome, Italy

Chronic intestinal pseudo-obstruction (CIPO) is a rare and severe gastrointestinal motility disorder characterized by a severe impairment of gastrointestinal motility leading to intestinal obstruction symptoms in the absence of mechanical causes. Symptoms can be non-specific, and result in this condition being diagnosed incorrectly or too late with consequences for morbidity and even mortality. There is no single diagnostic test or pathognomonic finding of CIPO, thus the diagnosis is usually clinical and diagnostic work is usually aimed to rule out mechanical obstruction and to identify any underlying diseases. Treatment is challenging and requires a multidisciplinary effort with participation of appropriately experienced gastroenterologists, specialized dieticians, surgeons, psychologists, and other subspecialists based on the presence of comorbidities. Medical therapies are mainly aimed to avoid complications such as sepsis or intestinal bacterial overgrowth and restore intestinal propulsion. So far, only few prokinetics have proven some efficacy in restoring intestinal propulsion, thus nutritional support, fluid/electrolyte replacement, and antibiotics are the mainstay of treatment. Decompression of distended intestinal segments via intermittent nasogastric tube or endoscopy is an important therapeutic target. In some cases a venting enterostomy, typically placed endoscopically can be helpful. Surgery may be indicated as a tool to collect gut biopsy specimens for histopathology, in emergency situations (massive bowel distension and perforation/ischemia) and to perform palliative procedures such as feeding/venting gastro-jejunostomies. Intestinal transplantation should be considered in patients with CIPO presenting with life threatening TPN-associated complications. Although there is little evidence emerging pilot studies have explored the use of faecal microbiota transplantation in CIPO with reports of improved symptoms, indirect markers of intestinal obstruction and small intestinal bacterial overgrowth.

## A56 The study of the natural history of Warts, Hypogammaglobulinemia, Infections, Myelokathexis Syndrome in a large cohort of 21 patients

### Laura Dotta^1^, Lucia Dora Notarangelo^2^, Jessica Galli^3^, Lorenzo Pinelli^4^, Serena Micheletti^3^, Giovanni Palumbo^4^, Elena Borroni^5^, Daniele Moratto^6^, Rajesh Kumar^6^, Fulvio Porta^2^, Annarosa Soresina^1^, Vassilios Lougaris^7^, Alessandro Plebani^7^, C. I. Edvard Smith^8^, Anna-Carin Norlin^9^, Andrea Cecilia Gòmez Raccio^10^, Eva Bubanska^11^, Patrizia Bertolini^12^, Giovanni Amendola^13^, Marcella Visentini^14^, Massimo Fiorilli^14^, Aldo Venuti^15^, Elisa Fazzi^3^, Massimo Locati^5^, Raffaele Badolato^7^

#### ^1^Department of Pediatrics & Institute of Molecular Medicine “Angelo Nocivelli”, Asst Spedali Civili of Brescia, Brescia, Italy; ^2^Pediatric Hematology Oncology Unit, Asst Spedali Civili of Brescia, Brescia, Italy; ^3^Child Neurology and Psychiatry Unit, ASST Spedali Civili of Brescia, Brescia, Italy; ^4^Neuroradiology Unit, Section of Pediatric Neuroradiology, ASST Spedali Civili of Brescia, Brescia, Italy; ^5^Humanitas Clinical and Research Center, Milano, Italy; ^6^Institute of Molecular Medicine “Angelo Nocivelli”, University of Brescia & Asst Spedali Civili of Brescia, Brescia, Italy; ^7^Department of Pediatrics & Department of Clinical and Experimental Sciences, Institute of Molecular Medicine “Angelo Nocivelli”, University of Brescia & Asst Spedali Civili of Brescia, Brescia, Italy; ^8^Clinical Research Center, Department of Laboratory Medicine, Karolinska Institutet, Karolinska University Hospital Huddinge, Huddinge, Sweden; ^9^Division of Therapeutic Immunology, Department of Laboratory Medicine, Karolinska Institutet & Department of Clinical Immunology and Transfusion Medicine, Karolinska University Laboratory, Karolinska University Hospital, Stockholm, Sweden; ^10^Immunology Unit, Children's Hospital Ricardo Gutierrez, Buenos Aires, Argentine; ^11^Department of Pediatric Oncology and Hematology, University Children's Hospital, Banska Bystrica, Slovakia; ^12^Pediatric Hematology Oncology Unit, Azienda Ospedaliero Universitaria of Parma, Parma, Italy; ^13^Department of Pediatrics, Nocera Inferiore Hospital, Salerno, Italy; ^14^HPV-Unit, UOSD Tumor Immunology and Immunotherapy, IRCCS Regina Elena National Cancer Institute, Roma, Italy; ^15^Department of Clinical Medicine, Sapienza University of Rome, Roma, Italy

##### **Correspondence:** Laura Dotta (lauradotta@icloud.com)

**Background**

The WHIM syndrome is a primary immunodeficiency caused by autosomal dominant mutations in the C-X-C chemokine receptor type 4 (CXCR4) [1]. Reported frameshifts, nonsense or deletion mutations result in the truncation of 10 to 19 amino acids from the C-terminus of the cytoplasmic domain and account for the *gain-of-function* activity [2]. The acronym refers to warts, hypogammaglobulinemia, infections, and myelokathexis, the abnormal retention of mature neutrophils in the bone marrow. Neutropenia associates with lymphopenia and monocytopenia leading to panleukopenia, the recurrence of pulmonary infections may evolve to chronic lung disease by early adulthood, and the specific susceptibility to chronic infection with Human Papilloma Virus (HPV) may expose patients to risk of malignancy [3-5]. Stating the role of CXCR4 since the embryonic brain and heart system development, other than hematopoiesis, the syndrome may feature congenital heart disease and neurological abnormalities [6-7]. Conventional treatments include Granulocyte-Colony Stimulating Factor, immunoglobulin therapy and/or antibiotic prophylaxis; the promising results reported with the CXCR4 antagonist plerixafor [8] paved the way to the future treatment of this condition with CXCR4-based therapies.

**Material and methods**

We investigated molecular, biological, clinical, radiological, and immunological features of 21 patients who referred to the Department of Brescia. Genetic testing was performed by Sanger sequencing, functional tests were performed by flow-cytometry, and data were collected from medical records or by a case report form.

**Results**

Patients have a median age of 22 years, with 1.6±3.8 at the time of clinical onset, but 9±10 at the time of molecular diagnosis. GOF mutations were identified in 18/21, while three patients presented a novel frameshift mutation with a not-GOF biological pattern (the L317fsX3). Warts occurred in 62% of patients, bacterial infections in 90%, the Tetralogy of Fallot in 19%; 4 out of 6 investigated patients had abnormal cerebellar morphology. All patients had severe neutropenia at onset (150±105 cells/mmc), monocytopenia was reported in 13/21 (61%), lymphopenia in 16/21 (76%), always associated with B lymphopenia. Hypogammaglobulinemia was present in 46%. No genotype- phenotype seemed significant.

**Conclusions**

WHIM syndrome may exhibit heterogeneous molecular and clinical phenotypes, and it should always be suspected in cases of severe congenital neutropenia. The diagnosis is confirmed by molecular testing, but functional tests are required to characterize the mutation. Novel clinical trials will explore CXCR4-targeted therapies to reverse panleukopenia and control HPV manifestations. Meanwhile, HPV vaccination should be recommended in all affected patients and immunoglobulin treatment suggested since early childhood to prevent the development of chronic lung disease.

**References**

1. Hernandez PA, Gorlin RJ, Lukens JN, Taniuchi S, Bohinjec J, Francois F, et al. Mutations in the chemokine receptor gene CXCR4 are associated with WHIM syndrome, a combined immunodeficiency disease. Nat Genet. 2003;34:70–4.
2. Gulino AV, Moratto D, Sozzani S, Cavadini P, Otero K, Tassone L, et al. Altered leukocyte response to CXCL12 in patients with warts hypogammaglobulinemia, infections, myelokathexis (WHIM) syndrome. Blood. 2004;104:444–52.
3. Dotta L, Tassone L, Badolato R. Clinical and genetic features of Warts, Hypogammaglobulinemia, Infections and Myelokathexis (WHIM) syndrome. Curr Mol Med. 2011;11:317–25.
4. Tassone L, Notarangelo LD, Bonomi V, Savoldi G, Sensi A, Soresina A, et al. Clinical and genetic diagnosis of warts, hypogammaglobulinemia, infections, and myelokathexis syndrome in 10 patients. J Allergy Clin Immunol. 2009;123:1170–3.
5. Dotta L, Notarangelo LD, Moratto D, Kumar R, Porta F, Badolato R. Long term outcome of WHIM syndrome in 18 patients: high risk of lung disease and HPV-related malignancies. J Allergy Clin Immunol Pract. 2019;7:1568-1577.
6. Badolato R, Dotta L, Tassone L, Amendola G, Porta F, Locatelli F, et al. Tetralogy of Fallot is an Uncommon Manifestation of Warts, Hypogammaglobulinemia, Infections, and Myelokathexis Syndrome. J Pediatr. 2012;10–2.
7. Galli J, Pinelli L, Micheletti S, Palumbo G, Notarangelo LD, Lougaris V, et al. Cerebellar involvement in warts Hypogammaglobulinemia immunodeficiency myelokathexis patients: neuroimaging and clinical findings. Orphanet J Rare Dis. 2019;14:61.
8. McDermott DH, Liu Q, Velez D, Lopez L, Anaya-O’Brien S, Ulrick J, et al. A phase 1 clinical trial of long-term, low-dose treatment of WHIM syndrome with the CXCR4 antagonist plerixafor. Blood. 2014;123:2308–16.

## A57 Formulas for NE and Ne for OS: selection criteria and nutritional characteristics

### Mirella Domenica Elia (domenica.elia@opbg.net)

#### U.O. Nutrizione Artificiale, Ospedale Pediatrico Bambino Gesù, Roma, Italy

The choice of enteral nutrition for infants, children and adolescents must be founded on a careful evaluation of the caloric, nutrient and electrolyte needs, and based on the following criteria: patient’s age and type of pathology, presence of food allergy or intolerance and mode or route of administration (through SNG or PEG or PEJ) [1,2,3]. Standard enteral formulas contain macro- and micronutrient needs; therapeutic enteral formulas contain also specific pharmaconutrients that may attenuate hyperinflammatory responses, enhance the immune responses to infection, or improve gastrointestinal tolerance. Polymeric formulas are useful in patients with integrity of the gastrointestinal tract. The use of semi-elemental formulas is indicated in children with compromised GI function and in case of post-pyloric enteral administration.

Elementary formulas are indicated only in cases of severe allergy, not responding to extensive hydrolyzates formulas. Clinicians must carefully consider different use of specialty formulas, paying close attention to the quality, patient population, clinical end points, cost and/or facility of administration for patients.

**References**

1. Becker P, Carney LN, Corkins MR, Monczka J, Smith E, Smith SE, et al. Consensus Statement of the Academy of Nutrition and Dietetics- American Society for Parenteral and Enteral Nutrition: indicators recommended for the identification and documentation of pediatric malnutrition (undernutrition). Nutr Clin Pract. 2015;30:147-61.

2. Brown B, Roehl K, Betz M. Enteral nutrition formula selection: current evidence and implications for practice. Nutr Clin Pract. 2015;20:72-85.

3. Hegazi RA, Wischmeyer PE. Clinical review: optimizing enteral nutrition for critically ill patients-a simple data-driven formula. Crit Care. 2011;15:234.

## A58 Pediatric nutrition: a few rules for an healthy growth

### Valentina Fabiano^1^, Gian Vincenzo Zuccotti^1^

#### ^1^Department of pediatrics, V. Buzzi Children’s Hospital, Università degli Studi di Milano, Milan, Italy

##### **Correspondence:** Valentina Fabiano (valentina.fabiano@unimi.it)

Chronic non communicable diseases (NCDs), such as those related to overweight and obesity, represent major health issues worldwide. It is commonly accepted that an early intervention in the pediatric age, or even earlier, during pregnancy, has a positive impact in reducing life course risk of NCDs [1]. Italian data in infants and children, aged between 6 and 36 months, show some alarming results. Infants and toddlers eat too much proteins, simple sugars, unhealthy fats and salt; on the contrary, their iron intake is below recommendation [2]. Some few, useful rules in pediatric nutrition are important for an healthy life. Breastfeeding, as recommended by World Health Organization (WHO), should be exclusive in the first 6 months of life and should be continued during the complementary feeding period, until 12 months, or even afterward. Complementary feeding should not be started before the completed fourth month of age. Energetically adequate foods should be introduced, paying attention to limit the protein intake and to favor the intake of iron-rich foods. High protein intakes in the early life has been associated with a later increased risk of overweight and obesity [3].

Intake of simple sugars, above all those from snacks and sugar-added beverages, should be limited or preferably avoided at all; it has been demonstrated that substituting sugar-sweetened beverages with water decreases body fatness development in the adolescence [4]. Similarly, polyunsaturated fatty acids (PUFAs) should be preferred in the diet of infants and children: literature data showed that quality of the ingested fats is more important that their quantity in a healthy diet [5]. Other favorable eating habits include the limited sodium intake and the regular breakfast consumption. Starting from childhood to decrease salt intake may prevent development of arterial hypertension in later life [6]. As for breakfast, it has been demonstrated that children who skip it have a lower diet quality, characterized by an increased caloric intake from consumption of unhealthy snacks [7]. Healthy eating habits start with good food choices, and reading product labels is the first important step toward awareness of the importance of a healthy nutrition as a prevention strategy for NCDs.

**References**

1. Godfrey KM, Gluckman PD, Hanson MA. Developmental origins of metabolic disease: life course and intergenerational perspectives. Trends Endocrinol Metab. 2010;21:199-205.
2. Zuccotti GV, Cassatella C, Morelli A, Cucugliato MC, Catinello G, del Balzo V, et al. Nutrient intake in Italian children and toddlers from north to south Italy: the Nutriontake 636 Study. Nutrients. 2014;6:3169-86.
3. Weber M, Grote V, Closa-Monasterolo R, Escribano J, Langhendries JP, Dain E, et al. European Childhood Obesity Trial Study Group. Lower protein content in infant formula reduces BMI and obesity risk at school age: follow-up of a randomized trial. Am J Clin Nutr. 2014;99:1041-1051.
4. Zheng M, Rangan A, Olsen NJ, Andersen LB, Wedderkopp N, Kristensen P, et al. Substituting sugar-sweetened beverages with water or milk is inversely associated with body fatness development from childhood to adolescence. Nutrition. 2015;31:38-44.
5. Agostoni C, Caroli M. Role of fats in the first two years of life as related to later development of NCDs. Nutr Metab Cardiovasc Dis. 2012;22:775-80.
6. Leyvraz M, Chatelan A, da Costa BR, Taffe P, Paradis G, Bovet P, et al. Sodium intake and blood pressure in children and adolescents: A systematic review and meta-analysis of experimental and observational studies. Int. J. Epidemiol. 2018;47:1796–1810.
7. Ramsay SA, Bloch TD, Marriage B, Shriver LH, Spees CK, Taylor CA. Skipping breakfast is associated with lower diet quality in young US children. *Eur J Clin Nutr.* 2018;72:548–56.

## A59 Neonatal sepsis: from a reductionist to an holistic approach design

### Vassilios Fanos, Michele Mussap

#### Department of Surgery, School of Medicine, University of Cagliari, Cagliari, Italy

##### **Correspondence:** Vassilios Fanos (vafanos@tiscali.it)

Updated reports on epidemiological data collected over the last decade clearly indicate a dramatic increase of sepsis and septic shock worldwide, from birth to ageing, leading to almost 6 million deaths per year [1]. Approximately 2,202 babies every 100,000 live births develop neonatal sepsis with a mortality rate ranging 11–19%; this corresponds to 3.0 million cases of neonatal sepsis annually [2]. In May 2017, the United World Health Assembly and the World Health Organization (WHO) adopted a resolution to improve, prevent, diagnose, and manage sepsis. Sepsis was recognized a global health priority, marking a quantum leap in the global fight against this disease. Thus, the early and accurate diagnosis, the clinical course and the long-term outcomes of neonatal sepsis remain notable laboratory medicine challenges [3]. Diagnostic microbiology stands at the epicenter of the diagnostic tests for sepsis. Despite several well-documented limitations, blood culture remains the gold standard; automation has substantially reduced the time needed to detect circulating bacteria and currently almost 90% of positive blood cultures are detected within 24 h of incubation. Attractive alternatives to culture-based techniques are mainly based on the Polymerase Chain Reaction (PCR) for amplifying a specific region in the bacterial genome to levels sufficient for detection and identification of the bacteria. Conventional biochemical tests for evaluating the inflammatory response include C-reactive protein (CRP), procalcitonin (PCT), fibrinogen, and haptoglobin. Despite many hundreds of published clinical studies involving ten of thousand of patients, no one so called emerging biomarker has come definitively to the bed side and passed the test of time by being incorporated into routine clinical practice. Among the plethora of potentially emerging biomarkers, the soluble CD14 subtype (sCD14-ST), also called presepsin, seems to fulfill most of the characteristics of an ideal biomarker for managing neonatal sepsis. In a recent meta-analysis, both pooled sensitivity and specificity of sCD14-ST were 91.0% and the diagnostic odds ratio was 170.28 [4]. Recently, the potential role of metabolites has emerged as key issue for the early diagnosis and prediction of neonatal sepsis [5]. Several metabolic pathways may be altered in neonatal sepsis, including those involved in the acute phase response, hypoxia, oxidative stress, energy supply. In a recent study on urine of septic newborns, we found a significant increase of glucose, lactate and acetate as well as a significant decrease of tamarind *4-Hydroxybenzoic acid* (THBA), ribitol, ribonic acid and citrate [6]. Metabolomics seems to promise impressive improvements in managing sepsis by characterizing human from microbial metabolites (Rosetta Stone of microbiomics), by monitoring the effectiveness and the toxicity of the antibiotic treatment and by predicting clinical outcomes [7].

**References**

1. Reinhart K, Daniels R, Kissoon N, Machado FR, Schachter RD, Finfer S. Recognizing sepsis as a global health priority- a WHO resolution. N Engl J Med. 2017;377:414-17.

2. Fleischmann-Struzek C, Goldfarb DM, Schlattmann P, Schlapbach LJ, Reinhart K, Kissoon N. The global burden of paediatric and neonatal sepsis: a systematic review. Lancet Respir Med. 2018;6:223-30.

3. Iroh Tam PY, Bendel CM. Diagnostics for neonatal sepsis: current approaches and future directions. Pediatr Res. 2017;82:574-83.

4. Bellos I, Fitrou G, Pergialiotis V, Thomakos N, Perrea DN, Daskalakis G. The diagnostic accuracy of presepsin in neonatal sepsis: a meta-analysis. Eur J Pediatr. 2018;177:625-32.

5. Eckerle M, Ambroggio L, Puskarich MA, Winston B, Jones AE, Standiford TJ, et al. Metabolomics as a driver in advancing precision medicine in sepsis. Pharmacotherapy. 2017;37:1023-32.

6. Fanos V, Caboni P, Corsello G, Stronati M, Gazzolo D, Noto A, et al. Urinary (1)H-NMR and GC-MS metabolomics predicts early and late onset neonatal sepsis. Early Hum Dev. 2014;90 Suppl 1:S78-83.

7. Fattuoni C, Pietrasanta C, Pugni L, Ronchi A, Palmas F, Barberini L, et al. Urinary metabolomic analysis to identify preterm neonates exposed to histological chorioamnionitis: A pilot study. PLoS One. 2017;12:e0189120.

## A60 A baby with neurological, endocrine and feeding disorders: management issues

### Daniela Fava^1^, Flavia Napoli^2^, Natascia Di Iorgi ^1, 2^, Anna Elsa Maria Allegri^2^, Mohamad Maghnie ^1, 2^

#### ^1^ Università degli Studi di Genova, Genoa, Italy; ^2^ Clinica Pediatrica, IRCCS Istituto Giannina Gaslini, Genoa, Italy

##### **Correspondence:** Daniela Fava (galanti.fava@gmail.com)

**Case report**

A 3-month-old male with progressive obesity was referred for investigations. On admission, he presented apparent febrile seizures and cushingoid features. He was then transferred to the ICU department because of a refractory Status Epilepticus. The patient was born full term by c-section because of failure to progress. At birth he presented a poor Apgar scores and umbilical artery acidosis requiring intubation. He was passively cooled for 72 hours. Hormonal testing showed ACTH and TSH deficiencies which have been adequately replaced. On ICU admission, he was stabilized with intravenous antiepileptic drugs and transferred to the neurology department: clinical examination, EEG and MRI were suggestive of severe and prolonged hypoxic-ischemic encephalopathy. Altered thermoregulation and hypothermia were reported; RBCs and albumin were transfused due to anemia and hypoalbuminemia. Since his Status Epilepticus he presented dysphagia and a nasal enteral tube was placed for feeding. The care of the critically ill infant was further complicated by severe hypernatremia and hypokalemia: the baby was transferred to our endocrinology department. After neurological and gastroenterological evaluationsi, t was decided to perform a gastrostomy for enteral feeding in order to prevent aspiration pneumonia, dehydration and malnutrition and to make it easier to manage the child at home. The parents, who initially disagreed with the gastrostomy procedure, gave their consensus after an extensive discussion. Positive aspects**:** Child's parents were young, with a medium-high level of education, well integrated into the social context and belonged to a large family with great possibility of being supported from relatives and friends. Parents trusted medical staff. The baby had no other vital organ involved except the brain, had a good response to medical therapies and no situations occurred requiring cardio pulmonary resuscitation. Negative aspects**:** Case management from three different departments.; No psychological assistance for parents and staff; Lack of directives on what to do in case of emergency; Communication difficulties.

**Conclusions**

Communication is time for care.

Outline simple goals: one problem at a time in the present, medium and long-term.

Facilitate a quick and easy return home by: 1) shared-decision making involving the parents into an education program on managing the child; 2) activating a multidisciplinary care pathway, including the pediatrician and local health services.

Informed consent to publish has been obtained from the parents

## A61 The value of the osteopathic integrated approach in children

### Monica Filisetti, Donatella Cattarelli, Stefano Bonomi

#### Pediatric and Neonatology Department, Desenzano del Garda Hospital, ASST del Garda, Brescia, Italy

##### **Correspondence:** Monica Filisetti (monica.filisetti@gmail.com)

**Background**

Osteopathy is a manual therapy based on the evaluation and soft mobilizations of the musculeskeletal and fascial systems, based on the following principles:

- Structure and function are related and interdependents
- The whole body is a dynamic unit of function
- The body has an inherent therapeutic potency by itself [1].

Dr. Still is the founder of osteopathic medicine. The application in children is due to the work and dedication of Dr. Viola M. Frymann (1921-2016), physician, founder of the Osteopathic Center for Children in San Diego (California) and international reference point for neonatal and pediatric osteopathy [2-3].

The main reasons for consultation are musculoskeletal and postural problems, disorders related to development, and gastrointestinal functional disorders. Osteopathic approach have a potential role also in prevention, integrated into primary care. It could be considered also for children with neurological problems as complementary treatment in the rehabilitative program. [4-8] Pediatric osteopathy require specific competences and training and is considered safe for children. [9]

**Materials and Methods**

Since 2010 osteopathy is included in a program of Early Intervention in the Service of “Neurodevelopmental Follow up" in Desenzano del Garda Hospital (Italy). The Service is dedicated to newborns at risk for developmental disorders and offer a program of early intervention [10-14]. This program consists of:

- Pediatric and neurological examination
- Osteopathic evaluation and treatment, performed by physicians with experience in traditional pediatric osteopathy, following teachings of Doctor Viola M. Frymann
- Home program to promote development involving parents to create stimulating environment, improve relationship and support motor and sensory development, according with recent knowledge about child development and brain maturation. [15-18]

**Results**

During 8 years of experience, the Service take care of 530 newborn, referred from Neonatology Department and Territorial Services. Mean age at the first evaluation is 2 months.

Children present following conditions: prematurity, dystocial or hypoxic birth, postural and orthopedic disorders, congenital syndromes and neurological problems, gastrointestinal functional disorders. When necessary medical advice from other specialists is requested, in a multidisciplinary perspective. The proposed integrated approach is helpful in many aspects: postural setting, motor behavior, neuromotor development, gastrointestinal functions, infant-parent relationship.

Families accepted the proposed follow up program. No child had any adverse effects.

**Conclusions**

Our experience encourages to explore the potential of osteopathy in neonatal and pediatric care. The integrated osteopathic approach could be considered a model of early intervention to promote development in newborn at risk.

**Acknowledgements**

Authors thanks Dr. Viola Frymann for teachings and for encouragement to develop the program.

**References**

1. Still AT. The philosophy and mechanical principles of osteopahy. Kirksville, Missouri, USA, 1892.

2. Frymann VM. The collected papers of Viola M. Frymann: Legacy of Osteopathy to Children. American Academy of Osteopathy. Indianapolis, USA, 1998.

3. Frymann VM, Carney RE, Springall P. Effect of osteopathic medical management on neurologic development in children. J Am Osteopath Assoc. 1992;92:729-44.

4. Amiel-Tison C, Soyez-Papiernik E. Cranial osteopathy as a complementary treatment of postural plagiocephaly. Arch Pediatr. 2008;15 Suppl 1:S24-30.

5. Lessard S, Gagnon I, Trottier N. Exploring the impact of osteopathic treatment on cranial asymmetries associated with nonsynostotic plagiocephaly in infants. Complement Ther Clin Pract. 2011;17:193-8.

6. Sergueef N, Nelson KE, Glonek T. Palpatory diagnosis of plagiocephaly. Complement Ther Clin Pract. 2006;12:101-10.

7. Snider KT, Snider EJ, DeGooyer BR, Bukowski AM, Fleming RK, Johnson JC. Retrospective medical record review of an osteopathic manipulative medicine hospital consultation service. J Am Osteopath Assoc. 2013;113:754-67.

8. Morin C, Desrosiers J, Gaboury I. Descriptive study of interprofessional collaboration between physicians and osteopaths for the pediatric population in Quebec, Canada. BMC Health Serv Res. 2017;17:726.

9. Hayes NM, Bezilla TA. Incidence of iatrogenesis associated with osteopathic manipulative treatment of pediatric patients. J Am Osteopath Assoc. 2006;106:605-8.

10. Hadders-Algra M, Boxum AG, Hielkema T, Hamer EG. Effect of early intervention in infants at very high risk of cerebral palsy: a systematic review. Dev Med Child Neurol. 2017;59:246-258.

11. Spittle AJ, Morgan C, Olsen JE, Novak I, Cheong JLY. early diagnosis and treatment of cerebral palsy in children with a history of preterm birth. Clin Perinatol. 2018;45:409-420.

12. Novak I, Morgan C, Adde L, Blackman J, Boyd RN, Brunstrom-Hernandez J, et al. Early, accurate diagnosis and early Intervention in cerebral palsy: Advances in diagnosis and treatment. JAMA Pediatr. 2017;171:897-907.

13. Puthussery S, Chutiyami M, Tseng PC, Kilby L, Kapadia J. Effectiveness of early intervention programs for parents of preterm infants: a meta-review of systematic reviews. BMC Pediatr. 2018;18:223.

14. Spittle A, Orton J, Anderson PJ, Boyd R, Doyle LW. Early developmental intervention programmes provided post hospital discharge to prevent motor and cognitive impairment in preterm infants. Cochrane Database Syst Rev. 2015 Nov 24.

15. Cioni G, Inguaggiato E, Sgandurra G. Early intervention in neurodevelopmental disorders: underlying neural mechanisms. Dev Med Child Neurol. 2016;58 Suppl 4:61-6.

16. Hadders-Algra M. Early human motor development: From variation to the ability to vary and adapt. Neurosci Biobehav Rev. 2018;90:411-427.

17. Adolph K and Franchak J. The development of motor behavior. Wiley Interdiscip Rev Cogn Sci. 2017; 8:10.

18. Moriceau S, Sullivan R. Neurobiology of Infant attachment. Dev Psychobiol. 2005; 47: 230–242.

## A62 New generation imaging for the identification of the epileptogenic zone in children who are eligible for epilepsy surgery

### Thomas Foiadelli^1^, Katrien Jansen^2^, Lieven Lagae^2^, Salvatore Savasta^1^, Gian Luigi Marseglia^1^

#### ^1^ Pediatric Clinic, Fondazione IRCCS Policlinico San Matteo; University of Pavia, Pavia, Italy; ^2^ Department of Development and Regeneration, University Hospitals Leuven, Leuven, Belgium

##### **Correspondence:** Thomas Foiadelli (t.foiadelli@smatteo.pv.it)

**Background**

One out of three children with epilepsy is drug-resistant. Children with drug-resistant epilepsy must undergo timely and thorough investigations to assess eligibility for epilepsy surgery. Subtraction ictal-SPECT co-registered to MRI (SISCOM) is a promising imaging technique that has been used in the recent years for the localization of the epileptogenic zone (EZ) in adults. We aimed to determine its feasibility and efficacy in children who are candidate for resective epilepsy surgery.

**Methods**

We retrospectively analysed all pediatric patients (≤16 years old) with drug-resistant epilepsy that had entered the screening protocol for epilepsy surgery in the University Hospital of Leuven (Belgium), from 2009 to 2018. Fifty-one SISCOM in 44 patients (24 males and 20 females) were included. Mean age was 9 years. SISCOM results were matched with the localization of the EZ as determined by seizure semiology and video-EEG, and compared to traditional imaging techniques. **Results**

SISCOM was feasible in terms of chronic medication management, need for rescue antiepileptic therapy during hospitalization and need for procedural sedation, even in very young children. Major quality outcomes in terms of operative timings were satisfied: 1) radiotracer injection occurred within 30 seconds from seizure onset in 91.4% of the patients; 2) ictal SPECT was performed within two hours from injection in all patients (mean: 40 minutes). SISCOM localized the EZ in 51%, and additionally lateralized the EZ in 17.6%, achieving significantly better results than ictal-SPECT, PET and MRI. SISCOM successfully localized the EZ even in 25% patients with non-informative video-EEG and in 27.8% with negative MRI. Of the 16 patients (36.4%) finally selected for surgery, 87.5% were seizure-free at 12 months (vs 29.6% of those who were not operated). Overall, a localizing SISCOM was associated with a good final outcome (Engel I-II) in 75% of the patients.

**Conclusion**

SISCOM is feasible in children, and adds precious non-invasive presurgical information with a potential prognostic value, especially when the EZ cannot be localized by EEG and MRI.

## A63 Newborns, infants, new parents: educational disorders

### Oronzo Forleo (forleoro56@gmail.com)

#### S.S. Annunziata UTIN, Taranto, Italy

The aim of this work is to clarify the complex phenomenon of motherhood at early age, analyzing the risk and protection factors of these new parents. The transition to the motherhood, in the case of the adolescents or underage, seems to be a particularly stressfull phenomenon because it conflicts with physical, emotional, cognitive and social challenges which are typical of this period of development. The phenomenon of motherhood at early age is certainly characterized by greater vulnerability and is related to pregnancy (obstetric complications and high risk of depression), lifestyles that put the health of the fetus at risk (abuse of substance, alcohol, drugs), increase job difficulties (parents less present in moments of care), low levels of instruction, less social support and unstable relationships. The children of the underaged mothers are at greater risk of development problems (such as: prematurity, lower academic performance, greater socio-emotional difficulties, behavioral problems) and also the mother-child attachment is characterized by the risk of greater criticality: a lower wealth of verbal communication, lower emotional closeness, punitive tendencies and devaluation of the cognitive skills of little child. The attachment of these children to young mothers is avoidant and disorganized, as a result of a limited availability on the maternal emotional level. All these factors characterize the new parental figures as high educational risk. Therefore, we are in front of a social need that we cannot ignore, but a global take-over of motherhood in the adolescence becomes necessary, considering the cognitive, emotional, relational and social aspects of the future mother. In this perspective, it will be useful presented to you an experimental protocol being implemented at the Complex Neonatal and Neonatal Intensive Care Unit of Taranto.

## A64 What is monitoring? Why monitoring a child patient? From the use of a “pulsilogium” to vital sign monitors and early warning scores

### Sara Forti, Arianna Dondi

#### Pediatric Emergency Unit, S.Orsola-Malpighi Hospital, University of Bologna, Italy

##### **Correspondence:** Arianna Dondi (arianna.dondi@gmail.com)

Monitoring is repeated or continuous observations or measurements of the patient, his or her physiological function, and the function of life support equipment, for the purpose of guiding management decisions, including when to make therapeutic interventions, and assessment of those interventions. The earliest acquisition of physiological data date back to 1625 when Santorio, who lived in Venice at the time, published his methods for measuring body temperature with the spirit thermometer and for timing the pulse rate with a pulsilogium, a device he created inspired by Galilei’s pendulum. Since then, things are keeping on evolving. Nowadays, monitoring of vital signs is the most efficient method for collecting accurate readings. Modern medicine relies on these data, inserted in the clinical context, to make accurate diagnosis, treatment planning, illness progression and follow-up. Individual devices can be used to measure separate aspects of the patient’s health, but combination monitors are most frequently used, especially in critical-care situations when vital signs can suddenly fluctuate. The most commonly required vital signs are heart rate, pulse oximetry, blood pressure, respiratory rate, temperature, electrocardiogram. Monitoring can be sequential (to screen children’s illness or deterioration, measure success of treatment) or more frequent and rigorous or even continuous (for children with risk factors for deterioration, or for those undergoing high risk treatments). In fact, in those cases in which vital signs can change quickly and unexpectedly, continuous monitoring reduces the chances of missed signals due to clinical teamwork overloads or sporadic variability. Even outside the intensive care units, repeated measurement may help early recognition of deterioration, improving morbidity and mortality. For an effective management of clinical worsening in hospitalized children, signs and symptoms of deterioration have to be recognized by the ward staff; secondly, staff should be empowered to call promptly for assistance which should be quickly available and appropriately skilled and this response should improve the outcomes. The monitoring of the vital signs is now part of routine clinical care and it is essential, alongside with clinical features, because it contributes to early warning scores designed to detect patients at higher risk of deterioration and possibly resulting in a near or actual cardiopulmonary arrest which may be preventable. This supports the idea that the quality of clinical decisions depends in part on the quality of information available to the decision-maker.

## A65 Non diabetic hyperglycemia in pediatric age

### Adriana Franzese (franzese@unina.it)

#### Department of Translational Medical Science, Section of Pediatrics, Federico II University of Naples, Naples, Italy

Non-diabetic Hyperglycemia (HY) is a pathological condition that must be understood and probably not appropriately treated, although is not due to a chronic insulinopenia nor to a chronic insulin resistance. However, while long-lasting HY of diabetic condition has been known that impacts immune defenses and that is an important factor able to increase endothelial damage and degenerative oxide-reduction processes, little is known about the biological effects of non-diabetic and non-long-lasting HY, in particular in pediatric age, and validated protocols to manage this condition are completely lacking. Stress HY is usually defined as HY>150 mg/dl in the course of physical stress, resolving spontaneously after dissipation of acute illness in patients without known diabetes. Literature and clinical practice allow to design two different situations: the first during severe and prolonged illness and during seriously threatening survival, mainly in emergency conditions and in resuscitation departments; the second during acute illness mainly non-threatening life. Furthermore non diabetic-HY in pediatric age it is possible as Drugs-induced Hyperglycemia: the most frequent conditions are secondary to Steroid therapy or Antineoplastic/immune-suppressor therapy.

## A66 The reasons for a statement on behavioral emergencies in adolescence

### Carlo Fraticelli (carlo.fraticelli@asst-lariana.it)

#### Department of Mental Health and Addictions, ASST Lariana, Como, Italy

Managing behavioural and psychiatric emergencies in developmental age is one of the most challenging areas of practice for paediatric, emergency, psychiatric, and child neuropsychiatric clinicians and is a very serious clinical and organizational national concern. Although children and adolescent present to the emergency evaluation for various reasons including psychosocial problems, the most common are suicidality, psychosis, agitation, aggressiveness, abuse, and eating disorders. The problem of increased access of young people with psychiatric problems to Emergency Departments (where processes and facilities may be limited, creating a suboptimal environment for their care) and more frequent admission to hospital wards, has been repeatedly documented and is becoming a public health issue. In fact, the World Health Organization estimates that by the year 2020, the most prevalent causes of morbidity, and disability for children will be neuropsychiatric disorders (1). The emergency room contact represents one of the keys to address the problem and it is a critical link for continuity of care for mental health needs. More integrated strategies for the childhood area combining early evaluation and multidisciplinary intervention can be of increase benefit to individuals and to social contexts. Today the current organization of mental health services is going towards these direction. Geographical variability in service provision has diminished over time: in many regions substance misuse disorders and mental disorders in children and adolescents are treated by specialist services which are part of the Department of Mental Health with better integration among different services. It is time to promote closer integration also with the rest of healthcare. Health agencies should pay particular attention to the psycho-physical conditions of children and adolescents and adequate resources should be allocated to mental health prevention, detection and intervention programs. Members of several Italian Scientific Associations (AcEMC, ACP, CNI SPDC, SIEP, SIMEUP, SINPIA, SIP, SIP-DIP, SISISM, SITOX) have shared a document with ten statements, with the aim of providing guidance to improve the quality of care. Safety, legal issues, training and education, homogeneous care paths on the national territory, attention to care rights of a particularly vulnerable population, and efficient assessment of needs and appropriate level of intervention required to professionals, are the main issues described in the document.

**References**

1. Haddad F, Gerson R. Helping kids in crisis: managing psychiatric emergencies in children and adolescents. American Psychiatric Publishing, Inc; US, Arlington, VA: 2015.

## A67 Diagnosis provided by details: silver nitrate burns occurring after pyogenic granuloma treatment

### Francesco Fugetto, Iria Neri

#### Division of Dermatology, Department of Experimental, Diagnostic and Specialty Medicine, University of Bologna, Bologna, Italy

##### **Correspondence:** Francesco Fugetto (francescofugetto@gmail.com)

**Background.** Pyogenic granuloma is a common, benign lesion that needs treatment because of its bleeding tendency. Local cauterization with silver nitrate represents a relatively easy and cheap procedure and can be performed in the office. Although generally safe, silver-containing products can cause several cutaneous and systemic complications such as chemical perilesional burns and argyria. Moreover topical silver nitrate treatment carries the risk of chemical burns also in unaffected skin when not used properly.

**Case Report.** A nine-year-old girl was referred for a few-days history of discrete, well defined multiple superficial erosions symmetrically distributed on her buttocks. The lesions had firstly started as pitted erosions, progressively surmounted by blackish necrotic crusts. The patient was born in Italy at 40+2 weeks of gestation via vaginal delivery. Her perinatal history was complicated by maternal chorioamnionitis and perinatal asphyxia, which required admission in neonatal intensive care unit and resulted in hypoxic-ischemic encephalopathy. Her medical history was characterized by seizures, spastic tetraparesis, visual disturbances, and alimentary difficulties, which required gastrostomy. On clinical examination we appreciated multiple, millimetric (1-2 mm of diameter) ulcerations with central black scar and erythematous halo located mainly on the right buttock but also scattered on the left buttock and lower right back (figure 1). Between the ulcers we observed brownish striae (figure 2) and at the site of gastrostomy we noted also a residual of pyogenic granuloma treated with silver nitrate pencils (figure 3). By recording accurately the anamnestic data, the correlation between the gluteal erosions and the treatment of pyogenic granuloma was suggested by black stain of the skin due to the past use of silver nitrate. This data allowed us to make our diagnosis of silver nitrate burns, which were probably caused by pulverized medication fallen on examination table during the treatment of granuloma, and exclude other diagnoses such as herpes simplex virus infection and Langerhans cell histiocytosis.

**Conclusion.** As a general rule, it is crucial to make a comprehensive anamnesis and physical examination. This is particularly true approaching complex patients as in this case, when a detail could allow us to make the proper diagnosis without requesting further analyses or fears of more serious etiologies like abuse.

**Consent to publish**

Informed consent to publish has been obtained from this patient’s parents.

**Fig. 1 (abstract A67).**
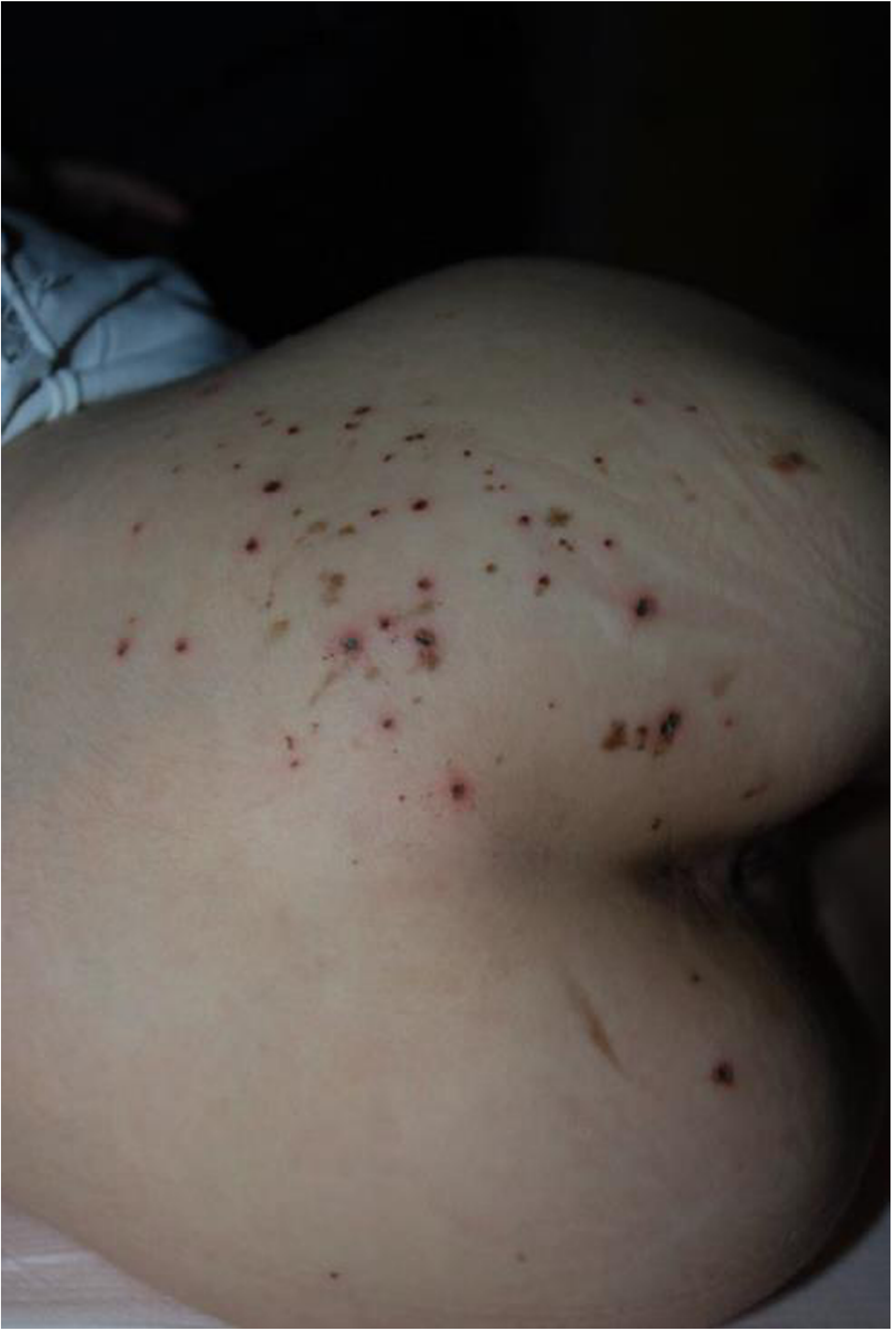
Ulcerations with central black scar and erythematous halo on the buttocks and lower back of the patient

**Fig. 2 (abstract A67).**
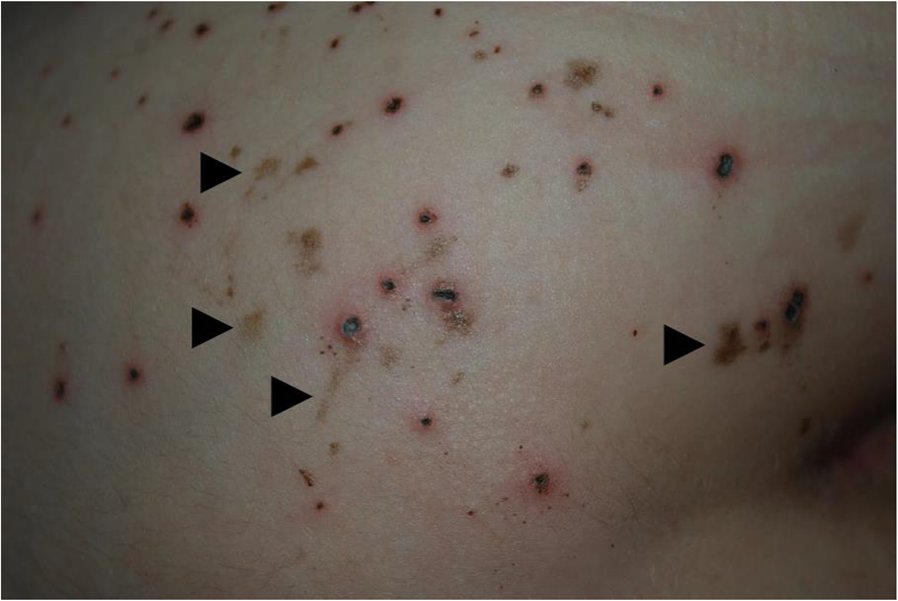
Brownish striae (black arrowheads)

**Fig. 3 (abstract A67).**
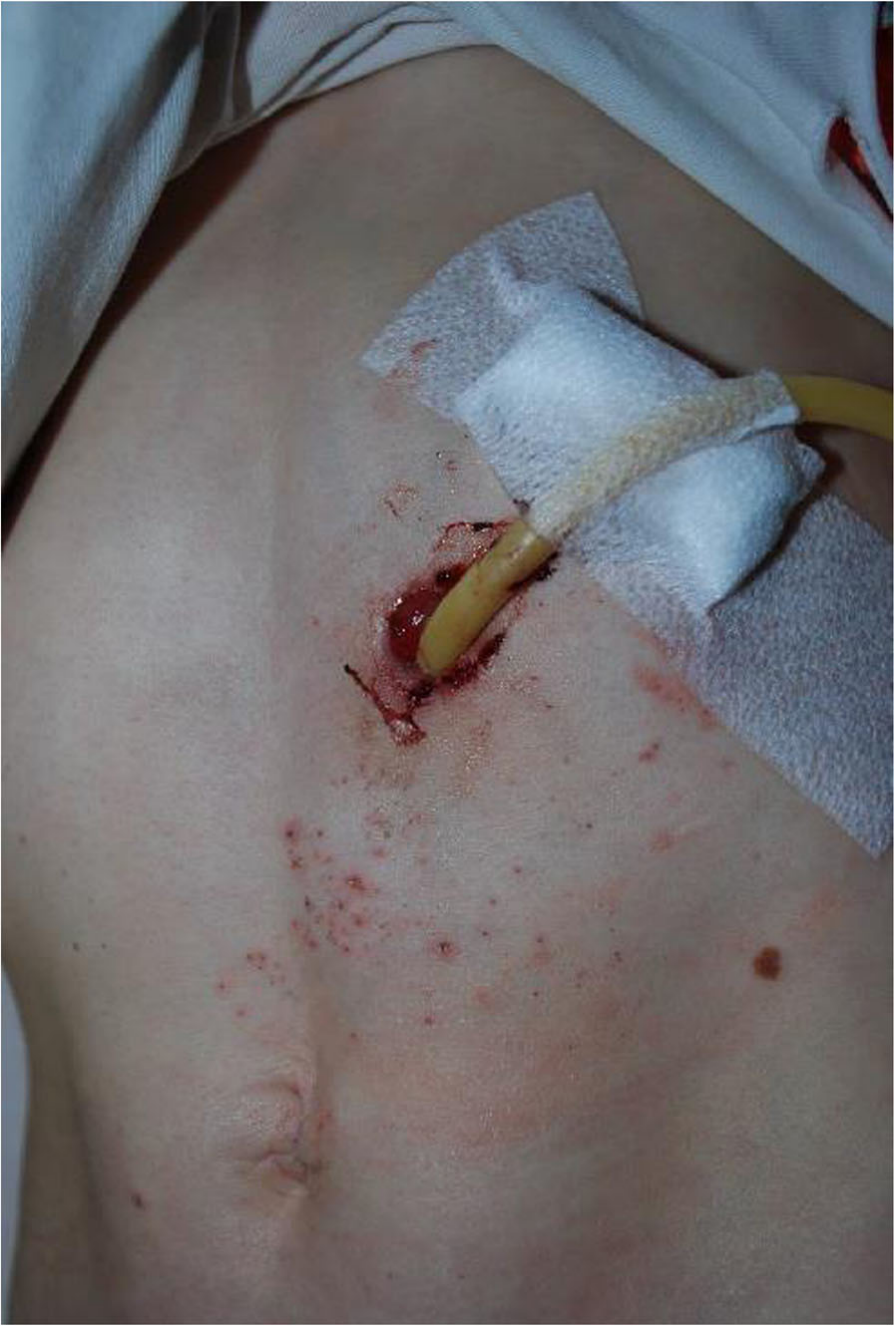
Pyogenic granuloma at the site of gastrotomy

## A68 If you recognize…you know: which kind of physical examination?

### Claudia Gandolfi (claudiagand@yahoo.it)

#### National Working Group for Migrant Children of the Italian Society of Pediatrics Red Cross Volunteer Doctor, Italy

In the context of health assistance of newly arrived migrant minors,

medical examination has an important role and requires a special attention; a careful and modulated physical examination is essential to evaluate health condition of migrant children not only to make a diagnosis but even to prevent other pathologies and to promote future well-being. During medical examination different typologies of migrant should be considered such as adopted children, unaccompanied minors, often adolescent boys and girls, children arrived with family in different ways, by sea, by land or immigrated after a long separation period from his parents, sons of asylum seekers, and “tourist migrants” , children coming back from countries of origin of their parents where they spent an holiday period; it is also necessary to consider the socio-cultural context where they lived and they grew up and geographical areas from which they come. Medical examination starts with a general evaluation which includes the assessment of vital signs: body temperature, pulse rate, breath rate, blood, pressure, hydration (dehydration signs and symptoms), weight and height (using the WHO Child Growth Standards) and the assessment of nutritional status; it goes on with an examination by system, giving particular consideration to important signs that should be detected and relative diagnostic hypothesis. A special attention is paid to pathologies which are more frequent in children coming from certain geographical areas, to journey related diseases, to signs of suffered violence and abuse, to aspects related to the culture of origin of the child, like results of ritual practices and of traditional medicine. With relation to skin examination, characteristics and peculiarities of dark skin have been stressed such as the diagnostic difficulties that can be experienced. Medical examination includes hearing, vision and oral health assessment, neurological, psychomotor and cognitive assessment, speech and language assessment, essential to highlight post traumatic symptoms related to separation, Ill-treatment, experienced or witnessed violence.

## A69 Pediatric publishing: Quaderni ACP

### Michele Gangemi (migangem52@gmail.com)

#### Pediatrician in Verona, Italy. Director of Quaderni ACP

The Quaderni ACP is a bi-monthly magazine published by the Pediatric Cultural Association (ACP). The target is represented by the non-super-specialized territorial and hospital pediatrician. The magazine is indexed in Google Scholar and is sent to ACP members in a paper format, while the online version (http://www.quaderniacp.it) is in open access.

Quaderni ACP does not host advertising and is entirely financed by the ACP. It was founded 25 years ago from the merger of “Quaderni” and “Teaching Aids”. Great attention is paid to health policy and pediatrician training. In the past seven years the magazine has activated a distance learning (FAD) based on skills training and with each dossier integrated with educational cases. This mode allows to switch from knowledge to skills. Each edition provides 18 ECM credits with a sponsor free high quality training and low costs. The path of the articles submitted to the journal is based on a peer review with internal and external referrals to the editorial staff. A special form is used to help the reviewer in his report. The articles, if necessary, are sent back to the authors with comments for optimization or possible re-presentation. The articulation of the magazine is based on fixed columns that host contributions from various authors not only in the pediatric area, but also in other fields of knowledge (psychological, pedagogical). Quaderni has also a part dedicated to medical humanities (books, films), often overlooked in pediatric education. In the last two years the magazine has been enriched with the “Electronic Pages” that host the ACP pediatric newsletters (critical reading of articles of main international scientific journals selected by specially trained groups), a section dedicated to the environment and child health and a third section that hosts documents relevant in the area of public health with expert comments.

The aim of the magazine is to create a professional who is attentive to complexity. There is also a careful look at the young pediatricians in training who are hosted with their abstracts both at the national ACP congress and at the yearly Tabiano / Parma congress. The editorial staff works mainly online and with face to face meetings at least twice a year in order to share the editorial line which is essentialy produced through a co costruction pathway. This way of working allows a continuous growth of the group of editors through both a real comparison and a particular attention to the reader.

## A70 Future nurses education: multimedia learning and assessment

### Andrea Gazzelloni, Valentina Pizziconi, Francesca Maria Meloni, Monica Venanzi, Giuliana D’Elpidio

#### Scuola delle Professioni Sanitarie “Pier Giorgio Frassati”, Ospedale Pediatrico Bambino Gesù, Roma, Italia

##### **Correspondence:** Andrea Gazzelloni (andrea.gazzelloni@opbg.net )

**Background**

Providing future nurses with professional knowledge is an ambitious and challenging task. Scientific knowledge changes quickly, technological innovations provide several possibilities. New skills and capacities are required by student nurses in the modern era. Education has to take into account these factors. Assessment is an important part of the educational process. This could be supported by modern technology. New technologies have the potential to give students the opportunity to show their practical skills and knowledge. Game-based assessment could provide such opportunity.

**Materials and methods**

The free game-based learning platform Kahoot! was used to assess knowledge for four nursing practical sessions planned for the first-year nursing degree. At the end of the four sessions, students were asked to evaluate the new assessment method. Data was collected from an anonymous web survey and the participation was on voluntary basis administered. The questionnaire investigated students’ opinions and feelings about multimedial assessment tools and the Kahoot! experience. Ethics Committee approval was obtained for this study.

**Results**

Forty students were involved in the present study and 93% of them positively evaluated the multimedial tool to assess: knowledge (97%); capacity (84%) and to clarify doubts (92%). 96% of students evaluated Kahoot! as an easy tool to use. The competitive and playful nature of the test was positively evaluated. Overall, students evaluated their Kahoot! experience positively at 92% and their multimedial assessment experience at 96%.

**Conclusions**

Assessment could become an opportunity to express and further develop personal skills. Interactive game-based assessment could be a method in order to offer this kind of opportunity to students. Feedback from the first-year nursing students was positive in our experience. Multimedial and game-based assessment should be considered an interesting method to explore. Possibilities to use modern technologies to develop and assess skills for future nurses should be further investigated.

## A71 Sustainability and reporting system in health education projects in schools

### Grazia Gentile^1^, Antonio Pagano^2^, Maria Carmen Pagano^3^

#### ^1^Departmental Rehabilitation and Prosthetic Service, ASL Salerno, Salerno, Italy; ^2^ ASL Naples 3 South, Naples, Italy; ^3^ Faculty of Medicine and Surgery "Magna Graecia" University, Catanzaro, Italy

##### **Correspondence:** Grazia Gentile (g.gentile@aslsalerno.it)

**Background**

Health Promotion, a concept codified in 1986 by the Ottawa Charter and defined by the WHO process that allows people to exercise greater control over their health and improve it, constitutes the current reference document for the development of health-oriented and identifies policies and identifies in the school the privileged gravitational area in which to send educational messages. The Ministry of Public Education promotes health education in carrying out educational activities in a network with Local Health Authorities, whose mission is to promote health in integration with schools.

**Materials and methods**

Review of health education projects in schools (years 2017-2019) of 2 Health Centers: ASL Salerno and A.S.Re.M.

**Results**

Both the projects examined are essentially based on: the drafting of a catalog of health education projects as a training offer addressed to schools; programming linked to indications from the Ministry of Health and Regional Prevention Plans; projects related to general objectives and quantitative indicators; target recognized in students of schools participating in the offer; financing deriving from the funds for prevention. The catalogs examined do not show: projects shared with schools; identification of specific territorial needs; final objectives; outcome indicators; cost/benefit analysis; no-responder schools recovery actions; time management; estimation of sustainability; publication of the results.

**Conclusion**

The National Health System, characterized by defined resources, assigns 5% of the Health Fund to prevention. Sector studies indicate the quality of assistance linked to the evaluation of the results of the interventions produced. It is therefore essential for the maintenance of health education projects, environmental determinants able to have a positive impact on the implementation area, to improve the programming and reporting system. In line with the document "Integrated policy guidelines for the school that promotes health" (State-Regions Conference of 17 January 2019) aimed at the inclusion in the educational curriculum of school students of all levels of health promotion as an educational proposal continuous and integrated throughout the schooling, it would be appropriate for the Healthcare Authorities to re-qualify the methodological approach to the provision of health promotion services in schools: activating in the planning phase the management by objectives which suggests expected characteristics for the prefixed objective to be specific, measurable, achievable, relevant, timely, implementing reporting based on cost/ benefit analysis demonstrating equity, efficiency during the reporting phase and sustainability of the intervention and disseminating the results obtained to contribute to evidence-based medicine.

## A72 Obesity? Obesity!

### Maria Giuseppina Gregorio^1^, Maria Grazia Clemente^2^

#### ^1^ATSSardegna – UOC Cagliari Area Vasta, GSSP, Associazione C.Susini, Cagliari, Italy; ^2^Dipartimento di Scienze Mediche, Chirurgiche e Sperimentali, Università di Sassari, Sassari, Italy

##### **Correspondence:** Maria Giuseppina Gregorio (mariagiuseppina.gregorio@atssardegna.it)

The World Health Organization (WHO) defines obesity as ‘globesity’: a chronic disease so widespread to be a global epidemic. In our country, 44% of Italians are overweight. Around 5 millions of people (10%) are obese and 34% of them have bariatric complications.

However, the identification of obesity as disease is recent: in medieval Europe, fat was a sign of wealth, prestige, power and often beauty. It is from the second half of the 19^th^-century that it becomes object of medical attention. The Paleolithic is famous for artistic statuettes with “steatopygic” shapes, symbol of fertility. When the food was poorly available, as in times of famine, only fat women were able to conceive and to complete the pregnancy. From the Neolithic, men are hunters as well as breeders and farmers. The forms become thinner and with graceful contours. In ancient Egypt, Pharaohs wives, ideal figure of beauty, are thin with round hips and breasts. In Roman times, the forms become wide and pale. From the Greek Venus to the Junoesque Roman beauties, from the refined Renaissance women to the 17^th^-century, to Michelangelo's cherubs, expressive models of epochs so distant in space and time have in common the same round lines of a body that nowadays we would define “fat”.

The Greeks were the first to recognize obesity as a health disorder. Hippocrates wrote “corpulence is not just a disease in itself but omen of other diseases”; therefore, obesity’s several secondary pathologies were already known. The Indian surgeon Susruta linked obesity to the onset of cardiac diseases and diabetes, suggesting physical exercise as measure of prevention. In “De medicina”, Aulo Cornelio Celso wrote “obese are mostly strangled by acute diseases and difficulty in breathing. The Medical School of Salerno in the Regimen prescribes “If you want to live healthy and without ills, avoid worries, beware of anger, drink and eat, but little.” However, it is only after World War II that the binomial “roundness-beauty” is replaced by “thinness-beauty” and the press published “Nature or Nurture?” to point out the role of the genetics, but just think of the natural example of identical twins, the lifestyle habit, and the most recent studies on leptins and gremlins… Therefore, if the hereditary component accounts for the 5%, it should not be an alibi to give up the very capillary prevention programs able to significantly reduce the size of the problem, with also good economic effects.

## A73 Characterization of the patients with lower respiratory tract infection who needed intensive care admision to the Intensive Care Unit at Hospital Nacional de Niños “Carlos Sáenz Herrera” during the first 43 days of the respiratory season 2014

### Silvia Sanabria Fonseca (silviasanafo@gmail.com)

#### Intensive Care Unit, Hospital Nacional de Niños, San José, Costa Rica

**Introduction**

Retrospective, observational, descriptive study of 59 patients admitted to the Intensive Care Unit of Hospital Nacional de Niños from August 1st to September 15th, 2014 to determine the prevalence of the lower tract infections that require intensive care admission, etiology, risk factors, clinical presentation and treatment.

**Materials and methods**

In the study were included all the patients between 0 and 13 years who were admitted to the Intensive Care Unit during the period determined with bronchiolitis or pneumonia diagnosis. For the statistical analysis, frequencies were calculated with the respective confidence interval for all the quantitative categorical variables and the central tendency measures for qualitative variables.

**Results**

RSV was isolated in the immunofluorescence assay in 74% of the patients. Prematurity and passive smoking were the risk factors most frequently found. Cough and tachypnea were the main clinical symptoms. Mechanical ventilation was required by 71% of the patients.

**Conclusions**

The prevalence of lower respiratory tract infection at the Intensive Care Unit at Hospital Nacional de Niños “Dr. Carlos Sáenz Herrea” during the study period was 60% which demonstrate the important burden this condition represents in the pediatric population of Costa Rica, especially in infants.

Antibiotics were prescribed in 95% of patients. These findings have already been documented by Ferone et al and Alhuarby et al ^1,2^. The respiratory syncytial virus was the most prevalent viral agent ^3-6^ . More diagnostic tools are required to guide the antibiotic treatment on this population.

**References**

1. Ferone EA, Berezin EN, Durigon GS, Finelli C, Felicio MCC, Storni JG. Clinical and epidemiological aspects relates to the detection of adenovirus or respiratory syncytial virus in infants hospitalized for acute lower respiratory tract infection. J Pediatr. 2014; 90: 42-49.

2. Alharbi S, Van Caeseele P, Consunji R. Epidemiology of severe pediatric adenovirus Lower respiratory tract infections in Manitoba. 1991-2005. BMC Infect Dis 2012;12:55.

3. Kwofie T YA, Nkrumah B, Annan A, Nguah SB, Owusu M. Respiratory viruses in children hospitalized for acute lower respiratory tracy infection in Ghana. Virology Journal. 2012; 9:78.

4. Hatipoglu N, Somer A, Badur S, Unüvar E, Akçay-Ciblak M, Yekeler E et al. Virus etiology in hospitalized children with acute lower respiratory tract infection. Turk J Pediatr. 2011; 53: 508-516.

5. Guerrier G, Goyet S, Chheng ET, Rammaert B, Borand L, Te V, et al. Acute lower respiratory tract infections in Cambodian children: clinical and epidemiologic characteristics. Pediatr Infect Dis J. 2013; 32: e8-e13.

6. Hasan R, Rhodes J, Thamthitiwat S, Olsen SJ, Prapasiri P, Naorat S, et al. Incidence and etiology of acute lower respiratory tract infections in hospitalized children younger than 5 years in rural Thailand. Pediatr Infect Dis J. 2014; 33: e45-e52.

## A74 HPV vaccination in men, women, young persons, adults

### Roberto Ieraci (roberto.ieraci@aslroma1.it)

#### Asl Roma 1, Rome, Italy

Cervical cancer is the fourth most common malignancy worldwide.

HPV infection is related to:

- cervical, vaginal, and vulvar cancers
- penile cancers
- oropharyngeal and anal cancers as well as genital warts

The majority of HPV-associated cancers are caused by HPV 16 and 18 [2].

Cancer primary prevention is based on virus avoidance: vaccination is recommended to prevent HPV infections and related diseases [http://www.salute.gov.it/imgs/C_17_pubblicazioni_2571_allegato.pdf] [1]. Currently a 9-valente vaccine (including HPV 16 and HPV 18) was available for use in girls, women, boys and men. For people who have been adequately vaccinated with 2vHPV or 4vHPV, there is no recommendation regarding additional vaccination with 9vHPV [https://www.acog.org/Clinical-Guidance-and-Publications/Practice-Advisories/FDA-Approval-of-9-valent-HPV-Vaccine-for-Use-in-Women-and-Men-Age-27-45?IsMobileSet=false]. Pediatricians are mainly and strongly involved in HPV primary prevention to protect against cancers caused by these viruses: the vaccine is most effective when given at younger ages. Routine vaccination is recommended at 11 or 12 y.o., with two doses and over 15 y.o. with three doses. If the vaccination schedule is interrupted, the series does not need to be restarted [https://www.acog.org/Clinical-Guidance-and-Publications/Practice-Advisories/FDA-Approval-of-9-valent-HPV-Vaccine-for-Use-in-Women-and-Men-Age-27-45?IsMobileSet=false][1]. ACIP recommends vaccination with 3 doses for people aged 9 through 26 years with immunocompromising conditions because immune response to vaccination might be attenuated[1] [https://www.acog.org/Clinical-Guidance-and-Publications/Practice-Advisories/FDA-Approval-of-9-valent-HPV-Vaccine-for-Use-in-Women-and-Men-Age-27-45?IsMobileSet=false]. Vaccine side effects with HPV include similar profiles with other vaccines: mild pain at injection site, bruising, faintness and syncope from a vasovagal response. In case of inadverted administration in pregnancy, no teratological risk are reported [4].

In countries where HPV vaccination programmes have been introduced, decrease in cancer rates is speculated. In Australia, the first country to establish an HPV vaccination programme in 2007 (more than 70% vaccine coverage in girls and boys aged 12 and 13 years), 38% reduction in highgrade cervical dysplasia was revealed [4]. What are the barriers to HPV vaccination? We can identify four parental vaccine hesitancy or refusal:

- about 10% lack of knowledge
- about 20% of parents consider vaccine unnecessary for their adolescent
- about 8% of parents belief that adolescent wasn’t sexually active
- about 7% received misinformation about side effects and adverse reactions

There is no evidence that demonstrates increased sexual risk behaviors in vaccinated adolescents despite parental fear is that the adolescent would become sexually active once the HPV vaccination was completed [3-4]. Collaborating with parents and the healthcare team, providers can provide patient centered health promotion care that will decrease risks of cancer in the future [4].

Increasing vaccination rates for HPV should be a priority for all healthcare providers who work with adolescents.

**References**

1. Petrosky E, Bocchini JA Jr, Hariri S, Chesson H, Curtis CR, Saraiya M, et al. Use of 9-valent human papillomavirus (HPV) vaccine: updated HPV vaccination recommendations of the advisory committee on immunization practices. MMWR Morb Mortal Wkly Rep. 2015;64:300-4.

2. Cohen PA, Jhingran A, Oaknin A, Denny L. Cervical cancer. Lancet. 2019;393:169-182.

3. Holloway GL. Effective HPV vaccination strategies: What does the evidence say? An integrated literature review. J Pediatr Nurs. 2019;44:31-41.

4. Spinner C, Ding L, Bernstein DI, Brown DR, Franco EL, Covert C et al. Human papillomavirus vaccine effectiveness and herd protection in young women. Pediatrics 2019;143: pii: e20181902.

## A75 Impact of new NGS techniques on inherited disease diagnosis

### Achille Iolascon (achille.iolascon@unina.it)

#### Dpt of Molecular Medicine and Medical Biotechnology- University Federico II of Naples and CEINGE- Advanced Biotechnologies, Naples, Italy

One of the most important dates in the history of Medical Genetics and perhaps of the human race is represented by Tuesday 27 June of the year 2000. On that date, in fact, the prestigious newspaper "The new York Times" published on the front page and on all the columns available an article titled: "Genetic Code of Human Life is cracked by scientists". This date, in my opinion, marks the end of the Mendelian era and the beginning of what we can say " genomic and post-genomic era". Before the onset of the new century, the study of Mendelian diseases had moved very slowly and this was because the technologies to discover the chromosomal location / localization of the genes and their sequences were extremely long. This made it possible to ask and solve only one question at a time and for this reason the causative mutation was identified and an attempt was made to explain the relationship between genotype and phenotype with it. Today Next-generation sequencing (NGS) is a high-throughput methodology that enables rapid sequencing of the base pairs in DNA or RNA samples. Supporting a broad range of applications, including gene expression profiling, chromosome counting, detection of epigenetic changes, and molecular analysis, NGS is driving discovery and enabling the future of personalized medicine. To date, the major current application of next generation sequencing (NGS) in diagnostics is through disease-targeted tests for which multiple causal genes are known. Some studies have already demonstrated the utility of targeted-NGS (t-NGS) approach in the study of specific subtypes of inherited condition patients. Here, we described the diagnostic workflow based on t-NGS that we developed for the diagnosis of patients affected by Hemolytic anemias as an example of inherited diseases. In conclusion Next-generation high throughput sequencing technologies have become available in the last few years and are in continuous development and improvement. They have been widely used in many projects, e.g., whole genome sequencing, metagenomics, small RNA discovery and RNA sequencing. Their common feature is the extremely high throughput data generation. As a result, new issues have to be addressed in order to exploit the full potential of these new instruments: firstly, the data analysis step has become very time consuming and requires a competent amount of manpower and expertise in bioinformatics; secondly, adequate computing resources are necessary to handle the data produced. Last point to address is that is required a continuous between bioinformatics experts and clinicians to finalize the diagnosis.

## A76 Post-traumatic osteoarticular infections in a pediatric cohort

### Andrzej Krzysztofiak, Marco Roversi, Daniele Deriu, Annalisa Grandin, Elena Bozzola, Maria Rosaria Marchili, Alberto Villani

#### Academic Department, Pediatric and Infectious Disease Unit, Children Hospital Bambino Gesù, Rome, Italy

##### **Correspondence:** Andrzej Krzysztofiak (andrzej.krzysztofiak@opbg.net)

**Background**

Trauma-related osteomyelitis are uncommon in children, but potentially devastating. The diagnosis of infection is based on a combination of clinical examination, laboratory data, plain radiography, advanced imaging (CT, MRI or bone scintigraphy) and microbiological culture (wound swabs and/or tissue specimen) or molecular diagnosis [1, 2]. Current culture-based diagnostic techniques are poor predictors of infecting organisms because bacteria cannot be identified using standard culture techniques [1]. Among bacterial pathogens responsible of osteomyelitis following trauma and/or surgical fixation, the most frequent is *Staphylococcus aureus*, including MRSA [3,4]. Aim of our work is to determinate clinical and microbiological characteristics of our pediatric cases with post-traumatic osteomyelitis of extremity fractures.

**Material and Methods**

We retrospectively analyzed the cases of 14 children [Table 1] who developed osteomyelitis after surgical treatment of a fracture. The median age was 9.6 years (range 3.7 – 16.3 years). The mean period of recovery was 40.2 days (range 7 – 143 days).

**Results**

The most frequent localization of bone fractures and subsequent infection were femur (28%), tibia/fibula (28%), ulna/radio (28%), humerus/elbow (14%), pelvic girdle and metatarsal bones. At admission the mean value of inflammatory index were: ERS 40.8 mm/ h (range 5 – 100 mm/h), CRP 6.85 mg/dl (range 0 – 34.65 mg/dl), WC 8932 mm^3^ (range 5800 – 16080 mm^3^). The most frequently identified pathogens were *Staphylococcus aureus* (36 %), in one case MRSA, *K. pneumoniae*, *Pseudomonas aeruginosa*, *Enterobacter cloacae*. In most cases (43%) no germ was isolated. The most used antibiotics were ceftriaxone (36 %), rifampicin (36 %), meropenem (29 %), linezolid (21%), amoxicillin (14%) and vancomycin (14 %). Ciprofloxacin was largely used (57 %), mainly in the outpatient setting. The mean period of antibiotic therapy was 5.8 weeks (range 3 – 12 weeks). Half the patients (50 %) were treated with Kirschner wires and/or external fixation. Four patients (28%) underwent more than two surgical approaches. Three patients (22 %) were dismissed with permanent sequelae. No deaths were registered.

**Conclusions**

Osteoarticular infections in children may be a severe complication of any surgical intervention on a fractured bone. The mean hospitalization period is often prolonged according to the long-term therapy and recurrent surgical approach [1,4]. Molecular diagnostics usually identifies bacteria that are not detected by standard microbiological cultures and allows to set a more appropriate antibiotic therapy [5]. In our experience, a proper multidisciplinary management is crucial to prevent sequelae, mainly chronic infections and disturbances of bone growth. Nonetheless, further studies in a pediatric population are required in order to delineate the optimal management of surgery-associated osteomyelitis.

**References**

1. Firoozabadi R, Alton T, Wenke J. Novel strategies for the diagnosis of post-traumatic infections in orthopaedic trauma patients. J Am Acad Orthop Surg 2015;23:443-451.

2. Govaert GA, IJpma FF, McNally M, McNally E, Reininga IH, Glaudemans AW. Accuracy of diagnostic imaging modalities for peripheral post-traumatic osteomyelitis - a systematic review of the recent literature. Eur J Nucl Med Mol Imaging. 2017;44:1393-1407.

3. Peng J, Ren Y, He W, Li Z, Yang J, Liu Y, Zheng Z, Kates SL, Schwarz EM, Xie C, Xu Y. Epidemiological, clinical and microbiological characteristics of patients with post-traumatic osteomyelitis of limb fractures in southwest China: A Hospital-based study. J Bone Jt Infect. 2017;2:149-153.

4. McNeil JC, Vallejo JG, Hultén KG, Kaplan SL. Osteoarticular infections following open or penetrating trauma in children in the post-community-acquired methicillin-resistant Staphylococcus aureus era: The impact of Enterobacter cloacae. Pediatr Infect Dis J. 2018;37:1204-1210.

5. Peltola H, Pääkkönen, M. Acute osteomyelitis in children. N Engl J Med. 2014;370:352–360.

**Table 1 (abstract A76).** Clinical and Microbiological characteristics of patients with osteomyelitis after fracture

*N pat*	*Age years*	*Sex*	*Days from trauma*	*Days recovery*	*Pathogen*	*Site*	*Sequele*	*K-wire
1	11,2	M	21	39	*P. aeruginosa*	femur	no	yes
2	11,7	M	8	26	*K. pneumoniae*	femur	no	yes
3	5,2	M	60	28	*ND*	tibia	no	no
4	16,3	M	5	21	*S. aureus*	astragalus	no	no
5	6,4	M	15	50	*E. cloacae*	femur	no	no
6	3,7	M	96	90	*S. aureus MRSA*	tibia fibula	yes	yes
7	15,2	M	5	15	*ND*	femur	no	no
8	8,5	M	51	67	*S. aureus MSSA*	humerus	no	no
9	10,2	M	67	18	*S. aureus MSSA*	radius ulna	no	yes
10	12	F	3	20	*ND*	radius	no	yes
11	6	M	8	8	*ND*	fibula	no	no
12	8,5	M	88	143	*S. aureus MSSA*	humerus	yes	yes
13	6,3	F	32	31	*ND*	radius	no	yes
14	12	M	2	7	*ND*	fibula	no	no

* K-wire = Kirschner wire and/or external fixation

## A77 GLNBM SIP Health-Reception for Migrant Children and Young People: Up-to-date

### Simona La Placa (simonalaplaca@gmail.com)

#### National Working Group for Migrant Children of the Italian Society of Pediatrics (GLNBM SIP), Italy

**Background**

Foreign children have become over time a relevant fraction of the EU and non-EU resident population (20,2%) in Italy. Between 2014 and 2018, 92.366 migrant minors arrived in Italy, 70.499 (76,3%) were unaccompanied by a caregiver. Following the changes that have characterized migrant paediatric population, these children face particular risks, including being exposed to discrimination and marginalization.

**Materials and Methods**

Several protocols concerning health care interventions for migrant children have been redefined from time to time, according to emerging issues, by the National Working Group for Migrant Children (GLNBM – SIP, http://www.glnbi.org, http://www.sip.it).

**Results**

Between 2016 and 2018, the GLNBM was panel member of the National Giudelines Program concerning the screening for communicable and non-communicable diseases among migrants at the border and in the welcome centres [1]. In the meantime, international guidances and reports on refugee and migrant health were recently published [2,3,4]. Concerning all above, the GLNBM SIP made the commitment to update the latest procedures for the health-reception of migrant children drafted in 2013, in order to take into consideration current scientific evidence to support paediatricians in the management of children and young people with migrant background, avoiding any standardized approach as well as to focus attention on epidemiological and infectious aspects without any real benefits in terms of public health. A comprehensive individualized health assessment by a well-trained pediatrician can identify health care needs that might otherwise go undetected for prolonged periods of time. In addition, the link of newly arrived migrant children and their families with primary health care and specialist services, reduces costs. In this regard, according to the Convention on the Rights of the Child (1989), Italy guarantees free foreign children’s access to pediatric health services as well as their National Healthcare System (NHS) registration, independently from legal status of their parents [5].

**Conclusions**

A public health strategy to promote well-being and health in migrant children should have a holistic framework, targeting risk factors on individual, family and community levels. The particular vulnerability of migrant minors, due to social fragility and inadequate reception, impose multisciplinary and transcultural approach, increasingly widespread. There is a need to improve health professionals’ skills in culturally sensitive health education, as well as culturally sensitive outreach programmes improve awareness and uptake of services among migrant populations. Eliminating barriers to access to healthcare and promoting protection of health of all people on the move, including those in irregular status, needs to be prioritised [6].

**References**

1. INMP, ISS, SIMM Controlli alla frontiera. La frontiera dei controlli. Controlli sanitari all’arrivo e percorsi di tutela per i migranti ospiti nei centri di accoglienza. Sistema Nazionale per le Linee-Guida. 2017. http://www.inmp.it/lg/LG_Migranti-integrata.pdf. Accessed 8 August 2019.

2. European Centre for Disease Prevention and Control. Public health guidance on screening and vaccination for infectious diseases in newly arrived migrants within the EU/EEA. Stockholm: ECDC; 2018. https://ecdc.europa.eu/sites/portal/files/documents/Public%20health%20guidance%20on%20screening%20and%20vaccination%20of%20migrants%20in%20the%20EU%20EEA.pdf Accessed 8 August 2019.

3. WHO Regional Office for Europe. Report on the health of refugees and migrants in the WHO European Region: no public health without refugee and migrant health (2018). Available at: http://www.euro.who.int/en/publications/abstracts/report-on-the-health-of-refugees-and-migrants-in-the-who-european-region-no-public-health-without-refugee-and-migrant-health-2018 Accessed 8 August 2019.

4. Health of refugee and migrant children. Copenhagen: WHO Regional Office for Europe; 2018 (Technical guidance on refugee and migrant health). Available at: https://reliefweb.int/report/world/health-refugee-and-migrant-children-technical-guidance. Accessed 8 August 2019.

5. Gazzetta Ufficiale of the Italian Republic no. 65 of March 18^th^ 2017. Decrete of the President of the Council of Ministers (DPCM), January 12^th^ 2017 with the new essential levels of care - LEA (Ordinary Supplement 15). http://www.gazzettaufficiale.it/eli/id/2017/03/18/17A02015/sg Accessed 8 August 2019.

6. The Lancet Public Health. No public health without migrant health. Lancet Public Health 2018;3:e259.

## A78 Child's fragility in the internet age

### Marcello Lanari, Laura Morgan, Carlotta Biagi, Monia Gennari

#### UO Pediatria d’urgenza, PS e OBI – Policlinico universitario S. Orsola- Unibo, Bologna, Italy

##### **Correspondence:** Laura Morgan (laura.morgan@studio.unibo.it)

Recent studies on Internet use by minors show that 88% of Italian children use it daily, a number that rises to 97% in the age range 15-17 years and drops to 51% in the band 9-10 years [1]. The most popular online activities among children are related to communication and entertainment, with an increasing tendency to privatize the experience through smartphones. The 2017 figures show how teenagers of 9-17 years, without gender differences, spend, on average, 2.6 hours a day online [1].

It is now known that, despite the positive opportunities arising from Internet development, an uncontrolled and excessive use can lead to dependence and cause problems of physical, psychological and social health [2]. The web can be a dangerous tool for children, as they don’t always understand its power; for instance, due to their tendency to irresponsible behavior, adolescence can easily engage in illegal and aggressive social interactions without awareness of the consequences. There are many other risks for minors connected to virtual navigation, because of its easy access and the space-time freedom it allows. The consequences of uncontrolled Internet use are so serious that it has led to the definition of a new type of dependence called PIU “Problematic internet use”. Adolescents and young adults are the subject most at risk [3]. Internet addiction has been described as a comorbid for psychiatric depression, attention deficit/hyperactivity disorder, social phobia, anxiety and impulse control disorders, and personality disorders. In Italy an increasing phenomenon among young people, particularly adolescents, is the "Hikikomori" syndrome, which identifies those who isolate themselves from social life, locking up in their room and refusing any direct contact with the external world. Hikikomori no longer seems to be only a Japanese cultural syndrome, but a social problem that affects all economically developed countries. It has been estimated that in our country there are at least hundred thousand cases [https://www.hikikomoriitalia.it/p/chi-sono-gli-hikikomori.html]. It is therefore essential the stakeholder collaboration to create a network, both to prevent and provide support for minors and families who face these problems. In 2016 was signed the "Protocollo D’Intesa", a memorandum between Police, the Ministry of Education, the Emilia-Romagna Region and the University of Bologna, about the conscious use of new technologies by young people and the prevention of cyberbullying [ http://istruzioneer.gov.it/wp-content/uploads/2018/03/def.pdf ]. This led to the creation of a listening point in the pediatric emergency department of Sant'Orsola hospital, a center to inform but also early identify and manage situations at risk.

**References**

1. Mascheroni G, Ólafsson K. Accesso, usi, rischi e opportunità̀ di internet per i ragazzi italiani. I risultati di EU Kids Online 2017. EU Kids Online e OssCom. https://www.miur.gov.it/documents *Accessed 20 July 2019.*

2. Choi M, Park S, Cha S. Relationships of mental health and internet use in korean adolescents. Arch Psychiatr Nurs. 2017; 31: 566–571.

3. Roma P, Ricci F, Kotzalidis GD, Guidarelli B, Pancheri C, Mazza C, et al. Psychopathology and personality in problematic internet users. Riv Psichiatr. 2019; 54: 24-30.

## A79 “Life changing” treatments in pediatric allergy

### Amelia Licari (amelia.licari@unipv.it)

#### Department of Pediatrics, Fondazione IRCCS Policlinico San Matteo, University of Pavia, Pavia, Italy

Severe asthma in childhood is a major public health problem and burden to both patient and pediatrician. Despite the fact that conventional treatment options for children with severe asthma are extremely limited, there are now some “revolutionary, and often life changing, therapies” emerging for selected patients suffering with severe asthma.

As our understanding of the underlying pathophysiology of the asthma spectrum expands, a number of pathways have drawn attention and led to the development of new targeted interventions [1]. Among these, the pharmacological blockade of IgE represents a novel successful strategy that has been applied to many different therapeutic areas, whose severe asthma is one that had its greatest development in the last 15 years [2]. Omalizumab, the first available humanized monoclonal anti-IgE with pediatric indication (aged ≥ 6 years), is now recommended as add-on treatment for children with severe allergic asthma. Binding to free IgE, omalizumab reduces cell-bound IgE, down-regulates IgE receptors and prevents the release of pro-inflammatory mediators [2]. The efficacy and safety of omalizumab have been established by several randomized controlled trials specifically conducted in pediatric patients [3]. Overall, omalizumab was effective in reducing the rate of asthma exacerbations, the number of hospitalizations for acute asthma attacks and the related need of oral corticosteroids in severe asthmatic children; these effects resulted in better asthma control and improvement of quality of life of children and their families [3]. Recent studies also reported a significant decrease in the number of seasonal exacerbations triggered by respiratory viruses in association with the restoration of antiviral defenses (in particular type I interferon production) in treated subjects [3]. A large amount of safety data showed that omalizumab is generally well-tolerated. The main side effects reported were local (pain at the injection site, skin reactions) and with a short resolution; moreover, there is no evidence to support an increased risk of malignancy in patients treated with omalizumab [3]. Other biologic therapies have provided new evidence of efficacy in the management of severe asthma, such as anti-IL-5 and IL-4/13 monoclonal antibodies; however, their efficacy and safety in children still needs to be supported by adequate research within the targeted age group [4]. The approved biologics for pediatric severe asthma have emerged as important and effective treatments to improve asthma outcomes; they are expensive therapies and require careful selection of suitable patients through identification of biomarkers in addition to clinical need to justify prescribing.

**Acknowledgements**

The author declares no conflict of interest, financial or otherwise.

**References**

1. Licari A, Marseglia GL. Current and future challenges in pediatric severe asthma. Curr Med Res Opin. 2018; 34: 943-944.

2. Licari A, Marseglia G, Castagnoli R, Marseglia A, Ciprandi G. The discovery and development of omalizumab for the treatment of asthma. Expert Opin Drug Discov. 2015; 10: 1033-1042.

3. Licari A, Marseglia A, Caimmi S, Castagnoli R, Foiadelli T, Barberi S, Marseglia GL. Omalizumab in children. Paediatr Drugs. 2014;16: 491-502.

4. Licari A, Castagnoli R, Brambilla I, Marseglia A, Tosca MA, Marseglia GL, Ciprandi G. Asthma endotyping and biomarkers in childhood asthma. Pediatr Allergy Immunol Pulmonol. 2018;31: 44-55.

## A80 Obesity and genetics

### Sandro Loche, Manuela Gallo

#### SSD Endocrinologia Pediatrica e Centro Screening Neonatale, Ospedale Pediatrico Microcitemico “A. Cao”, AO Brotzu, Cagliari, Italy

##### **Correspondence:** Sandro Loche (sandro.loche@aob.it)

Obesity and its related health complications are a major health concern worldwide. Obesity is also associated with and increased mortality. Rarely, severe obesity in children can be the result of a monogenic abnormality. Most of the genes involved belong to the leptin/melanocortin pathway that controls food intake. Recent studies have shown that some of these disorders can be successfully treated with recombinant leptin or with MC4R agonist drugs. Other forms od severe obesity are part of complex developmental syndromes caused by monogenic disorders, imprinting defects and other genetic and epigenetic alterations. There are more than 20 rare syndromes associated with obesity, and most of them present also with mental retardation. Exemples of these syndromes are Prader-Willi syndrome, Bardet Biedl syndrome, Alstrom syndrome, WAGR syndrome and others. However, the large majority of forms of obesity are the result of a complex interaction between predisposing genes and the environment. More than 500 genetic loci have been associated with adiposity traits. The function of most of these gene variants is unknown. Some of them implicate pathways that act in the brain whereas others are involved in pathways regulating adipocyte biology. Some of these genes could be potential targets for the development of anti-obesity drugs. Finally, recent research have shown that obesity and its complications are associated with epigenetic alterations. The implicated genes seem to play a role in the development of obesity by controlling processes such as adipogenesis, inflammation, appetite and glucose tolerance. Epigenetic modificatios also are involved in prenatal programming which ultimately results in predisposing the offspring.

Obesity and its related health complications are a major health concern worldwide. Obesity is also associated with and increased mortality. Rarely, severe obesity in children can be the result of a monogenic abnormality. Most of the genes involved belong to the leptin/melanocortin pathway that controls food intake. Recent studies have shown that some of these disorders can be successfully treated with recombinant leptin or with MC4R agonist drugs. Other forms od severe obesity are part of complex developmental syndromes caused by monogenic disorders, imprinting defects and other genetic and epigenetic alterations. There are more than 20 rare syndromes associated with obesity, and most of them present also with mental retardation. Exemples of these syndromes are Prader-Willi syndrome, Bardet Biedl syndrome, Alstrom syndrome, WAGR syndrome and others. However, the large majority of forms of obesity are the result of a complex interaction between predisposing genes and the environment. More than 500 genetic loci have been associated with adiposity traits. The function of most of these gene variants is unknown. Some of them implicate pathways that act in the brain whereas others are involved in pathways regulating adipocyte biology. Some of these genes could be potential targets for the development of anti-obesity drugs. Finally, recent research have shown that obesity and its complications are associated with epigenetic alterations. The implicated genes seem to play a role in the development of obesity by controlling processes such as adipogenesis, inflammation, appetite and glucose tolerance. Epigenetic modificatios also are involved in prenatal programming which ultimately results in predisposing the offspring to the development ov obesity.

## A81 Background EEG and electroclinical seizures

### Silvia Lori (silvia.lori@unifi.it)

#### Neurophysiology Unit, Neuro-Musculo-Skeletal Department, Careggi University Hospital, Florence, Italy

Electroencephalographic recording is routine in neonatal intensive care units for the recognition of seizures, neurological monitoring of the child with brain damage and, in the preterm infant, whose births have increased.In newborns, epileptic seizures represent a neurological emergency that requires timely recognition, to determine the etiology and treatment, as specific as possible. The problem is even more complex when considering seizures in premature babies, where the peculiarity of brain development can generate clinical and electrographic crises with different characteristics at different times of neuronal maturation. Carrasco [1] hypothesizes that in the extremely preterm child the clinical and electrographic phenomenology of convulsions reflects the specific pathophysiology of brain development in that period of life. Newborns can present different types of convulsions: clonic, tonic, myoclonic (axial, focal, irregular), epileptic spasms and thin crises, including autonomous signs or automatisms. The main etiology is hypoxic-ischemic encephalopathy (about 45%) with early crises and variable semiology, including all types of convulsions. Stroke is associated with early-onset unilateral focal seizures and interictal EEG is asymmetric, with focal or unilateral patterns. Other etiologies are less often linked to epileptic seizures and must be sought as brain infections, metabolic disorders, chromosomal abnormalities, innate metabolism errors, brain malformations and vitamin B6 dependence [2]. Neonatal epileptic syndromes may present favorable prognosis (neonatal benign familial epilepsy) or unfavorable (early infantile encephalopathy with epilepsy, early myoclonic encephalopathy and partial migration in childhood) [3]. Knowledge of neonatal EEG patterns is needed to correct recognize seizures and interpretation of interictal brain activity.

The accuracy of the EEG background activity test recorded as an integrated EEG in the amplitude or in the conventional EEG in the early stages of the life of the preterm and the neonatal term [4] is desirable for the correct interpretation of the critical clinical picture, the therapeutic choice and of the neurological development outcome.

**References**

1. Carrasco M, Stafstrom CE. How early can a seizure happen? Pathophysiological considerations of extremely premature infant brain development. Dev Neurosci. 2019 4:1-20.

2. Plouin P, Kaminska A. Neonatal seizures. Handb Clin Neurol. 2013;111:467-76.

3. Donovan MD(, Griffin BT, Kharoshankaya L, Cryan JF, Boylan GB. Pharmacotherapy for neonatal seizures: Current knowledge and future perspectives. Drugs. 2016;76:647-61.

4. Fogtmann EP, Plomgaard AM, Greisen G, Gluud C. Prognostic accuracy of electroencephalograms in preterm infants: A systematic review. Pediatrics. 2017;139: e20161951.

## A82 The mouth breathing child: a multidisciplinary approach

### Valeria Luzzi, Antonella Polimeni

#### Department of Oral and Maxillofacial Sciences, “Sapienza” University of Rome, Rome, Italy

##### **Correspondence:** Valeria Luzzi (valeria.luzzi@uniroma1.it)

In addition to hereditary factors, eugnatic growth depends largely on the existence of both a static and dynamic balance of all the orofacial musculature. As expressed in Moss's theory of the functional matrix, there is a relationship of mutual influence between form and function, according to which the changes in size, shape and position of each craniofacial skeletal component are modulated by the respective functional matrices which perform the specific functions of swallowing, breathing, chewing, and phonation. Mouth breathing plays a central role in the development of malocclusion in the pediatric age as it interferes with the functional growth of the upper jaw, whose reduced growth will be correlated not only to a transverse growth deficiency of the nasal cavities, induced by a reduced airflow from obstruction of the upper airways, due for example to the presence of allergic rhinitis, but also from the postural changes of the intraoral soft tissues, as it happens for the tongue that, by assuming a low position at the level of the oral floor, will no longer perform its role of functional matrix of the palate, thus contributing to a transverse growth deficit of the latter. These positional modifications of the soft and structural tissues of the skeletal segments contribute to the pathognomonic definition of both the extra and intraoral aspect of the mouth breathing child. Therefore, allergic rhinitis is one of the main causes of malocclusions in the pediatric age, contributing with a significant threefold increase of the risk. It is estimated that in Italy the prevalence of allergic rhinitis for subjects in the age range from 6 to 14 is 33-35%, with an incidence that in the last five years showed an increase of 5%. Pathognomonic clinical signs define the typical adenoid facies, characterized by accentuated dark circles, narrow nostrils, and labial incompetence. Associated dentoalveolar alterations are a transversal hypodevelopment of the maxilla, anterior openbite, increased overjet, and marginal gingivitis, typically correlated to the lack of humidification of this soft tissue. In light of the above, although numerous epidemiological studies show that allergic pathologies are significantly increasing in the pediatric population, many of the malocclusal conditions associated to airway obstruction can be resolved by means of interceptive orthodontic therapies, aimed at preventing the onset of more serious dentoskeletal alterations. In this sense, the role played by the pediatrician is fundamental in identifying occlusal alterations and in making parents aware of the need of a preventive dental examination.

## A83 Biologic therapy in pediatric chronic non-infectious uveitis

### Ilaria Maccora, Gabriele Simonini

#### SODc di Reumatologia, AOU ANNA MEYER, Dipartimento NEUROFARBA, Università degli Studi di Firenze, Italia

##### **Correspondence:** Gabriele Simonini (Gabriele.simonini@unifi.it)

Uveitis is a broad term for inflammation involving the eye, the estimated prevalence in the pediatric age is 31 per 100,000 patients of whom the 21% are associated to Juvenile Idiopathic Arthritis (JIA). Chronic pediatric uveitis can be a complex therapeutic challenge, that if not appropriate, can lead to significant visual impairment and blindness without treatment. Unfortunately, there are no standardized treatment protocols for pediatric non-infectious uveitis; recently, the SHARE initiative has given us recommendation for the diagnosis, screening, treatment and management of uveitis associated to JIA. There is agreement for the first-line therapy with topical corticosteroids, although systemic corticosteroids are used in more severe cases. In children with severe and refractory uveitis, disease-modifying antirheumatic drugs (DMARDS) and biologic agent have a pivotal role in order to achieve remission and minimize corticosteroid side effects. Methotrexate is usually the first line-steroid-sparing agent, whilst anti-tumor necrosis factor-α (anti-TNFα) agents, as adalimumab and infliximab are currently used as second line agents, in case of failure. Anti-TNFα may be used as first-line treatment in those patients with a severe and complicated disease presentation. However, it has been shown a higher probability of maintaining remission on adalimumab than infliximab during the time of treatment, and a better efficacy of Adalimumab if used as first anti-TNFα therapy. Etanercept is not recommended for treating chronic non-infectious uveitis. When the first anti-TNFα agent failed, there is evidence that increasing adalimumab dose and/or administration or switching to a second anti-TNFα both result in efficacy. In children with anti-TNFα resistant uveitis Abatacept, Tocilizumab and Rituximab are other possible options. Abatacept is a soluble fusion protein composed of human cytotoxic T lymphocyte antigen 4 (CTLA-4), and have shown promising results without significant difference in improvement in those patients who received Abatacept as first-line therapy versus second-line. Tocilizumab is anti-IL6 is an emerging treatment for uveitis and cystoid macular oedema. Rituximab and canakinumab are progressively showing an emerging role in chronic resistant uveitis. The lack of randomized controlled trials and the absence of guidelines make difficult the timely and optimal treatment in this clinical setting. Since fair and limited evidence exists, the best way of tapering medications is still a challenge.

## A84 Complementary and Alternative Medicine (CAM) and antibiotic resistance

### Francesco Macrì (profmacri@gmail.com)

#### Study Group on Complementary and Alternative Medicine – Italian Society of Pediatrics, Roma, Italy

Antibiotic resistance (AR) levels in Italy remain among the highest in Europe. This is presumably linked to excessive use of antibiotics, which are used in the community by about 1.5 million people (2.5% of the population) every day. The incidence of *Klebsiella Pneumoniae* resistance to carbapenems increased from 1.3% in 2009 to 27% in 2011 and 33% in 2015, resulting in about 2000 cases of bacteremia per year. A similar phenomenon has also been observed with other pathogens (*E. Coli, P. Aeruginosa, S. Aureo*) [1]. The causes of AR development are complex, but undoubtedly include the excessive and inappropriate use of antibiotics. The size of the problem has led researchers to search for new drugs to combat AR, but the slowness in achieving useful results means that there is no short-term solution. Could complementary and alternative medicine (CAM) help fight AR? Some reports have shown that the use of CAM in therapeutic settings based on integrated medicine (IM), a model that combines the use of both conventional medicine and CAM , can produce a general saving in health expenditure [2], mainly due to the reduced prescription of conventional drugs. Several studies demonstrate that limiting the prescription of antibiotics for respiratory tract infections can lower AR [3]; see for example Friedman and Whitney’s excellent editorial of April 2008 [4]. A therapeutic approach that limits the use of antibiotics can therefore counteract AR. In 2018 Van der Werf and coll. compared the prescriptive behavior of GPs who had adopted IM with the behavior of conventional GPs, finding that the prescription of antibiotics by the first group was 22% lower [5]. Similar results were obtained in other studies investigating homeopathy [6]. A recent study by our group in children with acute otitis showed that antibiotics were prescribed in 33% of cases treated with homeopathy, versus 62% in the control group (p 0.006). In conclusion, there is literature evidence demonstrating that the use of CAM integrated with conventional medicine can reduce the use of antibiotics, thus making this a useful strategy to help combat AR.

**References**

1. Sabbatucci M, Iacchini S, Iannazzo S, Farfusola C, Marella AM, Bizzotti V, et al. et al. Sorveglianza nazionale delle batteriemie da enterobatteri produttori di carbapenemasi. Rapporto 2013-2016. Roma:Istituto Superiore di Sanità;2017.

2. European Programme for Public Health Microbiology Training, Stockholm: European Centre for Disease Prevention and Control;2013.

3. Rossi E, Crudeli L, Endrizzi C, Garibaldi D.l Cost–benefit evaluation of homeopathic versus conventional therapy in respiratory diseases Homeopathy. 2009;98:2–10.

4. Guillemot D, Varon E, Bernede C, Weber P, Henriet L, Simon S, et al. Reduction of antibiotic use in the comunity reduces the rate of colonization with Penicillin G-non susceptible Streptococcus Pneumoniae. Clin Infect Dis. 2005;41:930-938.

5. Friedman CR, Whitney CG. It’s time for a change in practice: reducing antibiotic use can alter antibiotic resistance. J Infect Dis. 2008;197:1082-3.

6. van der Werf E, Duncan LJ, val Flotow P et al. Do NHS GP surgeries employng GPs additionally trained in Integrative Medicine have lower antibiotic prescribing rate? Retrospective cross sectional analysis of national primary care prescribing data in England in 2016 BMJ Open 2018; e020488.

7. Jacobs J, Springer DA, Crothers D. Homeopatic treatment of acute otitis media in children: a preliminary randomized placebo- control trial. Pediatr Infect Dis J. 2001;20:177-183.

## A85 Anti-inflammatory treatment: treat to target

### Maria Cristina Maggio (mariacristina.maggio@unipa.it)

#### Department of Health Promotion Sciences Maternal and Infantile Care, Internal Medicine and Medical Specialities “G. D’Alessandro”, University of Palermo, Italy

Autoinflammatory diseases (AIDs) are monogenic disorders [1] characterized by overproduction of IL-1β. Remission, prevention of complications, control of inflammation, a good quality of life are goals of the treatment. A personalized treatment must be started precociously [2] and approved by clinicians, patients and their families, considering children in these long-lasting treatment decisions. A common therapeutic approach is effective in multifactorial AIDs, where inflammatory cytokines are increased by different triggers, as sJIA, Kawasaki disease, HLH, IBD, Steven Johnsons Syndrome, autoimmune diseases: the infusion of high doses of IVIG, modifying cytokines and immune response. IVIG are effective in the resolution of fever, hepatitis and/or cholestasis also if triggered by infections [3]. Anti-TNF-α drugs are effective in IBD and Bechet disease; Infliximab in the control of fever in refractory KD, not improving coronary artery lesions (CAL). Anakinra, IL-1 receptor antagonist, is effective on clinical manifestations and CAL [4-5]. Treatment of sJIA improved with anti-IL-1 (Canakinumab, Anakinra) and anti-IL-6 (Roactemra) biological drugs. The early employ of Canakinumab in a “therapeutic window” increases the possibility to gain the remission [6]. Systemic Lupus Erythematous and monogenic interferonopathies are successfully treated with JAK inhibitors, a high selective treatment. Remission is now evaluated by clinimetrics to define the efficacy of treatment.

**References**

1) Papa R, Doglio M, Lachmann HJ, Ozen S, Frenkel J, Simon A, et al. A web-based collection of genotype-phenotype associations in hereditary recurrent fevers from the Eurofever registry. Orphanet J Rare Dis. 2017;121:167.

2) Sota J, Vitale A, Insalaco A, Sfriso P, Lopalco G, Emmi G, et al. Safety profile of the interleukin-1 inhibitors anakinra and canakinumab in real-life clinical practice: a nationwide multicenter retrospective observational study. Clin Rheumatol. 2018;37:2233-40.

3) Accomando S, Liotta A, Maggio MC, Cardella F, Corsello G. Infliximab administration effective in the treatment of refractory Kawasaki disease. Pediatr Allergy Immunol. 2010;21:1091-2.

4) Kone-Paut I, Cimaz R, Herberg J, Bates O, Carbasse A, Saulnier JP, et al. The use of interleukin 1 receptor antagonist (anakinra) in Kawasaki disease: A retrospective cases series. Autoimmun Rev. 2018;17:768-74.

5) Maggio MC, Cimaz R, Alaimo A, Comparato C, Di Lisi D, Corsello G. Kawasaki disease triggered by parvovirus infection: an atypical case report of two siblings. J Med Case Rep. 2019;13:104.

6) Maggio MC, Ragusa SS, Corsello G. Early treatment of systemic juvenile idiopathic arthritis with canakinumab and complete remission after 2 years of treatment suspension: Case report of an adolescent girl. Clin Drug Investig. 2019;39:491-4.

## A86 Courtesy robotic bodies for small bedridden patients

### Roberto Mancin^1^, Flavio Sartoretto^2^, Agnese Suppiej^3^

#### ^1^Department of Women's and Children's Health, University of Padua, Padua, Italy; ^2^Department of Environmental Sciences, Informatics and Statistics, University of Venice, Venice, Italy; ^3^Department of Medical Sciences- Pediaric Section, University-Hospital of Ferrara, Ferrara, Italy

##### **Correspondence:** Roberto Mancin (roberto.mancin@unipd.it)

The scientific literature reports an increasing number of actions aimed at providing mobile robots that allow telepresence experiences to bed-ridden adult patients [1]. A recent review published in BJM Open [2] shows that the use of such robots is increasing very quickly. The nationality of the authors of the articles analyzed in this work is mainly Italy. The use of humanoid and empathic robots such as Nao and Pepper is spreading also in Italian pediatric hospitals. It follows that it is possible and appropriate, as already happens for adults, to use these robots not only for rehabilitation, surgery and distraction [Dawe et al 2019] but also to promote inclusion, by employing them as a telepresence robot. In this case it is no longer the medical-nursing staff that "guides" the robot used for the patient but the patient himself who uses the humanoid robot as a "courtesy body", a robotic avatar, controlled from the bed or from the stay. At present, applications of this kind reported in literature are very scarce and focused exclusively in protected contexts (hospitals and other places of care) but not in other environments (museums, schools, homes for holidays, mountain refuges). The number of articles containing the keywords "robot" and "telepresence" has increased exponentially in the period 2000-2017. Yet, nothing has been published to date regarding the developmental age. The aim of this work was therefore to verify whether, within a pediatric hospital, humanoid robots can have an inclusive role in contexts typical of the developmental age (school, sports, oratory, summer camps). In particular 3 models of humanoid robots were used in extra-hospital settings and remote-controlled by a underage patient. From these preliminary experiences, telepresence robotics carried out with humanoid and empathic robots seems to be promising for hospitalized children. Entrusting a small robot avatar use to each underage patient, with the goal to reach an earlier inclusion, is not only technically possible but also sustainable from the social, economic and environmental points of view. The use of BCI (Brain Computer Interface) technologies in children could make this opportunity universal.

**References**

1. Young J, Langlotz T, Cook M, Mills S, Regenbrecht H. Immersive Telepresence and Remote Collaboration using Mobile and Wearable Devices. IEEE Trans Vis Comput Graph. 2019;25:1908-1918.

2.Dawe J, Sutherland C, Barco A, Broadbent E. Can social robots help children in healthcare contexts? A scoping review. BMJ Paediatr Open. 2018;0:e000371.

## A87 Kawasaki Disease

### Alessandra Marchesi, Alberto Villani

#### UOC Pediatria Generale e Malattia Infettive, Ospedale Pediatrico Bambino Gesù, Roma, Italy

##### **Correspondence:** Alessandra Marchesi (alessandra.marchesi@opbg.net)

Kawasaki disease (KD) is an acute, systemic vasculitis [1,2]. According to the “Revised International Chapel Hill Consensus Conference Nomenclature of Vasculitides” of 2012, its target are small and medium diameter vessels in each organ and apparatus. (3) KD is a self-limited disease with unknown, probably multi-factor, aetiology, which primarily affects infants and children under five years. Diagnosis of Kawasaki disease is clinic, based on diagnostic clinical criteria, supported by the results of blood and instrumental exams. This presentation aims to highlight news in guidelines than those published in 2008 [2-4]. We will evaluate the differences in the definition of different forms, new laboratory applications updates in therapy both in responder- patients (eg. changing the length and dosage of anti-inflammatory therapy with ASA),and in non-responders, and in major risk patients (adding the steroid therapy).

**References**

1. Newburger JW, Takahashi M, Gerber MA, Gewitz MH, Tani LY, Burns JC, et al. Diagnosis, treatment, and long-term management in Kawasaki disease: a statement for health professionals from the committee on rheumatic fever, endocarditis and Kawasaki disease, council on cardiovascular disease in the young, American Heart Association. Pediatrics. 2004;114:1708-33.

2. Marchesi A, Pongiglione G, Rimini A, Longhi R, Villani A. Malattia di Kawasaki: Linee Guida italiane. Prospettive in Pediatria. 2008;38:266-83.

3. Jennette JC, Falk RJ, Bacon PA, Basu N, Cid MC, Ferrario F, et al. 2012 Revised international chapel hill consensus conference nomenclature of vasculitides. arthritis rheum. 2013;65:1-11.

4. Marchesi A, Tarissi de Jacobis I, Rigante D, Rimini A, Malorni W, Corsello G, et al. Kawasaki disease: guidelines of italian society of pediatrics, part I - definition, epidemiology, etiopathogenesis, clinical expression and management of the acute phase. Ital J Pediatr. 2018;44:102.

## A88 Early prediction and prevention of renal and other vascular complications in adolescents with type 1 diabetes

### M. Loredana Marcovecchio (mlm45@medschl.cam.ac.uk)

#### Department of Paediatrics, University of Cambridge, Cambridge, UK

##### **Correspondence:** Alessandra Marchesi (alessandra.marchesi@opbg.net)

Micro- and macrovascular complications continue to be a major concern for people with type 1 diabetes (T1D), particularly those diagnosed at a young age, who are exposed for a longer period to the metabolic derangements of diabetes. Although HbA1c is a major risk factor for their development, and the main focus of current screening and treatment strategies, recommended targets are rarely achieved. Thus, there is a clear need for improved markers to identify subjects at risk at an early stage and to develop additional intervention strategies to prevent future complications. Among early markers, increased urinary albumin excretion is considered as the earliest clinical manifestation of nephropathy, and an independent risk factor for retinopathy and cardiovascular disease. My work with cohorts of childhood-onset T1D (Oxford Regional Prospective Study (ORPS), Nephropathy Family Study (NFS), Adolescent Type 1 Diabetes cardio-renal Intervention Trial (AdDIT)) has highlighted the value of urinary albumin excretion (measured by albumin-creatinine ratio (ACR)), as an early marker of renal, retinal and cardiovascular complications. In the contemporary AdDIT cohort (n 800), an ACR in the top tertile of the normal range was associated with increased glomerular filtration rate, dyslipidaemia and increased arterial stiffness during early adolescence (age 11-16 years). In addition, adolescents with the highest ACR at baseline had higher rates of microalbuminuria and retinopathy, and greater increases in carotid intima-media thickness and cardiovascular markers (blood pressure, high-sensitivity C-reactive protein) during a subsequent 2-4 years follow-up period. Of note, in this young cohort there were no major differences in HbA1c across tertiles of ACR, and the effect of ACR on complication risk was independent of glycaemic control. These data support the concept that risk stratification using ACR during early adolescence may be critical for the early identification of patients at risk of developing renal, retinal and cardiovascular complications and to guide the implementation of preventive and treatment strategies to reduce the burden associated with vascular complications of diabetes.

## A89 Cornelia de Lange syndrome spectrum

### Milena Mariani^1^, Silvia Tajè^1^, Barbara Parma^1^, Anna Cereda^2^, Paola Ajmone^3^, Antonella Costantino^3^, Angelo Selicorni^1^

#### ^1^ Pediatric Department, ASST Lariana, Como, Italy; ^2^ Pediatric Department, ASST Papa Giovanni XXIII, Bergamo, Italy; ^3^ Child and Adolescent Neuropsychiatric Unit, Fondazione IRCCS Cà Granda Ospedale Maggiore Policlinico, Milano, Italy

##### **Correspondence:** Angelo Selicorni (angelo.selicorni61@gmail.com)

Cornelia de Lange (CDLS) is a genetic syndrome firstly described in 1993 by the homonym Dutch pediatrician [1]. Peculiar facial dysmorphisms, prenatal and postnatal growth retardation, psychomotor and cognitive delay, microcephaly, hirsutism, small hand and feet or more severe upper limb major anomalies represent the key features of the disease. Various major malformations ( palate, heart, genital, urinary tract, central nervous system) have been described with different frequency but none of them is part of the “core phenotype” of the syndrome. Behavioral and communication problems are frequently observed. Several medical complications are part of the natural history of the disease. Gastro-esophageal reflux is the more frequent of them. [2].

A wide variability of the phenotype has been observed so that Liu and Krantz stated that up 20-30% of the patients can show a so called “mild phenotype” [3]. The molecular history of the disease started in 2004 with the discovery of the first CdLS gene: NIPBL gene [4,5]. In the following years pathogenetic variant in several other genes, all related to the cohesion pathway, have been described in CdLS/ CdLS like patients (SMC1A, SMC3, HDAC8, RAD21, BRD4) and somatic mosaicism has been demonstrated too [6,7,8,9,10,11]. In addition variant in other genes usually related to different diseases have been observed in cohort of CdLS like patients (ANKRD11, EP300 TAF6, AFF4, KMT2A, NAA10) [12,13,14,15]. In 2018 an international group of professionals, strictly in contact with the CdLS World Federation, published the first international consensus statement in which recommendations related to clinical/molecular diagnosis, medical follow-up from pediatric to adult age, rehabilitation and behavioral issues have been discussed [16]. In this consensus it was stated the existence of a wide CdLS spectrum which includes patients with “classical” and “non classical” CdLS phenotype mostly related to mutation in cohesin genes; these phenotypes should be distinct from others not overlapping with the CdLS spectrum but sometimes related to cohesion genes mutation too. A clinical score has been define in order to differentiate the two possibilities

**References**

1. De Lange C. Sur un type nouveau de degenerescence (typus Amsterlodamensis). Arch Med Enfants. 1933;36,713–719.

2. Luzzani S, Macchini F, Valade A, Milani D, Selicorni A. Gastroesophageal reflux and Cornelia de Lange syndrome: typical and atypical symptoms. Am. J Med Genet. 2003;119A, 283–287.

3. Liu J, Krantz ID Cornelia de Lange syndrome, cohesin, and beyond. Clin Genet. 2009;76:303-1

4. Krantz ID, McCallum J, DeScipio C, Kaur M, Gillis LA, Yaeger D, et al. Cornelia de Lange syndrome is caused by mutations in NIPBL, the human homolog of Drosophila melanogaster Nipped-B. Nat Genet. 2004;36:631-5.

5. Tonkin ET, Wang TJ, Lisgo S, Bamshad MJ, Strachan T. NIPBL, encoding a homolog of fungal Scc2-type sister chromatid cohesion proteins and fly Nipped- B, is mutated in Cornelia de Lange syndrome. Nat Genet. 2004;36,636–641.

6. Musio A, Selicorni A, Focarelli ML, Gervasini C, Milani D, Russo S, et al. X- Linked Cornelia de Lange syndrome owing to SMC1L1 mutations. Nat Genet. 2006;38,528–530.

7 Deardorff MA, Kaur M, Yaeger D, Rampuria A, Korolev S, Pie J. Mutations in cohesin complex members SMC3 and SMC1A cause a mild variant of cornelia de Lange syndrome with predominant mental retardation. Am J Hum Genet. 2007;80:485-94.

8. Deardorff MA, Wilde JJ, Albrecht M, Dickinson E, Tennstedt S, Braunholz D, et al. RAD21 mutations cause a human cohesinopathy. Am J Hum Genet. 2012 ;90:1014-27.

9. Olley G, Ansari M, Bengani H, Grimes GR, Rhodes J, von Kriegsheim A, et al. BRD4 interacts with NIPBL and BRD4 is mutated in a Cornelia de Lange-like syndrome. Nat Genet. 2018;50:329-332

10. Deardorff MA, Bando M, Nakato R, Watrin E, Itoh T, Minamino, et al. HDAC8 mutations in Cornelia de Lange syndrome affect the cohesin acetylation cycle. Nature. 2012;489:313-7.

11. Huisman SA, Redeker, EJW, Maas SM, Mannens MM, Hennekam RC, et al. High rate of mosaicism in individuals with Cornelia de Lange syndrome. J Med Genet. 2013;50:339–344.

12. Saunier C, Støve SI, Popp B, Gérard B, Blenski M, AhMew N, et al. Expanding the phenotype associated with NAA10-Related N-Terminal Acetylation Deficiency. Hum Mutat. 2016;38:755-64.

13. Parenti I, Gervasini C, Pozojevic J, Graul-Neumann L, Azzollini J, Braunholz, et al. Broadening of cohesinopathies: exome sequencing identifies mutations in ANKRD11 in two patients with Cornelia de Lange-overlapping phenotype. Clin Genet. 2016;89:74-81

14. Woods SA, Robinson HB, Kohler LJ, Agamanolis D, Sterbenz G, Khalifa M. Exome sequencing identifies a novel EP300 frame shift mutation in a patient with features that overlap Cornelia de Lange syndrome. Am J Med Genet A. 2014;164:251-8.

15 Izumi K, Nakato R, Zhang Z, Edmondson AC, Noon S, Dulik MC, et al. Germline gain-of-function mutations in AFF4 cause a developmental syndrome functionally linking the super elongation complex and cohesin. Nat Genet. 2015;47:338-44.

16 .Kline AD, Moss JF, Selicorni A, Bisgaard AM, Deardorff MA, Gillett PM, et al. Diagnosis and management of Cornelia de Lange syndrome: first international consensus statement. Nat Rev Genet. 2018;19:649-666.

## A90 Immunotherapy for aeroallergens: when, how, where and why

### Alberto Martelli, Francesca Atzeri, Lorenza Serradori

#### Department of Pediatrics. G. Salvini Hospital. Garbagnate Milanese. Italy

##### **Correspondence:** Alberto Martelli (agmartelli@asst-rhodense.it)

Allergen Immunotherapy (AIT) is a proven therapeutic option for the treatment of allergic rhinitis and/or asthma. AIT is the only therapy that can change the natural history of the allergic disease and AIT is still the only treatment of pollen allergy, providing a long-term effect. Some obstacles still reduce its practical implementation. Among them, the understanding of the mode of action and dose effects, the difficulties of the administration routes and the lack of recognition of AIT as a drug. More effective preparations with faster onset and reduced doses are likely to improve compliance.

The decision to prescribe AIT for the child should be individualized and based on the relevance of the allergens, the persistence of symptoms despite appropriate medications according to guidelines as well as on the availability of good-quality and efficacious extracts. However, there are intercontinental differences in AIT therapeutic products in terms of their application and regulation.

The three major limitation of subcutaneous immunotherapy (SCIT) are the pain perception inherent to its route of administration, the fear of repeated injections particularly among young children, and the possibility of inoculating directly into the vein, with immediate and dramatic side effects. In adults, systemic reactions may happen in 2.4% to 50% of patients and 0.2% of injections. In children, lower rates have been reported. These were the main reasons for the development of specific immunotherapy by alternative ways, including epicutaneous and sublingual specific immunotherapy (SLIT). SLIT is burdened by poor compliance to treatment. Usually the severity/persistence of seasonal symptoms in subjects during allergen immunotherapy was noted to be much lower than in trials of pharmacotherapy. Needle-free injectors are not young anymore. Initially developed as an alternative to the use of syringes, they have been extensively utilized for mass immunizations. In recent years needle-free delivery technology has gained momentum in other clinical areas. This “liquid needle” has sufficient force to pass through the skin and enter the subcutaneous tissue. The device utilizes a low cost, single use disposable ampule that minimizes the potential for cross-contamination, a serious issue that has been observed with jet injectors equipped with multiuse components.

## A91 Pictures of lymphoadenomegalies

### Davide Massano, Elisa Carraro, Marta Pillon

#### Clinic of Hematology-Oncology, University-Hospital, Padova, 35128, Italy

##### **Correspondence:** Davide Massano (davide.massano@aopd.veneto.it)

**Background**

The word lymphadenopathy (LAP) identifies a condition in which lymph nodes are abnormal in size, number or consistency. The development of a cervical LAP in childhood is quite common, while the development of axillaries or inguinal LAP is unusual. Studies have estimated the incidence of cervical LAP ranged from 41% in the preschool age to 90% in children of 4 to 8 years old.[1]

Causes of LAP are explained by a wide number of aetiologies, that can be benign and self-resolving or malignant. Paediatricians must know the correct approach to LAP in order to optimise its pathway of diagnosis and treatment.

**Materials and methods**

A systematic review of the literature was performed searching on PubMed: "Lymphadenopathy"[Mesh] AND (Review[ptyp] AND ("infant"[MeSH Terms] OR "child"[MeSH Terms] OR "adolescent"[MeSH Terms])). The research was performed on the 12th May 2019. Known publications on the management of LAP were added, also if excluded by PubMed results.

**Results**

The literature research reported 20 articles; 3 of them were focused on LAP [1-3]. A further review was added to the analysis [4]. All the papers agree that the main cause of LAP is the reactive/infective aetiologies, while less frequent causes are chronic lymphadenitis, primitive tumour as lymphomas, and rarely are IgG4-related disease, Kikuchi-Fujimoto disease, lymphoproliferative syndrome, etc. The first evaluation of a LAP is the clinical history and physical examination. Some symptoms could be associated with a high suspicion of malignancy: non-tender, firm or hard lymph nodes, diameter >3 cm, supraclavicular localization, axillary or inguinal in the absence of skin changes, persistent or generalized form, systemic signs as persistent fever, itching, night sweat, weight loss, fatigue, petechiae or other hemorrhagic lesions, hepatosplenomegaly, symptoms related to mediastinal and/or abdominal masses.

Ultrasonography is the first radiological exam to perform if there is a chronic LAP or a suspicious of malignancy. Ultrasonography features that suggest a malignancy are: heterogeneity of the node, round shape, absent hilum, irregular borders, cystic necrosis, or irregular blood flow patterns to the capsule. Patients who continue to have persistent symptoms despite appropriate therapies, should require a biopsy. The fine needle aspiration biopsy is not recommended due to its low sensitivity.

**Conclusions**

Paediatricians meet patients with LAP throughout their career. A prompt identification of the signs and symptoms of malignancies have to lead the paediatrician to refer its patient in a second level hospital with a Paediatric Oncology and Surgery.

**References**

1. Weinstock MS, Patel NA, Smith LP. Pediatric cervical lymphadenopathy. Pediatr Rev. 2018;39:433-43.

2. King SK. Lateral neck lumps: A systematic approach for the general paediatrician. J Paediatr Child Health. 2017;53:1091-5.

3. Nightingale M. Midline cervical swellings: What a paediatrician needs to know. J Paediatr Child Health. 2017;53:1086-90.

4. Chiappini E, Camaioni A, Benazzo M, Biondi A, Bottero S, De Masi S, et al. Development of an algorithm for the management of cervical lymphadenopathy in children: consensus of the Italian Society of Preventive and Social Pediatrics, jointly with the Italian Society of Pediatric Infectious Diseases and the Italian Society of Pediatric Otorhinolaryngology. Expert Rev Anti Infect Ther. 2015;13:1557-67.

## A92 Translational research: a research loop between innovation and sustainability issues

### Nicolò Mauro^1,2^, Davide Vecchio^3^, Mara Utzeri^1^

#### ^1^Department of “Scienze Biologiche, Chimiche e Farmaceutiche”, University of Palermo, Palermo, Italy; ^2^ Fondazione Umberto Veronesi, Milan, Italy; ^3^ Medical Genetics Unit “ V. Cervello” Hospital, Palermo, Italy

##### **Correspondence:** Nicolò Mauro (nicolo.mauro@unipa.it)

Translational research (TR) encloses i) the process of applying discoveries arisen from basic research to the development of clinical trials and ii) the research which promotes the adoption of best clinical practice [1]. The main aim of TR is to integrate basic research (T1), patient-oriented research (T2) and population-oriented research (T3) in a multidisciplinary environment so as to improve the public health. The process is multidirectional, implying that new knowledge generated at each step could be applied in the other research phases to improve outcomes. The genome editing technology such as retroviral vectors and CRISPR-Cas9 system represents the most clamorous success of recent TR. For example, De Luca et al. have applied retroviral gene therapy and stem-cell technologies to treat patients affected by junctional epidermolysis bullosa (JEB). They developed a method to extract stem cells from patients skin, add functional genes in them and grow healthy skin on scaffolds in vitro. Thus, they successful implanted this genetically modified skin in JEB patients for the first clinical trial (2002), replacing sloughed-off skin on the legs of a patient [2]. This is a good example of TR, in which the clinical outcomes were based on decades of basic research, albeit clinical knowledge during the follow-up also generated further insights into human skin biology, such as on the self-renewal mechanism of epidermis, thus completing concepts of basic research. Another example is the combination of CRISPR/Cas9 technology as a genome editing tool and CAR-T cell therapy (engineered T cells that express chimeric antigen receptors), which recently leads to further improvement of leukemia immunotherapy. Despite only 7 years have passed since CRISPR-cas9 system was used in eukaryotic cells at Berkeley, results of recent trials have led to an unprecedented FDA approval of Yescarta® and Kymriah® for the treatment of children with refractory B-cell acute lymphoblastic leukemia. However, they are personalized therapies and thus too expensive (from $373,000 to $475,000) to be applicable in Europe to ensure access to these therapies for all patients under the current functioning of healthcare systems [3]. In contrast, reverse translation (RT) (from bedside-to-benchtop) is a new concept of research approach which allow minimizing failed clinical trials, thus providing only expected and unexpected results useful for further basic and clinical explorations. A classic example of RT is drug repurposing arisen from clinical observations of side effects unrelated to its primary therapeutic goal [4]. The repurposing of statins for kidney protection shows well the pivotal role of RT in uncovering mechanism of action and highlights how clinical trials may redirect basic research to new therapeutic targets, thus leading to cost-effective innovative therapies [4, 5].

**References**

1. McGartland Rubio D, Shoenbaum E E, Lee L S, Schteingart D E, Marantz P R, et al. Defining Translational Research:Implications for Training. Acad Med. 2010; 85:470-475.

2. Mavilio F, Pellegrini G, Ferrari S, Di Nunzio F, Di Iorio E, Recchia A, et al. Correction of junctional epidermolysis bullosa by transplantation of genetically modified epidermal stem cells. Nat Med. 2006; 12:1397-1402.

3. CAR-T Cell Therapies: How much for survival? Association of European Cancer Leagues www.europeancancerleagues.org/wp-content/uploads/2018/06/CAR-T-ECL-Article_Final_20062018.pdf Accessed 8 August 2019.

4. McWilliam S J, Antoine D J, Pirmohamed M. Repurposing statins for renal protection:is it a class effect?. Clin Transl Sci. 2018; 11:100-102.

5. Shakhnovich V. It’s time to reverse our thinking: the reverse translation research paradigm. Clin Transl Sci. 2018; 11:98-99.

## A93 Evaluation of a new method to distinguish viral and bacterial etiology in respiratory infections in pediatric age

### Elisabetta Mencaroni, Susanna Esposito

#### Clinica Pediatrica, Università degli Studi di Perugia, Azienda Ospedaliera Santa Maria della Misericordia, Perugia

##### **Correspondence:** Elisabetta Mencaroni (elisabetta.mencaroni@ospedale.perugia.it)

**Background**

Febrile respiratory infections (RTI) are the most frequent infections in childhood. Although more than 80% of RTIs recognize a viral etiology, clinical features and common laboratory tests, especially in the beginning, may fail to discriminate a bacterial or viral origin, leading to an inappropriate use of antibiotics and the development of bacterial resistances. “AutoPilot-Dx” (NCT03052088) is an EU funded, multinational, prospective study designed to assess sensitivity and specificity of the ImmunoXpert™ method in differentiating viral or bacterial etiology of RTIs in children, evaluating three biomarkers: C-reactive protein (CRP), TNF-related apoptosis-inducing ligand (TRAIL) and interferon-γ-induced protein 10 (IP-10).

**Materials and methods**

Children aged between 30 days and 17 years, with fever >38° C for less than 7 days, not treated with antibiotics for more than 48 hours, without clinical signs of localization, were included in the study. Each child was assessed for "ImmunoXpert™", and for nasal and nasopharyngeal swab with polymerase chain reaction for viruses and bacteria. ImmunoXpert™ provided an overall score between 0 and 100, suggesting bacterial (100-65), viral (0-35) or indeterminate (36-64) etiology. Clinical features and the results of swabs were also evaluated by a blinded team of experts, which proposed an etiological suspicion.

**Results**

Of the 657 enrolled children, 465 were classified as viral. Of them, 317 and 57 were defined as having non-severe and severe viral disease, respectively. TRAIL levels were markedly increased in all viral patients 171±136 vs 68 ± 27 pg/mL; p<0.001). Patients with severe viral infection exhibited significantly lower TRAIL levels as compared to non-severe infection (non-severe 158±114; severe 107±60; p=0.001), implying that viral-induced TRAIL levels may be counterbalanced by the severity state. Age and time from fever onset were not found to be confounders. IP-10 levels were significantly higher in viral than in the bacterial infections (22.2±28.5 vs 62.4±31.0; p=0.003). Notably, common bacterial markers such as CRP, white blood cell counts and neutrophil counts exhibited no significant difference between viral and bacterial groups. ImmunoXpert™ showed a sensitivity of 96.1% and a specificity of 95.3% for the identification of viral etiology.

**Conclusions**

ImmunoXpert™ has a sensitivity and specificity in discriminating viral infections higher than that of many laboratory tests, common anamnestic parameters and clinical objectivity, suggesting its potential for reducing abuse of antibiotics.

## A94 Hemophagocytic lymphohistiocytosis and macrophage activation syndrome

### Francesca Minoia (francesca.minoia@hotmail.com)

#### Fondazione IRCCS Ca’ Granda Ospedale Maggiore Policlinico, Milano, Italy

Hemophagocytic lymphohistiocytosis (HLH) is a life-threatening hyperinflammatory syndrome caused by a severely dysregulated immune response. It is characterized by highly activated lymphocytes and macrophages that infiltrate tissues and produce large amounts of proinflammatory cytokines. A set of clinical, laboratory, and histopathological features define the acute syndrome, including unremitting fever, hepatosplenomegaly, cytopenia, hypofibrinogenemia, elevated ferritin, liver enzymes, triglycerides and hemophagocytosis. HLH comprises a heterogeneous spectrum of clinically similar, but etiologically diverse subtypes, affecting all ages. In the current classification, it is subdivided into primary and secondary forms. Primary HLH (pHLH) refers to cases associated with several inherited monogenic disorders. Secondary HLH (sHLH) complicates various medical conditions, including infections, malignancies, and rheumatic diseases. By convention, sHLH seen in rheumatic disorders is termed macrophage activation syndrome (MAS). In childhood, this condition occurs most commonly in systemic juvenile idiopathic arthritis (sJIA). Primary HLH and MAS share many similarities in their genetic background, pathogenic and cytokine pathway, clinical and laboratory features. However MAS develops in the context of an underlying highly inflammatory condition, while pHLH is a primary disorder with genetic basis. It is, thus, conceivable, that other contributors might be involved to the development of sHLH, including MAS, such as infections or underlying inflammation. Hemophagocytic syndromes are potentially fatal and require immediate recognition to initiate prompt treatment. While diagnosis of pHLH is based on HLH-2004 criteria definitely confirmed by genetic results, sHLH and especially MAS diagnosis is often challenging, due to the lack of a single pathognomonic marker and to the overlap with other confoundable conditions, such as sepsis or flare of underlying disease. Classification criteria for MAS complicating sJIA were published in 2016; however no universally embraced diagnostic criteria for MAS in other condition are available so far. Furthermore, the discrimination between MAS and pHLH might be difficult, especially when MAS develops as the first manifestation of a rheumatic disease.

Because no controlled studies on the treatment of MAS are available, the management of this condition is largely empiric. The mainstay of the therapy is constituted by high doses of corticosteroids. Cyclosporine-A is considered as adjunctive first-line treatment in MAS, due to its role in pHLH treatment and to numerous reports of marked efficacy. In the last years increasing evidence supports the use of anakinra in the treatment of MAS associated with sJIA. Recently, the dramatic effect of the anti INF-gamma in pHLH led to plan a trial in sHLH with promising results.

## A95 Therapeutic strategy in pediatric rheumatology: what we learnt form the past and what is new

### Francesca Minoia (francesca.minoia@hotmail.com)

#### Fondazione IRCCS Ca’ Granda Ospedale Maggiore Policlinico, Milano, Italy

Over the past two decades, the treatment of pediatric rheumatology diseases has been revolutionized by the increased tendency toward early aggressive interventions and the availability of the novel biologic medications. Focusing on juvenile idiopathic arthritis (JIA), the most common rheumatic disease in childhood and the filed in which the greatest efforts in therapeutic trials were made, we analyse the multiple steps done in the last twenty years in the management of rheumatic diseases. Since the introduction of methotrexate in the early ‘90s, the treatment of JIA was dramatically improved by the availability of biologic medications, especially anti-tumor necrosis factor (TNF) in polyarticular JIA and anti interleukin-1 and interleukin-6 in systemic juvenile idiopathic arthritis. Intrarticular corticosteroid injections (IACI) revolutionized not only the treatment of oligoarticular JIA, but also significantly reduced the use of systemic corticosteroid therapy even in polyarticular JIA patient, with a pivotal effect in limiting treatment toxicity. A more rational approach to the management of JIA is being fostered by the recent publication of therapeutic recommendations, consensus treatment plans, and advice for the optimal care. Due to the increasing potential of attaining inactive disease in children with JIA, there is an urgent need for randomized controlled trials, analyses of clinical data sets, and expert advice to guide discontinuation of medications once complete disease quiescence has been achieved. Other open issues to address include: identification of pathways biomarkers to better personalize treatment of different diseases, comparison of different strategies in order to minimize treatment toxicity, increasing international accessibility of standardized care and implementation of international registry to carefully monitor long term safety of treatments.

## A96 Human social genomics

### Manuela Monti^1^, CarloAlberto Redi ^2^

#### ^1^Centro Ricerche Medicina Rigenerativa – Fondazione IRCCS Policlinico San Matteo – Pavia, Italia; ^2^Dipartimento Biologia e Biotecnologie – Università di Pavia – Pavia, Italia

##### **Correspondence:** Manuela Monti (m.monti@smatteo.pv.it)

The great pathologist and statesman Rudolf Virchow (1821 - 1902) already suggested the existence of a socio-biological transition,that is how our body (i.e. cells and the DNA) is affected by the way we lead our lives. In other words, the way good and/or bad emotions, nutrition and pollutants are epigenetically recorded by our cells throughout our entire life from the zygote to embryo to fetus and adult life [1]. Germ cells are particularly susceptible to the socio-biological transition and they are able to transfer the epigenetic marks to the future generations. In particular, mother-fetus interactions can assign prospective negative health predictors to the newborns depending on the mother’s and father’s socio-economic status [2].

Here we present some examples emphasizing the role of the current socio-economic inequalities as predictors of health outcomes.

**References**

1. Aizer A. Currie J. The intergenerational transmission of inequality: Maternal disadvantage and health at birth. Science 2014;344:856-861.

2. Underwood E. Can disparities be deadly? Science 2014;344:829-831.

## A97 Point of care ultrasound in the emergency department

### Anna Maria Musolino (annamaria.musolino@opbg.net)

#### Pediatric Emergency Department, Bambino Gesù Children’s Hospital, Rome, Italy

Point-of-care ultrasonography (POCUS) is a safe, non-invasive and effective diagnostic or procedural guidance ultrasound [1] performed by a clinician to help guide the evaluation and management of the patient, complementary to a medical examination, introduced by the American College of Emergency Medicine (ACEP) in the 90s. [2]. It can be used in hospital and non-hospital settings to exclude severe pathological findings, to increase clinical diagnostic suspicions and to monitor therapy, using a portable ultrasound system at the “point of care” of the patients and reducing at the same time the risks associated with ionizing radiations [3].

Applications. POCUS is largely applied for evaluating pediatric respiratory symptoms identifying pneumonias (sensitivity of 96% and a specificity of 93%) [4], pleural effusions and distinguishing between parapneumonic pleural effusion and empyema. It can be used in the management of children with bronchiolitis [5] [6] It has also demonstrated 100% accuracy in diagnosing pneumothoraces and useful to guide needle aspiration [7,8]. Focused cardiac ultrasound should be considered for assessing patients with hemodynamic failure; it enables physicians to diagnose pericardial effusions, assess cardiac contractility and left ventricular enlargement with 91% accuracy. [9] It is useful even during pulse checks during cardiac arrest resuscitation [10] and for categorization of shock state and initial management strategy [11]. Several protocols have been created to a global approach to a critical patient. Rapid Ultrasound for Shock and Hypotension (RUSH) exam includes standardized views of the heart, inferior vena cava, lungs and abdomen in order to categorize the type of shock. [3] Focused Assessment with Sonography in Trauma (FAST) [12] is applied to assess identification of free fluid at the initial evaluation of the patient with thoracic or abdominal trauma. It allows to check hemothorax, pericardial effusion and tamponade, and to identify an intra-abdominal source of bleeding [11] .The E-FAST (Extended FAST) allows to check for pneumothorax.

Training. Physician must be trained and assessed for competency (understanding the clinical indications, having the technical skill for image acquisition, the ability to interpret the images and to integrate the findings clinically) in order to be credentialed (through local criteria required to utilize POCUS in their clinical practice) and thus granted privileges by an institution to perform POCUS [13]. Pathways for obtaining basic POCUS competency are training based and practice-based [14]. It’s mandatory maintaining competency participating in CME (Continuing Medical Education) as well as perform a minimum number of POCUS examinations annually [13].

**References**

1. Le Coz J, Orlandini S, Titomanlio L, Rinaldi VE. Point of care ultrasonography in the pediatric emergency department. Ital J Pediatr 2018;44:87.

2. American College of Emergency Physicians. Council resolution on ultrasound. ACEP News. 1990;9.

3. McLario DJ, Sivitz AB. Point-of-Care Ultrasound in Pediatric Clinical Care. JAMA Pediatr 2015;169:594-600.

4. Pereda MA, Chavez MA, Hooper-Miele CC, Gilman RH, Steinhoff MC, Ellington LE, et al. Lung ultrasound for the diagnosis of pneumonia in children: a meta-analysis. Pediatrics 2015;135:714–722.

5. Supino MC, Buonsenso D, Scateni S, Scialanga B, Bock C, Chiaretti A. Point-of-care lung ultrasound in infants with bronchiolitis in the pediatric emergency department: a prospective study. Eur J Pediatr. 2019;178:623-632

6. Hajalioghli P, Nemati M, Dinprast Saleh L, Fouladi DF. Can chest computed tomography be replaced by lung ultrasonography with or without plain chest radiography in pediatric pneumonia? J Thorac Imaging 2016;31:247–252.

7. Raimondi F, Rodriguez Fanjul J, Aversa S, Chirico G, Yousef N, De Luca D. Lung Ultrasound in the Crashing Infant (LUCI) Protocol Study Group. Lung ultrasound for diagnosing pneumothorax in the critically ill neonate. J Pediatr 2016;175:74–78.

8. Migliaro F, Sodano A, Capasso L, Raimondi F. Lung ultrasound- guided emergency pneumothorax needle aspiration in a very preterm infant. BMJ Case Rep 2014;14:2014.

9. Spurney CF, Sable CA, Berger JT, Martin GR. Use of a hand carried ultrasound device by critical care physicians for the diagnosis of pericardial effusions, decreased cardiac function, and left ventricular enlargement in pediatric patients. J Am Soc Echocardiogr 2005;18:313–319.

10. Gaspari R, Weekes A, Adhikari S, Noble VE, Nomura JT, Theodoro D, et al. Emergency department point-of-care ultrasound in out-of-hospital and in-ED cardiac arrest. Resuscitation 2016;109:33-39.

11. Whitson MR, Mayo PH. Ultrasonography in the Emergency Department. Crit Care 2016;20:227.

12. Richards JR, McGahan JP. Focused assessment with sonography in trauma (FAST) in 2017: What radiologists can learn. Radiology 2017;283:30-48.

13. Abo AM, Alade KH, Rempell RG, Kessler D, Fisher JW, Lewiss RE, Raio CC, Marin JR. Credentialing pediatric emergency medicine faculty in point-of-care ultrasound. Pediatric Emer Care 2019. [Epub ahead of print]

14. Marin JR, Lewiss RE, American Academy of Pediatrics, Committee on Pediatric emergency Medicine; Society for Academic Emergency Medicine, Academy of Emergency Ultrasound; American College of Emergency Physicians, Pediatric Emergency Medicine Committee, et al. Point-of-Care ultrasonography by pediatric emergency medicine physicians. Pediatrics 2015;135:1113-22.

## A98 Adverse reactions following immunization and contraindications

### Luciana Nicolosi (luciana.nicolosi@opbg.net)

#### UOC Paediatrics and Infectious Diseases, Bambino Gesù Children’s Hospital, IRCCS, Rome, Italy

##### **Correspondence:** Manuela Monti (m.monti@smatteo.pv.it)

An adverse event following immunization (AEFI) is defined as any untoward medical occurrence which follows immunization and which does not necessarily have a causal relationship with the use of the vaccine. The adverse event may be any unfavourable or unintended sign, an abnormal laboratory finding, a symptom or a disease. An AEFI is defined as serious if it results in one of the following outcomes: death, hospitalization or extension of a pre-existing hospitalization, persistent or severe disability, congenital malformation.

Causality assessment usually will not prove or disprove an association between an event and the immunization. It is meant to assist in determining the level of certainty of such an association. A definite causal association or absence of association often cannot be established for an individual event. In 2013 the WHO published the User Manual for the revised classification on causality assessment of adverse events following immunizations, which aims to standardize the process of attribution of the causal link between adverse events and vaccines.

In AIFA’s Vaccine Report 2017, accounts of suspected adverse reactions in 2017 have disclosed an increasing trend, on one hand suggesting a general improvement in the performance of the vaccinovigilance system in Italy on the other hand hinting at possible external factors that might have influenced the progress of reports, referring to the new vaccination legislation. In fact, 57% of the reports for all vaccines were received from "another healthcare provider", with those coming from citizens or patients exceeding those coming from doctors. The in-depth analysis of the cases at the level of the individual report and the general trend do NOT SUGGEST, in fact, the presence of additional risks beyond those known that could modify the benefit / risk ratio of the vaccines used.

Recognizing and discriminating adverse reactions to vaccines presupposes a background of knowledge on the disease observed, on the type of vaccine administered, on the plausible timing for the presentation of that specific reaction with that specific vaccine. Once an adverse reaction has been established, it remains to be determined if in the particular case the picture observed can be correlated to the vaccination, taking into account the presence or absence of alternative causes.

From the overall results of the analyses conducted for type of vaccine in 2017 no possible association between suspected additional risks and vaccines emerged at the moment and therefore, no safety signals that may elicit a vaccine safety alert.

## A99 Role of the hospital pediatrician in vaccination

### Luciana Nicolosi (luciana.nicolosi@opbg.net)

#### UOC Paediatrics and Infectious Diseases, Bambino Gesù Children’s Hospital, IRCCS, Rome, Italy

The task of the Hospital Pediatrician, with regard to vaccinations, is essentially to adapt the vaccination calendar to the various situations known as "at risk", including both the "allergy risk" in subjects suffering from severe forms of allergies and / or adverse reactions to previous vaccine doses, and the "risk of complications of vaccine-preventable infectious diseases" that may occur more frequently in subjects with chronic diseases and / or worsen the prognosis of the underlying disease. In specific situations, therefore, both physiological and pathological, vaccinations must be carried out taking precautions that require special knowledge, adapting the vaccination calendar to the subject, based on the specific problem.

In a hospital setting, frequently encountered situations could include:

1. Premature babies
2. Patients treated with immunoglobulins or other blood products
3. "Non-responder" subjects
4. Alterations in the immune system
5. Chronic conditions
6. Pre- and post-solid organ transplantation
7. Pre- and post-bone marrow transplantation
8. Anatomic or functional asplenia
9. Allergic patients and / or with a previous vaccine dose reaction

Anyhow, reference to true and false contraindications to vaccinations and to precautionary measures will be most helpful in choosing the vaccination to be administered.

The priority to be given and the timing of the vaccination in each specific patient is essential in choosing the vaccine in various types of chronic disease; this may require to anticipate the minimum age set by the calendar for that given vaccine, applying instead the lowest possible age in which to carry out that particular vaccination; it may also be important to adapt the interval between subsequent doses to the shortest possible interval between doses, for a given vaccine. This requires using specific tables showing both the minimum age at which the child can be vaccinated and the minimum interval between doses. On the other hand, for those children with a history of “IgE mediated” allergic reaction to a previous vaccine dose, it is possible to adopt the vaccination method based on “desensitization”. In a nutshell, although the vaccination calendar remains an element of constant reference in the active immunization of the population, it must be considered an agile and dynamic tool adaptable to each particular situation, allowing, in most cases, to direct the choices of health care professionals in the irreplaceable role of protecting the health of the developing child through vaccination.

## A100 Hemoglobinopathies

### Agostino Nocerino (agostino.nocerino@uniud.it)

#### ASUI Udine, Italy

Anemias represent an important burden disease in migrant children, and therefore 2012 CDC guidelines recommend routinary screening for anemia. The highest prevalence rate is observed in Africa (62%), but the majority of cases are in South-East Asia and in Eastern Mediterranean (WHO 2015). Iron-Deficiency Anemia represents the most common cause of anemia in immigrant children, but hemoglobinopathies are also frequent. Thalassemias are very well known in Italy, but many hemoglobinopathies have become frequent only with the arrival of immigrants from other countries.

One of the best known of these conditions is sickle cell disease (SDC) in its main forms SS, S-β0, Sβ+ and SC, common in children originating in central Africa, but also from other countries (as Middle East). SDC is characterized by very severe vaso-occlusive painful crises, but also acute thoracic syndrome, stroke, aplastic crises, and very serious infections (especially in the first 5 years of life) requiring antibiotic prophylaxis after the second month of life, mainly in SS and S-β0 genotypes. Priapism is quite common but very often misunderstood by the affected subjects themselves. Please refer to the AIEOP Guidelines for further details. HbE disease is frequent in some groups of immigrants, particularly in those from Southeast Asia. Both heterozygotes and homozygotes subjects are asymptomatic, but can have hypochromic microcytosis and mild anemia. However, in carriers of both the hemoglobin E gene and beta-thalassemia severe anemia may result.

HbC disease is frequent in people originary from West Atlantic Africa. The anemia is mycrocitic, usually mild or moderate, with a slight degree of hemolysis, sometimes with splenomegaly.

The high frequency of hemoglobinopathies in migrant children makes an early diagnosis essential either by a simple blood cells count (CBC) as already indicated in the National Guidelines “Salute Migranti”) or by a more sensitive globin chain analysis. Recently, low-cost, rapid and easy diagnostic tests by finger sticks for SCD and other hemoglobinopathies have been developed, making the diagnosis faster and cheaper. Currently the estimated sensitivity is estimated to be up to 97.6% for HbSS detection, but further studies are needed. For children born in Italy, neonatal screening is strongly recommended and it is now widespread in different reference centers. Currently, however, a regional neonatal screening is present only in Friuli Venezia Giulia for newborns considered at risk. In Udine since October 2018 a screening with CBC, high-performance liquid chromatography, and genetic studies in selected cases is performed in all newborns.

**Acknowledgments**

I thank Ilaria Liguoro for collaboration.

## A101 The workflow in a home telemonitoring project for cystic fibrosis patients: the role of the nurse

### Clarissa Paglia, Bella Sergio

#### Cystic Fibrosis Unit, Bambino Gesù Children’s Hospital Rome, Italy

##### **Correspondence:** Clarissa Paglia (clarissa.paglia@opbg.net)

Cystic Fibrosis (CF) is the most common genetic disease with an ominous outcome that affects the Caucasian population. Classically, the CF is known as a respiratory disease, where it causes repeated infections and progressive bronchiectasis formations until the development of respiratory failure. Respiratory exacerbations are a critical event for the CF patient, leading to a deterioration in quality of life, increasing lung damage, costs and intensity of care. Continuous monitoring of the patient's clinical situation helps to prevent and / or minimize exacerbations, through a timely recognition of symptoms of exacerbation, with positive consequences for the patient's life expectancy and the rationalization of hospital admissions. Since 2001, at the Center for Cystic Fibrosis of the Bambino Gesù Children’s Hospital I.R.C.C.S. in Rome, we apply the telemonitoring of respiratory parameters in the follow-up of patients at home. From February 2018 a nurse assists the doctor in the management of the service. Currently, around 40 patients are followed in the project, ranging in age from 11 to 50, from central-southern Italy and the major islands. The responsible nurse collects, analyzes, evaluates the transmissions of spirometric data, which the patients perform weekly from home and send to a dedicated platform. Together with other parameters (SpO2, nocturnal saturimetry, heart rate, PA, blood glucose and body weight) makes an assessment of the patient's clinical status, which also occurs through the responses of the same to a questionnaire on subjective symptoms (shortness of breath for small 'sputum, increased sputum,etc.). The interview with the doctor allows the nurse to discuss "the assistance plan", on the basis of which they decide the therapeutic interventions that will be communicated to the patient, or to the parents, through a telephone interview. The virtual contact with the patients is the fulcrum of the intervention of the responsible nurse, who has the possibility to evaluate their care needs, to request further data transmissions or to call to hospital for assessments and treatment under Ambulatory, Day Hospital or Ordinary Hospitalization. The nurse is also responsible for monitoring the proper functioning of the instruments used by the patients, dealing with the calibration of the spirometer, the updating of the indicators on which the measurements of FEV1 (weight and height) and of the instrument's software are based, interfacing with the company responsible for the assistance of the same.

## A102 Allergen Specific Immunotherapy: the time is ripe for no more doubts

### Giovanni B. Pajno, Andrea Barbalace, Stefano Passanisi, Stefania Arasi,Tommaso Aversa, Giuseppina Zirilli, Lucia Caminiti

#### Department of Pediatrics, Allergy Unit, University of Messina, Messina, Italy

##### **Correspondence:** Giovanni B. Pajno (giovanni.pajno@unime.it)

During the last few decades there has been an increase in the prevalence of allergic diseases and it has been predicted that by 2025, half of entire EU population will be affected [1]. The treatment of allergic respiratory diseases is based on allergen avoidance, pharmacotherapy, allergen immunotherapy and patient education. Vaccines are utilized in medicine as immune modifiers. So too is allergen specific immunotherapy (AIT). Therefore, in contrast to symptom control by pharmacotherapy: AIT aims to modify the immune system via tolerance induction [2], and is potentially able to alter the course of allergic disease [3,4]. A significant body of data accumulated regarding the efficacy and safety of AIT vaccines in adults and in children both for respiratory allergy and for venom hypersensitivity and recent data look promising also for IgE-mediated food allergy. However AIT for many reason remains underused. The European Academy Allergy and Clinical Immunology (EAACI) guidelines provide a series of recommendations and also the clinical relevant steps to be followed to move AIT for major allergic diseases closer to being embraced by the medical community [5-9]. The appraisal of AIT in allergic children was reported by Società Italiana di Allergologia ed Immunologia Pediatrica (SIAIP) [4]. In order to achieve successful results it is important to begin AIT in childhood when bronchial asthma and other allergic comorbidities (i.e rhinitis) are often less severe than in adults and children show usually one or few sensitizations [10]. In this context pediatricians should know the local and regional aerobiology and be aware of the potential allergens in the patient’s indoor and outdoor environments.

Currently the efficacy of AIT is no longer considered as a generic “class effect” and each AIT product is evaluated according to its scientific evidence. This led to the approval of single AIT preparation by European (EMA) and American (FDA) medical regulatory agencies [9,11]. Relief of allergic symptoms and long-lasting efficacy represent two goals that can be attained in allergic children treated with AIT. Clinical principles of allergen specific immunotherapy represent the key concepts in order to implement a good clinical practice. (Table1).

These effects are of particular relevance in pediatric patients: in whom the careful selection of allergic children is the most important question to by faced for suppressing or reducing allergic symptoms through modification of antibody responses, lymphocytes responses, and other target cell responses to allergen.

**References**

1. Agache I. EAACI guidelines on allergen immunotherapy-Out with the old and in with the new. Allergy. 2018;73:737-738.

2. Jutel M, Agache I, Bonini S, Burks AW, Calderon M, Canonica W et al. International Consensus on Allergen Immunotherapy II: Mechanisms, standardization, and pharmacoeconomics. J Allergy Clin Immunol. 2016;137:358-68.

3. Dhuram SR, Emminger W, Kapp A, Colombo G, de Monchy JG, Rak S et al. Long-term clinical efficacy in grass pollen-induced rhinoconjunctivitis after treatment with SQ-standardized grass allergy immunotherapy tablet. J Allergy Clin Immunol. 2010;125:131-8.e1-7.

4. Pajno GB, Bernardini R, Peroni D, Arasi S, Martelli A, Landi M et al. Clinical practice recommendations for allergen-specific immunotherapy in children: the Italian consensus report. Ital J Pediatr. 2017;43:13.

5. Halken S, Larenas-Linnemann D, Roberts G, Calderón MA, Angier E, Pfaar O et al. EAACI guidelines on allergen immunotherapy: Prevention of allergy. Pediatr Allergy Immunol. 2017;28:728-745.

6. Sturm GJ, Varga EM, Roberts G, Mosbech H, Bilò MB, Akdis CA et al. EAACI guidelines on allergen immunotherapy: Hymenoptera venom allergy. Allergy. 2018;73:744-764.

7. Roberts G, Pfaar O, Akdis CA, Ansotegui IJ, Durham SR, Gerth van Wijk R et al. EAACI Guidelines on Allergen Immunotherapy: Allergic rhinoconjunctivitis. Allergy. 2018;73:765-798.

8. Pajno GB, Fernandez-Rivas M, Arasi S, Roberts G, Akdis CA, Alvaro-Lozano M et al. EAACI Guidelines on allergen immunotherapy: IgE-mediated food allergy. Allergy. 2018;73:799-815.

9. Bonertz A, Roberts GC, Hoefnagel M, Timon M, Slater JE, Rabin RL et al. Challenges in the implementation of EAACI guidelines on allergen immunotherapy: A global perspective on the regulation of allergen products. Allergy. 2018;73:64-76.

10. Pajno GB. Allergen immunotherapy in early childhood: between Scylla and Charybdis! Clin Exp Allergy. 2005;35:551-3.

11. Ryan D, Gerth van Wijk R, Angier E, Kristiansen M, Zaman H, Sheikh A et al. Challenges in the implementation of the EAACI AIT guidelines: A situational analysis of current provision of allergen immunotherapy. Allergy. 2018;73:827-836.

**Table 1 (abstract A102).** Clinical principles of allergen specific immunotherapy

CLINICAL PRINCIPLES OF ALLERGEN SPECIFIC IMMUNOTHERAPY	
Immunotherapy is effective only in IgE-mediated diseases such as allergic rhinitis, allergic asthma and stinging insect anaphylaxis.	
Successful therapy require years to reach maximal benefit. Efficacy is dose dependent .	
Either sublingual route (SLIT) and subcutaneous route(SCIT) could be used in clinical practice.	
Allergen(s) in the patient’s indoor and outdoor environments could display local and regional difference.	
Risk factors for systemic reaction such as of uncontrolled or severe asthma or high allergen exposure need to be considered.	

## A103 Imaging in haemangioma study

### Guglielmo Paolantonio^1^, George K. Parapatt^1^, Maya El Hachem^2^, Massimo Rollo ^1^, Paolo Tomà ^1^

#### ^1^Department of Imaging, Bambino Gesù Children’s Hospital IRCCS, Piazza S. Onofrio n. 4, 00165 Rome, Italy; ^2^Dermatology Unit, Bambino Gesù Children's Hospital, IRCCS, Piazza Sant'Onofrio, 4, 00165, Rome, Italy

##### **Correspondence:** Guglielmo Paolantonio (guglielmo.paolantonio@opbg.net)

Vascular anomalies represent the most common cause of pediatric soft-tissue masses and encompass a broad and heterogeneous spectrum of lesions representing a diagnostic and therapeutic challenge. Particularly, the infantile haemangioma is the most common vascular anomaly encountered in children. Despite the International Society for the Study of Vascular Anomalies (ISSVA) classification has expanded over the years and is now widely adopted, haemangioma and vascular anomalies may be still misunderstood and inaccurate nomenclatures keep on creating considerable confusion in the clinical practice. Unfortunately, the term “haemangioma” is still generally applied to denote any vascular anomaly. Since the treatment strategy depends on the type of vascular lesion, correct radiological assessment is therefore crucial. Imaging plays a pivotal role in the differential diagnosis between the vascular masses in children. Gray-scale ultrasonography (US) combined with color-Doppler US represent the first modality of choice for the initial assessment and characterization of a vascular lesion, usually allowing differentiation between hemangiomas and other vascular anomalies. Magnetic Resonance (MR) imaging coupled with MR angiography is the second level method useful to evaluate deeper lesions and their relationship to adjacent structures and is mandatory for the uncertain cases and in the assessment of syndromes associated with vascular tumors. Imaging findings of infantile haemangiomas and differential diagnosis with other vascular anomalies will be discussed.

## A104 Clinical costing and pediatric hospital

### Alberto Pasdera (studiopasdera@gmail.com)

#### Coordinatore scientifico del N. I. San (Network Italiano Sanitario), Mirano, Italy

The Association of Italian Paediatric Hospitals (AOPI) has carried out a project for the realization of the standard costs of paediatric hospital admissions, respecting the international requirements of the Clinical Costing. The standard costs were divided by production factor / activity, type of hospitalization (ordinary, outliers, 0-1 days, day hospital), DRG, age group (0-28 days; 29 days-365 days). ;> 365 days- <6 years;> 5 years- <18 years). The project focused on hospital discharges in the period 1 January-31 December 2016 of 12 hospital companies. The "size" of the activity examined was the following: 124,681 resignations for acute cases (ordinary admissions, outliers, 0-1 days); 199,611 days of access for day hospital / day surgery; 2.219 Short remarks. By "size" the database of paediatric DRG research is the second most important in Europe (after Germany), among those that respect the dictates of Clinical Costing. With reference to the results of the research, with particular reference to the comparison with the tariffs, it is known that these do not cover costs; but this is not the only problem. The real problem lies in the fact that the current tariff system is inconsistent with the reality of the costs to be borne by patients. The study found that the fee for hospitalization as a whole "covers" 63% of the costs actually incurred. However, if we analyse the data more deeply, we find that the tariffs do not "affect" the different types of hospitalization in the same way. In fact, while for day hospitals the average rates cover 45% of costs; for other types of hospitalization the situation is quite different. For example, for outliers (the usually "longer" and complex hospitalizations and with the highest number of hospitalization days in percentage in intensive care), the rates reach less than one-third of the costs (32%).

## A105 Endocrine consequences in cancer survivors

### Stefania Pedicelli (stefania.pedicelli@gmail.com)

#### Endocrinology Unit, “Bambino Gesù” Children’s Hospital, IRCCS, Rome, Italy

The continuous improvement in treatment and care of childhood cancer has led to increased long-term survival rates. This has driven the attention to long-term endocrine consequences. Both treatment and neoplasms may affect several endocrine glands (pituitary, thyroid, gonads, etc), leading to growth failure, hypothyroidism, adrenal insufficiency, impaired puberty and fertility, metabolic disorders and/or reduced bone density. Recent data show that 40% to 50% of childhood cancer survivors will develop at least one endocrine disorder over the course of their lifetime. Therefore, the endocrine monitoring plays a crucial role for early identification and treatment, especially for younger patients who underwent radiotherapy and/or therapy with alkylating agents. Growth hormone (GH) replacement therapy raises the issue of safety and data about the risk of second neoplasia are still debated, whereas the risk of primary cancer relapse seems to be not increased. Particular attention has been paid in the last years on strategies to preserve fertility. In conclusion, the follow-up of childhood cancer survivors requires a multidisciplinary approach, and, considering that endocrine disorders may occur many years after the diagnosis and/or the end of treatment, long-term endocrine surveillance is needed.

## A106 Children’s physical activity and air pollution

### Diego G Peroni^1^, Giulia Nuzzi^1^, Irene Trambusti^1^, Maria E Di Cicco, Giuliana Ferrante^2^, Giovanna Cilluffo^3^, Stefania La Grutta^3^

#### ^1^UO Pediatria Universitaria AOUP, Università di Pisa, Scuola di Specializzazione in Pediatria, Università di Pisa, Pisa, Italy; ^2^Dipartimento di Scienze della Promozione della Salute, Materno-Infantile, Medicina Interna e Specialistica d'Eccellenza, Università di Palermo, Palermo, Italy; ^3^Istituto di Biomedicina e Immunologia Molecolare (IBIM), Consiglio Nazionale delle Ricerche (CNR), Palermo, Italy

##### **Correspondence:** Diego G Peroni (diego.peroni@unipi.it)

There is now ample evidence that physical activity is an integral part of a healthy lifestyle as it contributes to a significant reduction in the risk of developing chronic diseases and improves mental health and wellbeing. Education for regular movement and healthy eating helps children to grow in good health and to prevent the problem of overweight, obesity and numerous diseases of young and adult age. As suggested by the American Academy of Pediatrics, children should practice at least 60 minutes of diversified and fun-filled physical activity per day. There is no better sport than another: it is important that physical activity is varied and fun, preferably in the open air, although exercising outdoors can expose children and adolescents to air pollution that can have adverse effects especially on respiratory health. The main air pollutants which can damage airways are: benzene, sulfur dioxide (SO_2_), particulate matter with an aerodynamic diameter of less than 2,5 microm (PM_2.5_ ) and less than 10 microm (PM_10_), ozone (O3), nitrogen dioxide (NO_2_) and carbon monoxide (CO). Similarly to outdoor pollutants, indoor pollution is a problem of considerable relevance for respiratory health in developmental age, in consideration of the fact that children spend more than 90% of their time indoors. Several studies suggested an association between increment of indoor concentrations of PM_10_ and PM_2.5_ and increased incidence of asthma and exertion asthma symptoms. In this context the pediatrician plays a role of primary importance not only as responsible for the health of the child but also as an educator and promoter of a healthy lifestyle. This report summarizes the latest scientific knowledge on the link between exposure to air pollution and adverse health effects in children and aims to underscore the importance to achieve the involvement of the global health community, in particular health authorities, in order to reduce the burden of air pollution with the ultimate goal of promoting children’s health and climate benefits.

## A107 Congenital erythrocytosis

### Silverio Perrotta (silverio.perrotta@unicampania.it)

#### Dipartimento della Donna, del bambino e di Chirurgia Generale e Specialistica, Università degli Studi della Campania “L. Vanvitelli”, Naples, Italy

Erythrocytosis is characterized by an increase in the red blood cell mass defined by hemoglobin and hematocrit values above 97th percentile for age and sex. It can be due to physiological processes, as high altitude living, pathological condition, as dehydration, or personal habits, as smoking. These conditions, as well as iron deficiency, a potential confounding factor, have to be ruled out before any additional examination for absolute erythrocytosis. First line exams include Oxygen Saturation evaluation, venous blood gas analysis with p50 measurement and plasmatic erythropoietin (Epo) dosage, whose results may guide the molecular screening. Low plasmatic levels of Epo are typical of the Primary erythrocytosis, showing endogenous erythroid colony formation in vitro, as the Familial erythrocytosis type 1 or the Polycythemia Vera. High, or inappropriately normal, Epo values are a feature of the erythrocytosis due to a defective Oxygen Sensing mechanism or to abnormal hemoglobin with high oxygen affinity (1-5). The proper therapeutic approach is still debated due to the rarity and heterogeneity of these conditions and, to date, a patient-tailored therapy and follow-up are recommended. Keeping a good hydration and avoid any kind of physical activity that can increase blood viscosity (i.e. diving with oxygen tank, climbing, sky-diving, smoking) is mandatory, as well as performing venous thromboembolism prophylaxis when needed.

**References**

1. Bento C, Percy MJ, Gardie B, Maia TM, van Wijk R, Perrotta S, et al. Genetic basis of congenital erythrocytosis: mutation update and online databases. Hum Mutat. 2014;35:15-26.

2. Perrotta S, Stiehl DP, Punzo F, Scianguetta S, Borriello A, Bencivenga D, et al. Congenital erythrocytosis associated with gain-of-function HIF2A gene mutations and erythropoietin levels in the normal range. Haematologica. 2013;98:1624-32.

3. Perrotta S, Cucciolla V, Ferraro M, Ronzoni L, Tramontano A, Rossi F, et al. EPO receptor gain-of-function causes hereditary polycythemia, alters CD34 cell differentiation and increases circulating endothelial precursors. PLoS One. 2010;5:e12015.

4. Perrotta S, Della Ragione F. The HIF2A gene in familial erythrocytosis. N Engl J Med. 2008;358:1966.

5. Perrotta S, Nobili B, Ferraro M, Migliaccio C, Borriello A, Cucciolla V, et al. Von Hippel-Lindau-dependent polycythemia is endemic on the island of Ischia: identification of a novel cluster. Blood. 2006;107:514-9.

## A108 The nurse in the pediatric family surgery; yesterday, today and tomorrow

### Silvia Piccirillo (silvia.picci2590@gmail.com)

#### Studio AIP, Firenze, Italy

In this work we describe a survey aimed at nurses who collaborate in the manage the pediatric family surgery. Carried out in order to analyse the current role of the nurse. The data obtained show the degree of nursing autonomy, the role and the activities they perform within the clinic and their willingness to introduce new activities in order to get to take charge of the whole family, thus expanding your role. Also, the survey pointed out the degree of professional satisfaction, the relationship with the pediatrician and the entire crew that work in the clinic (secretary, health assistant, etc.). The report will describe the change in the role of the nurse from his origins to the present day; the innovations brought in the nursing community and the gradual implementation of training schools: initially boarding schools followed by vocational training courses and finally by degree courses. In our opinion, the new nursing professional should be given more decision-making space both in the educational field and in clinical practice and would have the right to be heard in decision-making forums. To reduce overcrowding in hospital centers the new health care system must face up to necessity of users to have access to extra – hospital services, and health professionals have to use resources to the new population’s demand. It is in this reality that the figure of the family nurse arises; a link between the user and the doctors of the territory, a facilitator that allows to guarantee to the users the right hospital services and allows to manage the chronic patient on the territory. About pediatric clinic, nurse takes care of the whole family, no more like a small family unit, but, by now, an enlarged "community".

## A109 Growth and nutrition: epigenetic role from Leonardo to Barker

### Angelo Pietrobelli^1,2,3^, Marco Zaffanello^2^, Franco Antoniazzi^2^, Luca Pecoraro^2^

#### ^1^UOS I Primi Mille Giorni del Bambino per la Prevenzione delle Patologie Non Trasmissibili dell’Adulto, Italy; ^2^Clinica Pediatrica - Università degli Studi di Verona, Verona, Italy; ^3^Pennington Biomedical Research Center Baton Rouge, LA, USA

##### **Correspondence:** Angelo Pietrobelli (angelo.pietrobelli@univr.it)

**Background**: Three decades ago, Barker noticed an odd correlation on a map: the poorest regions of England and Wales were the ones with the highest rates of heart disease. Why would this be, he wondered, when heart disease was supposed to be a condition of affluence of sedentary lifestyles and rich food? After comparing the adult health of some 15,000 individuals with their birth weight, he discovered an unexpected link between small birth size (1921-25)— often an indication of poor prenatal nutrition — and heart disease in middle age (1968-78) [1]. Faced with an inadequate food supply, the fetus diverts nutrients to its most important organ, the brain, while skimping on other parts of its body — a debt that comes due decades later in the form of a weakened heart [1-3]. The first 1000 days of life, the period from conception to age of two, is fundamental for body and brain development and is the best time to build healthy nutrition and subsequent healthy growth [4]. In this crucial period many growth drivers, such as genetic and epigenetic factors, and hormonal regulation, can have important effects on fetal and postnatal growth, on development and on risk of developing common non-communicable diseases later in life [5].

**Material and methods**: Aim of this talk, based on literature review, was to develop and discuss a proposal of ten good practices to help to prevent obesity in the unique window of opportunity, the first 1000 days of life.

**Results**: Good practices: 1. Both mother and father behavior matter, even before conception. 2. Before and during pregnancy, at birth and during early life body fat and composition should be measured. 3. Exclusive breastfeeding for the best start in life. 4. Begin weaning between 4 and 6 months. 5. Fruit and vegetable liking begin early. Repeated exposure up to 8 times are efficient strategies to increase acceptance of food not well accepted at first. There is no need to add sugar or salt during complimentary feeding. 6. Respect child appetite. 7. Control animal protein intake. 8. Assure adequate qualitative fat intake. 9. Parents have a model role in feeding, with TV and other screens turned of during meals. 10. Promoting physical activity and child has to get sleep sufficiently.

**Conclusion**: Given the suggestion described in this presentation, concerted public health efforts are needed to achieve the healthy objectives for obesity and nutrition and to fight the childhood obesity epidemic [6].

**References**

1. Barker DJ, Osmond C. Infant mortality, childhood nutrition, and ischaemic heart disease in England and Wales. Lancet. 1986;1:1077-81.

2. Barker DJ, Winter PD, Osmond C, Margetts B, Simmonds SJ. Weight in infancy and death from ischaemic heart disease. Lancet. 1989;2:577-80.

3. Barker DJ, Gluckman PD, Godfrey KM, Harding JE, Owens JA, Robinson JS. Fetal nutrition and cardiovascular disease in adult life. Lancet. 1993;341:938-41.

4. Pietrobelli A, Agosti M, Zuccotti G. Putting the Barker Theory into the Future: Time to Act on Preventing Pediatric Obesity. J Environ Res Public Health. 2016;13.

5. Godfrey KM**,** Lillycrop KA, Burdge GC, Gluckman PD, Hanson MA. Non imprinted epigenetics in fetal and postnatal development and growth. In: Gillman MW, Gluckman PD, Rosenfeld RG, editors. Recent advances in growth research: nutritional, molecular and endocrine perspectives. Nestlè Workshop, Nestlè Ltd, Vevey/s. Basel (Switzerland): Karger AG; 2013. p. 57-63.

6. Pietrobelli A, Agosti M, MeNu Group. Nutrition in the First 1000 Days: Ten Practices to Minimize Obesity Emerging from Published Science. Int J Environ Res Public Health. 2017;14: pii: E1491.

## A110 General Movements (GMs): assessment and predictive value

### Ettore Piro, Vita Angileri, Veronica Vanella, Claudio Montante, Veronica Notarbartolo, Irene Greco, Giulia Mincuzzi, Giovanni Corsello

#### Department of Health Promotion Sciences, Maternal and Infant Care, Internal Medicine and Medical Specialties “G. D’Alessandro”. University of Palermo, Palermo, Italy

##### **Correspondence:** Ettore Piro (ettore.piro@unipa.it)

In the early 1980s De Vries, Visser, Prechtl and Hopkins, described the emerging of General Movements (GMs) in fetal life and their evolution from preterm to 3-5 months corrected age. [1,2,3] During the first two months post-term, GMs are described as having an elliptical form defined “writhing”, thereafter at 6 to 9 weeks post-term age a more complex type of movements gradually emerges involving the neck, trunk and limbs, characterized by moderate speed with variable acceleration in all directions and defined “fidgety movements” (FMs). The introduction in clinical practice of GMs assessment offered to neonatologists, developmental pediatricians and child neuropsychiatrists an important tool for early identification of infants with an increased risk of neurological and developmental impairment. The persistence of atypical GMs (defined “poor repertoire”, “cramped synchronized” and “chaotic”), without a normally appearing FMs from 6-9 to 20 (mean 12) weeks of age (corrected for prematurity), is generally considered an ominous sign predictive of cerebral palsy (CP)[4]. The integration of perinatal history, clinical evaluation, cerebral ultrasonographic-doppler examination, biochemical and neurophysiological investigations, might suggest in neonatal period a diagnosis and the relative prognostic profile. Advanced cerebral imaging techniques (cerebral DWI and conventional MR), carrying the most accurate neurodevelopmental prognostic value, are not always available in all neonatological contexts. A recent study showed in term infants with hypoxic ischemic encephalopathy a strict correlation between GMs and the site and severity of brain lesions assessed by early cerebral MR. Central gray matter involvement leads to cramped-synchronized GMs and poor motor outcome [5]. Thus, the assessment of general movements being non-intrusive, easy to acquire, cost-effective and with high predictive values, is considered a reliable prognostic clinical tool, especially in low-resource contexts. In recent years many researchers implemented the studies on GMs, focusing on their neural substrate and central pattern generators, more detailed assessment of GMs, extraction of movement patterns from video records for analysis of movements of skeletal key points and correlation with long term neuropsychiatric disorders such as autism and ADHD [6-10]. Moreover, the opportunity for the clinician to receive home video records of GMs, might help to mark the dates of clinical assessment and to plan a more individualized follow up. This opportunity has been recently standardized, using a video recording application for smartphones, in research institutes to assist in early detection of CP and other neurodevelopmental outcomes [11].

**References**

1. de Vries JI, Visser GH, Prechtl HF. The emergence of fetal behaviour. I. Qualitative aspects. Early Hum Dev. 1982;7:301-322.

2. Hopkins B, Prechtl HFR. A qualitative approach to the development of movements during early infancy, In: Precthl HFR, editor.Continuity of Neural Functions from Prenatal to Postnatal Life, Clinics in Developmental Medicine N. 94. London (UK), Spastic International Medical Publication; 1984. p. 179–197.

3. Prechtl HFR, B. Hopkins B. Developmental transformations of spontaneous movements in early infancy. Early Hum Dev. 1986;14:233–238.

4. Kwong AKL, Fitzgerald TL, Doyle LW, Cheong JLY, Spittle AJ. Predictive validity of spontaneous early infant movement for later cerebral palsy: a systematic review. Dev Med Child Neurol 2018;60:480–489.

5. Ferrari F, Todeschini A, Guidotti I, Martinez-Biarge M, Roversi MF, Berardi A, et al. General movements in full-term infants with perinatal asphyxia are related to basal ganglia and thalamic lesions. J Pediatr. 2011;158:904-911.

6. Ferrari F, Frassoldati R, Berardi A, Di Palma F, Ori L, Lucaccioni L, et al.The ontogeny of fidgety movements from 4 to 20 weeks post-term age in healthy full-term infants. Early Hum Dev 2016;103:219–224.

7. Einspieler C, Marschik PB, Pansy J, Scheuchenegger A, Krieber M, Yang H, et al. The general movement optimality score: a detailed assessment of general movements during preterm and term age. Dev Med Child Neurol. 2016;58:361–368.

8. Marchi V, Hakala A, Knight A, D'Acunto F, Scattoni ML, Guzzetta A, Vanhatalo S. Automated pose estimation captures key aspects of General Movements at eight to 17 weeks from conventional videos. Acta Paediatr. 2019. doi: 10.1111/apa.14781.

9. Einspieler C, Sigafoos J, Bölte S, Bratl-Pokorny KD, Landa R, Marschik PB. Highlighting the first 5 months of life: General movements in infants later diagnosed with autism spectrum disorder or Rett Syndrome. Res Autism Spectr Disord. 2014;8:286-291.

10. Athanasiadou A, Buitelaar JK, Brovedani P, Chorna O, Fulceri F, Guzzetta A, Scattoni ML. Early motor signs of attention-deficit hyperactivity disorder: a systematic review. Eur Child Adolesc Psychiatry. 2019. doi: 10.1007/s00787-019-01298-5.

11. Spittle AJ, Olsen J, Kwong A, Doyle LW, Marschik PB, Einspieler C, Cheong J. The Baby Moves prospective cohort study protocol: using a smartphone application with the General Movements Assessment to predict neurodevelopmental outcomes at age 2 years for extremely preterm or extremely low birthweight infants. BMJ Open. 2016;6:e013446.

## A111 Observational study of headache in Pediatric Emergency Department

### Roberta Puxeddu^1^, Rino Agostiniani^1^ , Andrea Messeri^2^, Anna Salvatore^3^, Lucia Calistri^3^, Stefano Masi^3^, Giuliana Rizzo^4^

#### ^1^Department of Pediatrics, Saint Jacopo Hospital , Pistoia, Italy; ^2^ Department of Pain Therapy and Palliative Care, University Hospital Meyer, Florence, Italy; ^3^ Pediatric Emergency Department of University Hospital Meyer, Florence, Italy; ^4^ Department of Pediatric Neuroanesthesiology, University Hospital Meyer, Florence, Italy

##### **Correspondence:** Roberta Puxeddu (gigi.puxeddu50@gmail.com)

**Background**

Headache in children is the most common cause of admission in the Emergency department. The most common type of Primary headaches among children are migraine and tension type headache (TTH), however their differential diagnosis is not completely clear and they often are mixed headaches with few studies in literature. The aim of our study is to identify and to manage the pain in the likely primary headache in particular migraine and TTH among patients admitted in Pediatric emergency department in Meyer Hospital.

**Materials and Methods**

It is an observational, prospective, descriptive study lasted one year (2016-2017**).** Children aged 4-17 years meeting diagnostic criteria for likely primary headache according to the 2013 ICHD3-Beta were included[1]. Pain level was assessed at the arrival, at 1 and 2 hours after paracetamol (20 mg/kg orally or 15 mg/kg IV) or ibuprofen (10mg/kg orally) administration according to OMS guidelines and pain scales [2]. The primary end point was reached when the reduction of pain was by at least 2 points or intensity ≤ 3 at 2 hours after drugs administration. The study was approved by Tuscany Pediatric Ethics Committee on 22nd November 2016, approval number 1. Informed consent to publish has been obtained from the parents of the children.

**Results**

Of 385 children 100 children had likely primary headache who had 35% TTH, 21%, mixed headaches, 54% migraine. All the likely primary headache were confirmed by Headache Centre later 87% of children reached the primary endpoint. Ibuprofen (OR 24.00) is more effective in TTH at 2 hours after administration compared to paracetamol (OR 6.00). Paracetamol administered both orally (OR 2.00) and intravenously IV (OR 2.50) is more effective in mixed headaches than ibuprofen (OR 1.75). Ibuprofen (OR 5.50) and paracetamol administered IV (OR 5.50) are more effective than paracetamol administered orally (OR 1.50).

**Conclusion**

It may need easier criteria for primary headache's diagnosis, in Emergency department considering multidisciplinary approaches, including family education for the correct management of pain in primary headache in children. Ibuprofen maybe more effective in the migraine and TTH than paracetamol administered orally, but it needs more studies to confirm our preliminary studies.

**References**

1. Headache Classification Committee of the International Headache Society (IHS). The International Classification of Headache Disorders, 3rd edition. Cephalalgia. 2013;33:629-808.

2. World Health Organization. WHO guidelines on the pharmacological treatment of persisting pain in children with medical illnesses. Geneva: World Health Organization; 2012. pp 26-41.

## A112 Clinical features of the newborn with neuromuscular disease

### Carmelo Rachele (carmelo.rachele@virgilio.it)

#### Pediatra di Famiglia FIMP (Federazione Italiana Medici Pediatri), Board Scientifico Malattie Neuromuscolari, Esperto sui Disturbi del Neurosviluppo, Italy

Neuromuscular pathologies are characterized by structural and functional alterations at the level of the motor unit, varying, in relation to the site of the lesion, in diseases of the motor neuron, peripheral neuropathies, myasthenic syndromes and myopathies. The classification of these different forms of pathologies is constantly evolving in relation to the new knowledge of molecular genetics, that have revolutionized, in recent years, the etiopathogenetic and pathophysiological features of numerous hereditary degenerative forms, allowing, in many cases to isolate the genes and to identify the encoded proteins.

Duchenne muscular dystrophy, characterized by progressive atrophy and weakness of skeletal and myocardial muscles, is the most widespread neuromuscular genetic disease in children. Prompt diagnosis is an absolute priority in order to achieve an early therapeutic and rehabilitative pathway, that has a positive impact on the progression of the disease and that is capable of improving family members and patients’ quality of life. Children with neuromuscular disorders generally have deficits in gross motor skills, but delays in fine motor and cognitive skills may also be present. Careful anamnestic and neurological examination of the newborn is essential for the early recognition of risk indicators and related warning signs. For a thorough neuro-evolutionary evaluation of the newborn, it is necessary to investigate spontaneous motor skills, posture, muscle tone and osteotendinous reflexes and to initiate, in case of clinical suspicion, a diagnostic process to confirm the existence of a neuromuscular disease.

## A113 Acute Ataxia

### Umberto Raucci (umberto.raucci@opbg.net)

#### Pediatric Emergency Department, Bambino Gesù Children’s Hospital, IRCCS, Rome, Italy

Ataxia is a neurological sign consisting of impaired coordination of motor activity. It is principally due to a dysfunction of the complex circuitry connecting the basal ganglia, cerebellum and cerebral cortex. Affected patients classically present a wide-based gait, truncal instability, dysmetria, dysdiadochokinesia, intention tremor, dysarthria and nystagmus [1-4]. From an etiological point of view, ataxia is classified as acquired, inherited or sporadic. The course may be distinguished in acute, intermittent and recurrent, chronic-non-progressive and chronic-progressive. Only a few studies have described the different conditions that can be found in children presenting with acute ataxia (AA), but conclusions were divergent because of the different settings of recruitment, small sample sizes and heterogeneous study designs [4–9]. Few data exist about the real incidence and prevalence of AA in childhood [4]. A large Italian multicenter study on AA, conducted in a pediatric emergency setting, shows a frequency of 0.02% of all emergency attendances [5]. Two previous monocentric pediatric emergency department-based studies reported a frequency of 0.024% and 0.04% [8,9]. AA in childhood poses a diagnostic dilemma because of the broad differential diagnosis. While the most common causes have a “benign” self-limited course, AA can be due to potentially invalidating or life-threatening conditions, including tumour and central nervous system infection, requiring early diagnosis and prompt intervention [1-3].

In a very recent Italian multicenter study it emerged that AA is a relatively uncommon neurological emergency in childhood. Acute post-infectious cerebellar ataxia is the first cause of acute ataxia in children, with varicella-zoster the most frequently involved pathogen. Brain tumors are the second most common cause of pediatric acute ataxia while clinically urgent neurological pathology (CUNP) accounts for over a third of cases. The Authors defined CUNP as any nervous system disorder requiring early diagnosis and prompt medical or surgical intervention to prevent disabling or life-threatening evolution, namely neoplastic, cerebrovascular and infectious CNS disorders, demyelinating diseases, acute neuropathies (AN), genetic or metabolic disorders, and CNS malformations [5]. In AA assessment, the presence of focal neurological or meningeal signs, hyporeflexia, ophtalmoplegia, seizures, or a longer time of evolution of symptoms, is associated with a higher risk of a severe underlying pathology [5]. A careful history, together with a systematic general and neurological examination, can help narrow down the differential diagnosis and selected investigations are recommended to quickly identify patients with potentially life-threatening causes of AA.

**References**

1. Caffarelli M, Kimia AA, Torres AR. Acute Ataxia in Children: A review of the differential diagnosis and evaluation in the emergency department. Pediatr Neurol. 2016;65:14-30.

2. Pavone P, Praticò AD, Pavone V, Lubrano R, Falsaperla R, Rizzo R, et al. Ataxia in children: early recognition and clinical evaluation. Ital J Pediatr. 2017;43:6.

3. Whelan HT, Verma S, Guo Y, Thabet F, Bozarth X, Nwosu M, et al.Evaluation of the child with acute ataxia: a systematic review. Pediatr Neurol. 2013;49:15-24.

4. Poretti A, Benson JE, Huisman TA, Boltshauser E. Acute ataxia in children: approach to clinical presentation and role of additional investigations. Neuropediatrics. 2013;44:127-41.

5. Garone G, Reale A, Vanacore N, Parisi P, Bondone C, Suppiej A, et al. Acute ataxia in paediatric emergency departments: a multicentre Italian study. Arch Dis Child. 2019. doi: 10.1136/archdischild-2018-315487.

6. Gieron-Korthals MA1, Westberry KR, Emmanuel PJ. Acute childhood ataxia: 10-year experience. J Child Neurol. 1994;9:381-4.

7. Martínez-González MJ, Martínez-González S, García-Ribes A, Mintegi-Raso S, Benito-Fernández J, Prats-Viñas JM. Acute onset ataxia in infancy: its aetiology, treatment and follow-up. Rev Neurol. 2006;42:321-4.

8. Rudloe T, Prabhu SP, Gorman MP, Nigrovic LE, Harper MB, Landschaft A, Kimia AA. The yield of neuroimaging in children presenting to the emergency department with acute ataxia in the post-varicella vaccine era. J Child Neurol. 2015;30:1333-9.

9. Thakkar K, Maricich SM, Alper G. Acute ataxia in childhood: 11-year experience at a major pediatric neurology referral center. J Child Neurol. 2016;3:1156-60.

## A114 Appropriateness in paediatrics

### Nicola A. Romeo (nicant.rom@gmail.com)

#### Unit of Paediatrics, San Marino State Hospital, Republic of San Marino

The theme of appropriateness in care providing have been the centre of health policy during the last 20 years, representing the most important quality factor, bringing in itself the concepts of efficacy, efficiency, adequacy, accessibility, timeliness. This paper aim to highlights the complexity that characterise the concept of appropriateness in healthcare, both in the organizational and in the prescriptive dimension, and the lack adequate indicators/standards for its assessment, in particular for childhood health care. Among the different definitions reported in the literature, the one provided by the Italian Health Ministry defined as “appropriate any health intervention (preventive, diagnostic, therapeutic, rehabilitative) related to the patient's (or community) provided in adequate manner and time, based on recognised standards with a positive balance between benefits, risks and costs” [1]. Basing on this definition, we can do some considerations:

- The need for efficacy indicators about health interventions provided to patients;
- The need for optimization of available resources in order to guarantee healthcare to each one and to the community, which is an essential condition for the viability of the National Health Service;
- The need for considering preferences and expectations of each patient, avoiding any economic influence.

About efficacy indicators, we should consider that these are available just for some health interventions, like the diagnostic or therapeutic ones. The optimization of the use of available resources in order to satisfy the different health needs of the community represent a vital target for the National Health Service viability, strictly relating the concept of appropriateness and the economic aspect of the health services offered. The evaluation of the benefit/risk ratio for the healthcare interventions and the need to consider the patient as the pivot of the healthcare-related choices highlights the close connection existing between appropriateness and ethic.

Moreover, it is important that healthcare professionals indicate clearly to the healthcare policy managers the appropriateness standards that the National Health System must guarantee to the different stakeholders, firstly to the patients. Hence, the need to enrich the current roster of indicators for appropriateness in paediatric cares, both in the hospital setting and in the primary care.

**Reference**

1. Glossario Ministero della Salute. www.salute.gov.it/portale/temi/p2_6.jsp?id=314&area=qualità£menu=sicurezza

Accessed 8th August 2019.

## A115 Infectious encephalopathies versus non-infectious: how to distinguish them

### Anna Rosati (anna.rosati@meyer.it)

#### Centro di Eccellenza in Neuroscienze, Azienda Ospedaliero-Universitaria “A. Meyer”, Firenze, Italy

**Background** Encephalitis in paediatric age recognizes a wide range of etiologies, clinical presentations, and outcomes. A major challenge in diagnosis is that different symptoms may appear at different times and different levels of intensity, so that the disease may mimic many other disorders. Identification of the etiology is a priority, since it allows for a prompt and specific treatment, avoiding unnecessary medications and laboratory tests, and it may contribute to improve prognosis.

**Material and Methods** A Medline research was made with the following terms ("encephalitis/diagnosis"[Mesh] OR "encephalitis/etiology"[Mesh]) AND children, and with the filters “Review”, “Humans”, “English”, “Child: birth-18 years”. We only kept articles published between January 2000 and May 2019.

**Results:** A total of 394 articles were obtained and 77 of them contribute to this review. The main limitations of the studies were their retrospective design. Neurological manifestations of infections of the CNS vary with the type and severity of the infection, and include headache, meningismus, seizures, cognitive and behavioral disturbances, ataxia, and altered levels of consciousness up to coma. Autoimmune encephalitis (AE) is a severe condition in which the immune system attacks the brain, impairing function. In contrast to typical cases of infective encephalitis, which tend to begin relatively abruptly over 24-72 hours, AE can begin and progress insidiously, often over a period of days to a few weeks. Neuro-psychiatric symptoms, such as anxiety, agitation, delusional or paranoid thoughts, mutism or catatonia, memory dysfunction, and visual or auditory hallucinations, are the prominent manifestations of AE in both adults and children. Approximately 50% of children with AE have seizures or movement disorders (choreoathetosis and orofacial dyskinesia). Several antibodies have been identified, including auto-antibodies directed against NMDA, LGI1, CASPR2, VGKC-complex antibodies, AMPA, and GABA. In some patients, encephalopathy can be part of a paraneoplastic phenomenon, although in children with AE, an underlying malignancy is rare. Nevertheless, there is a group of patients with suspected AE who are antibody negative in which etiology remains unknown. Steroid-responsive encephalopathy associated with autoimmune thyroiditis (Hashimoto encephalopathy) in children or adolescents can begin abruptly or subacutely, producing seizures, psychosis, chorea, tic, coma, migraine-like headaches, or acute focal neurological dysfunction.

**Conclusions**. Infectious and non-infectious encephalitis share similar clinical signs and symptoms. However, neuro-psychiatric symptoms and refractory seizures are more commonly observed in either autoimmune or unknown etiologies. The poor outcome, especially in the latter, underlines the importance of future research in this area.

## A116 Adverse drug reactions: to be or not to be?

### Francesca Saretta (francescasaretta@gmail.com)

#### Pediatric Department, AAS2 Bassa Friulana-Isontina, Palmanova (UD), Italy; Pediatric Allergy Unit, Department of Medicine, Udine (UD), Italy

In the last decades it has been observed an increase in antibiotic resistance [1, 2], probably due both to drug misuse and to “*drug allergy*” mislabeling. When dealing with a possible adverse drug reaction, a careful word selection must be done: some reactions are indeed due to collateral effects (type A reactions), others are hypersensitivity reactions (type B), while only a few are true “*drug allergy*”. Especially in childhood, “*antibiotic allergy*” mislabeling could be difficult to remove without proper (and sometimes extensive) evaluation, and could, therefore, lead to antibiotic misuse. It is important to properly and timely perform an allergological evaluation in order to avoid those mislabeling errors.

Type A (Augmented) reactions are due to pharmacological properties of the drug, are usually predictable, such as antihistamine drowsiness, and should be addressed before antibiotic use. Type B (Bizarre) reactions are, instead, unpredictable, and comprise 15% of all adverse drug reactions. The vast majority are due to an immune system alterations and are usually defined as “drug hypersensitivity” (DHR) [3]. Some recognize inflammatory mechanisms or immunological reactions as described by Gells and Coombs. Hypersensitivity reactions due to an apten-carrier mechanism (Type I) show typical IgE mediated signs and symptoms (urticaria, angioedema, anaphylaxis). Other type of hypersensitivity reactions have been described (Type II-IV) and require specific in vivo and in vitro tests to be ruled out.

The allergological evaluation of DHR relies mostly on a careful anamnesis: timing of reaction, drug type and dose, signs and symptoms description, presence of atopy, familiarity, cofactors such as concurrent infections, clinical evolution. According to type of reaction (immediate or delayed, mild or severe) different skin and blood tests could be performed, followed by a challenge test. In mild cutaneous delayed reactions, updated guidelines suggest to directly perform an oral challenge test [4, 5]. In SCAR (severe cutaneous drug reaction) almost all guidelines suggest to avoid drug without performing any tests.

**References**

1) Nicolini G, Sperotto F, Esposito S. Combating the rise of antibiotic resistance in children. Minerva Pediatr. 2014; 66: 31-9

2) Smith RA, M'ikanatha NM, Read AF. Antibiotic resistance: a primer and call to action. Health Commun. 2015; 30: 309-14

3) Pichler WJ. Immune pathomechanism and classification of drug hypersensitivity. Allergy 2019 ;74:1457-1471.

4) Caubet JC, Eigenmann PA. Curr Opin Allergy Clin Immunol 2012;12:341-347.

5) Gomes ER, Brockow K, Kuyucu S, Saretta F, Mori F, Blanca-Lopez N, et al. Drug hypersensitivity in children: report from the pediatric task force of the EAACI Drug Allergy Interest group. Allergy. 2016;71:149-61.

## A117 Development of the active surveillance for the neurodevelopmental disorders’ early detection: the collaborative approach at the Istituto Superiore di Sanità

### Maria Luisa Scattoni^1^, Antonella Costantino^2^, Alberto Villani^3^, Fabio Mosca^4^, Federica Zanetto^5^, Paolo Biasci^6^, Rinaldo Missaglia^7^

#### ^1^Research Coordination and Support Service, Istituto Superiore di Sanità, Rome, Italy; ^2^Società Italiana di Neuropsichiatria dell’Infanzia e dell’Adolescenza, SINPIA, Milano, Italy; ^3^Società Italiana di Pediatria, SIP, Rome, Italy; ^4^Società Italiana di Neonatologia, SIN, Milano, Italy; ^5^Associazione Culturale Pediatri, ACP, Narbolia, Oristano, Italy; ^6^Federazione Italiana Medici Pediatri, FIMP, Rome, Italy; ^7^Sindacato Medici Pediatri di Famiglia, SiMPeF, Rome, Italy

##### **Correspondence:** Maria Luisa Scattoni (marialuisa.scattoni@iss.it)

**Background**

Neurodevelopmental disorders require early diagnosis and timely interventions. Routinely pediatric assessments, properly implemented, could be a strategic observatory for applying sustainable strategies for the early detection of neurodevelopmental disorders. Last November 4th 2017, the Istituto Superiore di Sanità, the Società Italiana di Neuropsichiatria dell'Infanzia e dell'Adolescenza together with the Federazione Italiana Medici Pediatri, the Associazione Culturale Pediatri, the Sindacato Medici Pediatri di Famiglia and the Società Italiana di Pediatria signed an agreement within the framework of the project ‘Osservatorio nazionale per il monitoraggio dei disturbi dello spettro autistico’ to promote the implementation of the well-baby check-up and establish a formal network between pediatricians and child neuropsychiatrists throughout the entire Italian territory. The education system is also crucial in the first years of life and, thanks to the establishment of the integrated system 0-6, it will now be possible to promote new and more stable professionals and novel territorial networks also between educators and health workers.

**Materials and methods**

The early detection of neurodevelopmental disorders tool, applicable within the routinely pediatric assessment, was implemented in three steps. First, we completed a systematic overview review that collected the systematic reviews and meta-analysis describing early behavioral markers of neurodevelopmental disorders in the first three years of the child's life. More than 1500 works were screened and 5 were selected.(1-5) The overview review provided evidences for delays in the gross and fine motor development, in the expressive and receptive language development, and reduced social engagement in children later diagnosed with neurodevelopmental disorders. Second, we collected and evaluated the existing national and international protocols for the early detection of neurodevelopmental disorders. (6) Third, the group selected in the reviews/protocols collected in the steps 1 and 2 the items solved by the 90% of typical developmental children.

**Results**

Using the materials systematically collected, the group developed a protocol including 8 worksheets for each routinely pediatric assessment at 1, 3, 6, 9, 12, 18, 24, 36 months. Each worksheet contains 6 to 8 questions about the infant/child neurodevelopment (motor, language/social/communicative/play and emotion regulation/ self-regulatory skills).

The present protocol will be disseminated on the Italian territory and provided to all Italian pediatricians for its inclusion in the routinely pediatric assessment. In addition, a brief and targeted training on the protocol implementation will be provided in all local health authorities (Aziende Sanitarie Locali) for its widest dissemination.

**Acknowledgements**

Italian Ministry of Health Project – Capitolo 2S57 [Articolo 1, comma 401, Legge 28 dicembre 2015, n. 208, recante “Disposizioni per la formazione del bilancio annuale e pluriennale dello Stato (legge di stabilità 2016)”].

The abstract is being presented on behalf of the ISS Neurodevelopmental Disorders group.

**References**

1. Athanasiadou A, Buitelaar JK, Brovedani P, Chorna O, Fulceri F, Guzzetta A, & Scattoni ML. Early motor signs of attention-deficit hyperactivity disorder: a systematic review. Eur Child Adolesc Psychiatry. 2019. doi: 10.1007/s00787-019-01298-5.

2. Fisher EL. A systematic review and meta-analysis of predictors of expressive-language outcomes among late talkers. J Speech Lang Hear Res. 2017;60:2935-2948.

3. Fuentefria RDN, Silveira RC, & Procianoy RS. Motor development of preterm infants assessed by the Alberta Infant Motor Scale: Systematic review article. J Pediatria. 2017;93:328-342.

4. Garrido D, Petrova D, Watson LR, Garcia‐Retamero R, & Carballo G. Language and motor skills in siblings of children with autism spectrum disorder: A meta‐analytic review. Autism Res. 2017;10:1737-1750.

5. Palomo R, Belinchón M, & Ozonoff S. Autism and family home movies: a comprehensive review. J Develop Behav Pediatr. 2006; 27: S59-S68.

6. Ertem, IO, Krishnamurthy V, Mulaudzi MC, Sguassero Y, Balta H, Gulumser O, et al. Similarities and differences in child development from birth to age 3 years by sex and across four countries: a cross-sectional, observational study. Lancet Glob Health. 2018;6:e279-e291.

## A118 Arfid: Avoidant/Restrictive Food Intake Disorder

### Valentina Sottili, Giuseppe Banderali

#### Department of Clinical Paediatric, San Paolo Hospital, University of Milan, Milan, Italy

##### **Correspondence:** Valentina Sottili (valentina.sottili@unimi.it)

DSM-5 defines Avoidant/Restrictive Food Intake Disorder (ARFID) as a persistent failure to meet appropriate nutritional and /or energy needs, associated to one or more among significant weight loss/failure to achieve expected weight gain, significant nutritional deficiency, dependence on enteral feeding/oral nutritional supplements and marked interference with psychosocial functioning. The disturbance can't be better explained by lack of available food or by an associated culturally sanctioned practice. It doesn't occur exclusively during the course of anorexia or bulimia nervosa, from which differ for the absence of concern about body's weight or shape. Finally, ARFID is not attributable to a concomitant medical condition or it isn't better explained by another mental disease. When co-exist a mental disorder, the severity exceeds that routinely associated with the basal disease. Three primary presentations of ARFID are described, which can occur independently or in combination,[1]and a three-dimensional model is suggested into the pathogenesis [2] . Firstly a sensory sensitivity may led to avoid eating some foods due to aversions to specific tastes or smells (severe “picky and fussy eaters” are more at risk). Secondly some patients show complete lack of interest in eating, as fast eaters (decreased activation of the hypothalamus and anterior insula). Thirdly children may stop eating entirely following a traumatic experience meal associated as choking, vomiting or other forms of gastroenterological distress (negative valence systems).

Incidence and prevalence of ARFID in the general population are unknown because of the lack of large epidemiological studies, even if literature shows it might be common among children in the general population. Males aged 8-13 years seem to be more at risk, [3] but the disease may be present also in younger than 6 years.[4] A prevalence of 1,5% is described among gastrointestinal disorders aged 8-18 years [5] and betweeen 7.2% and 17.4% across eating disorders. [6] Psychiatric comorbidities, including anxiety disorders, autism spectrum disorder and attention deficit hyperactivity disorder (ADHD) are common among individuals with ARFID [7]. ARFID can lead to severe medical sequelae due to malnutrition. Retrospective studies show risk for amenorrhea, bradycardia, prolonged QT interval and hypokalemia till to neurological degeneration because of folate deficiency. It seems ARFID may also be the clinical presentation of PANS (pediatric acute-onset neuropsychiatric syndrome) and PANDAS (pediatric autoimmune neuropsychiatric disorder associated with streptococcal infections) [8]. There aren't still current guidelines in treatment of ARFID.[9] A multidisciplinary approach is necessary [10].

**References**

1. American Psychiatric Association. Diagnostic and statistical manual of mental disorders (DSM-5). Washington, DC (US): American Psychiatric Publishing; 2013.

2. Thomas JJ, Lawson EA, Micali N, Misra M, Deckersbach T, Eddy KT. Avoidant/restrictive food intake disorder: a three-dimensional model of neurobiology with implications for etiology and treatment. Curr Psychiatry Rep. 2017;19:54.

3. Norris ML, Robinson A, Obeid N, Harrison M, Spettigue W, Henderson K. Exploring avoidant/ restrictive food intake disorder in eating disordered patients: a descriptive study. Int J Eat Disord. 2014; 47:495–9.

4. Norris ML, Spettigue WJ, Katzman DK. Update on eating disorders:Current perrspective on avoidant/restrictive food intake disorder in child and youth. Neuropsychiatr Dis Treat. 2016; 12:213-218.

5. Eddy KT, Thomas JJ, Hastings E, Edkins K, Lamont E, Nevins CM, et al.. Prevalence of DSM-5 avoidant/restrictive food intake disorder in a pediatric gastroenterology healthcare network. Int J Eat Disord. 2015; 48: 464–70.

6. Nicely TA, Lane-Loney S, Masciulli E, Hollenbeak CS, Ornstein RM. Prevalence and characteristics of avoidant/restrictive food intake disorder in a cohort of young patients in day treatment for eating disorders. J eat disord. 2014; 8 2;2(1):21.

7. Pennel A, Couturier J Granct C, Johnson N. Severe avoidant/restrictive food intake disorder and coexisting stimulant treated attention deficit hyperactivity disorder. Int J Eat Disord. 2016; 49(11):1036-1039.

8. Toufexis MD, Hommer R, Gerardi DM, Grant P, Rothschild L, D' Souza P, et al. Disordered eating and food restrictions in children with PANDAS/PANS. J Chil Adolesc Psychopharmacol. 2015; 25(1):48-56.

9. Kelly NR, Shank LM, Bakalar JL, Tanofskykraff M. Pediatric feeding and eating disorders: Current state of diagnosis and treatment. Curr Psychiatry Rep. 2014; 16 (5):446.

10. Ozier AD, Henry BW. American Dietetic Association. Position of the american dietetic association: Nutrition intervention in the treatment of eating disorders. J Am Diet Assoc. 2011; 111 (8): 1236-1241.

## A119 Diet and functional gastrointestinal disorders

### Angela Maria Caprio^1^, Marianna Casertano^1^, Sabrina Cenni^2^, Alessandra Vitale^1^, Caterina Strisciuglio^1^

#### ^1^Department of Woman, Child and General Specialized Surgery, University of Campania “ Luigi Vanvitelli”, Napoli, Italy; ^2^Department of Translational and Medical science, Section of Pediatrics, University of Naples “Federico II”,Napoli, Italy

##### **Correspondence:** Caterina Strisciuglio (caterina.strisciuglio@unicampania.it)

**Background and aims :** Functional gastrointestinal disorders (FGIDs) are common disorders characterized by chronic or recurrent gastrointestinal symptoms, not related to structural or biochemical abnormalities. The etio-pathogenesis of FGIDs is still unclear. The diet seems to play an important role in these disorders, in particular in functional abdominal pain. Indeed, most adult and children with FGDIs perceive their symptoms to be related to consumption of certain foods. In particular, gluten free diet (GFD) and diet with a low consumption of Fermentable Oligosaccharides, Disaccharides, Monosaccharides, and Polyol (FODMAPs) have been proposed for the treatment of these disorders, especially irritable bowel syndrome(IBS). Therefore we want to provide an overview of the available literature on the efficacy of a low FODMAPs diet and a GFD in reducing symptoms associated with functional abdominal pain disorders, focusing on pediatric patients.

**Methods:** We analyzed randomized controlled trials (RCTs), prospective and retrospective studies, systematic reviews and meta- analyses, reporting the efficacy of the FODMAPs diet intervention or GFD in FGIDs patients.

**Results:** In children, there is only one study that reported positive results of a low FODMAPs diet. Compared to typical american diet, children had fewer daily abdominal pain episodes during the low FODMAP diet (P<0.01). For the GFD, there is one randomized Double-Blind Placebo-Controlled Crossover Trial for the Diagnosis of Non-Celiac Gluten Sensitivity (NCGS) in children, which demonstrated that the prevalence of NCGS is 0.36% in patient referred for gastrointestinal symptoms . More studies have been performed on the adults. Most of these studies found an effect of low FODMAPs diet as well as GFD on gastrointestinal symptoms control, however the duration of the trials and the content of FODMAPS or gluten in the diet were all different and there is a lack of standardization of the trials.

**Conclusion:** FODMAPs-restricted diet may be an effective dietary intervention for reducing IBS, and also in children there are promising data .Gluten may contribute to the occurrence of gastrointestinal symptoms in patients with IBS, nevertheless data on the efficacy of a GFD continue to be limited and the long-term efficacy remains doubtful. Further studies are needed to better clarify the role of these restriction diets , which need to be medically indicated and closely monitored by a healthcare provider, since they can lead to nutritional deficiencies especially in pediatric patients.

## A120 Andrological pathologies in pediatric age: preventing infertility

### Matteo Sulpasso (sulpasso.matteo@libero.it)

#### Pederzoli Hospital, Peschiera del Garda (Verona), Italy

**Background**

Pediatric andrology is the branch of medicine that deals with the diagnosis and therapy of male reproductive organs diseases and malformations that may compromise sexual activity and / or fertility. Pediatric andrology includes male pubertal development from birth to puberty and problems related to prevention of diseases connected to sexual and reproductive functions.

Andrological diseases in children are important because, if not treated precociously and adequately, may cause serious repercussions on normal fertility and, sometimes, ease cancer outbreak.

**Materials and methods**

Andrological screening should be inserted within health examinations. The need of such screenings and the necessity that the paediatrician becomes further aware of these aspects derive from a series of epidemiological studies which highlight a high prevalence of neglected and unsolved andrological diseases. The author has developed a new prevention model based on an Andrological Form to be inserted in the periodic medical examination by the family paediatrician.

This project has already been approved by the Italian Society of Paediatrics and the Italian Andrology Society.

**Results**

Pediatric Andrologists, Pediatric Endocrinologists, Pediatric Surgeons and Urologists are all convinced that the well-being of man passes also through sexual health: precocious puberty, delayed puberty, sexually transmitted diseases, varicocele, cryptorchidism and testicular cancer are often preventable and more often curable.

Already at birth and than as children sexual and reproductive health should be checked. The increase of male genital diseases such as varicocele, cryptorchidism, increased incidence of testicular cancer, reduction in semen quality and increase of hypospadias are the expression of one single phenomenon: testicular dysgenesis syndrome (TDS).

It is conceivable that TDS is the consequence of adverse environmental conditions. Data from important epidemiological studies show that 1 out of 3 adolescents has an andrological disease. The first cause of male infertility is varicocele. Varicocele occurs in the development age (paediatric-adolescent age) and more and more cases occur in prepubertal age often associated with testicular hypotrophy. Cryptorchidism is the second leading cause of male infertility. The risk of developing testicular cancer is 3 to 4 times higher in a criptorchide patient. Early treatment of cryptorchidism reduces the risk not only of infertility but also of testicular cancer.

**Conclusions**

These studies and physicians daily experience confirm the absolute need to intervene in the early stages of male development, which is why the pediatrician's role is irreplaceable.

## A121 Neurological functions of the healthy neonate: an overview

### Agnese Suppiej (agnese.suppiej@unife.it)

#### Department of Medical Sciences- Paediatric Section, University of Ferrara, Ferrara, Italy

The perinatal period, defined as the time from the 20th week post-conception to the 28th day of corrected age is crucial for the development of neurological functions. Even in the absence of overt brain damage, neonates may be at risk of neurological impairment leading to developmental disorders later in life. A dedicated and comprehensive neurological evaluation is important to timely suspect brain dysfunction and plan interventions during the appropriate developmental window. To this goal, enormous advancements have been made in the last century. In the early days, infant functioning was associated with the model of reflexes, subsequently, implemented by models looking at more generalized motor functioning. These were, however, poor indicators of higher cerebral functions. A most important advance was the introduction of the concept of state [1]. States were differentiated, structured organizations of the brain, behaviour and physiology. Different responses where elicited by the same stimulus in different states, indicating that the infant’s brain was not reflexive only. A more behavioural orientation was introduced by Brazelton. His approach was innovative because recognized that neonate is able to regulate its own level of arousal and states, to habituate, to attend and orient, to organize his motor acts and to communicate through its behaviour [2]. The psychologist Als [3] further expanded the behavioural approach proposing the Synactive Theory of Newborn Behavioral Organization and Development. She suggested that development is the maturation of the way the child adopt to handle the experiences from environment in order to maintain his homeostasis (the fundamental neurological function for survival), rather than the attainment of individual abilities. Other important advancements were introduced by Dubowitz [4] who proposed a grading system for muscle tone and posture and by Precthl [5] who characterized the endogenously generated complex and spontaneous neonatal movements (General Movements). Bearing in mind the fundamental steps made by the pioneers of modern neonatal neurological examination can help even the busy clinician to implement the traditional examination with the observation of the general movements and behavioural states. The examination may be further improved by including observation of the autonomic and motor stability at rest or during handling, the behavioural states transition and the interaction with the caregiver, inspired by the Als’s theory. The gestalt impression of abnormality will suggest the need for a more formal and specialized evaluation.

**References**

1. Prechtl HFR, Beintema D. The neurological examination of the full term newborn. SIMP/ Heinemann.London;1964.

2. Brazelton T, Nugent K. Neonatal Behavior Assessment Scale, 3rd ed, Mac Keith Press. London;1995.

3. Als H. Toward a synactive theory of development:Promise for the assessment of infant individuality. Infant Ment Health J.1982;3:229–243.

4. Dubowitz LMS, Dubowitz V, Mercuri E. The neurological assessment of the preterm and full-term infant, 2nd ed, Mac Keith Press. London; 1999.

5. Prechtl HFR. Spontaneous motor activity as a diagnostic tool. functional assessment of the young nervous system. Early Hum Dev. 1997;50;148.

## A122 Pain in intellectually disabled children

### Silvia Tajè^1^, Luigi Memo^2^, Angelo Selicorni^1^

#### ^1^U.O.C. Pediatria. ASST-Lariana, Ospedale Sant’Anna, Como, Italy; ^2^ U.O.C. Pediatria. USSL 1 Dolomiti, Ospedale San Martino, Belluno, Italy

##### **Correspondence:** Silvia Tajè (silvia.taje@asst-lariana.it)

Children with intellectual disability experience pain more frequently that healthy children.

In a cross sectional multicentre European study, in 2010, Parkinson et al. estimated a prevalence of pain of 60-73% in children with cerebral palsy[1]. In 2003 on 4 weeks period, Breau et al. recorded that each week 35%-52% of children with intellectual disability have pain. Mean pain duration is longer than 9 hours per week, giving a huge impact on their quality of life. Children with the fewest abilities have more nonaccidental pain[2]. The problem also concern the severity of pain: in a study based on health professionals perception, pain is perceived as mild in 61,1%, as moderate in 25%, as severe in 13,9%[3]. Children with intellectual disability often suffer from chronic conditions and associated diseases that could evoke continuous or recurrent pain, like gastro-esophageal reflux, chronic constipation, muscular contractures, hip dislocation, pathologic bone fractures and tooth problems. Moreover, they frequently need invasive diagnostic or therapeutic procedures that can be painful and cause of stress. Pain in these children is often not recognized or underestimated: most of them have communication difficulties and can’t self-report their sensations, but they show their suffering in different ways, like atypical facial responses, laughing, hypertonicity or increased spasticity, hands clapping, behavioral changes or aggressiveness (head banging, self-biting or other self-injurious behavior). The parents or caretaker know how to recognize every change in their child’s behavior, so they should be consulted regarding their perception of pain. Therefore, currently-used scales for pain assesment in pediatric age are not applicable to cognitively impaired children, but it’s advisable to use specific validated scales for cognitive impairment patients that include pain signs typical for these children, for example the FLACC Revised scale [4]. Pain is often under-treated with lower doses of analgesic or anesthetic drugs, because of possible side effects, pharmacokinetics interactions with other drugs in polytherapy or problems due to comorbidity, like gastro-intestinal or respiratory diseases or epilepsy. Moreover, given the wide variety of causes of intellectual disability, pre-existing medical problems, individual sensitivity and co-medication in these patients, it’s not easy to set up large-scale pharmacological trials. During treatment, measurement of pain is mandatory and essential to determine the effectiveness of therapy. Intellectual disabled children care is complex on many aspects, but every clinician has to recognize, measure and manage pain too, in order to improve the quality of life of these patients and their families.

**References**

1. Parkinson KN, Gibson L, Dickinson HO, Colver AF. Pain in children with cerebral palsy: a cross-sectional multicentre European study. Acta Paediatr. 2010;99:446-51.

2. Breau LM, Camfield CS, McGrath P, Finley GA. The incidence of pain in children with severe cognitive impairments. Arch Pediatr Adolesc Med. 2003;157:1219-1226.

3. Badia M, Riquelme I, Orgaz B, Montoya P. Pain, motor function and health-related quality of life in children with cerebral palsy as reported by their physiotherapists. BMC Pediatr. 2014;14:192.

4. Malviya S, Voepel-Lewis T, Burke C, Merkel S, Tait AR. The revised FLACC observational pain tool: improved reliability and validity for pain assessment in children with cognitive impairment. Paediatr Anaesth. 2006;16:258-65.

## A123 Obesity, new approaches to care: the family, the stigma, the network

### Rita Tanas^1^, Vita Cupertino^2^, Giampaolo De Luca^3^

#### ^1^Pediatric Endocrinologist, Ferrara, Italy; ^2^Community pediatrician, ASP Cosenza, Italy; ^3^Family pediatrician, Cosenza, Italy

##### **Correspondence:** Rita Tanas (tanas.rita@gmail.com)

Is there any development in obesity treatment?

Obesity prevalence keeps rising among poorest countries and minorities in rich countries, during adolescence, and severe forms are increasing. Classic treatments lead to modest results [1] and this calls for the design of longer, complex and expensive studies, which are difficult to be implemented within public health. The acceptance of the treatment among patients is quite low at early stages (<50% of subjects) and it decreases after a year (only <25%) [2-3].

New treatments aim for a multifactorial, rather than a multidisciplinary, and intersectoral project [4], to be carried out jointly by politics, industry, media, and schools, starting with obesity stigma. Stigma – which nowadays is spreading and increasing – concerns, alas, the pediatric field too [5]. Only lately, it has been acknowledged as an obstacle to treatment. It consists of the well-known mechanism of attributing negative qualities to a stranger, based only on features such as gender, religion or race. Nowadays, most discriminations concerns body weighty. Stigma can be the result of a “conscious” or even “unconscious” prejudice, the latter one prevailing among professionals too. Failure to achieve the expected results in obesity treatment leads to disappointment and guilt in pediatricians, who blame families and try to scare them despite the evidence of genetic and environmental factors. Lastly, the patients, especially if stigmatized during childhood, eventually accept the stigma, share it, and mock themselves: the “internalization” phenomenon [6-9]. Stigma has already proved to lead to mental and physical health deterioration (Table 1).

When mockery adsorbs all the energies, the healing process hails to a stop; when it is repeated in time, it nullifies the only good treatment: self-efficacy.

Everyone today, including healthcare professionals, knows how difficult is to heal from obesity. Lack of confidence in the results in addition to mockery makes treatment unsuccessful. On the contrary, professionals who followed an educative, non-judgmental, trust-based, and collaborative approach have seen surprising and unexpected results.

What can we do differently, then?

The path is long and challenging. Above all, it’s urgent to revolutionize the professional training by focusing on equal relationships with families, collaboration between sectors, treatment of stigma through verbal (avoid all words linked with guilt or shame) and non-verbal (more insidious) communication, and a new evaluation of the results (Table 2), based on attitudes more than anthropometric parameters.

**Acknowledgements**

The abstract is presented on behalf of the SIP Adolescent Study Group.

**References**

1. Ells LJ, Rees K, Brown T, Mead E, Al-Khudairy L, Azevedo L, et al. Interventions for treating children and adolescents with overweight and obesity: an overview of Cochrane reviews. Int J Obes (Lond). 2018;42:1823-1833.

2. Mameli C, Krakauer JC, Krakauer NY, Bosetti A, Ferrari CM, Schneider L, et al. Effects of a multidisciplinary weight loss intervention in overweight and obese children and adolescents: 11 years of experience. PLoS One. 2017;12:e0181095.

3. Purromuto S, Tanas R, Romano MA, Musso D, Corsello G. Ricominciare a Curare i bambini in eccesso ponderale con la Consensus SIP-SIEDP. Successi e Problemi di un percorso in Rete fra Pediatria di Famiglia e Ambulatori di 2° Livello a Ragusa. Area Pediatrica [In press].

4. Dietz WH, Solomon LS, Pronk N, Ziegenhorn SK, Standish M, Longjohn MM, et al. An integrated framework for the prevention and treatment of obesity and its related chronic diseases. Health Aff (Millwood). 2015;34:1456-63.

5. Tanas R, Gil B, Caggese G, Baggiani F, Valerio G, Corsello G. Professional stigma on weight in the pediatric care in italy and andalusia: recognize it to successfully treat obesity. J Obes Ther. 2017;1:1.

6. Puhl RM, Himmelstein MS, Quinn DM. Internalizing Weight Stigma: Prevalence and Sociodemographic Considerations in US Adults. Obesity (Silver Spring). 2018;26:167-175.

7. Palad CJ, Yarlagadda S, Stanford FC. Weight stigma and its impact on paediatric care. Curr Opin Endocrinol Diabetes Obes. 2019;26:19-24.

8. Himmelstein MS, Puhl RM. Weight-based victimization from friends and family: implications for how adolescents cope with weight stigma. Pediatr Obes. 2019;14(1).

9. Nutter S, Ireland A, Alberga AS, Brun I, Lefebvre D, Hayden KA, et al. Weight Bias in Educational settings: a systematic review. Curr Obes Rep. 2019. doi: 10.1007/s13679-019-00330-8.

**Table 1 (abstract A123).** Documented consequences of weight stigma on health

1. Increase of depression and anxiety, low self-esteem, risk of suicidal ideas	
2. Increase of unhealthy/extreme eating habits, body image dissatisfaction , eating disorders	
3. Increase of weight, obesity comorbidities and death rate among the population	
4. Decreased motivation toward physical activity, treatment requests, plans to change and self-efficacy	
5. In health care, shorter examinations, faster and less compelling messages, greater physical distance between physician and patient	

**Table 2 (abstract A123).** New tasks to develop in obesity’s Health Network

1. Work with families and support good qualities and intentions	
2. Accept obesity as a disease, indeed a chronic disease, and actually take care of it. Because treatment of all diseases is a health professional’s task, including social and incurable ones.	
3. Accept that once obesity has been established, weight loss results can be small. In fact, even avoiding further weight gains reduces physical and mental comorbidities and improves quality of life.	
4. Become aware of the stigma that pervades us all, deal with it, and reduce it in all areas, by training colleagues, nurses, teachers and media to form a network.	

## A124 Reasons why children should play sport

### Giancarlo Tancredi, Mario Pietropaolo, Roberto Ferrara, Alessandra Favoriti, Caterina Lambiase

#### Dipartimento di Pediatria – U.O.C di Cardiologia pediatrica – Sapienza Università di Roma, Roma, Italy

##### **Correspondence:** Giancarlo Tancredi (francesco.tancredi@gmail.com)

The promotion of sport, physical activity and correct lifestyles in children and adolescents favors the start of virtuous social behaviors, respect for rules and opponents. It is essential to adopt a correct lifestyle that includes regular physical activity, a moderate and balanced diet and excludes the intake of substances that are harmful to the body such as smoking, drugs and alcohol abuse. Physical activity improves the learning abilities and adaptability of adolescents to daily tasks, promotes good emotional control, improved self-esteem and increases socialization skills. From a physiopathological point of view, the practice of regular physical activity in children is an important form of prevention of respiratory and cardiovascular diseases, obesity and metabolic disease. Regular physical activity with an adequate workload reduces minute ventilation and lactic acidosis, improves maximum oxygen consumption (VO2max), strengthens the muscle groups involved during exercise, increases capillary vascularization and the number of mitochondria. In particular, in our previous study we assessed the influence of Body Mass Index (BMI) on cardiopulmonary function in a cohort of asthmatic children. We selected 435 subjects (73.3% boys, means age 12.5 ± 2.5 years). All subject underwent a cardiopulmonary exercise test to measure maximal oxygen uptake (VO2max). According to BMI percentiles children were classified as underweight if <5th percentile, as normal weight (N) if between the 5th and the 85th percentile, as overweight (OV) if between the 85th and the 95th percentile and as obese (OB) if >95th percentile. Overall 415 patients were included in the study. Asthma was reported in 197(47.5%) children: 133 (67.5%) with normal weight, 37(18.8%) were overweight and 27(13.7%) were obese. Non asthmatic children were 218 (52.5%): 172(78.9%) N, 26(11.9%) OV, 20(9.2%)OB. Particularly, VO2max (ml/min/kg) was significantly lower in OB and O than N children, in both asthmatic and non-asthmatic children (Asthmatic:N:=43±8.6,OV=40.6%±8.7,OB=38.3±5; Non Asthmatic:N=51.6±9.9,OV=48.2±9.9, OB=44.5±7.7;F = p < 0.0001). We found an association between high BMI and childhood asthma. Overweight/obese children had lower VO2max respect the normal weight group. Finally, the practice of regular physical activity determines the reduction of risk factors for respiratory and coronary disease, through the reduction of heart rate and arterial pressure at rest, serum triglyceride concentration, intra-abdominal and total body fat and lastly of insulin requirement resulting in improved glucose tolerance.

## A125 Approach to the child with intellectual disability or global developmental delay

### Giovanna Tezza^1^, Ennio Del Giudice^2^

#### ^1^Pediatric Department, Franz Tappeiner Hospital, Merano (BZ), Italy; ^2^Department of Medical Translational Sciences, Section of Pediatrics, Federico II University of Naples, Italy

##### **Correspondence:** Giovanna Tezza (giovanna.tezza@gmail.com)

Global developmental delay (GDD) is a significant delay in two or more of areas of developmental domains: gross and fine motor, speech/language, cognitive, social/personal and daily living activities [1]. Such delays are evaluated through norm-referenced and age-appropriate standardized measures of development [1]. This term is usually reserved for children younger than 5 years of age. Intellectual disability (ID) is characterized by childhood or adolescent performance in standardized cognitive tests of at least 2DSs below average [2]. Intelligence quotient, adaptive behaviour and systems of support afforded the individual are included in the ID evaluation [3].

The etiological diagnoses of GDD and ID overlap and early detection of these disorders is of crucial importance for possible treatment [4]. Nevertheless, they are not always identifiable and the diagnostic rate varies according to the severity of GDD/ID being nearly 80% in cases of severe ID and 24% in mild forms [5]. Flow charts are aimed to limit time-consuming tests [6]. The initial step includes clinical history (family pedigree, pregnancy and birth history) and detailed physical examination. Child’s developmental status evaluation in all domains (gross and fine motor, language, socioemotional and cognitive) can either point toward a specific diagnosis or guide laboratory testing [6]. These steps are crucial because the underlying cause is identified by history and examination alone in about one third of cases while in the remaining the clinical assessment prompts appropriate investigations even though some diagnoses are only possible through investigations [6]. Chromosome microarray (CGH array) and X-fragile test are recognized as a first-line investigation in children with GDD/ID [1,5,7]. When no etiological diagnosis has been identified following history and physical examination, metabolic test panel for inborn errors of metabolism (IEM) is recommended as well as brain imaging (MRI) [8]. TreatableID.org (www.treatable-id.org) contains an algorithm that is regularly updated and describes 81 treatable IDs by their biochemical defects, diagnostic tests, clinical features and treatment options [9]. It has been developed to help clinicians in the work up of these patients. In conclusion, patient’s history and physical examination are still the mandatory first steps. If a specific diagnosis is suspected, investigation to confirm that etiology should be ordered first. This should not delay the referral to rehabilitation services while waiting for the results. Test for treatable inborn errors of metabolism in children with unexplained GDD/ID should be performed, even if clinical suspicion is absent. Genetic exams, in particular CGH array and X fragile tests are first line investigations for unexplained GDD/ID.

**References**

1. Moeschler JB. Medical genetics diagnostic evaluation of the child with global developmental delay or intellectual disability. Curr Opin Neurol.2008; 21:117-22.

2. Helsmoortel C, Vandeweyer G, Ordoukhanian P. Challenges and opportunities in the investigation of unexplained intellectual disability using family-based whole-exome sequencing. Clin Genet 2015;88:140-8.

3. Schalock RL, Luckasson RA, Shogren KA. The renaming of mental retardation: Understanding the change to the term intellectual disability. Intellectual and Developmental Disability, 2007;45:116–124.

4. Jimenez-Gomez A, Standridge SM. A refined approach to evaluating global developmental delay for the international medical community. Pediatr Neurol 2014;51:198–206.

5. American Academy of Neurology. Evaluation of the child with global developmental delay. http//:www.aan.com/guidelines Accessed 20 July 2019.

6. Mithyantha R, Kneen R, McCann E. Current evidence-based recommendations on investigating children with global developmental delay. Arch Dis Child 2017;102:1071-1076.

7. Manning M, Hudgins L, Professional Practice and Guidelines Committee American College of Medical Genetics. Array-based Technology and Recommendations for Utilization in Medical Genetics Practice for Detection of Chromosomal Abnormalities 2010. Genet Med 2010;12:742–745.

8. Bèlanger SA, Caron J. Evaluation of the child with global developmental delay and intellectual disability. Paediatr Child Health 2018;23:403-419.

9. van Karnebeek CD, Shevell M, Zschocke J. The metabolic evaluation of the child with an intellectual developmental disorder: Diagnostic algorithm for identification of treatable causes and new digital resource. Mol Genet Metab 2014;111:428–38.

## A126 Pediatric emergency department overcrowding: review of a rising concern

### Vincenzo Tipo, Sara Viscovo

#### A.O.R.N. Santobono Pausilipon, Naples, Italy

##### **Correspondence:** Vincenzo Tipo (enzotipo@libero.it)

Pediatric emergency department (PED) overcrowding is considered an international crisis, widely involving hospitals all over western countries. It compromises physicians’ work and increases PED length of stay, with higher morbidity rates for patients [1]. While it remains unclear whether PED overcrowding might represent the cause or the effect of rising hospital admissions, these phenomena are indubitably connected [1]. Indeed, the reasons behind this issue are numerous and complex, but an increasing number of accesses (input) and a lack of inpatient beds (output) appear to be crucial factors [2]. Among the elements influencing PED input, nonurgent visits are considered the most important [3]. The misperception of primary care physician’s role has been highlighted as one of the determinants for the larger numbers of low-acuity PED admissions [4,5]. However, flu season is another recognized cause of higher PED flow [3]. On the other hand, the major factor of output induced PED overcrowding is considered to be the hospital bed shortage [3]. There are studies supporting the hypothesis that output is the principal rate‐limiting step in PED crowding [2,6]. In this setting, high hospital census leads to prolonged waits for admitted patients, which in turn leads to PED boarding and decreased ability to place and evaluate new patients. However, there are contradictory evidences suggesting that this might not be the only driving force behind PED crowding and that there could be a significant variability depending on hospital characteristics (such as geographical location) [7,8]. As for its causes, a definitive solution to the problem is still to be found too and PED overcrowding continues to significantly affect both quality and access to care. Instruments to identify and assess PED overcrowding have been proposed, such as the Score for Pediatric Emergency Department Overcrowding (PEDOCS), and markers of PED overcrowding have been described, including measures of patient volume, operational processes at PED and delays in transferring patients to inpatient beds [7]. Feasible solutions for PED overcrowding can include the addition of personnel, the adoption of observation units to increase hospital bed access, specific pathways for nonurgent referrals, ambulance diversion and destination control [3].

**References**

1. Chan M, Meckler G, Doan Q. Paediatric emergency department overcrowding and adverse patient outcomes. Paediatr Child Health. 2017; 22:377–81.

2. Asaro PV, Lewis LM, Boxerman SB. The impact of input and output factors on emergency department throughput. Acad Emerg Med. 2007; 14:235–42.

3. Hoot NR, Aronsky D. Systematic review of emergency department crowding: causes, effects, and solutions. Ann Emerg Med. 2008; 52:126-136.

4. Long CM, Mehrhoff C, Abdel-Latief E, Rech M, Laubham M. Factors influencing pediatric emergency department visits for low-acuity conditions. Pediatr Emerg Care. 2018. doi: 10.1097/PEC.00000000000015535.

5. Haasz M, Ostro D, Scolnik D. Examining the appropriateness and motivations behind low-acuity pediatric emergency department visits. Pediatr Emerg Care. 2018; 34:647–9.

6. Lucas R, Farley H, Twanmoh J, Urumov A, Olsen N, Evans B, et al. Emergency department patient flow: the influence of hospital census variables on emergency department length of stay. Acad Emerg Med. 2009; 16:597–602.

7. Stang AS, McGillivray D, Bhatt M, Colacone A, Soucy N, Léger R, et al. Markers of overcrowding in a pediatric emergency department. Acad Emerg Med. 2010; 17:151–6.

8. Timm NL, Ho ML, Luria JW. Pediatric emergency department overcrowding and impact on patient flow outcomes. Acad Emerg Med. 2008;15:832–7.

## A127 The impact of rheumatic fever in a Province of Central-North Italy

### Elena Tronconi^1^, Erika Miglionico^1^, Andrea Donti^2^, Angela Miniaci^1^, Andrea Pession^1^

#### ^1^Department of Pediatrics, Sant’Orsola-Malpighi Hospital, University of Bologna, Bologna, Italy; ^2^Pediatric Cardiology and Adult Congenital Heart Disease Program, Sant’Orsola-Malpighi Hospital, University of Bologna, Bologna, Italy

##### **Correspondence:** Elena Tronconi (elena.tronconi@studio.unibo.it)

**Background**

Acute rheumatic fever (ARF) is a multisystem complication of group A streptococcal (GAS) infection. The incidence of ARF in the 5-14 years age group varies greatly in different geographic areas, with the lowest incidence in the US and Western Europe (0.5-3 out of 100 000 per year).

In 2015 a new version of Jones Criteria has been published [2], incidence less than 2 out of 100.000 per year was established as low-risk (LR) populations.

Due to the lack of nationwide epidemiologic data in Italy, it is difficult to apply the epidemiologic standard of Jones criteria.

Aim of our study is to estimate the incidence of ARF in a metropolitan area of Central-North Italy and to study the clinical characteristics of the disease in a developed country.

**Materials and Methods**

We retrospectively analyzed the data of all patients with ARF aged 5-14 years old, diagnosed according to the classical and now LR Jones criteria [2], referred to Sant’Orsola-Malpighi Hospital of Bologna from January 2012 to December 2017 and living in the province of Bologna.

**Results**

We identified a total of 34 patients. Every year the annual incidence of ARF was above 2 out of 100.00. The mean incidence was 5.0/100.000 with the highest value in 2013 (10.5 out of 100 000). Carditis was present in 27 patients (79.4% of cases) while chorea in 10 children (29.4%). In 4 patients the diagnosis of ARF was made after the detection of subclinical cardiac. Only 4 patients presented polyarthritis at diagnosis while 17 had polyartralgia and 6 monoarthritis. Ten patients presented with fever (≥38.5°C), 18 children had elevated inflammatory markers as erythrocyte sedimentation rate (ESR) ≥ 60 mm/h and/or C-reactive protein (CRP) ≥3.0 mg/dL.

**Conclusions**

Our study confirms the previous published data of Breda *et al.* [3] and Licciardi *et al.* [4] in other Italian areas supporting the evidence for high risk (HR) in our region. Furthermore, this study shows that in our population only few patients had the classical polyarthritis presentation while monoarthritis and polyarthralgia are more frequent, supporting the need to consider the latter as major criteria. It is important to consider the new HR criteria for arthritis in order not to miss diagnosis. A careful clinical history is needed because the demonstration of GAS infection is often difficult. These data may strengthen the hypothesis to consider our whole country as a HR area for ARF. A nationwide study is mandatory.

**References**

1. Carapetis J, McDonald M, Wilson N. Acute rheumatic fever. Lancet. 2005;366:155-168.

2. Gewitz H, Baltimore R, Lloyd T, Craig S, Stanford S, Carapetis J. Revision of the Jones Criteria for the diagnosis of acute rheumatic fever in the era of Doppler echocardiography: a scientific statement from the American Heart Association. Circulation. 2015;131:1806-1818.

3. Breda L, Marzetti V, Gaspari S, Del Torto M, Chiarelli F, Altobelli E. Population-based study of incidence and clinical characteristics of rheumatic fever in Abruzzo, central Italy, 2000-2009. J Pediatr. 2012;160:832-836.e1.

4. Licciardi F, Scaioli G, Mulatero R, Marolda A, Delle Piane M, Martino S, e al. Epidemiologic Impact of the New Guidelines for the Diagnosis of Acute Rheumatic Fever. J Pediatr. 2018;198:25-28.e1.

## A128 Diabetic ketoacidosis

### Stefano Tumini, Flavia Amaro

#### Department of pediatrics, University of Chieti-Pescara, Chieti, Italy

##### **Correspondence:** Stefano Tumini (stefano.tumini@gmail.com)

The diabetic ketoacidosis is one of the most frequent endocrine diseases in pediatric age all over the world and in Italy [1]. Its prevalence varies from 10 to 70% among the T1DM onset cases [1].

The DKA is a metabolic disorder caused by the total or partial insulin deficiency and it is characterized by hyperglycemia, acidosis and ketosis defined according to ISPAD criteria: blood glucose value > 200 mg/dl; pH < 7,3; HcO3^-^ < 16 mmol/L, ketonemia or ketonuria [2].

The main risk factors of DKA are: age < 5 years old (in particular age below 2 years), a diagnostic error, the membership to ethnic minorities, the absence of health protection system, a low BMI, possible previous infections, a delay in beginning treatment, the family low sociocultural level, eventual psychiatric disorders. In 38% of DKA cases, before the onset a medical examination is done without making the correct diagnosis [1]. On the contrary, protective factors for possible DKA are: high T1DM prevalence in population, high family sociocultural level, a first-degree relative suffering with T1DM. The most feared DKA complication is cerebral edema, that occurs in 0.5-0.9% of all cases [2]. Recently, the “PECARN FLUID Study” [3] has been completed and, on its basis, ISPAD [2] suggests a fluid resuscitaton over 24-48 hours (with 0.45% and 0.9% saline solutions) in patients with a 5-10% deydration level. So, if we consider these range values, there doesn’t seem to be differences between different protocols used to prevent cerebral edema.

**References**

[1] Usher-Smith JA, Thompson MJ, Sharp SJ, Walter FM. Factors associated with the presence of diabetic ketoacidosis at diagnosis of diabetes in children and young adults: a systematic review. BMJ. 2011;343:d4092.

[2] Wolfsdorf JI, Glaser N, Agus M, Fritsch M, Hanas R, Rewers A, et al. ISPAD Clinical Practice Consensus Guidelines 2018: Diabetic ketoacidosis and the hyperglycemic hyperosmolar state. Pediatr Diabetes. 2018;19:155–77.

[3] Kuppermann N, Ghetti S, Schunk JE, Stoner MJ, Rewers A, McManemy JK, et al. Clinical Trial of Fluid Infusion Rates for Pediatric Diabetic Ketoacidosis. N Engl J Med.2018;378:2275–87.

## A129 Sport in the first 6 years of life

### Attilio Turchetta, Federica Gentili

#### Ospedale Pediatrico Bambino Gesu’, Dipartimento Medico Chirurgico Cardiologia Pediatrica, UOSD di Medicina dello Sport, Roma, Italy

##### **Correspondence:** Attilio Turchetta (attilio.turchetta@opbg.net)

There are many studies that document the benefits of physical activity in school-age and adolescent children (6-17 years) as well as in adulthood (> 18 years), evidence that led to the drafting of specific guidelines and specific guidelines policy choices for health protection. In recent years more interest has been placed in analyzing the effects of physical / motor activity even in preschool, from 0 to 6 years [1]. A positive correlation emerged between physical / motor activity in these age groups and multiple health indicators such as musculoskeletal health (bone mineral density, bone mineral content, skeletal area, vitamin D), motor skills (motor skills of basic and purpose, locomotor skills and object control), psychosocial health (self-efficacy, self-esteem, prosocial behavior, quality of life), cognitive development (language development, attention span, executive functioning), various cardiometabolic parameters (pressure , insulin resistance, adiponectin, leptin, tot / HDL cholesterol, triglycerides) [2].

**References**

1. American College of Sports MedicineACSM’s Complete Guide to fitness & Health. Bushman BA, editor. Leeds (UK): Human Kinetics; 2012.

2. American Academy of Pediatrics. Children, adolescent and Media. Pediatrics 2013; 132:958:61

## A130 Safety of treatment and drug therapy: the skill calculation, a pediatric peculiarity

### Liliana Vagliano^1^, Irene Balcet^1^, Federica Vignali^1^, Marilena Bambaci^2^, Fulvio Ricceri^3-4^, Ilaria Bergese^2^

#### ^1^ Department of Paediatrics, University of Turin, Turin, Italy; ^2^ Department of Pediatric, Regina Margherita Children's Hospital, Città della Salute e della Scienza di Torino, University Hospital, Turin, Italy; ^3^ Department of Clinical and Biological Sciences, University of Turin, Italy; ^4^ Unit of Epidemiology, Regional Health Service ASL TO, Grugliasco, Italy

##### **Correspondence:** Liliana Vagliano (liliana.vagliano@unito.it); Ilaria Bergese

**Background**

The process of drug administration is very complex and in the pediatric population it can lead to higher errors than in other age groups [1-5]. The process includes the preparation of the drug to be administered, which may include mathematical calculations in order to obtain a correct and accurate dose ^6-7^. So, mathematical skills are essential in avoiding errors and harm to patients and calculation skills must be acquired and practiced by nursing students [6-8]. The objective of the study is to test the computational skills of students enrolled in Italian Degree Courses in Pediatric Nursing (CLIP) and a sample of nurses of a Northern Italy Children's Hospital.

**Materials and Methods**

A cross-sectional survey was conducted on students enrolled in 7 Italian undergraduate nursing courses and on a sample of pediatric nurses working in a pediatric hospital. The tool used to test calculation skills is a questionnaire built by 7 expert professionals (pharmacologists, pharmacists, pediatricians and nursing professors in the pharmacological field). The questionnaire includes 38 excercises divided into 6 sections, including infusion rates in ml / h and in drops / min, parenteral and enteral administration, concentration transformation from percentages to mg and ml to mg. An abbreviated form with 25 questions was used for professionals. The difference in the scoring of correct answers between the various groups was tested with the Wilcoxon test for the sum of the ranks (two groups) or Kruskal - Wallis (more than two groups). The differences for which the p value was less than 0.05 were considered significant. All tests were two-sides and were performed with the SAS V 9.2 program.

**Results**

Pediatric Nursing Students: 144 questionnaires were collected. The average of the correct answers is 27.08 (± 6.98) on 38 questions. The average of the correct answers varies based on the course CLIP, but also on the basis of the high school of origin, the gender and the year of graduation.Pediatric nurses / nurses professionals: 90 questionnaires were collected (87%). The total average of the correct answers was 21.2 (± 3.6) and the main differences where found among departments (p <0.0001).

**Conclusions**

The results of this study show that there is a lack of computational skills in pediatric nurse and students. It suggests that it is necessary to introduce an effective method for their improvement in order to avoid calculation errors during the preparation of drugs.

**Acknowledgments**

We would like to thank all the coordinators of the Degrees in Paediatric that helped us in the management of this study: Gina Bordone (Novara), Elena Bezze (Milan), Giuliana D’Elpidio (Rome), Laura Fornoni (Genoa), Maria Grazia Greco (Naples), Carmela Otero (Naples) ed Anna Persico (Turin).

We would like to thank Dr. Marisa Sacco and Dr. Laura Odetto for they help as an executive manager.

**References**

1. Otero P, Leyton A, Mariani G, Ceriani Cernadas JM and Patient Safety Committee. Medication errors in pediatric inpatients: prevalence and results of a prevention program. Pediatrics. 2008;122:e737-e743

2. Prot S, Fontan JE, Alberti C, Bourdon O, Farnoux C, Macher MA, et al. Drug administration errors and their determinants in pediatric in-patients. Int J Qual Health Care 2005;17:381-9

3. Chua SS, Chua HM, Omar A. Drug administration errors in paediatric wards: a direct observation approach. Eur J Pediatr. 2010;169:603-11.

4. Gonzales K. Medication administration errors and the pediatric population: a systematic search of the literature. J Pediatr Nurs. 2010;25:555-65.

5. Doherty C, Mc Donnell C. Tenfold medication errors: 5 years’ experience at a university-affiliated pediatric hospital. Pediatrics. 2012;129:916-24.

6. Bagnasco A, Galaverna L, Aleo G, Grunetti AM, Rosa F, Sasso L. Mathematical calculation skills required for drug administration in undergraduate nursing students to ensure patient safety: A descriptive study. Drug calculation skills in nursing students. Nurse Educ Pract. 2016;16:33-39.

7. Harvey S, Murphy F, Lake R, Jenkins L, Cavanna A, Tait M. Diagnosing the problem: using a tool to identify pre-registration nursing students' mathematical ability. Nurse Educ Pract. 2010;10:199-25.

8. McMullan M, Jones R, Lea S. Patient safety: numerical skills and drug calculation abilities of nursing students and Registered Nurses. J Adv Nurs. 2010;66:891-9.

## A131 Recurrent respiratory infections in children with special needs

### Diletta Valentini^1^, Chiara Di Camillo^1^, Nadia Mirante^1^, Valentina Marcellini^2^, Rita Carsetti^2,3^, Alberto Villani^1^

#### ^1^ Pediatric and Infectious Disease Unit, Bambino Gesù Children's Hospital, IRCCS, Rome, Italy; ^2^Immunology Research Area, B-cell development Unit, Bambino Gesù Children's Hospital, IRCCS, Rome, Italy; ^3^Immunological Diagnosis Unit, Bambino Gesù Children's Hospital, IRCCS, Rome, Italy

##### **Correspondence:** Diletta Valentini (diletta.valentini@opbg.net)

**Background**

Life expectancy in children with Down syndrome (DS) has increased significantly over the last decade, but children with DS remain at higher risk of neonatal and infant mortality than children without DS, respectively (1.65% vs. 0.36% and 4% vs. 0.48%). Important causes for increased mortality in DS are congenital heart disease, leukemia, testicular cancer and sepsis. In addition, respiratory tract infection represents the second leading cause of death, in children with DS, up to the age of 18. Children with DS show a high susceptibility to recurrent infections (RI), caused by immune defects and abnormalities of the airways. Our goal was to investigate the effects of Pidotimod on RI prevention in children with DS, comparing immune and clinical parameters before (T0) and after (T1) the treatment of Pidotimod.

**Material and methods**

The study was conducted at the Down syndrome outpatient Center of Bambino Gesù Children’s Hospital, in Rome. We reviewed, retrospectively, the medical records of all children with a positive history for RI during 2016 and 2017 and who received oral prophylaxis of Pidotimod. The study was approved by Bambino Gesù childrens hospital’s Ethics Board, approval number 1753.

**Results**

Thirty-three children met the inclusion criteria (males: 51.5%; average age: 6 years ±SD: 3). We found a significant decrease in the number of children with upper respiratory infections (82% at T0 *vs* 24% at T1; p=0,0001) and with lower respiratory infections (36% at T0 *vs* 9% at T1; p=0.003) after treatment with Pidotimod. We demonstrated also a significant decrease in number of children hospitalized for respiratory infections (18% at T0 *vs* 3% at T1; p=0.03). We measured T and B cells in the peripheral blood and B cell function in *vitro* at T0 and T1. We found that the response to CpG improved at T1. A significant increase of B cells frequency (p=0.0009), B cell proliferation (p=0.0278) and IgM secretion (p=0.0478) were observed in children with DS after treatment. **Conclusions:** our results provided the evidence that Pidotimod may be able to prevent RI in children with DS.

## A132 Vaccination

### Piero Valentini (Piero.valentini@unicatt.it)

#### Pediatrics, Department of Woman and Child Health and Public Health, Fondazione Policlinico Universitario “A. Gemelli”, Università Cattolica del Sacro Cuore, Roma, Italy

The increase in migratory flows towards our country over the last thirty years has led to the arrival of new types of children from geo-political contexts different from the Italian one; many of them might be under-immunised and lack vaccination card, putting them at increased risk of vaccine-preventable diseases. Furthermore, children belonging to foreign families are more likely to undertake trips to their parents' countries of origin, with an increased risk of coming into contact with non-endemic infectious agents in our country. Considering that sub-optimal immunity was registered, in recent years, also in native-born population, the fear for infectious diseases outbreaks is understandable^1^. Hence, the need to create a new healthcare first approach, always in order to facilitate, as soon as possible, the regular access of these children to the paths provided by the National Health Service for the pediatric population. There is no international consensus on the vaccination strategy to follow for recently immigrated child, although the prevalent opinion is to avoid the expenditure of economic resources and to establish or favor migrant immunization as quickly and easily as possible. Different guidelines produced in several countries (USA, UK, Canada, Australia, New Zealand, Italy) share the idea of ​​accepting the vaccinations performed, provided they are validated by official documentation; instead, in subjects without documentation, an age-tailored *catch-up* strategy is appropriate because reduces the interval between doses and number of accesses in health facilities concentrating more vaccinations on the same day. Finally, it is considered useful to carry out an HBV screening only in subjects coming from endemic areas^2^. The Migrant Child Working Group of the Italian Society of Pediatrics, over the years (2002, 2007, 2013) proposed more editions of an Healthcare Approach for Migrant Children Programme, always incorporating international indications on vaccination: now, that Programme needs a review, setting up different strategies for different groups of migrant children, taking into account international data on vaccine immunity, but also the organization problems of our health services and the mistrust sometimes manifested towards vaccination practices; mostly, we need trying to make this particular health problem an opportunity to get better the life conditions of these new italian citizens.

**References**

1. European Centre for Disease Prevention and Control. Public health guidance on screening and vaccination for infectious diseases in newly arrived migrants within the EU/EEA. Stockholm: *ECDC*; 2018.

2. Hui C, Dunn J, Morton R, Staub LP, Tran A, Hargreaves S, et al. Interventions to Improve Vaccination uptake and cost effectiveness of vaccination strategies in newly arrived migrants in the EU/EEA. A systematic review. Int J Environ Res Public Health. 2018;20;15.

## A133 Vulnerable child syndrome and developmental vulnerabilities

### Elena Vanadia, Gianna Palladino

#### Istituto di Ortofonologia, Rome, Italy

##### **Correspondence:** Elena Vanadia (elena.vanadia@libero.it)

One of the current challenges in the childhood and adolescence neuropsychiatric field is the early identification of vulnerabilities, in particular about psycological and behavioral development, also considering the increase in mental disorders (the WHO reports an increase between 10 and 20% in the developmental age) and the anticipation of the age of psychopatology onset. Taking into account both biological and environmental/social factors, variables and specificities of the evolutionary phases must be considered. In fact, in the developmental period (from birth to adolescence) the same symptom or behavior can assume physiological or pathological connotations depending on the age of presentation or its persistence. Hypotheses and theories about development and neuropsychiatric pathologies have always been based on the observation of behaviors, and over time different diagnostic, pathogenetic and therapeutic approaches have followed. Starting from the first observations of doctors and psychoanalysts and arriving at an analysis of what has changed over time can make us understand, both clinically and historically, what risks and what benefits derive from changes in man and his social being. Consequently being able to identify the early signs of vulnerability, especially of psychopathology, becomes the new frontier to counteract the increase in disorders and the anticipation of the age of onset. In this regard, the following may be paradigmatic: in 1964 two American pediatricians, Green and Solnit, and in 1996 Leslie and Boyce, had described the "vulnerable child syndrome" to describe how an 'anxious' affective and educational style can effect behavioral and relational organization, due to an emotional perception of difficulty not so much related to the severity of the child's clinical condition, as to the parents' experience in educating a child perceived as vulnerable. The increase in the survival of premature or perinatal suffering began to result in an increase in parental anxieties compared to the future of the children themselves, an aspect that still today is current and extended to increasingly extreme situations (survival, birth and even fertilization). Finally, theories and observations on those components of anger and aggression that today represent a psycho-behavioral emergency will be described, reaffirming the importance of observation in clinical practice and the complexity underlying expressed behaviors.

## A134 Let's eat! Home prepared, or industrial food?

### Andrea Vania (andrea.vania@uniroma1.it )

#### Centro di Dietologia e Nutrizione Pediatrica, Policlinico Umberto I – Sapienza Università di Roma, Rome, Italy

All around the world there is still a wide debate about pros and cons of commercially prepared vs. home-cooked baby-food [1]. Unfortunately, this debate is often overcharged with ideology, instead of science, thus increasing the gap between supporters and opponents of both preparations. As it happens frequently, the truth lies somewhere in between. Two major and undeniable cons of industrial baby-foods are the high cost (when compared with a similar recipe prepared at home), and the homogeneity of taste, which is always the same, at least until the recipe undergoes some change. Most other disadvantages attributed to commercial baby-foods are ideologically driven and provenly false, at least in Italy and in most European countries, such as: excess of salt and sugar (totally eliminated in Italy by most companies), presence of artificial flavours and preservatives (forbidden by laws), low quality of ingredients (unlikely, if not proven), contributing to general environment’s pollution (jar’s glass and lid’s steel are both perfectly recyclable). The majority of commercial baby-foods provides a correctly energy-dense meal with greater vegetable variety per meal then their home-cooked counterparts. Two negative aspects, on the contrary, are rarely considered: the portion size for meat-based preparations may be too big, and the recent decision from companies to diversify the percentage of meat in their products (20-30-40%) make difficult for families to understand when the preparation can be used for one meal, and when for more. Besides, the jar is often used as a measure for home-prepared homogenized meat, thus leading to exceed paediatrician’s advice. Home prepared foods are for sure cheaper, and provide the child with never the same taste. Nonetheless, Italian (European?) families rarely have a correct eating style, and this affects the quality of the child’s meal: reduced variety of vegetables, and in scarce quantity; excess of proteins; presence of salt; inadequate type/quantity of fats; inappropriate cooking methods. Safety about toxics and pollutants is another big difference between the two types of baby-food, that should be taken into consideration, since legislation for commercial baby-foods is much stricter than for commonly consumed (even bio!) foods. All the above mentioned aspects should be considered –with the help of a wise paediatrician– by families, who should be aware of the risks/advantages of their choices.

**Reference**

1. Carstairs SA, Craig LC, Marais D, Bora OE, Kiezebrink K. A comparison of preprepared commercial infant feeding meals with home-cooked recipes. Arch Dis Child. 2016;101:1037-1042

## A135 We listen and communicate

### Edvige Veneselli (edvige.veneselli@fastwebnet.it)

#### Professor of Child Neuropsychiatry, DINOGMI, University of Genoa, Gaslini Institute, Italy

The comprehensive management of enuresis includes the global knowledge of the disorder and must understand in particular the underlying psychological factors. They are mainly classified as causal, favoring, reactive, coexisting. In the context of neurodevelopmental and child psychiatric disorders, enuresis is more frequent in attention deficit / hiperactivity disorder and is often accentuated by stress; rarely but significantly, the onset of secondary enuresis in school age, specially in a girl, can raise suspicion of posttraumatic stress disorder, also related to abuse.or domestic violence. When we listen the child with enuresis and his parents, it is therefore important to collect the necessary elements to highlight the presence and the role of this factors and disorders in the individual case. In general ayway, listening to the child is aimed at gathering mostly what psychological problems he presents, then how the enuretic disorder lives and if he has feelings of inferiority, rejection, sham. From the parents we listen what problems the child presents at their opinion and specifically how they live the enuretic disorder of the child (included the possibility of consequent mistreatment) and how they think they can help him, and how much they perceive the burden of trouble for him. In our diagnostic-therapeutic observation, in parallel we gradually give information about the enuretic disorder to the child and the family, starting from its frequency and then listing the role of familiarity, habits on drinking, eating and sleeping, with a first return on hygiene related to this. Subsequently we indicate the essential lines of the diagnostic procedure and of the treatment; at the same time we express a prudent confidence in the solution of the disorder. In the following meetings we verify the compliance with the psychoeducational advice provided, the difficulties encountered and any corrections to be implemented. So let's evaluate with parents the opportunity for psychological counseling if problems or concerns have emerged. In this process we like to privilege the relationship with the child so that he can understand, share the various passages; we use drawings, examples, metaphors. And the children feel the commitment on our part and respond with equal commitment and with a particular satisfaction.

## A136 Recurrent respiratory infections. Epidemiology

### Maria Carmen Verga^1^, Michele Fiore^2^, Giovanni Simeone^3^, Marcello Bergamini^4^

#### ^1^Primary care pediatrician, ASL Salerno, Vietri sul Mare. Italy; ^2^Primary care pediatrician, ASL3 “genovese”, Genova, Italy; ^3^Primary care pediatrician, ASL Brindisi, Mesagne, Italy; ^4^Primary care pediatrician, ASL Ferrara, Ferrara, Italy

##### **Correspondence:** Maria Carmen Verga (mariacarmenverga@gmail.com)

Epidemiological data of Recurrent Respiratory Infections (RRIs) are difficult to detect and evaluate, first of all due to a non-uniform definition of this condition. The prevalence of IRRs in the unselected pediatric population can vary between 5 and 25%, especially in preschool [1,2,3], taking into account that on average, in this age group, there can be between 3 and 5 episodes / year. Exceptions are the first 12 months of life, in which it may be considered normal to present up to 6.3 episodes of acute respiratory infection (ARI) [1, 2]. It is estimated that in Italy about 6% of preschool children are affected by RRIs [6].The incidence is related to the various risk factors, even if some are more complex to evaluate or have given discordant results. The age of entry into the community, for example, in some studies appears to be a protective factor, inversely associated with the risk of Influence-Like Illness ILI (at 1 year, adjusted Incidence Rate Ratio-aIRR: 1.4, IC 95%: 1.1–1.9, at 4 years aIRR : 1.1, 95% CI: 0.8–1.4, not significant) [7], in others it does not involve risk differences (P = 0.74) [8]. The incidence of respiratory diseases is, in Italy, comparable to that of Western Europe and different from the global one. [9]. Table 2 shows the DALYs (Disability-Adjusted Life Year) due to the different types of ARIs, by age group and geographical location, therefore it gives us the measure of how much these pathologies in pediatric age affect our health compared to all other possible causes of disability and death. RRIs do not differ in duration and severity from those found in children with a normal number of respiratory diseases. In 80% of the cases the agents responsible for these infections are rhinoviruses, parainfluenza viruses type 1, 2, 3 and 4 and influenza viruses type A and B. The remaining 20% of IRR episodes are caused by β-hemolytic streptococcus of group A. Far more rarely the agents responsible are Haemophilus influenzae, Streptococcus pneumoniae and Moraxella catarrhalis [6]. Very interesting is the dramatic decrease, from 1991 to 2017, of both Pneumococcus pneumoniae and HIB infections, for which universal prevention measures have been prepared, both for RSV and for influenza, for which preventive interventions have been adopted targeted only to subjects at risk. (Table 2) [9].

**References**

1. De Martino M, Balloti S. The child with recurrent respiratory infections: Normal or not? Pediatr. Allergy Immunol. 2007;18 (Suppl. 18):13–18.

2. Toivonen L1, Karppinen S, Schuez-Havupalo L, Teros-Jaakkola T, Vuononvirta J, Mertsola J, et al.. Burden of recurrent respiratory tract infections in children: a prospective cohort study. Pediatr Infect Dis J. 2016;35:e362-e369.

3. De Benedictis FM, Bush A. Recurrent lower respiratory tract infections in children. BMJ 2018;362:k2698.

4. Kusel MMH, de Klerk N, Holt PG, Landau LI, Sly PD. Occurrence and management of acute respiratory illnesses in early childhood. J Paed Child Health. 2007;43:139-46.

5. von Linstow M-L, Kahler Holst K, Larsen K, Koch A, Andersen PK, Høgh B. Acute respiratory symptoms and general illness during the first year of life: a population-based birth cohort study. Ped Pulmonol. 2008;43:584- 93.

6. Fiore M, Napoleone E, Careddu D, Meglio P, Fiocchi A, Cardinale F, et al. Le infezioni respiratorie ricorrenti. I consigli della FIMP. Il Medico Pediatra. 2010;3:9-19.

7. Enserink R, Lugne´ A, Suijkerbuijk A, Bruijning-Verhagen P, Bruijning-Verhagen P, Smit HA, et al. Gastrointestinal and respiratory illness in children that do and do not. attend child day care centers: a cost-of-illness study. PLoS ONE 2014;9(8): e104940.

8. Kaur R, Morris M, Pichichero ME. Epidemiology of acute otitis media in the postpneumococcal conjugate vaccine era. Pediatrics. 2017;140: pii: e20170181.

9. Global Burden of Disease - GBD 2017. http://www.healthdata.org/gbd. Accessed 6 September 2019.

**Table 1 (abstract A136).** RRIs incidence in Italy and in the world (% new cases on all new cases of all age-related diseases)[9]

Respiratory infections (% of total new cases)	<5 years	
	Italy	Global
Lower Respiratory Infections - LRIs	=======	1.73
Upper Respiratory Infections - URIs	74.93	44.38
Otitis	4.18	3.59
Respiratory infections (% of total new cases)	5-14 years	
	Italy	Global
*Lower Respiratory Infections -* LRIs	====	1.61
*Upper Respiratory Infections -* URIs	69.25	46.21
Otitis	1.23	1.37

**Table 2 (abstract A136).** DALYs (Disability Adjusted Life Year) due to the different types of ARI, by age group and geographical location[9]

Italy 5-14 years DALYs /100.000			
Infection	1991	2017	Difference %
*Pneumococcus pneumoniae*	26.52 (15.38-36.08)	9.26 (4.08 – 15.3)	-65.1
RSV	2.39 (1.51-3.54)	1.1 (0.66-1.66)	-53.87
Influenza	1.37 (0.89-2.0)	0.61 (0.39-0.62)	-55.2
<5 years DALYs /100.000			
Infection	1991	2017	Difference%
*Pneumococcus pneumoniae*	491.24 (315.35-661.57)	56.07 (20.89 – 97.1)	-88.59
*Haemophilus influenzae*	113.66 (32.08-209.9)	5.93 (1.35-12.05)	-94.78
RSV	67.18 (39.84-113.18)	13.31 (7.62-21.48)	-80.19
Influenza	10.47 (6.34-16.22)	2.09 (1.27-2.19)	-80.0

## A137 Growth and endocrinological problems in migrant and international adopted children

### Raffaele Virdis (rafvir12@gmail.com)

#### Ambulatorio Caritas and Università di Parma, Parma, Italia

Migrations and international adoptions by themselves do not cause growth or endocrine disorders, but it is not uncommon to find these problems in such children, especially if they came from developing countries (DC). Here we consider only growth, puberty, thyroid and dysvitaminosis D. GROWTH: The migrant child usually grows regularly and at percentiles slightly lower than those of the peers of the countries of arrival but higher than the compatriots who remain at home. These studies have been carried out mostly in countries with average tall statures (North Europe). The weight, expressed as BMI, increases and exceeds the average of the country in which one lives, especially in later generations, with increased incidence of obesity and its serious consequences up to DM2 and metabolic syndrome. All this may depend on various endogenous and exogenous factors that influence growth, such as genetics, puberty (time, duration), nutritional status (malnutrition, dysvitaminosis), psycho-physical stress (abandonment, child labor, violence, migrations and adoptions itself), and endocrine disruptors, including maternal alcohol and drugs in pregnancy. These factors can counteract growth, but the classic endocrine pathologies (GH deficiency in the head) are no more common in migrant children than in native children. PUBERTY. In the case of migration, the behavior is similar to that of growth: it adapts to the characteristics of native peers and is often anticipated compared to compatriots. Precocious puberty is no more frequent than in native peers except in the case of girls adopted by DC, in which it is 10-20 times more frequent. The main causes are the nutritional and psychological improvements (love, parenthood, security, support). All the factors already mentioned for growth are valid also for puberty. THYROID: many children, who come from areas with iodine deficiency, where congenital hypothyroidism also appears to be more frequent, once they take normal iodine amounts they run the risk of developing autoimmune thyroiditis. Moreover, the frequent finding of elevated TSH at arrival must be rechecked over time, observing, but not always, normalization in the following year. RICKETS: the risk is frequent both on arrival and after and it is due to the more pigmented skin and to nutritional and cultural factors and must be prevented and early recognized.

## A138 Pertussis in the first months of life

### Anna Chiara Vittucci, Chiara Di Camillo, Alberto Villani

#### General Pediatric and Infectious Diseases Unit, Bambino Gesù Children’s Hospital, Rome, Italy

##### **Correspondence:** Anna Chiara Vittucci (annachiara.vittucci@opbg.net)

Pertussis is an endemic, underdiagnosed, bacterial respiratory infection caused by Bordetella pertussis*.* Despite a widespread vaccination program, pertussis continues to be a common worldwide infection in paediatric and adult populations peaking every 2 to 5 years. The World Health Organization estimates that in 2016, 139.535 cases were reported, with 89.000 estimated deaths. Even if 95% of reported cases occurred in developing countries, epidemics have recently been reported in Europe, US, Canada and Australia too.

Infants are known to acquire pertussis from adolescents’ and adults’ contacts that return susceptible to the disease because of waning immunity as well as from unvaccinated children. Clinical manifestations may be different depending on age: in previously immunised or infected adolescents and adults pertussis can be atypical and often asymptomatic, with the main symptom being persistent cough. Severe symptoms are common in infants; however, cases with atypical clinical presentations do occur and may be often unrecognized, especially during the winter season, when other respiratory viruses circulate [1]. Serious complications (neurological, respiratory and nutritional), even if relatively rare, can be fatal. Pulmonary complications are in absolute terms the most frequent and are associated with most of the deaths. Risk factors for severe cases are: leukocytosis with lymphocytosis, young age, low birth weight, prematurity and increased pulse and respiratory rates [2]. The cause of deaths in infants is irreversible pulmonary hypertension associated with aggregates of white blood cells in pulmonary arterioles due to leukocytosis with lymphocytosis. No medications provide symptomatic relief from pertussis-associated cough. Antibiotics eliminate Bordetella pertussis from nasopharynx and reduce the risk of transmission; they have not, however been shown to reduce the duration or severity of cough [3]. In infants with extreme leucocytosis, an exchange blood trasfusion must be considered [2]. Recent recommendations made by Italian experts, in accordance with WHO, demonstrate that the best cost-effective intervention in newborns pertussis prevention is the vaccination with dTap vaccine in the pregnant woman, ideally between 27 and 36 weeks’ gestation to optimize transplacental transfer of maternal antipertussis antibodies to the fetus and to protect the newborn in the window time from the birth to the first vaccination. Many studies have shown that vaccinating pregnant women will prevent most infections and virtually all deaths in infants during the first 2 months of life [4].

**References**

1) Vittucci AC, Spuri Vennarucci V, Grandin A, Russo C, Lancella L, et al. Pertussis in infants: an underestimated disease.BMC Infect Dis. 2016;16:414.

2) Cherry JD. The prevention of severe pertussis and pertussis deaths in young infant. Expert Rev Vaccines. 2019;18:205-208.

3) Kilgore PE, Salim AM, Zervos MJ, Schimitt HJ. Pertussis: microbiology, disease, treatment and prevention. Clin Microbiol Rev. 2016;29:449-86.

4) Furuta M, Sin J, NG ESW, Wang K. Efficacy and safety of pertussis vaccination for pregnant women - a systematic review of randomised controlled trials and observational studies. BMC Pregnancy Childbirth. 2017;17:390.

## A139 The pediatric early warning score for clinical monitoring of children

### Enrica Zambelli^1^, Anne Destrebecq^2^, Stefano Terzoni^3^

#### ^1^Head nurse, Maternal and Child Health department, San Paolo teaching hospital, Milan, Italy; ^2^University of Milan, Department of Biomedical Sciences for Health, Milan, Italy; ^3^San Paolo teaching hospital, bachelor school of Nursing, Milan, Italy

##### **Correspondence:** Enrica Zambelli (enrica.zambelli@asst-santipaolocarlo.it)

**Background**

P-Alarm is one of the several tools grouped under the name PEWS (Paediatric Early Warning Scores/Systems) available over the world. PEWS records and measures the child’s vital signs (heart and respiratory rates, pressure) and behaviors. The parameters are weighted and the overall score provides indications on clinical deterioration of the child, often allowing prevention of severe events. Traditionally, PEWS are found in the Emergency Department or Intensive Care Units, but pediatric inpatient units are beginning to use them as well. The aims of this study were: 1) to verify the applicability of the P-Alarm in a Paediatric unit, 2) to adapt and validate it in the pediatric inpatient unit of the San Paolo hospital.

**Materials and methods**

the P-Alarm was chosen after a literature review (PubMed, Cochrane Library and CINAHL) and back-translated after written consent from the author. All nurses working in the outpatients unit were trained to use the tool; all children admitted between Dec 12, 2016 and Jan 27, 2017 underwent monitoring with the P-Alarm.

**Results**

129 children were enroled (72 males, 57 females, age 4 weeks-18 years). The P-Alarm showed an excellent validity and good internal consistence (Cronbach’s alpha=0.80). A total of 1547 observations were conducted. In 18 cases (2 children) clinical deterioration was present; the P-Alarm correctly identified 17 cases. One child showed significant alteration of the P-Alarm, which led to activation of the medical emergency team. In this case, the P-Alarm allowed early detection of clinical deterioration.

**Conclusions**

the Italian version of the P-Alarm is a valid, simple and reliable tool for clinical monitoring of childred admitted to inpatient units. It allows early recognition of severe deterioration, thus saving precious time for early medical intervention.

## A140 Errors in pediatric emergency: review of the literature and evaluation of the proposed strategies

### Stefano Zampogna^1^, Maria DeFilippo^1^, Antonella Centonze^2^

#### ^1^Pediatric Department- Policlico S. Matteo, Pavia, Italy; ^2^Pediatric Department - Azienda Ospedaliera Pugliese Ciaccio, Catanzaro, Italy

##### **Correspondence:** Stefano Zampogna (stezampogna@gmail.com)

**Background**

Emergency pediatric care is recognized as an environment with a high risk of error due to numerous factors. In 1999 the medical institute reported "To Err is Human", in this article it is stated that medical errors remain one of the main causes of morbidity and mortality throughout the United States and that every year between 44,000 and 98,000 American patients die due to errors committed by health personnel [1,2.3]. Between 2007 and 2009, the British National Health Service - NHS - conducted a national survey to study the phenomenon of errors in the pediatric field. This study found that 79% of incidents involving children and 94% of those involving newborns occurred in the critical area and that the therapeutic error in pediatric patients was 3 times that found in adult patients [4,5,6,7,8,9]. The purpose of our work is to re-evaluate the literature on errors reported in pediatric emergency departments in order to identify which strategies are proposed to reduce human error.

**Method**

We have carried out a systematic review of the works published on Pubmed over the past 5 years using the following keywords: errors, pediatric emergency. 439 articles were found, of these 45 are reviews and 67 clinical studies. Full texts were available for 67 publications.

**Conclusion**

We have summarized the recommendations and strategies reported in the literature to reduce errors in pediatric urgency. We believe that error prevention should be considered a priority of health care and we believe that simulation can prove to be a fundamental educational method to reach this goal: reduce errors and improve safety.

**References**

1. Doherty C, Mc Donnell CG. Tenfold medication errors: 5 years' experience at a university-affiliated pediatric hospital. Pediatrics 2012;129:916-24.

2. Kaufmann J, Laschat M, Wappler F. Medication errors in pediatric emergencies-a systematic analysis. Dtsch Arztebl Int .2012;109:609−16.

3. Ghaleb AM, Barber N, Franklin BD,Yeung VVS, Khaki Z et al. Systematic review of medication errors in pediatric patients. Ann Pharmacother. 2006;40:1766-76.

4. Selbst SM, Levine S, Mull C, Bradford K, Preventing medical errors in pediatric emergency medicine. Pediatr Emerg Care. 2004;20:702-9.

5. Benjamin L, Frush K, Shaw K, Shook JE, Snow SK, American Academy of Pediatrics Committee on Pediatric Emergency Medicine, et al.. Pediatric medication safety in the emergency department. Pediatrics. 2018;71:e17-e24.

6. Dreisinger N, Zapolsky N. Ethics in the Pediatric Emergency Department: When mistakes happen: an approach to the process, evaluation, and response to medical errors. Pediatr Emerg Care. 2017;33:128-131.

7. Unbeck M, Lindemalm S, Nydert P, Ygge BM, Nylén U, Berglund C, et al. Validation of triggers and development of a pediatric trigger tool to identify adverse events. BMC Health Serv Res. 2014;14:655.

8. Hirata KM, Kang AH, Ramirez GV, Kimata C, Yamamoto LG. Pediatric weight errors and resultant medication dosing errors in the emergency department. Pediatr Emerg Care. 2017:35:637-642.

9. Lalande J, Vrignaud B, Navas D, Levieux K, Herbreteau B, Guillou A, et al. A prospective observational study of medication errors in a pediatric emergency department. Arch Pediatr. 2018;25:355-358.

## A141 Antibiotic prescription profile in pediatrics

### Federica Zanetto^1^, Antonio Clavenna^2^

#### ^1^Associazione Culturale Pediatri, Italy; ^2^Laboratory for Mother and Child Health, Department of Public Health, Istituto di Ricerche Farmacologiche Mario Negri IRCCS, Milan, Italy

##### **Correspondence:** Federica Zanetto (federica.zanetto@virgilio.it)

Antibiotics represent the most widely prescribed therapeutic agents in the paediatric population. During 2017, 41% of outpatient Italian children and adolescents received one or more antibiotic prescriptions. The prevalence was greater in preschool-age children (50%) and decreased afterward with increasing age [1]. Italian children and adolescents have a 3-fold higher likelihood of receiving an antibiotic prescription than their peers living in the Netherland or UK. Moreover, nearly 1 out of 5 outpatient children treated with antibiotics in Italy receive a cephalosporin, while this class is scantly prescribed out-of-hospital in other European countries [2]. Differences exist at regional and local level in the antibiotic prescription profile. The South of Italy is characterized by an higher prescription prevalence and a greater use of cephalosporins and macrolides [3]. Amoxicillin, the first choice antibiotic drug for the most common infections in the paediatric population, is scantly prescribed, covering 20% of antibiotic prescriptions, but with wide differences between and within regions. In Lombardy region a better quality of antibiotic prescriptions was observed in local health units where paediatricians have been engaged in educational and training programmes. No differences were found in the qualitative profile between family paediatricians and emergency department physicians. It is worth to be noticed that only in 6 out of 56 emergency departments in Lombardy region amoxicillin was prescribed to more than 50% of children treated with antibiotic for pharyngotonsillitis.

In conclusion, more should be done to improve rational use of antibiotics in the paediatric population, and educational interventions including health professionals and parents are strongly needed.

**References**

1) Osservatorio Nazionale sull’impiego dei Medicinali. L’uso degli antibiotici in Italia. Rapporto Nazionale 2017. Agenzia Italiana del Farmaco. 2019. https://www.aifa.gov.it/documents/20142/0/Rapporto-L%27uso_degli_antibiotici_in_Italia_2017.pdf/3ae31357-45c6-b327-8996-2f99e6e80090 Accessed 8 August 2019.

2) Clavenna A, Bonati M. Differences in antibiotic prescribing in paediatric outpatients. Arch Dis Child. 2011;96:590-5.

3) Piovani D, Clavenna A, Cartabia M, Bonati M; Antibiotic Collaborative Group. The regional profile of antibiotic prescriptions in Italian outpatient children. Eur J Clin Pharmacol. 2012;68:997-1005.

4) Piovani D, Clavenna A, Cartabia M, Bortolotti A, Fortino I, Merlino L, et al Assessing the quality of paediatric antibiotic prescribing by community paediatricians: a database analysis of prescribing in Lombardy. BMJ Paediatr Open. 2017;1:e000169.

